# Post-stroke acute heart failure in patients with large vessel occlusion undergoing endovascular treatment: A pooled analysis of individual patient data from multicenter studies with mediation analysis

**DOI:** 10.1371/journal.pmed.1004752

**Published:** 2026-07-07

**Authors:** Liyuan Chen, Jiaxing Song, Changwei Guo, Linyu Li, Tao Xu, Chen Gong, Liping Huang, Shuyu Jiang, Lin Gao, Xinyu Li, Gang Wu, Xue Wang, Thanh N. Nguyen, Jeffrey L. Saver, Yangmei Chen, Wenjie Zi, Chang Liu

**Affiliations:** 1 Department of Neurology, The Second Affiliated Hospital, Chongqing Medical University, Chongqing, China; 2 Department of Cardiology, The Second Affiliated Hospital, Chongqing Medical University, Chongqing, China; 3 Department of Neurology, Xinqiao Hospital and The Second Affiliated Hospital, Army Medical University (Third Military Medical University), Chongqing, China; 4 Institute of Medicine and Equipment for High Altitude Region, College of High-Altitude Military Medicine, Army Medical University (Third Military Medical University), Chongqing, China; 5 Department of Neurology, Boston Medical Center, Boston University Chobanian and Avedisian School of Medicine, Boston, Massachusetts, United States of America; 6 Department of Neurology and Comprehensive Stroke Center, David Geffen School of Medicine at UCLA, Los Angeles, California, United States of America; South African Medical Research Council, SOUTH AFRICA

## Abstract

**Background:**

Cardiac complications rank among the leading contributors to poor outcomes in ischemic stroke patients along with the neurological impairment, while the risk stratification and prognostic significance of post-stroke acute heart failure (PSHF) remain poorly characterized. This study aimed to investigate the incidence, predictors, and impacts of PSHF in patients with large vessel occlusion stroke (LVO) undergoing endovascular treatment (EVT). Given that cardioembolic stroke inherently involves underlying cardiac pathology, a secondary aim was to test whether the effect of stroke severity on PSHF was modified by cardioembolic etiology and whether PSHF mediated the effect of stroke severity on functional outcome in these patients.

**Methods and findings:**

In a pooled analysis of individual patient data from four multicenter prospective studies conducted in China between January 2014 and June 2023, we included 3,415 patients with LVO who underwent EVT. The primary outcome was very poor functional outcome, defined as 90-day modified Rankin Scale (mRS) 5–6. Multivariable regression models, interaction testing, and mediation analysis were used, with adjustment for clinically relevant covariates including demographic characteristics, vascular risk factors, baseline stroke severity, imaging characteristics, and treatment-related factors. PSHF developed in 278 patients (8.14%), with its incidence reaching peak at 1 day after stroke onset. PSHF was significantly associated with a higher rate of very poor outcome (62.23% versus 31.08%, adjusted odds ratio (aOR) 3.09, 95% confidence interval (CI) [2.25, 4.24]). A significant interaction was observed between cardioembolism and the baseline National Institutes of Health Stroke Scale (NIHSS) score (*p* for interaction = 0.016). Moderate-to-severe stroke significantly increased the risk of PSHF in patients with cardioembolic stroke (aOR 1.91, 95% CI [1.28, 2.87]), but not in those with non-cardioembolic stroke (aOR 0.97, 95% CI [0.81, 1.82]). Mediation analysis showed that PSHF mediated 7.70% (95% CI [2.40, 12.40]) of the effect of moderate‑to‑severe stroke on very poor outcome among cardioembolic patients. The main methodological limitations were the pooled design using studies with different protocols and the potential for residual unmeasured confounding.

**Conclusions:**

PSHF was significantly associated with very poor outcome in LVO patients undergoing EVT. Moderate-to-severe cardioembolic LVO substantially elevated the risk of PSHF, with PSHF partially mediating the adverse prognostic impact of stroke severity. Early risk assessment and monitoring for PSHF may optimize management in this high-risk population.

## Author summary

**Why was this study done?:**

- Acute heart failure is a common cardiovascular emergency, but the frequency and timing of post-stroke acute heart failure (PSHF) remain poorly understood in patients with large vessel occlusion (LVO) stroke who undergo endovascular treatment (EVT).
- Although EVT could restore blood flow in LVO patients, many patients still experienced poor outcome, which highlighted the need to better understand the risk stratification and prognostic significance of PSHF.
- Because cardioembolic stroke often involves pre-existing cardiac abnormalities, we wanted to understand whether patients with more severe cardioembolic stroke are especially vulnerable to developing acute heart failure.

**What did the researchers do and find?:**

- We analyzed data from 3,415 stroke patients across four multicenter studies in China, and found that 278 patients (about 8%) developed PSHF within the first week, with most cases occurring on the first day.
- Patients who developed PSHF were much more likely to die or have severe disability requiring continuous care than those who did not develop this complication.
- Among patients with cardioembolic stroke, moderate-to-severe stroke was linked to a markedly higher risk of PSHF, and this complication partly explained the association between moderate-to-severe stroke and poorer outcome.

**What do these findings mean?:**

- PSHF is a serious complication in LVO patients receiving EVT and is strongly linked to worse recovery.
- Patients with moderate-to-severe cardioembolic stroke may need closer heart monitoring and more careful management during the early period after stroke treatment.
- These findings should be interpreted with caution because the study combined data from different studies, and factors that were not measured may have affected the results.

## Abstract 3

In a pooled analysis of individual patient data, Liyuan Chen and colleagues find that post-stroke heart failure is associated with very poor outcome, and is linked to severity of cardioembolic stroke.

## Introduction

In the endovascular treatment (EVT) era, despite the fact that the recanalization rate for ischemic stroke patients with large intracranial vessel occlusion (LVO) is over 90%, less than 50% achieve favorable outcomes [1]. Alongside neuronal damage, cardiac complications rank among the leading causes of poor outcomes after stroke [2]. Acute heart failure is one of the most common cardiovascular emergencies, marked by rapidly evolving and highly characteristic clinical manifestations [3]. However, the incidence, risk factors, and impacts of post-stroke acute heart failure (PSHF) on outcomes among LVO patients undergoing EVT remain poorly characterized [4].

Despite limited research, available data indicated that PSHF predominantly occurred within seven days after stroke onset [5]. The study conducted by Ishiguchi and colleagues, which pooled multiple studies conducted between 1994 and 2006, reported that the incidence of PSHF was approximately 1.40% [6]. However, recent updates to acute heart failure diagnostic guidelines and refinement of highly sensitive indicators have revealed a PSHF incidence of approximately five percent in contemporary studies [7,8]. Nevertheless, these updated studies were single-center investigations with limited sample size that neither addressed the treatment modalities nor accounted for variations in occlusion vessel size among ischemic stroke patients [8]. Given that LVO carries the poorest prognosis of all ischemic stroke subtypes, and that EVT could substantially enhance patient recovery, investigating the roles of PSHF in patients with LVO undergoing EVT is crucial for optimizing periprocedural management strategies and further improving outcomes [9].

The etiology of ischemic stroke may influence the risk of PSHF [10]. Cardioembolic stroke, typically arising from atrial fibrillation or other cardiac sources, often reflects pre-existing cardiac structural abnormalities or dysfunction [8]. These cardiac vulnerabilities may predispose patients to acute heart failure under pathological stress [2]. However, whether stroke severity modifies the association between cardioembolic etiology and the development of PSHF remains unexplored in the EVT era, as stroke severity is closely linked to the degree of pathological stress. PSHF may subsequently contribute to worse functional outcomes through hemodynamic instability and secondary hypoperfusion of vulnerable cerebral tissue [11]. We therefore hypothesized that PSHF mediated part of the effect of stroke severity on poor outcome in cardioembolic stroke patients, representing a promising modifiable target, and performed mediation analysis accordingly. In addition, preclinical studies have established that systemic inflammation following ischemic stroke contributes to myocardial injury and worsens cardiac dysfunction, suggesting a potential therapeutic role for anti-inflammatory agents [12]. The MARVEL trial, which randomized methylprednisolone versus placebo in LVO patients undergoing EVT, provided an opportunity for exploratory assessment of the effects of early corticosteroid use on PSHF incidence and subsequent clinical outcomes in a randomized framework [13].

Therefore, in a pooled analysis of BASILAR registry [14], DEVT trial [15], RESCUE-BT trial [16], and MARVEL trial [13], we aimed to characterize the incidence and temporal pattern of PSHF among patients with LVO undergoing EVT. We further evaluated the association of PSHF with 90-day functional outcome and assessed whether cardioembolic etiology modified the relationship between stroke severity and PSHF risk. We hypothesized that PSHF would be associated with very poor functional outcome after EVT and would partly mediate the adverse association between moderate-to-severe stroke and functional outcome in patients with cardioembolic stroke.

## Methods

### Study design and patients

In the current study, we performed an individual patient-level analysis for LVO patients undergoing EVT based on our previous research including the Endovascular treatment for acute basilar artery occlusion study (BASILAR, ChiCTR1800014759, https://www.chictr.org.cn/) [14], Direct endovascular thrombectomy versus combined intravenous thrombolysis and endovascular thrombectomy for patients with acute large vessel occlusion in the anterior circulation trial (DEVT, ChiCTR-IOR-17013568, https://www.chictr.org.cn/) [15], Endovascular treatment with versus without tirofiban for patients with LVO stroke (RESCUE-BT, ChiCTR-IOR-17014167, https://www.chictr.org.cn/) [16], and Methylprednisolone as adjunctive to endovascular treatment for acute large vessel occlusion (MARVEL, ChiCTR2100051729, https://www.chictr.org.cn/) [13]. The design and results of these studies have been described previously (S1 File), with patients included across 144 centers in China from 2014 to 2023. The BASILAR study included patients with a symptomatic and radiologically confirmed acute basilar occlusion within 24 hours. The DEVT trial assessed the efficacy and safety of EVT alone versus alteplase plus EVT among LVO patients. The RESCUE-BT trial was a prospective, double-blind, randomized clinical trial of intravenous tirofiban versus placebo for LVO receiving EVT within 24 hours. The MARVEL trial was a prospective, double-blind, randomized clinical trial of methylprednisolone versus control among patients with LVO who received EVT within 24 hours.

Among all patients in the BASILAR registry and the intention-to-treat population in the other three trials, patients meeting the following inclusion criteria were included: (1) LVO confirmed by digital subtraction angiography, magnetic resonance angiography, or computed tomography angiography; (2) received EVT; (3) premorbid modified Rankin Scale (mRS) less than 3. Exclusion criteria were (1) minor stroke (National Institutes of Health Stroke Scale [NIHSS] at admission ≤5) [17]; (2) missing 90-day follow-up; and (3) absence of key baseline clinical and procedural characteristics. The flowchart of screening eligible patients was summarized in the flowchart provided in Fig A in S1 Text.

This study adheres to the Strengthening the Reporting of Observational Studies in Epidemiology (STROBE) guideline (S1 STROBE Checklist). A description of our planned analyses and changes made during peer review is included in the supporting information (S2 Text).

### Ethics approval and consent

This secondary analysis used data from human participants in the BASILAR registry (ChiCTR1800014759, https://www.chictr.org.cn/), DEVT trial (ChiCTR‐IOR‐17013568, https://www.chictr.org.cn/), RESCUE-BT trial (ChiCTR-IOR-17014167, https://www.chictr.org.cn/), and MARVEL trial (ChiCTR2100051729, https://www.chictr.org.cn/). The protocols of the original studies were approved by the Xinqiao Hospital Ethics Committee and the ethics committees of the participating centers. Written informed consent was obtained from participants or their legally authorized representatives in the original studies. The original studies were conducted in accordance with the Declaration of Helsinki. The present analysis used de-identified data from the original studies and did not involve new participant recruitment, participant contact, or collection of new identifiable information. No directly identifying information or identifiable participant images were included in this manuscript. Therefore, no additional informed consent was required for this secondary analysis.

### Definition of post-stroke acute heart failure and outcomes

PSHF was diagnosed according to the 2021 ESC guideline on heart failure and ESC Practical Guidance on Natriuretic Peptide Concentrations [18,19]. In patients presenting with new‑onset cardiopulmonary symptoms or signs suggestive of acute heart failure, natriuretic peptide (B-type Natriuretic Peptide/N-terminal pro-B-type Natriuretic Peptide, BNP/NT‑proBNP) was measured promptly upon clinical suspicion, typically within the initial 24-hour diagnostic window in accordance with the Chinese clinical guideline for acute heart failure [20]. Blood samples were processed in the clinical laboratories of the participating centers following standardized protocols and stringent quality control procedures, and all results were standardized to picogram per milliliter (pg/mL).

The diagnostic criteria required simultaneously elevated natriuretic peptides (BNP >400 pg/mL or NT-proBNP exceeding age-specific thresholds: 450 pg/mL for age <50 years, 900 pg/mL for 50–75 years, and 1,800 pg/mL for >75 years) and a new-onset or severe worsening of heart failure symptoms after stroke onset (at least 3 out of 6 symptoms or signs of heart failure had to be present: orthopnoea, paroxysmal nocturnal dyspnoea, fatigue, pulmonary rales, peripheral oedema, and gut congestion) after exclusion of alternative explanations [18]. Differential diagnosis from competing cardiopulmonary conditions is detailed in Method A in S1 Text [18,19,21]. For patients unable to communicate due to aphasia or altered consciousness, diagnosis relied on elevated natriuretic peptides meeting ESC thresholds plus ≥3 findings from an alternative set of 6 objective signs including pulmonary rales, peripheral oedema, gut congestion, elevated jugular venous pressure, hepatojugular reflux, or a third heart sound, without requiring patient-reported symptoms [18,19,21]. Pre-existing chronic heart failure was defined as a documented heart failure diagnosis before the index stroke and chronic use of heart failure medications before stroke onset [22]. It was captured as a baseline comorbidity distinct from PSHF. Patients with PSHF were stratified by pre-existing chronic heart failure status to distinguish de novo acute heart failure from acute decompensation of chronic heart failure. Given that PSHF predominantly occurred in the acute phase of ischemic stroke based on prior literature, the current study focused on PSHF cases occurring within the first week after stroke onset or last known well [7,23]. Two investigators (C.L. and L.Y.C.) independently screened and diagnosed PSHF. Discrepancies were resolved by consensus discussion or a third investigator (W.J.Z.) when consensus could not be reached. These investigators were blinded to 90-day functional outcomes and treatment assignment in trials. Inter-rater agreement was assessed across the entire cohort of 3,415 patients (*κ* = 0.94, 95% CI [0.91, 0.96]).

Conducted by the same research group, the registry and the trials featured standardized outcome assessments, with the primary outcome of 90-day mRS evaluated by blinded, trained personnel, ensuring consistency in methodology and oversight [13–16]. Given that acute heart failure substantially increases the risks of mortality and severe disability, we selected mRS 5–6 as the primary outcome to capture the severe impacts of PSHF [24]. Specifically, mRS 5 represents a distinct clinical state in which patients are completely bedridden and incontinent, requiring continuous nursing care, with medical costs and family caregiving burdens substantially exceeding those of mRS 0–4 [25]. This endpoint aligns with emerging literature recognizing mRS 5–6 as a critical threshold for identifying the devastating consequences of acute ischemic stroke [26,27]. The secondary outcomes included functional independence at 90 days defined as a mRS score of 0–2; independent ambulation at 90 days, defined as a mRS score of 0–3; and the level of disability at 90 days (range, an mRS score of 0 [no symptoms] to 6 [death]). The NIHSS score at 5–7 days (or discharge if earlier) was also assessed. The demographic, clinical variables and outcomes were collected by investigators blinded to the outcomes of interest and diagnosis of PSHF from the databases of the BASILAR registry, the DEVT trial, the RESCUE-BT trial, and the MARVEL trial.

### Combining individual participant data

Data from the one registry and three trials were combined into a harmonized dataset. The studies were reviewed to identify common variables (baseline and outcomes), and a harmonized dataset was assembled. Outcome definitions in each trial were reviewed to ensure consistency. The retained cohort was incorporated into subsequent data analyses. We compared the observed variables between excluded and included patients to assess the potential for selection bias.

### Statistical analysis

Demographic factors, medical history, and baseline clinical characteristics were compared using the Chi-squared test or Fisher’s exact test for categorical variables and the Mann–Whitney *U* test for continuous variables. As detailed in Method B in S1 Text, these outcomes were analyzed as binary variables including very poor outcome (mRS 5–6 at 90 days), functional independence (mRS 0–2 at 90 days), independent ambulation (mRS 0–3 at 90 days) and mortality; as ordinal variable for 90-day mRS distribution; and as continuous variable for NIHSS at 5–7 days or at early discharge.

#### Primary analysis.

To account for potential heterogeneity across the four component studies and clustering within centers, we employed mixed-effects models as the primary analytical framework across all outcomes. For binary outcomes including very poor outcome, functional independence, and independent ambulation, we used mixed-effects logistic regression with study source and center as random intercepts, reporting adjusted odds ratios (aOR). For mortality, we used mixed-effects Cox proportional hazards regression with the same random effects structure, reporting hazard ratios (HR). For ordinal outcome (90-day mRS distribution), we applied mixed-effects ordinal logistic regression, reporting common odds ratios (cOR), with the proportional odds assumption tested by the Brant test. If this assumption was violated (Brant test *P* < 0.050), we implemented a Win Ratio analysis using stratified inverse probability of treatment weighting (IPTW) weights. This stratified IPTW‑weighted Win Ratio simultaneously accounts for center and study heterogeneity through stratification, addressing confounding, and handles ordinal outcome without requiring the proportional odds assumption [28]. For continuous outcomes of NIHSS at 5–7 days or at early discharge, we used linear mixed-effects models, reporting *β* coefficients.

#### Covariate selection.

Covariates for multivariable adjustment were pre-specified based on clinical knowledge and established causal relationships, guided by temporal precedence (Method B and Fig B in S1 Text). The following variables were included as potential confounders: age, sex, baseline NIHSS score, baseline Alberta Stroke Program Early CT Score (ASPECTS), history of atrial fibrillation, pre-existing chronic heart failure, premorbid mRS, occlusion site, expanded Thrombolysis in Cerebral Infarction (eTICI) grade, stroke etiology (Trial of Org 10172 in Acute Stroke Treatment, TOAST classification), and onset to recanalization time. Both TOAST and eTICI were retained based on their central causal roles, regardless of their *p* values in baseline comparisons. Sensitivity analyses using hierarchical adjustment strategies confirmed the validity of this pre‑specified set, as detailed in the Method C in S1 Text.

#### Interaction analysis between NIHSS and cardioembolism.

The primary model included a pre-specified linear interaction between continuous baseline NIHSS and cardioembolic etiology. This interaction was based on the premise that cardioembolic stroke inherently involves underlying cardiac pathology, which may modify the impact of neurological injury on cardiac outcomes, as detailed in the Introduction section. To characterize the clinical significance of this interaction, we plotted the adjusted odds ratio for PSHF comparing cardioembolic versus non-cardioembolic stroke across the NIHSS spectrum following the methodology established by the HERMES collaboration [29]. The solid curve indicates the best linear fit and dashed curves indicate 95% confidence intervals. The threshold of statistical significance for the etiology-specific effect was identified where the lower bound of the 95% CI crossed 1.00. To verify this interaction, we conducted two additional analyses including a test of nonlinear interaction using restricted cubic splines with three knots and a test of interaction using NIHSS categorized into 5‑point increments (5–10, 10–15, 15–20, 20–25, and >25) according to previous literature [30]. To account for multiplicity, we performed both Bonferroni and False Discovery Rate (FDR) corrections on these three interaction tests.

#### Mediation analysis.

Mediation analysis was performed following a guideline for reporting mediation analyses (AGReMA, S2 AGReMA Checklist) to assess and quantify the extent to which PSHF mediated the association between stroke severity and poor outcome in cardioembolic patients [31]. As an exploratory analysis, we also conducted mediation analyses in non-cardioembolic patients to assess the specificity of the observed mediation effect. Interpretation relies on five key identification assumptions: temporal precedence between exposure, mediator, and outcome; no unmeasured confounding for the relationships between exposure and outcome, between exposure and mediator, and between mediator and outcome; and no confounders of the mediator and outcome relationship that are affected by the exposure.

The causal structure underlying the mediation analysis was specified using a DAG (Fig C in S1 Text), which illustrated the assumed relationships among exposure (moderate-to-severe stroke severity), mediator (PSHF), outcome (very poor outcome), and baseline confounders. The DAG confirmed that only pre-exposure (T0 in Method B in S1 Text) variables were adjusted for, while post-treatment variables (T1 in Method B in S1 Text) were not included in the models to prevent overadjustment for variables potentially lying on the causal pathway between exposure and mediator. We estimated the proportion mediated using nonparametric bootstrap (1,000 simulations). Sensitivity analyses included (1) adjustment for time-varying confounders at T1 and (2) rho-sensitivity analysis for unmeasured confounding. To account for multiple comparisons, FDR and Bonferroni corrections were applied to the mediation results after adjustment for time-varying confounders. No formal sample size calculation was performed for the mediation analysis. However, with 1,489 cardioembolic patients and 278 PSHF events, the study had >80.00% power to detect a small mediation effect (proportion mediated >5%) at *α* = 0.05.

#### Propensity score matching.

Propensity score matching (PSM) was applied to match subjects with a similar distribution of confounders to create novel cohorts with a different status of PSHF [32]. Based on the “MatchIt” package, PSM was performed with a 1:1 matching based on the nearest neighbor matching algorithm with a caliper width of 0.20 of the propensity score (Fig D in S1 Text). After PSM, mixed-effects models were applied to investigate the roles of PSHF on outcomes.

#### Subgroup and sensitivity analyses.

For subgroup analysis, we further investigated the heterogeneity in association between PSHF and the primary outcome within the following subgroups: age (<65 versus ≥65 years old), sex (female versus male), baseline NIHSS score (≤16 versus >16), ASPECTS (<6 versus ≥6), stroke etiology, and time from last known well to recanalization (≤360 versus >360 min), and the presence of pre-existing chronic heart failure (with versus without). Given the clinical importance and the high prevalence of pre-existing chronic heart failure in the PSHF group, the association between the pre-existing chronic heart failure and outcomes were also evaluated based on mixed‑effects model. We performed both Bonferroni and FDR corrections on these interaction tests.

For sensitivity analyses, given that our pooled analysis combined data from four studies with different protocols and recruitment periods, we assessed the impact of between‑study heterogeneity on our findings. First, we adjusted for data source as a fixed-effect covariate in the multivariable model to account for potential confounding by study-specific characteristics. Second, to assess whether the association between PSHF and outcomes was consistent across studies, we included study × PSHF interaction terms in mixed-effects models and used likelihood ratio tests to compare models with and without these interaction terms. Pooled estimates of PSHF were reported using sample size-weighted averages only in the absence of substantial effect heterogeneity. Third, to evaluate whether trial‑specific interventions influenced our main findings, we examined the effect of randomized treatment allocation on outcomes within each trial. We fitted separate logistic regression models including an interaction term between randomized intervention and PSHF, adjusted for the same covariates in the primary analysis, to estimate treatment effects and test for interactions with PSHF.

Motivated by the potent anti‑inflammatory effects of methylprednisolone and the significant interaction between baseline NIHSS score and cardioembolic etiology, we conducted two exploratory analyses across four subgroups defined by baseline severity and etiologic subtype in the MARVEL trial [33]. First, to assess the effect of methylprednisolone on PSHF incidence, we used a time‑dependent Cox model with randomization time as time zero. This approach leveraged the 3‑day sustained regimen in MARVEL that created time‑varying exposure and was not feasible in the single‑exposure RESCUE‑BT or DEVT trials. Second, to evaluate treatment effect of corticosteroid among patients who developed PSHF, we applied logistic regression adjusted for covariates from the primary analyses. We controlled for multiple comparisons across subgroups using the FDR and Bonferroni corrections. Given the post hoc nature of these analyses and the small sample size within this subgroup, results are intended solely for hypothesis generation and not as evidence of efficacy.

All statistical analyses were performed using R software (version 4.4.2). Results were considered statistically significant at two-tailed *p* < 0.05. No artificial intelligence tools or technologies were used to generate, edit, or analyze the study or article content. The authors take full responsibility for the final manuscript.

## Results

### Patient characteristics

**Fig 1:**
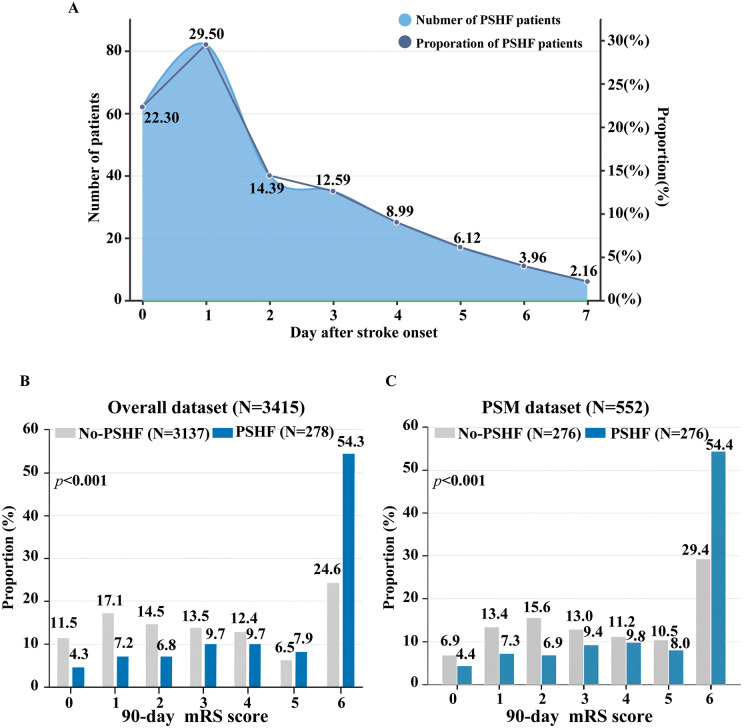
Association of post-stroke acute heart failure (PSHF) with 90-day functional outcomes after stroke. **(A)** The incidence of PSHF after stroke onset. **(B)** The distribution of the modified Rankin Scale (mRS) score at 90 days among all patients stratified by the presence of PSHF. **(C)** The distribution of the mRS at 90 days among novel cohort constructed by propensity score matching (PSM). The *p* values were calculated using the *χ*^2^ test to compare the proportions of patients with very poor outcomes (mRS 5–6) between the PSHF and no-PSHF groups.

Of 3,500 patients initially meeting inclusion criteria, 3,415 (97.57%) were included in the final analysis (Fig A in S1 Text). Missing 90-day follow-up data occurred in 7 patients. The rate of missing 90‑day follow‑up data was comparable between the registry and RCTs (0.00% versus 0.25%, *p* = 0.362). Patients excluded due to missing key baseline clinical or procedural characteristics had similar available baseline characteristics to those included in the analysis (all *p* > 0.050, Table A in S1 Text). Baseline characteristics of patients included in the four studies were presented in Table B in S1 Text. Among included patients, 8.14% developed PSHF. The occurrence of PSHF reached peak level at one day after stroke onset and decreased gradually (Fig 1A). The baseline characteristics of patients are shown in Table 1. Compared to LVO patients without PSHF, PSHF patients were older (median 74.00 [IQR 66.00, 79.00] versus 67.00 [58.00, 75.00], *p* < 0.001), with a higher proportion of female patients (54.68% versus 38.19%, *p* < 0.001), atrial fibrillation (68.71% versus 33.57%, *p* < 0.001), and pre-existing chronic heart failure (44.60% versus 5.26%, *p* < 0.001). The etiology of stroke in patients with PSHF was most often cardioembolism (75.90% versus 40.74%, *p* < 0.001). We also observed lower median (IQR) ASPECTS (6.00 [4.00, 8.00] versus 7.00 [5.00, 8.00], *p* < 0.001), and higher median (IQR) baseline NIHSS (20.00 [17.00, 23.00] versus 18.00 [15.00, 21.00], *p* < 0.001) in PSHF compared to no-PSHF patients.

**Table 1:** Baseline features of patients stratified by the presence of post-stroke acute heart failure (PSHF).

	All (*N* = 3,415)	No-PSHF (*N* = 3,137)	PSHF (*N* = 278)	*p*
Age, median [IQR], year	68.00 [58.00, 75.00]	67.00 [58.00, 75.00]	74.00 [66.00,79.00]	<0.001
Sex, *n* (%)				<0.001
Male	2,065 (60.47)	1,939 (61.81)	126 (45.32)	
Female	1,350 (39.53)	1,198 (38.19)	152 (54.68)	
Baseline NIHSS score, median [IQR]	18.00 [15.00, 22.00]	18.00 [15.00, 21.00]	20.00 [17.00,23.00]	<0.001
Medical history, *n* (%)				
Hypertension	2,094 (61.32)	1,919 (61.17)	175 (62.95)	0.560
Diabetes mellitus	697 (20.41)	635 (20.24)	62 (22.30)	0.414
Pre-existing chronic heart failure	289 (8.46)	165 (5.26)	124 (44.60)	<0.001
Atrial fibrillation	1,244 (36.43)	1,053 (33.57)	191 (68.71)	<0.001
Previous ischemic stroke	572 (16.75)	525 (16.74)	47 (16.91)	0.942
Prestroke modified Rankin scale, *n* (%)				0.028
0	3,168 (92.77)	2,921 (93.11)	247 (88.85)	
1	184 (5.39)	160 (5.10)	24 (8.63)	
2	63 (1.84)	56 (1.79)	7 (2.52)	
Occlusion site, *n* (%)				0.182
Internal carotid artery	802 (23.48)	724 (23.08)	78 (28.06)	
M1 segment	1,637(47.94)	1,517(48.36)	120(43.17)	
M2 segment	360(10.54)	334(10.65)	26(9.35)	
Basilar artery	616(18.04)	562(17.92)	54(19.42)	
Baseline ASPECTS, median [IQR]	7.00 [5.00, 8.00]	7.00 [5.00, 8.00]	6.00 [4.00, 8.00]	<0.001
Stroke etiology, *n* (%)				<0.001
Large artery atherosclerosis	1,503 (44.01)	1,452 (46.29)	51 (18.35)	
Cardioembolism	1,489 (43.60)	1,278 (40.74)	211 (75.90)	
Other	124 (3.63)	122 (3.89)	2 (0.72)	
Unknown	299 (8.76)	285 (9.09)	14 (5.04)	
Intravenous thrombolysis	879 (25.48)	801 (25.53)	69 (24.82)	0.830
eTICI, *n* (%)				0.274
0	199 (5.83)	185 (5.90)	14 (5.04)	
1	25 (0.73)	25 (0.80)	0 (0.00)	
2a	172 (5.04)	155 (4.94)	17 (6.12)	
2b	725 (21.23)	661 (21.07)	64 (23.02)	
2c	456 (13.35)	428 (13.64)	28 (10.07)	
3	1,838 (53.82)	1,683 (53.65)	155 (55.76)	
Workflow times, median [IQR], min				
Onset to puncture time	339.00 [225.00,565.00]	340.00 [225.00,571.00]	305.00 [210.50, 516.00]	0.057
Onset to recanalization time	430.00 [306.00, 663.00]	433.00 [310.00,670.00]	388.50 [292.50,584.30]	0.020

Data are presented as median [interquartile range (IQR)] for continuous variables and *n* (%) for categorical variables. *p* values were calculated using the Mann–Whitney *U* test for continuous variables and the *χ*^2^ test or Fisher’s exact test for categorical variables, as appropriate.

After PSM, baseline characteristics between these two groups were balanced, with details shown in Fig D and Table C in S1 Text. A total of 552 patients with or without PSHF were evaluable for the matched-pairs analysis with the multivariable method.

### Clinical outcomes according to the presence of PSHF

The distribution of the 90-day mRS score in the overall population is presented in Fig 1B. Patients with PSHF had a higher rate of very poor outcomes compared to those without PSHF (62.23% versus 31.08%; adjusted odds ratio (aOR) 3.09, 95% CI [2.25, 4.24]; *p* < 0.001). After additionally accounting for between-study heterogeneity and center clustering using mixed-effects models, PSHF remained significantly associated with adverse outcomes across all measures (Table 2). For the primary outcome, PSHF was associated with increased risk of very poor outcome (aOR 3.09, 95% CI [2.25, 4.24]; *p* < 0.001) and mortality (adjusted hazard ratios (aHR) 2.05, 95% CI [1.67, 2.52]; *p* < 0.001). Functional independence was lower in the PSHF group (mRS 0–2, aOR 0.35, 95% CI [0.24, 0.51]; *p* < 0.001). Early neurological status evaluated by NIHSS at 5–7 day or at early discharge was also poorer in those PSHF patients (*β* coefficient 6.51, 95% CI [4.97, 8.05]; *p* < 0.001). In the propensity score matched cohort analyzed with mixed-effects models, results were consistent with the primary analysis (Fig 1C and Table 2).

**Table 2:** Adjusted association between post-stroke acute heart failure and outcomes.

Outcomes	Before matching							PSM		
	All (*N* = 3,415)	No-PSHF (*N* = 3,137)	PSHF (*N* = 278)	Treatment Effect	Effect value [95% CI]	Adjusted effect value [95% CI]	*p*	Effect value [95% CI]	Adjusted effect value [95% CI]	*p* ^a^
**Primary outcome**										
**Modified Rankin scale score of 5–6 at 90 days, *n* (%)** ^a^	2,267 (66.38)	975 (31.08)	173 (62.23)	Odds ratio	3.86 [3.00, 5.05]	3.09 [2.25, 4.24]	<0.001	2.42 [1.71,3.44]	2.97 [2.00, 4.42]	<0.001
**Secondary outcome**										
**Score on the modified Rankin scale at 90 days, no. of wins/total no. of pairs (%)** ^b^	705,588/ 872,086 (80.9%)	192,259/ 872,086 (22.0%)	513,329/ 872,086 (58.9%)	Win ratio	0.36 [0.24, 0.52]	0.42 [0.32, 0.52]	<0.001	0.46 [0.27, 0.92]	0.53 [0.41, 0.68]	<0.001
**Modified Rankin scale score of 0–3 at 90 days, *n* (%)** ^a^	1,850 (54.17)	1,772 (56.49)	78 (28.06)	Odds ratio	0.29 [0.22, 0.38]	0.37 [0.26, 0.52]	<0.001	0.39 [0.27, 0.56]	0.30 [0.20, 0.46]	<0.001
**Modified Rankin scale score of 0–2 at 90 days, *n* (%)** ^a^	1,401 (41.02)	1,350 (43.03)	51 (18.35)	Odds ratio	0.29 [0.21, 0.40]	0.35 [0.24, 0.51]	<0.001	0.33 [0.22, 0.49]	0.26 [0.16, 0.41]	<0.001
**Mortality within 90 days, *n* (%)** ^a^	922 (27.00)	771 (24.58)	151 (54.32)	Hazard Ratio	2.55 [2.13, 3.05]	2.05 [1.67, 2.52]	<0.001	1.82 [1.40, 2.37]	1.95 [1.49, 2.55]	<0.001
**NIHSS at 5–7 day or at early discharge, median [IQR]** ^^a^,^^c^	12.00 [4.00,26.00]	11.00 [3.00,23.00]	23.00 [10.00,36.00]	β coefficient	8.17 [6.57, 9.78]	6.51 [4.97, 8.05]	<0.001	5.81 [3.38, 8.24]	6.02 [3.89, 8.14]	<0.001

^a^Unadjusted models included no covariates. Adjusted mixed-effects models were controlled for age, sex, history of atrial fibrillation, pre-existing chronic heart failure, premorbid modified Rankin Scale Score, occlusion site, baseline Alberta Stroke Program Early CT Score, baseline National Institutes of Health Stroke Scale, stroke etiology (Trial of Org 10172 in Acute Stroke Treatment), expanded Thrombolysis In Cerebral Infarction, and onset to recanalization time, with study source and medical center as random intercepts. The *p* values were obtained from the corresponding unadjusted or adjusted models.

^b^Unadjusted models included no covariates. Adjusted models were estimated using stratified inverse probability of treatment weighting, with weights derived from propensity score models including age, sex, history of atrial fibrillation, pre-existing chronic heart failure, premorbid modified Rankin Scale Score, occlusion site, baseline Alberta Stroke Program Early CT Score, baseline National Institutes of Health Stroke Scale, stroke etiology (Trial of Org 10172 in Acute Stroke Treatment), expanded Thrombolysis In Cerebral Infarction, onset to recanalization time, study source and medical center. The *p* values were obtained from the corresponding unadjusted or adjusted analysis.

^c^Were missing for 5 patients.

### Predictors for the presence of PSHF

Among demographic variables, after univariable and multivariable analysis, older age (aOR 1.03, 95% CI [1.02, 1.05]; *p* < 0.001), history of atrial fibrillation (aOR 1.58, 95% CI [1.09, 2.30]; *p* = 0.017) and pre-existing chronic heart failure (aOR 10.40, 95% CI [7.50, 14.42]; *p* < 0.001) were associated with the development of PSHF (Table D in S1 Text). In addition, higher baseline NIHSS score (aOR 1.04, 95% CI [1.01, 1.07]; *p* = 0.003), lower ASPECTS (aOR 0.91, 95% CI [0.85, 0.98]; *p* = 0.008), and the etiology of cardioembolism (aOR 2.10, 95% CI [1.37, 3.21]; *p* < 0.001) were detected to be associated with the increased incidence of PSHF.

### Interaction between NIHSS and cardioembolism in association with PSHF

**Fig 2:**
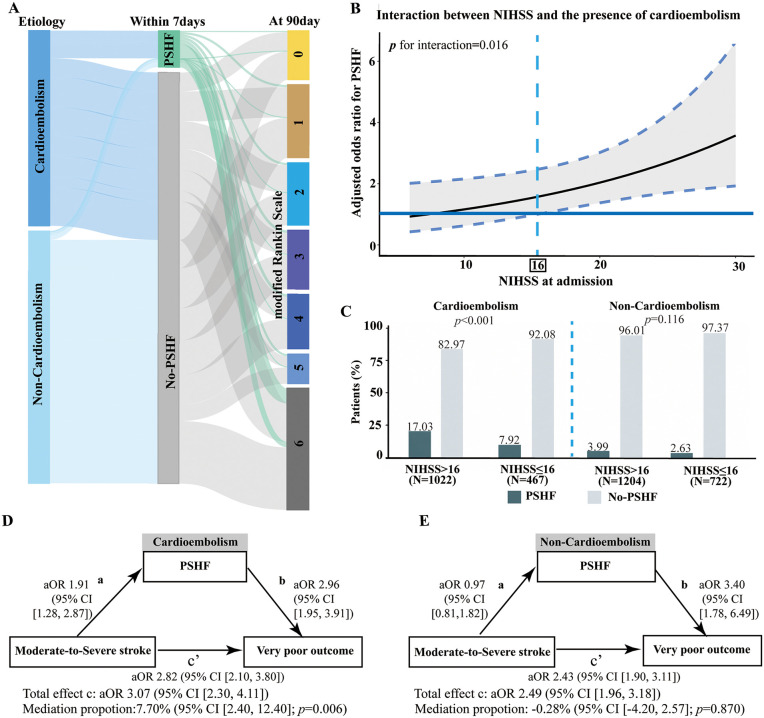
The interaction between stroke severity and cardioembolic etiology in association with post-stroke acute heart failure (PSHF). **(A)** Sankey diagram for the association between PSHF and 90-day outcomes. **(B)** Association of baseline National Institutes of Health Stroke Scale score (NIHSS) with the effect of cardioembolic etiology on PSHF risk. The solid curve indicates the best linear fit between the adjusted odds ratio for PSHF in cardioembolic vs. non-cardioembolic stroke across continuous baseline NIHSS. The dashed curves indicate 95% confidence intervals. The lower bound of the 95% CI crosses 1.00 (vertical red line, nominal *p* for interaction = 0.016, false discovery rate-adjusted *p* = 0.048, Bonferroni-adjusted *p* = 0.048). The nominal p value for interaction was obtained from logistic regression model including a linear interaction term between continuous baseline NIHSS score and cardioembolic etiology. False discovery rate- and Bonferroni-adjusted *p* values were calculated to account for multiple interaction tests. **(C)** The distribution of PSHF among included patients stratified by the stroke severity and the etiology of cardioembolism. The *p* values were calculated using the *χ*^2^ test to compare the proportions of PSHF across different populations. **(D)** The mediation analysis for PSHF among cardioembolic patients. The adjusted Odds ratio (aOR) and 95% confidence intervals (CI) for paths a, b, c, and c′ were obtained from logistic regression models corresponding to each path. The 95% CI and *p* values for the indirect effect and proportion mediated were estimated using nonparametric bootstrap resampling with 1,000 simulations. **(E)** The mediation analysis for PSHF among non-cardioembolic patients. The aOR and 95% CI for paths a, b, c, and c′ were obtained from logistic regression models corresponding to each path. The 95% CI and *p* values for the indirect effect and proportion mediated were estimated using nonparametric bootstrap resampling with 1,000 simulations.

Fig 2A illustrated the temporal trajectory of PSHF diagnosis within 7 days and the corresponding 90-day functional outcomes in patients stratified by the presence of PSHF. The modifying effect of cardioembolic etiology on PSHF risk varied continuously with stroke severity (Fig 2B). The transition from a non-significant to a significant etiology-specific effect occurred at an NIHSS score of 16, where the lower bound of the 95% confidence interval crossed the null threshold, and this effect continued to strengthen progressively through higher severity scores. The linear interaction between continuous baseline NIHSS and cardioembolism was nominally significant (*p* = 0.016) and remained significant after Bonferroni correction (*p* = 0.048) and FDR correction (*p* = 0.048). In contrast, neither the nonlinear interaction using restricted cubic splines (*p* = 0.953) nor the categorical interaction using clinical cutpoints (*p* = 0.227) was significant, with all corrected *p* values exceeding 0.050 (Table E in S1 Text).

Patients were stratified into four subgroups according to stroke severity (NIHSS >16 versus ≤16) and etiology (cardioembolism versus non-cardioembolism, Fig 2C). Moderate-to-severe stroke significantly increased the risk of PSHF in patients with cardioembolic stroke (aOR 1.91, 95% CI [1.28, 2.87], Fig 2D), but not in those with non-cardioembolic stroke (aOR 0.97, 95% CI [0.81, 1.82], Fig 2E). Among patients with moderate-to-severe cardioembolic LVO, the prevalence of PSHF reached 17.03%, representing 62,.59% of all identified PSHF cases in the cohort (Fig E in S1 Text). Mediation analysis in cardioembolic patients showed that moderate-to-severe stroke indirectly increased the risk of poor outcome through PSHF, accounting for 7.70% (95% CI [2.40, 12.40]; *p* = 0.006) of the total effect (Fig 2D). In contrast, among non-cardioembolic patients, the mediation effect was not significant (proportion mediated −0.28%, 95% CI [−4.20, 2.57]; *p* = 0.870, Fig 2E). Sensitivity analyses indicated that these findings were insensitive to unmeasured confounding (Fig F in S1 Text) and time‑varying confounding (Table F in S1 Text).

### Subgroup and sensitivity analysis

The fixed‑effects models (Table G in S1 Text) and fixed‑effects models with additional adjustment for data source (Table H in S1 Text), presented as a sensitivity analysis, yielded results consistent with the primary analysis. To further evaluate the stability of our findings, we included study×PSHF interactions in the mixed-effects model. The pooled estimates of PSHF remained highly consistent with the very poor outcome with the *p* value for likelihood ratio tests higher than 0.050 (Table I in S1 Text). The pharmacological intervention in each trial showed no significant effect on PSHF incidence across trials (Table J in S1 Text), and randomized treatments demonstrated no significant interaction with PSHF status on functional outcomes in any trial (Table K in S1 Text). In addition to the very poor outcome, we found that PSHF was also significantly associated with lower utility-weighted mRS scores (Table L and Method D in S1 Text), and consistently reduced quality-of-life (Table M and Method D in S1 Text).

**Fig 3:**
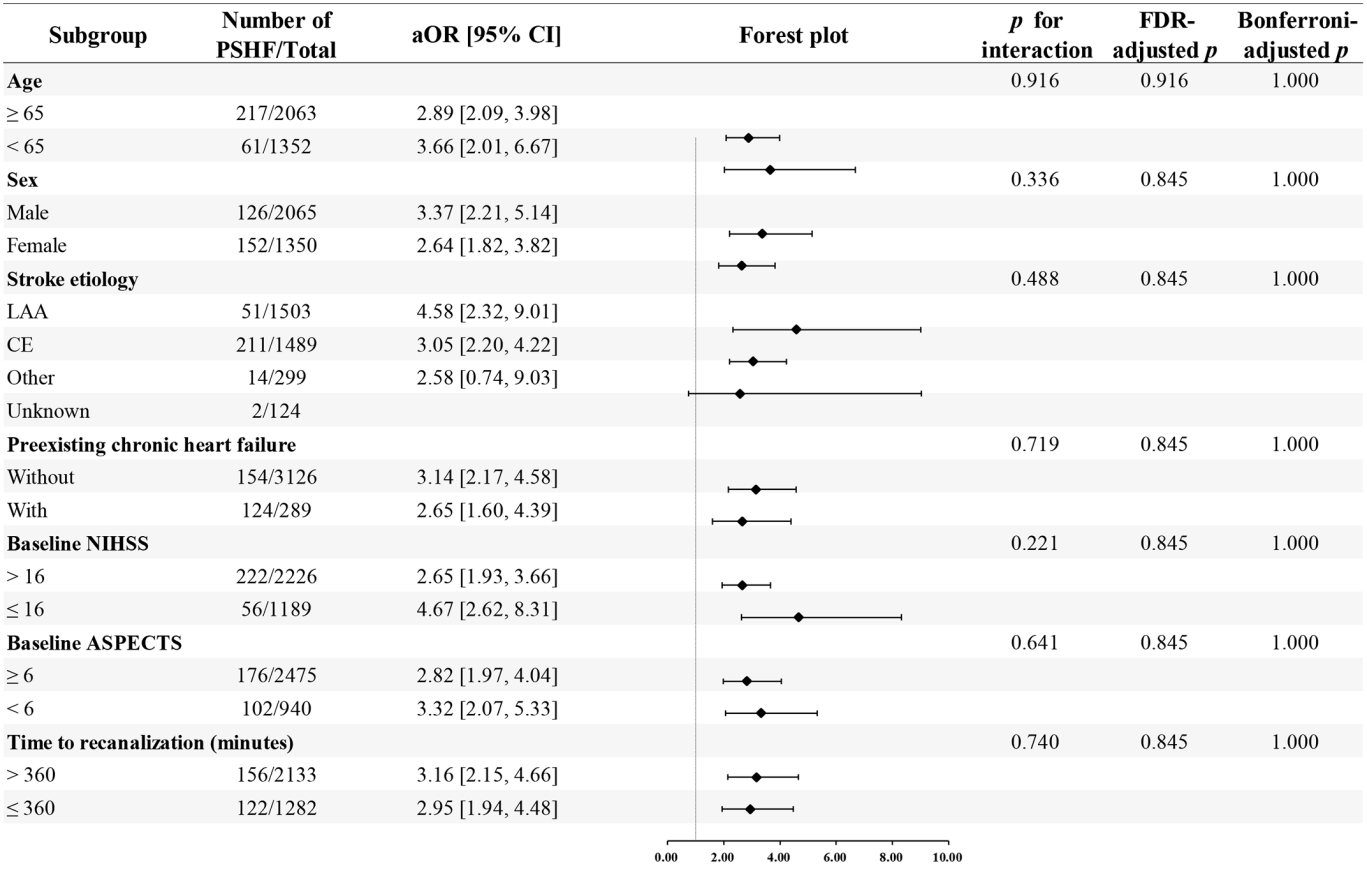
Subgroup analyses for the association between post-stroke acute heart failure (PSHF) and very poor outcome. Very poor outcome was defined as a modified Rankin Scale score (mRS) of 5–6 at 90 days. The *p* values for interaction were obtained from mixed-effects logistic regression models. Multiple comparisons were adjusted using the false discovery rate and Bonferroni methods. No significant interactions were observed between PSHF and any subgroup after correction for multiple comparisons.

Subgroup analyses were based on the full dataset. There was no interaction by age, sex, NIHSS, eTICI, and ASPECTS between the PSHF and no-PSHF group with regard to very poor outcome (Fig 3). Consistent results were observed across all secondary outcomes (all *p* for interaction after correction >0.050). The association between PSHF and very poor outcome (mRS 5–6 at 90 days) remained significant in both patients without pre-existing chronic heart failure (aOR 3.14, 95% CI [2.17, 4.58]) and those with pre-existing chronic heart failure (aOR 2.65, 95% CI [1.60, 4.39]), without significant interaction (*p* for interaction = 0.719; FDR‑adjusted *p* for interaction = 0.845; Bonferroni‑adjusted *p* for interaction = 1.000). We also investigated the impacts of cerebral hemorrhage events on the incidence of PSHF (Fig G in S1 Text). Our findings indicated that neither any cerebral hemorrhage (aOR 1.31, 95% CI [0.94, 1.81]; *p* = 0.108) nor symptomatic cerebral hemorrhage (aOR 1.42, 95% CI [0.92, 2.21]; *p* = 0.114) was associated with the occurrence of PSHF.

Among patients stratified by the cardioembolic stroke etiology and the stroke severity in the MARVEL trial, methylprednisolone was not associated with reduced PSHF incidence (aOR 0.92, 95% CI [0.53, 1.62]; *p* = 0.785, Table N in S1 Text). In a post hoc exploratory analysis of PSHF patients in the MARVEL trial with moderate-to-severe cardioembolic LVO, methylprednisolone showed a potential signal for improved outcomes (aOR 0.33, 95% CI [0.11, 0.85]; *p* = 0.027) that did not reach conventional statistical significance after multiplicity correction (FDR‑adjusted *p* = 0.054, Bonferroni‑adjusted *p* = 0.054, Table O in S1 Text).

## Discussion

This multicenter study represented the systematic investigation into the incidence and prognostic significance of PSHF among LVO undergoing EVT. The major findings were (1) the prevalence of PSHF was nearly 8.14%, with its occurrence reaching the peak on the first day after stroke onset; (2) PSHF was correlated with higher rate of very poor outcome, reduced functional independence, and elevated 5–7 day NIHSS; (3) the risk of PSHF was substantially increased in moderate-to-severe LVO caused by cardioembolism; (4) among these cardioembolic stroke patients, 7.70% of the effect of moderate‑to‑severe stroke severity on very poor outcome was mediated by PSHF.

Among cardiac disorders after stroke, the PSHF is characterized by its distinct clinical manifestations and comparatively high incidence rate [5]. In contrast to studies conducted by Shima and colleagues and Heuschmann and colleagues which included ischemic stroke patients across all vessel sizes, our study detected a higher frequency of PSHF by focusing solely on LVO patients, who presented with more severe neurological deficits, indicated by the much higher baseline NIHSS scores and lower ASPECTS [7,8]. Consistent with prior literature, our study found that cardioembolism was a significant predictor of PSHF [34]. The higher prevalence of cardioembolism in Western populations may partially account for the lower incidence of PSHF observed in our study compared to the 13.30% reported by Hays and colleagues [35]. The heterogeneity in reported incidence of PSHF across studies may originate from the multifactorial determinants, in particular, clinical severity and the underlying etiology for ischemic stroke.

Based on the significant interaction between baseline NIHSS and the presence of cardioembolic etiology in relation to PSHF, we proposed the “double-hit” hypothesis for the development of PSHF, as shown in Fig H in S1 Text. The first hit is the cardiac vulnerability in patients with existing cardiac structural, rhythm, or coronary vascular disorders, which form the structural basis for cardioembolic stroke and the development of acute heart failure [36]. The second insult is the secondary injury to heart from moderate-to-severe LVO. The moderate-to-severe LVO could lead to an imbalance of the autonomic nervous system, the hypothalamic–pituitary–adrenal axis and systemic inflammation response, which would increase the myocardial oxygen consumption and cardiac load or pose injury to cardiomyocytes, thereby exacerbating the cardiovascular stress response and promoting the occurrence of PSHF [37–39]. Consistent with this hypothesis, the incidence of PSHF was nearly tripled in cardioembolic LVO with moderate-to-severe severity comparing to the others.

Building upon established evidence that acute heart failure significantly elevated the mortality and risks of severe disability, and that those with a 90-day mRS 5 diminished life quality and imposed a greater burden on healthcare resources, families, and society compared to patients with mRS 0–4, we designated the very poor outcome (mRS 5–6) as the primary outcome in the current study [40–42]. This outcome has also attracted increased concern in recent researches [27]. We found that PSHF could significantly increase the proportion of very poor outcome in LVO patients. The multifactorial pathophysiological mechanisms underlying PSHF fundamentally determined the poor outcomes in this cohort. PSHF could induce the hypoperfusion for cerebral tissues which were oxygen-glucose-sensitive, even if the large vessel recanalization has been achieved [43]. Moreover, emerging evidence highlighted that acute heart failure remarkably elevated the risks of multisystem complications, including pulmonary infection, renal impairment, electrolyte imbalance, which significantly deteriorated outcomes of LVO [44]. After the onset of acute heart failure, the systemic inflammatory cascade was activated through upregulated proinflammatory cytokines like Interleukin −6, which could exacerbate neurological deficits [45].

In the post hoc exploratory analysis restricted to the MARVEL trial, methylprednisolone showed a nominal association with improved outcomes among PSHF patients with moderate‑to‑severe cardioembolic LVO (nominal *p* = 0.027), but this did not remain significant after multiplicity correction (corrected *p* = 0.054). We caution against overinterpretation given the exploratory nature, limited sample size, multiple comparisons, and inadequate power to assess safety risks. Although corticosteroids may attenuate systemic inflammation, their use is associated with well‑documented risks, including infection, hyperglycemia, and gastrointestinal bleeding [46–48]. Moreover, corticosteroids may increase the risk of pneumonia through immunosuppression and exacerbate pulmonary edema through fluid retention [49]. The absence of significant benefits combined with these safety considerations does not support routine corticosteroid use outside clinical trials [50]. Dedicated randomized trials with prespecified PSHF endpoints and comprehensive safety monitoring are required for validation.

The DEVT and RESCUE-BT trials were designed to evaluate whether adjunctive alteplase or tirofiban could improve outcomes beyond standard EVT by enhancing recanalization [15,16]. However, our analyses showed that these trial-specific interventions had no significant impact on either PSHF incidence or functional outcomes in patients who developed PSHF. These results might suggest that recanalization-targeted strategies may not influence outcomes of PSHF patients among LVO patients, further supporting the notion that PSHF pathophysiology is more closely related to underlying cardiac vulnerability and stroke-induced systemic responses than to large-vessel recanalization status alone, as illustrated in Fig H in S1 Text [39]. In addition, sensitivity analyses accounting for study source and trial-specific interventions supported the consistency of our primary conclusions, confirming the validity of pooling data across these studies.

Our study was conducted entirely in Chinese stroke centers, and direct extrapolation of our findings to Western populations warrants careful consideration. Differences in healthcare systems, particularly periprocedural management strategies and intensive care practices may influence the detection of PSHF and the outcomes of patients with PSHF [51]. Nevertheless, several factors might enhance the applicability of our findings to broader settings. Cardioembolism, which accounted for 75.90% of PSHF cases in our cohort, is also one of the leading etiologies of ischemic stroke in Western countries [52]. The baseline NIHSS score in our study was comparable to that reported in Western randomized controlled trials for LVO patients [52]. The underlying pathophysiology of PSHF involving autonomic dysregulation and systemic inflammatory response is biologically shared across populations [53]. Our findings may support the value of cardiac monitoring in high-risk patients, which may warrant consideration across different healthcare settings. Validation in prospective multicenter studies involving diverse populations is warranted.

These results need to be interpreted within the limitations of the study. First, despite applying mixed-effects regression models with study-level random effects and center clustering, residual unmeasured confounding likely persists due to the observational design and pooling of studies with different protocols. While PSM balanced observed covariates, substantial baseline differences before matching suggested the presence of unmeasured confounders that these methods cannot fully address. Therefore, our findings require prospective validation. Second, transthoracic echocardiography was not systematically performed, as current stroke guidelines did not mandate it in the hyperacute phase of LVO due to potential delays in critical neurovascular interventions. Consequently, these quantitative parameters including left ventricular ejection fraction and *E*/*e*′ ratio were not captured [54]. Even when performed postoperatively, echocardiography cannot reliably distinguish pre-existing phenotypes, as cardiac function evolved dynamically after stroke through mechanisms such as neurogenic myocardial injury and stress-induced cardiomyopathy, rather than reflecting pre-existing heart failure with reduced ejection fraction or preserved ejection fraction. The absence of these echocardiographic data precluded effective evaluation of the relationship between cardiac function parameters and PSHF. This limitation underscores the value of incorporating pre-stroke and peri-procedural echocardiographic assessment in future investigations. Third, detailed peri-procedural management data including early diuretic use, vasodilator administration, and fluid management were not systematically recorded. Variation in timing, duration, intensity, and agent selection across centers precluded reliable adjustment. Exploration of their impacts would benefit from studies with standardized protocols. Fourth, BNP/NT-proBNP was measured based on clinical indication rather than universal screening across the four included studies. Although this reflected the real-world clinical practice, we acknowledge that mild or asymptomatic PSHF cases may have been undetected. Prospective studies with universal screening would help further clarify the incidence and impact of mild or subclinical PSHF. Fifth, we applied age-specific natriuretic peptide thresholds without renal function stratification. Creatinine was measured at admission, while natriuretic peptides were sampled at variable post-procedure time points [55]. Concurrent creatinine measurements were not systematically recorded. Serial renal function assessment and the use of renal function-stratified thresholds represent relevant directions for future research. Last, our findings are based on data from Chinese centers. The generalizability of these findings to Western healthcare systems is uncertain due to differences in racial demographics, comorbidity profiles, and medical practices, highlighting the need for validation in broader populations.

In conclusion, PSHF significantly worsens early and long-term outcomes in LVO patients undergoing EVT. Patients with moderate-to-severe cardioembolic LVO had a higher risk of developing PSHF. These findings suggest that routine preoperative PSHF risk assessment may be valuable in optimizing LVO patient management, though further prospective studies are needed to confirm these observations.

## Supporting information

S1 STROBE Checklist Strengthening the Reporting of Observational Studies in Epidemiology (STROBE) checklist (von Elm and colleagues, PLoS Medicine, 2007). The STROBE checklist is distributed under the Creative Commons Attribution License (CC BY 4.0): https://creativecommons.org/licenses/by/4.0/. Checklist is available at https://www.strobe-statement.org/.
(DOC)

S2 AGReMA Checklist Strengthening the reporting of mediation analysis by AGReMA (A Guideline for Reporting Mediation Analyses) guideline. Checklist downloaded from: https://agrema-statement.org.
(DOCX)

S1 Text Additional results and supplemental methods. **Fig A.** Flowchart of included patients. Of 3,500 patients initially meeting inclusion criteria, 3,415 (97.57%) were included in the final analysis. The 19 patients with missing baseline data were distributed across studies, with 7 from BASILAR registry (1.08% of 647), 4 from DEVT (1.71% of 234), 0 from RESCUE-BT (0.0% of 945), and 8 from MARVEL (0.48% of 1,674). Missing data rates were comparable between the registry and RCTs for both outcomes (Registry: 0/647 vs. RCTs: 7/2853; 0.00% vs. 0.25%; Fisher’s exact test, *P* = 0.362). **Fig B.** Temporal directed acyclic graph showing the sequence from baseline (T0) through post-procedure (T1) and acute phase (T2) to 90-day outcome (T3). Demographic characteristics (age, sex), medical history (atrial fibrillation, chronic heart failure, premorbid modified Rankin Scale), and disease features (occlusion site, baseline National Institutes of Health Stroke Scale, baseline Alberta Stroke Program Early CT Score, stroke etiology) at T0, together with onset to recanalization time and expanded Treatment in Cerebral Infarction score (eTICI) at T1, may influence PSHF development at T2 and subsequent poor functional outcome (modified Rankin Scale, mRS 5–6) at T3. **Fig C.** Simplified directed acyclic graph for mediation analysis. The exposure (moderate-to-severe stroke, defined as baseline National Institutes of Health Stroke Scale >16) may affect the outcome (very poor outcome, defined as modified Rankin Scale 5–6 at 90 days) directly (path c′) or indirectly through the mediator (PSHF). Path a represents the effect of exposure on mediator; Path b represents the effect of mediator on outcome, conditional on exposure. T0 include age, sex, atrial fibrillation, premorbid modified Rankin Scale (mRS), pre-existing chronic heart failure, baseline Alberta Stroke Program Early CT Score (ASPECTS), occlusion site, and stroke etiology, act as confounders in mediation analysis. **Fig D.** Propensity score matching assessment. Before matching, significant imbalances were observed across multiple covariates. After 1:1 propensity score nearest neighbor matching, all covariates achieved adequate balance with standardized mean differences (SMDs) <0.1, indicating successful elimination of baseline confounding between groups. **Fig E. Distribution of post-stroke acute heart failure cases stratified by cardioembolic etiology and stroke severity.** The post-stroke acute heart failure (PSHF) prevalence was highest among patients with moderate-to-severe cardioembolic large vessel occlusion accounting for 62.59% of all PSHF cases, nearly triple that of other subgroups. **Fig F. Rho-sensitivity analysis for unmeasured confounding in mediation analysis.** Rho (*ρ*) represents the correlation between unmeasured confounders and both the mediator and outcome. In the cardioembolism group, the observed mediation effect (7.70%) remained insensitive to unmeasured confounding of moderate strength (*ρ* < 0.80), with nullification requiring very strong confounding (*ρ* ≈ 0.90). In contrast, as an exploratory analysis to assess the specificity of the observed mediation effect in cardioembolic stroke patients, the non-cardioembolism group showed no meaningful mediation across all assumed confounding strengths. **Fig G. Association between cerebral hemorrhage and post-stroke acute heart failure.** Neither any cerebral hemorrhage (adjusted odds ratio (aOR) 1.34, 95% CI [0.94, 1.81]; *p* = 0.108) nor symptomatic cerebral hemorrhage (aOR 1.42, 95% CI [0.92, 2.21]; *p* = 0.114) was associated with the development of post-stroke acute heart failure. **Fig H in S1 Text** The “double-hit” hypothesis for the development of post-stroke acute heart failure (PSHF). PSHF arises from the synergistic interaction between pre-existing cardiac vulnerability and stroke-induced secondary cardiac injury including autonomic dysregulation, hypothalamic-pituitary-adrenal axis activation, and systemic inflammation. **Table A.** Comparison of baseline characteristics between included patients and those excluded due to missing baseline clinical or procedural characteristics. **Table B.** Comparison of baseline characteristics of patients among the four included studies. **Table C.** Baseline features of patients stratified by the presence of post-stroke acute heart failure based on propensity score matching dataset. **Table D.** Univariable and multivariable analysis for predictors of post-stroke acute heart failure based on mixed-effects models. **Table E.** Interaction models for National Institutes of Health Stroke Scale score and cardioembolism. **Table F.** Sensitivity analysis adjusting for time-varying confounding in medication analysis. **Table G.** Sensitivity analysis. Adjusted association between post-stroke acute heart failure and outcomes by fixed models. **Table H.** Sensitivity analysis. Adjusted association between post-stroke acute heart failure and outcomes by incorporating source studies as one of the adjustment covariates into the fixed models. **Table I.** Sensitivity analysis. Mixed-effects models inforparating study × post-stroke acute heart failure (PSHF) interaction for the association between PSHF and clinical outcomes. **Table J.** Sensitivity analysis. Pharmacological interventions in each trial did not substantially influence the development of post-stroke acute heart failure. **Table K.** Sensitivity analysis. The effects of trial interventions on 90-day functional outcome did not significantly differ between patients with and without post-stroke acute heart failure. **Table L.** The association between PSHF and Utility-weighted modified Rankin Scale (UW-mRS). **Table M.** The association between post-stroke acute heart failure and quality-of-life. **Table N.** Effects of adjunctive application of methylprednisolone on the incidence of post-stroke acute heart failure among patients in the MARVEL trial. **Table O.** The impact of methylprednisolone on the frequency of very poor outcomes among post-stroke acute heart failure patients in MARVEL trial. **Method A.** Key features distinguishing post-stroke acute heart failure from competing cardiopulmonary conditions. **Method B.** An overview of the variables and measurements in the study. **Method C.** Hierarchical adjustment strategies for sensitivity analysis of covariate selection. **Method D.** Additional outcome.
(DOCX)

S2 Text Planned analyses and changes. (DOCX)

S1 File Protocols for researches in this study. This compressed file contains the study protocols for the four multicenter studies included in this pooled analysis: BASILAR registry, DEVT trial, RESCUE-BT trial, and MARVEL trial. Each protocol document is provided in PDF format.
(ZIP)

## Abbreviations

aHR: adjusted hazard ratios
aOR: adjusted odds ratio
ASPECTS: Alberta Stroke Program Early CT Score
CI: confidence interval
cOR: common odds ratios
eTICI: expanded Thrombolysis in Cerebral Infarction
EVT: endovascular treatment
FDR: False Discovery Rate
HR: hazard ratios
IPTW: inverse probability of treatment weighting
LVO: large vessel occlusion stroke
mRS: modified Rankin Scale
NIHSS: National Institutes of Health Stroke Scale
PSHF: post-stroke acute heart failure
PSM: propensity score matching
STROBE: Strengthening the Reporting of Observational Studies in Epidemiology
