# Supplementary material for: Post-stroke acute heart failure in patients with large vessel occlusion undergoing endovascular treatment: A pooled analysis of individual patient data from multicenter studies with mediation analysis
Source: PLoS Med. 2026 Jul 7;23(7):e1004752. doi: 10.1371/journal.pmed.1004752 (PMC13340808; doi:10.1371/journal.pmed.1004752)
Supplement: S1 STROBE Checklist — The STROBE checklist is distributed under the Creative Commons Attribution License (CC BY 4.0): https://creativecommons.org/licenses/by/4.0/. Checklist is available at https://www.strobe-statement.org/. (DOC) [file pmed.1004752.s001.doc]

STROBE Statement—Checklist of items that should be included in reports of ***cohort studies***

|  | Item No | Recommendation | **Reported on** |
| --- | --- | --- | --- |
| **Title and abstract** | 1 | (*a*) Indicate the study’s design with a commonly used term in the title or the abstract | Abstract, Background, paragraph 1 |
|  |  | (*b*) Provide in the abstract an informative and balanced summary of what was done and what was found | Abstract, Conclusions, paragraph 1 |
| Introduction | | |  |
| Background/rationale | 2 | Explain the scientific background and rationale for the investigation being reported | Introduction, paragraph 1 |
| Objectives | 3 | State specific objectives, including any prespecified hypotheses | Introduction, paragraph 1 |
| Methods | | |  |
| Study design | 4 | Present key elements of study design early in the paper | Methods, Study design and patients, paragraph 1 |
| Setting | 5 | Describe the setting, locations, and relevant dates, including periods of recruitment, exposure, follow-up, and data collection | Methods, Methods, Study design and patients, paragraph 1-2 |
| Participants | 6 | (*a*) Give the eligibility criteria, and the sources and methods of selection of participants. Describe methods of follow-up | Methods, Methods, Study design and patients, paragraph 1-2 |
|  |  | (*b*) For matched studies, give matching criteria and number of exposed and unexposed | Methods, Study design and patients, paragraph 1-2 |
| Variables | 7 | Clearly define all outcomes, exposures, predictors, potential confounders, and effect modifiers. Give diagnostic criteria, if applicable | Methods, Definition of post-stroke acute heart failure and outcomes, paragraph 1 |
| Data sources/ measurement | 8* | For each variable of interest, give sources of data and details of methods of assessment (measurement). Describe comparability of assessment methods if there is more than one group | Methods, Definition of post-stroke acute heart failure and outcomes, paragraph 2-3 |
| Bias | 9 | Describe any efforts to address potential sources of bias | Methods, Definition of post-stroke acute heart failure and outcomes, paragraph 4 |
| Study size | 10 | Explain how the study size was arrived at | Methods, Study design and patients, paragraph 2 |
| Quantitative variables | 11 | Explain how quantitative variables were handled in the analyses. If applicable, describe which groupings were chosen and why | Methods, Definition of post-stroke acute heart failure and outcomes, paragraph 1-2 |
| Statistical methods | 12 | (*a*) Describe all statistical methods, including those used to control for confounding | Methods, Statistical analysis, paragraph 1 |
|  |  | (*b*) Describe any methods used to examine subgroups and interactions | Methods, Statistical analysis, paragraph 4 and 7 |
|  |  | (*c*) Explain how missing data were addressed | Methods, Statistical analysis, paragraph 2 and 3 |
|  |  | (*d*) If applicable, explain how loss to follow-up was addressed | Methods, Statistical analysis, paragraph 2 and 3 |
|  |  | (*e*) Describe any sensitivity analyses | Methods, Statistical analysis, paragraph 7 |
| Results | | |  |
| Participants | 13* | (a) Report numbers of individuals at each stage of study—eg numbers potentially eligible, examined for eligibility, confirmed eligible, included in the study, completing follow-up, and analysed | Methods, Study design and patients, paragraph 2 |
|  |  | (b) Give reasons for non-participation at each stage | Methods, Study design and patients, paragraph 2 |
|  |  | (c) Consider use of a flow diagram | Methods, Study design and patients, paragraph 2 |
| Descriptive data | 14* | (a) Give characteristics of study participants (eg demographic, clinical, social) and information on exposures and potential confounders | Results, Patient characteristics, paragraph 1 |
|  |  | (b) Indicate number of participants with missing data for each variable of interest | Results, Patient characteristics, paragraph 1 |
|  |  | (c) Summarise follow-up time (eg, average and total amount) | Results, Patient characteristics, paragraph 1-2 |
| Outcome data | 15* | Report numbers of outcome events or summary measures over time | Results, Clinical outcomes according to the presence of PSHF, paragraph 1 |
| Main results | 16 | (*a*) Give unadjusted estimates and, if applicable, confounder-adjusted estimates and their precision (eg, 95% confidence interval). Make clear which confounders were adjusted for and why they were included | Results, Clinical outcomes according to the presence of PSHF, paragraph 1 |
|  |  | (*b*) Report category boundaries when continuous variables were categorized | Results, Clinical outcomes according to the presence of PSHF, paragraph 1 |
|  |  | (*c*) If relevant, consider translating estimates of relative risk into absolute risk for a meaningful time period | Results, Predictors for the presence of PSHF, paragraph 1 |
| Other analyses | 17 | Report other analyses done—eg analyses of subgroups and interactions, and sensitivity analyses | Results, Interaction between NIHSS and cardioembolism in association with PSHF, paragraph 1-2 ; Subgroup and sensitivity analysis, paragraph 1-3 |
| Discussion | | |  |
| Key results | 18 | Summarise key results with reference to study objectives | Discussion, paragraph 1 |
| Limitations | 19 | Discuss limitations of the study, taking into account sources of potential bias or imprecision. Discuss both direction and magnitude of any potential bias | Discussion, Limitations,  paragraph 1 |
| Interpretation | 20 | Give a cautious overall interpretation of results considering objectives, limitations, multiplicity of analyses, results from similar studies, and other relevant evidence | Discussion, Conclusion, paragraph 1 |
| Generalisability | 21 | Discuss the generalisability (external validity) of the study results | Discussion, Conclusion, paragraph 1 |
| Other information | | |  |
| Funding | 22 | Give the source of funding and the role of the funders for the present study and, if applicable, for the original study on which the present article is based | Other information, Funding, paragraph 1 |

*Give information separately for exposed and unexposed groups.

**Note:** An Explanation and Elaboration article discusses each checklist item and gives methodological background and published examples of transparent reporting. The STROBE checklist is best used in conjunction with this article (freely available on the Web sites of PLoS Medicine at http://www.plosmedicine.org/, Annals of Internal Medicine at http://www.annals.org/, and Epidemiology at http://www.epidem.com/). Information on the STROBE Initiative is available at http://www.strobe-statement.org.
