# Supplementary material for: Post-stroke acute heart failure in patients with large vessel occlusion undergoing endovascular treatment: A pooled analysis of individual patient data from multicenter studies with mediation analysis
Source: PLoS Med. 2026 Jul 7;23(7):e1004752. doi: 10.1371/journal.pmed.1004752 (PMC13340808; doi:10.1371/journal.pmed.1004752)
Supplement: S2 AGReMA Checklist — Checklist downloaded from: https://agrema-statement.org. (DOCX) [file pmed.1004752.s002.docx]

**S1 Text. Additional results and supplemental methods**

**Fig A in S1 Text Flowchart of included patients.** Of 3,500 patients initially meeting inclusion criteria, 3415 (97.57%) were included in the final analysis. The 19 patients with missing baseline data for clinical and procedural characteristics were distributed across studies, with 7 from BASILAR registry (1.08% of 647), 4 from DEVT (1.71% of 234), 0 from RESCUE-BT (0.00% of 945), and 8 from MARVEL (0.48% of 1,674). Missing data rates for 90-day follow-up were comparable between the registry and RCTs for both outcomes (Registry: 0/647 vs. RCTs: 7/2853; 0.00% vs 0.25%; Fisher’s exact test, P=0.362).

**Abbreviations:** BASILAR, Endovascular treatment for acute basilar artery occlusion study; DEVT, Direct endovascular thrombectomy versus combined intravenous thrombolysis and endovascular thrombectomy for patients with acute large vessel occlusion in the anterior circulation trial; LVO, large vessel occlusion; MARVEL, Methylprednisolone as adjunctive to endovascular treatment for acute large vessel occlusion; RCTs, randomized controlled trials; RESCUE-BT, Endovascular treatment with vs without tirofiban for patients with LVO stroke.


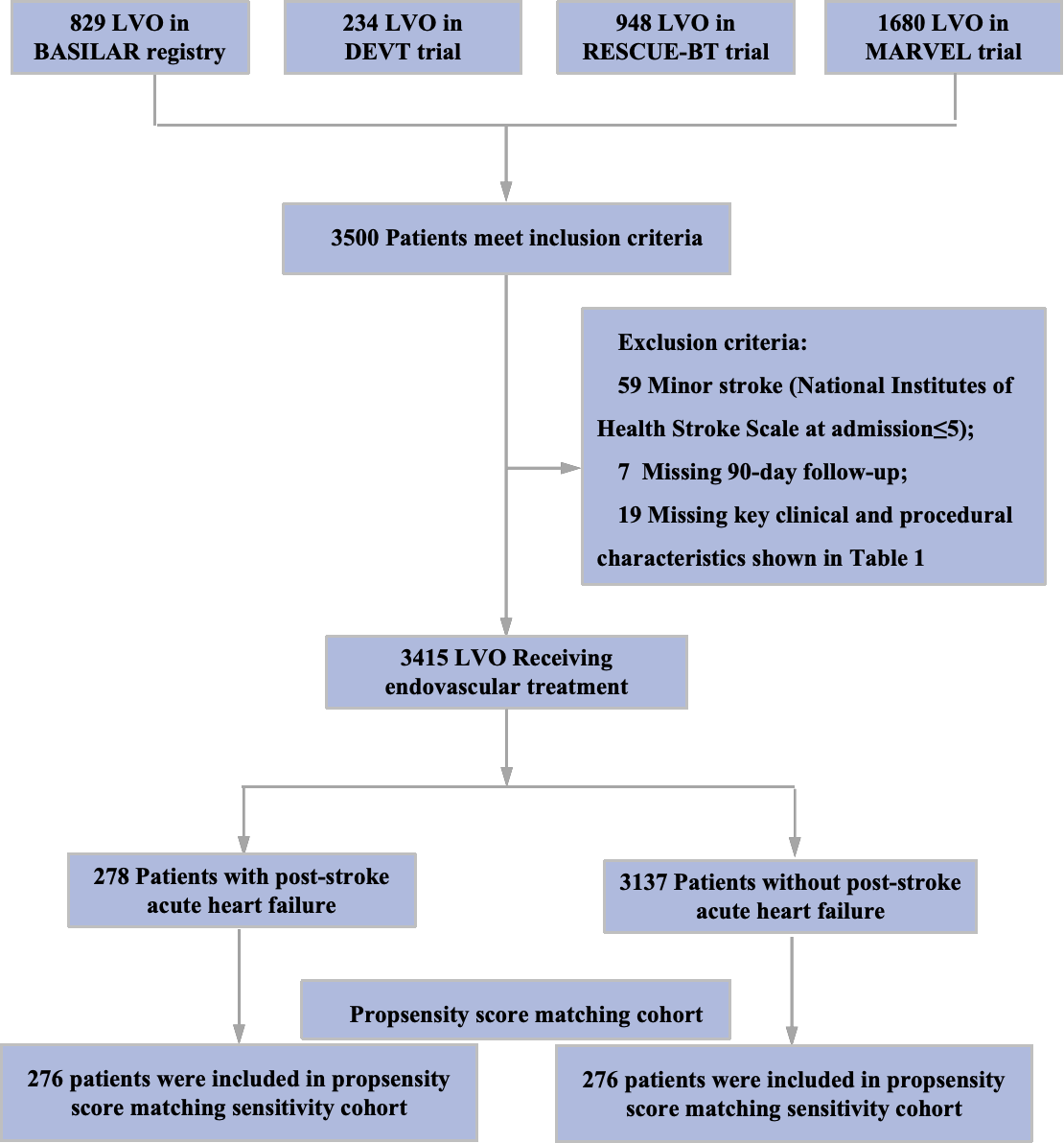


**Fig B in S1 Text Temporal directed acyclic graph of the sequence from baseline (T0) through post-procedure (T1) and early postoperative period (T2) to 90-day outcome (T3).** Demographic characteristics (age, sex), medical history (atrial fibrillation, pre-existing chronic heart failure, premorbid modified Rankin Scale), and disease features (occlusion site, baseline National Institutes of Health Stroke Scale, baseline Alberta Stroke Program Early CT Score, stroke etiology) at T0, together with onset to recanalization time and expanded Treatment in Cerebral Infarction score at T1, may influence post-stroke acute heart failure (PSHF) development at T2 and subsequent poor functional outcome (modified Rankin Scale score, mRS 5-6) at T3.

**Abbreviations:** ASPECTS, Alberta Stroke Program Early CT Score; DAG, directed acyclic graph; eTICI, expanded Treatment in Cerebral Infarction; mRS, modified Rankin Scale; NIHSS, National Institutes of Health Stroke Scale; PSHF, post-stroke acute heart failure; T0, baseline; T1, post-procedure; T2, 0-7 days after stroke onset; T3, 90 days.


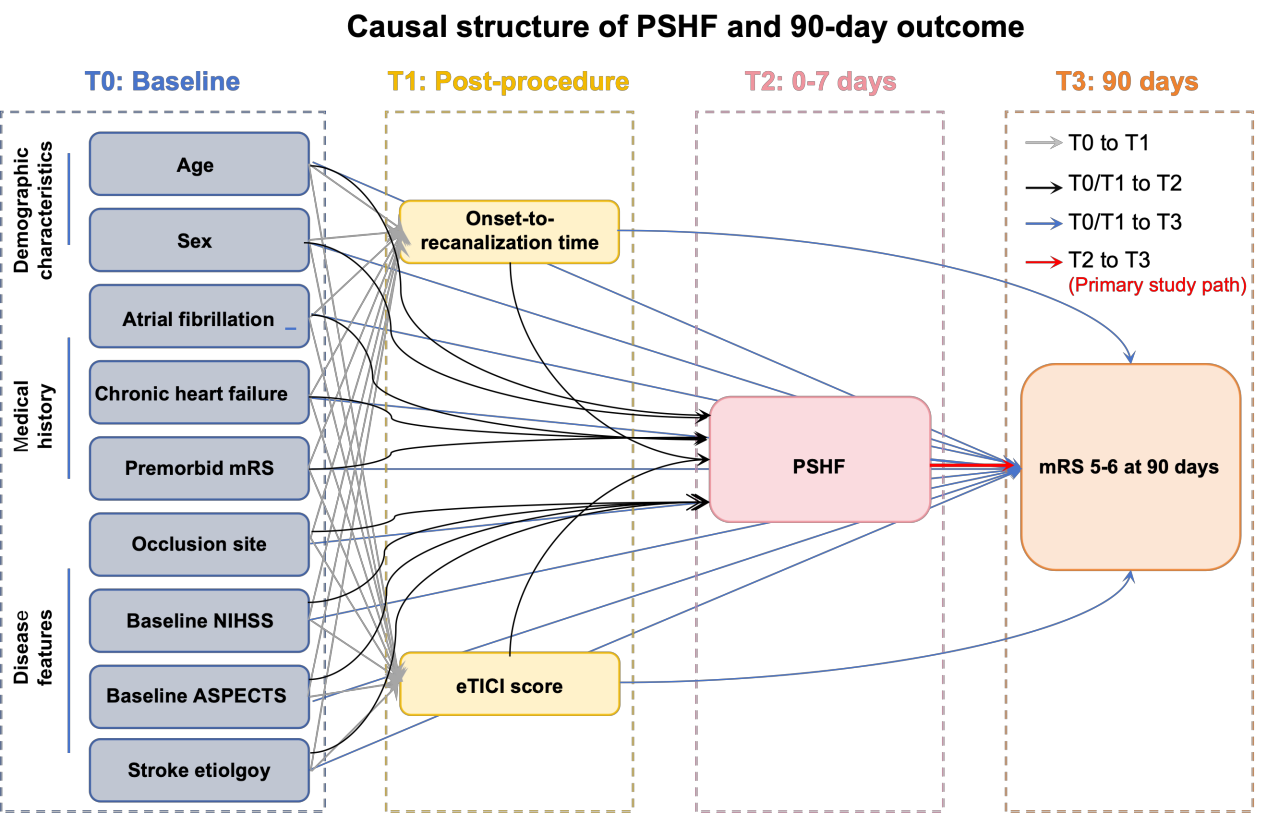


**Fig C in S1 Text Simplified directed acyclic graph for mediation analysis.** The exposure (moderate-to-severe stroke, defined as baseline National Institutes of Health Stroke Scale >16) may affect the outcome (very poor outcome, defined as modified Rankin Scale 5-6 at 90 days) directly (path c’) or indirectly through the mediator (PSHF). Path a represents the effect of exposure on mediator; Path b represents the effect of mediator on outcome, conditional on exposure. T0 variables (age, sex, atrial fibrillation, premorbid mRS, pre-existing chronic heart failure, baseline Alberta Stroke Program Early CT Score, and occlusion site) are included as confounders in the mediation analysis.

**Abbreviations:** ASPECTS, Alberta Stroke Program Early CT Score; mRS, modified Rankin Scale; NIHSS, National Institutes of Health Stroke Scale; PSHF, post-stroke acute heart failure.


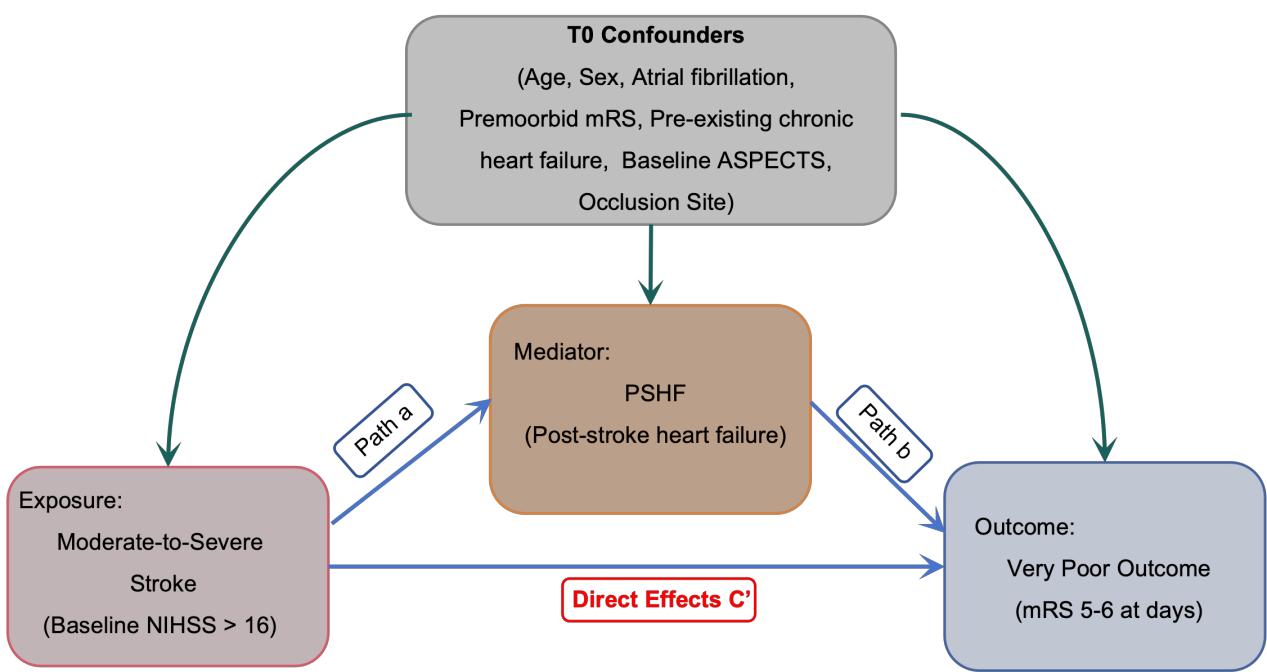


**Fig D in S1 Text Propensity score matching matching assessment.** Before matching, significant imbalances were observed across multiple covariates. After 1:1 propensity score nearest neighbor matching, all covariates achieved adequate balance with standardized mean differences <0.1, indicating successful elimination of baseline confounding between groups.

**Abbreviations:** ASPECTS, Alberta Stroke Program Early CT Score; CE, cardioembolism; eTICI, expanded Treatment in Cerebral Infarction; ICA, internal carotid artery; mRS, modified Rankin Scale; M1, M1 segment of the middle cerebral artery; M2, M2 segment of the middle cerebral artery; NIHSS, National Institutes of Health Stroke Scale; PS, propensity score; PSM, propensity score matching; SMD, standardized mean difference; TOAST, Trial of Org 10172 in Acute Stroke Treatment.


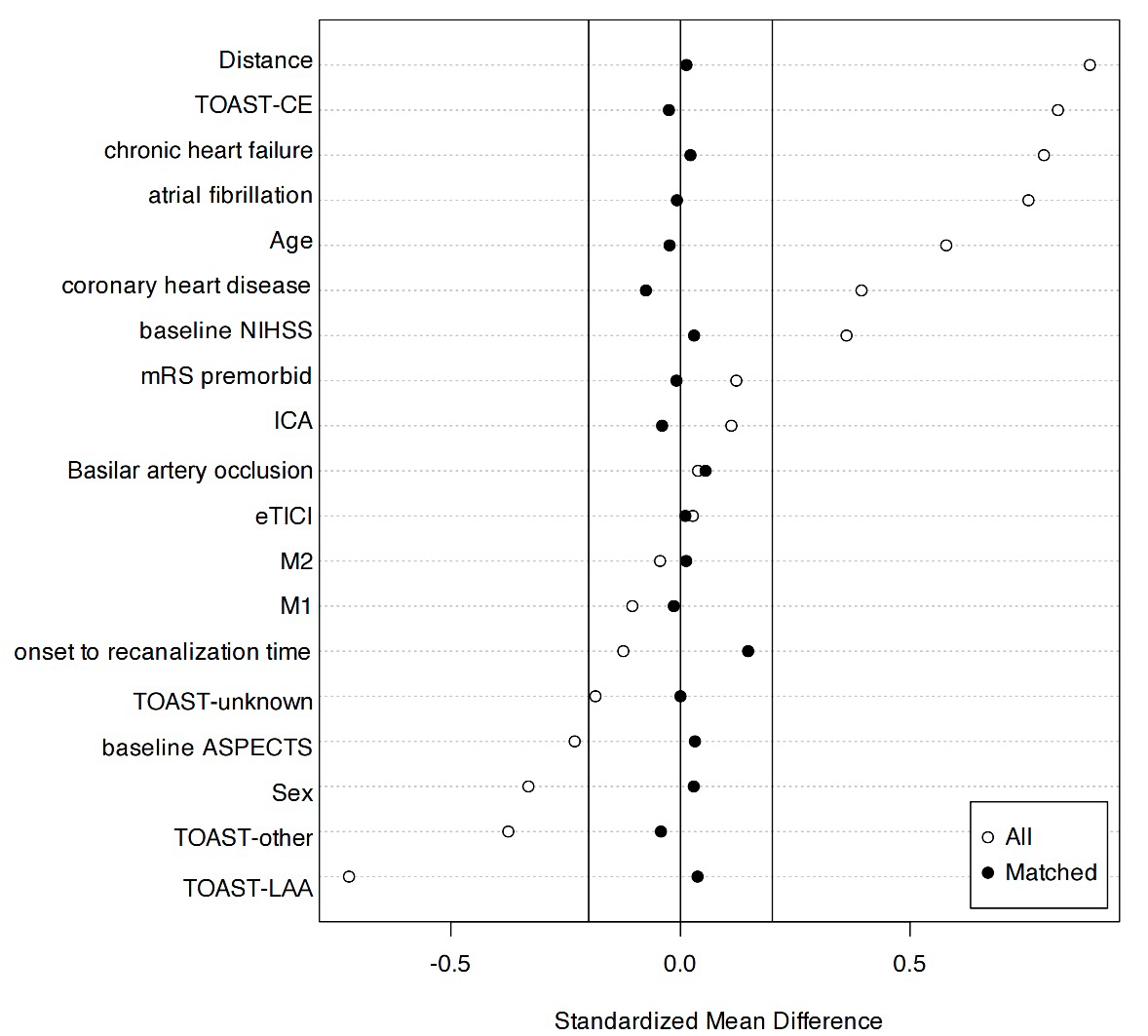


**Fig E in S1 Text Distribution of post-stroke acute heart failure cases stratified by cardioembolic etiology and stroke severity.** The post-stroke acute heart failure (PSHF) prevalence was highest among patients with moderate-to-severe cardioembolic large vessel occlusion accounting for 62.59% of all PSHF cases, nearly triple that of other subgroups.

**Abbreviations:** NIHSS, National Institutes of Health Stroke Scale; PSHF, post-stroke acute heart failure.

**
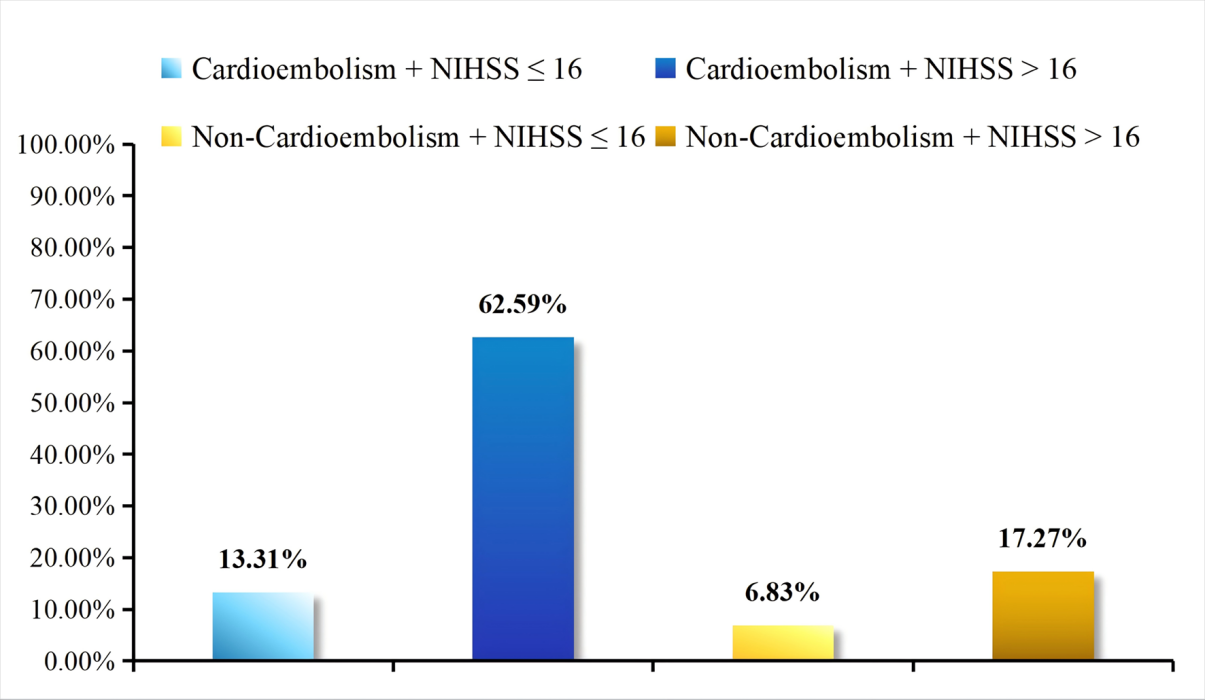
**

**Fig F in S1 Text.** **Rho-sensitivity analysis for unmeasured confounding in mediation analysis.** Rho (ρ) represents the correlation between unmeasured confounders and both the mediator and outcome. In the cardioembolism group, the observed mediation effect (7.70%) remained insensitive to unmeasured confounding of moderate strength (ρ < 0.80), with nullification requiring very strong confounding (ρ ≈ 0.90). In contrast, as an exploratory analysis to assess the specificity of the observed mediation effect in cardioembolic stroke patients, the non-cardioembolism group showed no meaningful mediation across all assumed confounding strengths.

**
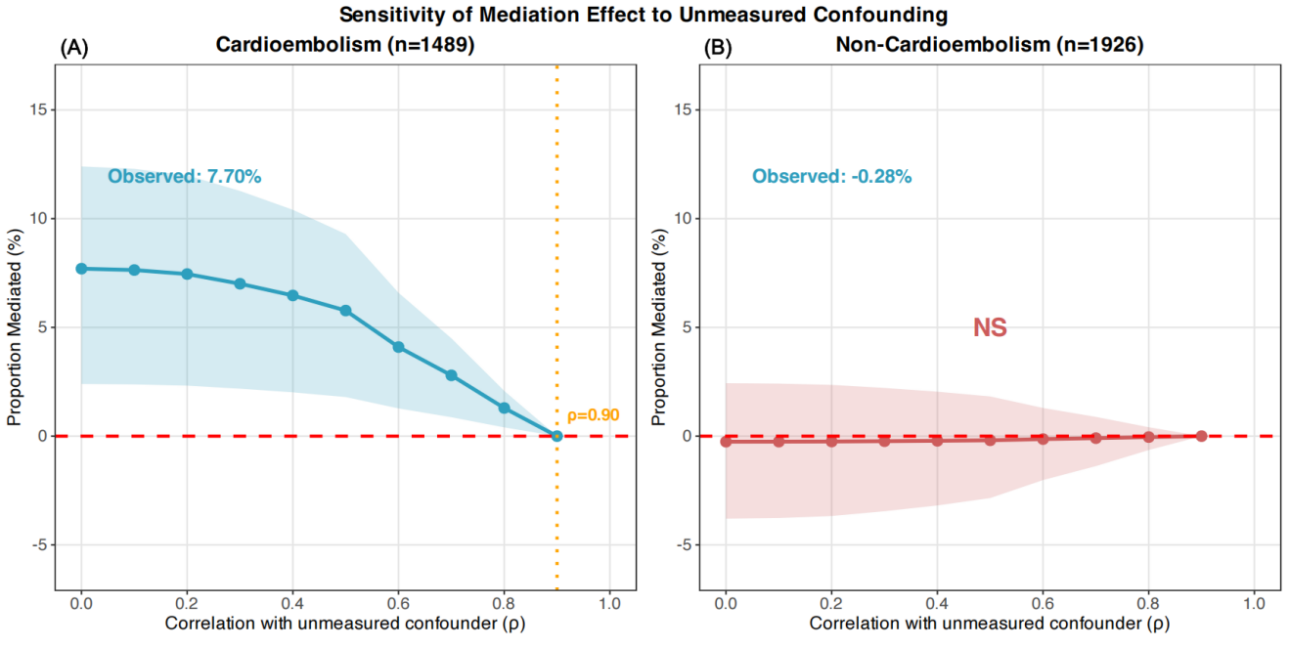
**

**Fig G in S1 Text Association between cerebral hemorrhage and post-stroke acute heart failure.** Neither any cerebral hemorrhage (adjusted odds ratio (aOR) 1.31, 95% confidence interval (CI) [0.94, 1.81]; *p*=0.108) nor symptomatic cerebral hemorrhage (aOR 1.42, 95% CI [0.92, 2.21]; *p*=0.114) was associated with the development of post-stroke acute heart failure.


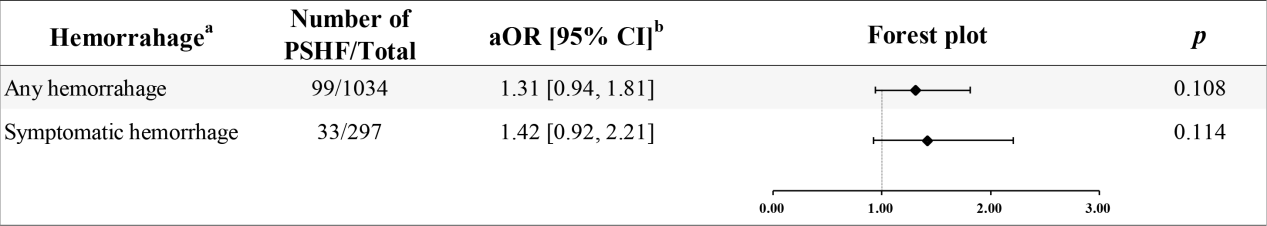


^a^ 31 patients without follow-up CT were excluded.

^b^ Mixed-effects models were adjusted for age, sex, history of atrial fibrillation, pre-existing chronic heart failure, premorbid mRS score, occlusion site, baseline Alberta Stroke Program Early CT Score, baseline National Institutes of Health Stroke Scale, stroke etiology (Trial of Org 10172 in Acute Stroke Treatment), expanded Thrombolysis In Cerebral Infarction, and onset to recanalization time. The *p* values were obtained from adjusted mixed-effects logistic regression models.

**Abbreviations:** aOR, adjusted odds ratio; CI, confidence interval; PSHF, post-stroke acute heart failure.

**Fig H in S1 Text The “double-hit” hypothesis for the development of post-stroke acute heart failure.** The post-stroke acute heart failure might arise from the synergistic interaction between pre-existing cardiac vulnerability and stroke-induced secondary cardiac injury including autonomic dysregulation, hypothalamic-pituitary-adrenal axis activation, and systemic inflammation. This figure was partially created in BioRender. Chen, L. (2026) https://BioRender.com/rxvw4pp.

**
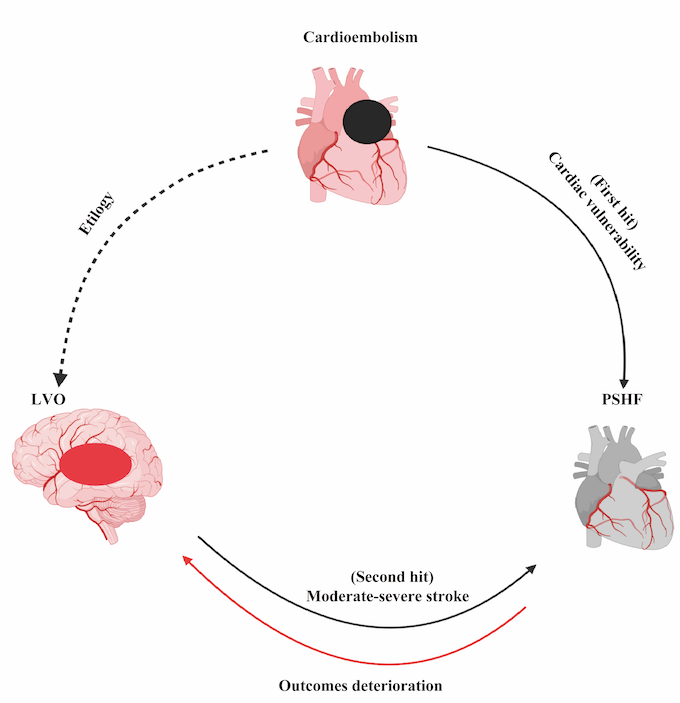
**

**Abbreviations:** PSHF, post-stroke acute heart failure. LVO, large vessel occlusion.

**Table A in S1 Text Comparison of baseline characteristics between included patients and those excluded due to missing baseline clinical or procedural characteristics.**

|  | **Excluded Patients**  **(N=19)** | **Included patients**  **(N=3415)** | ***p*** |
| --- | --- | --- | --- |
| Age, median [IQR], year | 67.00 [57.00, 72.00] | 68.00 [58.00, 75.00] | 0.395 |
| Sex, n (%) |  |  | 0.811 |
| Male | 12 (63.16) | 2065 (60.47) |  |
| Female | 7 (36.84) | 1350 (39.53) |  |
| Baseline NIHSS score, median [IQR] | 19.00 [15.50, 23.50] | 18.00 [15.00, 22.00] | 0.319 |
| Medical history, n (%) |  |  |  |
| Hypertension | 12 (63.16) | 2094 (61.32) | 0.870 |
| Diabetes mellitus | 5 (26.32) | 697 (20.41) | 0.524 |
| Atrial fibrillation | 10 (52.63) | 1244 (36.43) | 0.143 |
| Previous ischemic stroke | 2 (10.53) | 572 (16.75) | 0.468 |
| Prestroke modified Rankin scale, n (%) |  |  | 0.524 |
| 0 | 17 (89.47) | 3168 (92.77) |  |
| 1 | 2 (10.53) | 184 (5.39) |  |
| 2 | 0 (0.00) | 63 (18.4) |  |
| Occlusion site, n (%) |  |  | 0.069 |
| Internal carotid artery | 5 (26.32) | 802 (23.48) |  |
| M1 segment | 4 (21.05) | 1637 (47.94) |  |
| M2 segment | 3 (15.79) | 360 (10.54) |  |
| Basilar artery | 7 (36.84) | 616 (18.04) |  |
| Baseline ASPECTS^a^, median [IQR] | 8.00 [7.00, 9.00] | 7.00 [5.00, 8.00] | 0.067 |
| Stroke etiology, n (%) |  |  | 0.560 |
| Large artery atherosclerosis | 7 (36.84) | 1503 (44.01) |  |
| Cardioembolism | 11 (57.89) | 1489 (43.60) |  |
| Other | 0 (0.00) | 124 (3.63) |  |
| Unknown | 1 (5.26) | 299 (8.76) |  |
| Intravenous thrombolysis | 4 (21.05) | 870 (25.48) | 0.659 |
| eTICI^b^, n (%) |  |  | 0.245 |
| 0 | 2 (14.29) | 199 (5.83) |  |
| 1 | 0 (0.00) | 25 (0.73) |  |
| 2a | 2 (14.29) | 172 (5.04) |  |
| 2b | 4 (28.57) | 725 (21.23) |  |
| 2c | 0 (0.00) | 456 (13.35) |  |
| 3 | 6 (42.86) | 1838 (53.82) |  |
| Workflow times, median [IQR], min |  |  |  |
| Onset to puncture time | 262.00 [197.50, 383.00] | 339.00 [225.00, 565.00] | 0.086 |
| Onset to recanalization time^c^ | 407.00 [318.00, 449.50] | 430.00 [306.00, 663.00] | 0.331 |

Data are presented as median [interquartile range (IQR)] for continuous variables and n (%) for categorical variables. The *p* values were calculated using the χ² test or Fisher's exact test for categorical variables and the Mann-Whitney U test for continuous variables.

**Abbreviations:** ASPECTS, Alberta Stroke Program Early CT Score; eTICI, expanded treatment in cerebral infarction; IQR, interquartile range; M1, M1 segment of the middle cerebral artery; M2, M2 segment of the middle cerebral artery; NIHSS, National Institutes of Health Stroke Scale; PSHF, post-stroke acute heart failure.

^a^10 patients missing data of baseline ASPECTS

^b^5 patients missing data of eTICI

^c^4 patients missing data of onset to recanalization time

**Table B in S1 Text Comparison of baseline characteristics of patients among the four included studies.**

|  | **BASILAR**  **(N=616)** | **RESCUE-BT**  **(N=917)** | | **DEVT**  **(N=223)** | **MARVEL**  **(N=1659)** | ***p* value** |
| --- | --- | --- | --- | --- | --- | --- |
| **Study Overview** |  |  | |  |  |  |
| Study type | Prospective Registry | Trial | | Trial | Trial | — |
| Time Period | January 2014 to  May 2019 | October 2018 to October 2021 | | May 2018 to  May 2020 | February 2022 to June 2023 | — |
| Aim | To evaluate the association between EVT and clinical outcomes of patients with  acute posterior large vessel occlusion | To assess the efficacy and adverse events of intravenous tirofiban before  EVT for acute ischemic stroke secondary to anterior large  vessel occlusion | | To investigate whether EVT alone is noninferior to intravenous alteplase followed by EVT for achieving functional independence at 90 days among patients with anterior large vessel occlusion stroke. | To assess the efficacy and adverse events of adjunctive intravenous low-dose  methylprednisolone to EVT for acute ischemic stroke secondary  to anterior large  vessel occlusion | — |
| Intervention | — | Intravenous tirofiban | | Intravenous alteplase | Intravenous low-dose  methylprednisolone | — |
| Country | China  (47 medical centers) | China  (55 medical centers) | | China  (33 medical centers) | China  (82 medical centers) | — |
| Age, median [IQR], year | 64.00 [57.00, 73.00] | 68.00 [57.00, 75.00] | | 70.00 [61.00, 78.00] | 69.00 [59.00, 76.00] | <0.001 |
| Sex, n (%) |  |  | |  |  | <0.001 |
| Male | 461 (74.84) | 537 (58.56) | | 125 (56.05) | 942 (56.78) |  |
| Female | 155 (25.16) | 380 (41.44) | | 98 (43.95) | 717 (43.22) |  |
| Baseline NIHSS score, median [IQR] | 27.00 [18.00, 34.00] | 16.00 [12.00, 19.00] | | 16.00 [13.00, 20.00] | 19.00 [16.00, 21.00] | <0.001 |
| Medical history, n (%) |  |  | |  |  |  |
| Hypertension | 435 (70.62) | 509 (55.51) | | 135 (60.54) | 1015 (61.18) | <0.001 |
| Diabetes mellitus | 142 (23.05) | 195 (21.26) | | 41 (18.39) | 319 (19.23) | 0.168 |
| Pre-existing chronic heart failure | 41 (6.66) | 46 (5.02) | | 16 (7.17) | 186 (11.21) | <0.001 |
| Atrial fibrillation | 129 (20.94) | 309 (33.70) | | 120 (53.81) | 686 (41.35) | <0.001 |
| Previous ischemic stroke | 136 (22.8) | 155 (16.90) | | 32 (14.35) | 249 (15.01) | 0.001 |
| Prestroke modified Rankin scale, n (%) |  |  | |  |  | <0.001 |
| 0 | 519 (84.25) | 839 (91.49) | | 207 (92.83) | 1603 (96.62) |  |
| 1 | 68 (11.04) | 58 (6.32) | | 16 (7.17) | 42 (2.53) |  |
| 2 | 29 (4.71) | 20 (2.18) | | 0 (0.00) | 14 (0.84) |  |
| Occlusion site, n (%) |  |  | |  |  | <0.001 |
| Internal carotid artery | 0 (0.00) | 190 (20.72) | | 40 (17.94) | 572 (34.48) |  |
| M1 segment | 0 (0.00) | 594 (64.78) | | 174 (78.03) | 869 (52.38) |  |
| M2 segment | 0 (0.00) | 133 (14.50) | | 9 (4.04) | 218 (13.14) |  |
| Basilar artery | 616 (100.0) | 0 (0.00) | | 0 (0.00) | 0 (0.00) |  |
| Baseline ASPECTS, median [IQR] | 8.00 [7.00, 9.00] | 8.00 [7.00, 9.00] | | 8.00 [7.00, 9.00] | 6.00 [4.00, 7.00] | <0.001 |
| Stroke etiology, n (%) |  |  | |  |  | <0.001 |
| Large artery atherosclerosis | 398 (64.61) | 416 (45.37) | | 55 (24.66) | 634 (38.22) |  |
| Cardioembolism | 163 (26.46) | 396 (43.18) | | 130 (58.30) | 800 (48.22) |  |
| Other | 19 (3.08) | 29 (3.16) | | 4 (1.79) | 72 (4.34) |  |
| Unknown | 36 (5.84) | 76 (8.29) | | 34 (15.25) | 153 (9.22) |  |
| eTICI, n (%) |  |  | |  |  | <0.001 |
| 0 | 55 (8.93) | 39 (4.25) | | 4 (1.79) | 101 (6.09) |  |
| 1 | 14 (2.27) | 3 (0.33) | | 2 (0.90) | 6 (0.36) |  |
| 2a | 52 (8.44) | 36 (3.93) | | 22 (9.87) | 62 (3.74) |  |
| 2b | 122 (19.81) | 228 (24.86) | | 64 (28.70) | 311 (18.75) |  |
| 2c | 106 (17.21) | 135 (14.72) | | 40 (17.94) | 175 (10.55) |  |
| 3 | 267 (43.34) | 476 (51.91) | | 91 (40.81) | 1004 (60.52) |  |
| Workflow times, median [IQR], min |  |  |  | |  | |
| Onset to puncture time | 323.50  [219.00, 488.50] | 395.00  [255.00, 616.00] | | 204.00  [170.00, 250.00] | 350.00  [235.00, 601.00] | <0.001 |
| Onset to recanalization time | 436.00  [326.00,621.20] | 481.00 [325.00,710.00] | | 289.00 [238.50, 335.00] | 436.00  [311.50, 691.00] | <0.001 |

Data are presented as median [interquartile range (IQR)] for continuous variables and n (%) for categorical variables. The *p* values for overall comparison were calculated using the Kruskal-Wallis test for continuous variables and the χ² test for categorical variables, as appropriate.

**Abbreviations:** ASPECTS, Alberta Stroke Program Early CT Score; eTICI, expanded treatment in cerebral infarction; IQR, interquartile range; M1, M1 segment of the middle cerebral artery; M2, M2 segment of the middle cerebral artery; NIHSS, National Institutes of Health Stroke Scale; PSHF, post-stroke acute heart failure.

**Table C in S1 Text Baseline features of patients stratified by the presence of post-stroke acute heart failure based on propensity score matching dataset.**

|  | **No-PSHF**  **(N=276)** | **PSHF**  **(N=276)** | ***p*** |
| --- | --- | --- | --- |
| Age, median [IQR], year | 74.00 [68.00, 80.00] | 74.00 [66.00, 79.00] | 0.328 |
| Sex, n (%) |  |  | 0.268 |
| Male | 138 (50.00) | 125 (45.29) |  |
| Female | 138 (50.00) | 151 (54.71) |  |
| Baseline NIHSS score, median [IQR] | 20.00 [17.00, 24.00] | 20.00 [17.00, 23.00] | 0.941 |
| Medical history, n (%) |  |  |  |
| Hypertension | 176 (63.77) | 173 (62.68) | 0.923 |
| Diabetes mellitus | 59 (21.38) | 60 (21.74) | 0.918 |
| Pre-existing chronic heart failure | 117 (42.39) | 122 (44.20) | 0.668 |
| Atrial fibrillation | 183 (66.30) | 189 (68.48) | 0.586 |
| Previous ischemic stroke | 54 (19.57) | 46 (16.67) | 0.377 |
| Prestroke modified Rankin scale, n (%) |  |  | 0.491 |
| 0 | 242 (87.68) | 245 (88.77) |  |
| 1 | 22 (7.97) | 24 (8.70) |  |
| 2 | 12 (4.35) | 7 (2.54) |  |
| Occlusion site, n (%) |  |  | 0.719 |
| Internal carotid artery | 72 (26.09) | 78 (28.26) |  |
| M1 segment | 114 (41.30) | 120 (43.48) |  |
| M2 segment | 32 (11.59) | 26 (9.42) |  |
| Basilar artery | 58 (21.01) | 52 (18.84) |  |
| Baseline ASPECTS, median [IQR] | 7.00 [4.00, 8.00] | 6.00 [4.00, 8.00] | 0.446 |
| Stroke etiology, n (%) |  |  | 0.953 |
| Large artery atherosclerosis | 51 (18.48) | 51 (18.48) |  |
| Cardioembolism | 210 (76.09) | 209 (75.72) |  |
| Other | 1 (0.36) | 2 (0.72) |  |
| Unknown | 14 (5.07) | 14 (5.07) |  |
| Intravenous thrombolysis | 75 (27.17) | 69 (25.00) | 0.561 |
| eTICI, n (%) |  |  | 0.390 |
| 0 | 19 (6.88) | 14 (5.07) |  |
| 1 | 3 (1.09) | 0 (0.00) |  |
| 2a | 12 (4.35) | 17 (6.16) |  |
| 2b | 56 (20.29) | 63 (22.83) |  |
| 2c | 26 (9.42) | 28 (10.14) |  |
| 3 | 160 (57.97) | 154 (55.80) |  |
| Workflow times, median [IQR], min |  |  |  |
| Onset to puncture time | 298.00 [211.50, 480.75] | 306.00 [210.00, 518.50] | 0.355 |
| Onset to recanalization time | 374.00 [274.50, 574.25] | 388.50 [293.50, 585.50] | 0.306 |

Data are presented as median [interquartile range (IQR)] for continuous variables and n (%) for categorical variables. The *p* values were calculated using theχ² test or Fisher's exact test for categorical variables and the Mann-Whitney U test for continuous variables.

**Abbreviations**: ASPECTS, Alberta Stroke Program Early CT Score; eTICI, expanded treatment in cerebral infarction; IQR, interquartile range; M1, M1 segment of the middle cerebral artery; M2, M2 segment of the middle cerebral artery; NIHSS, National Institutes of Health Stroke Scale; PSHF, post-stroke acute heart failure.

**Table D in S1 Text Univariable and multivariable analysis for predictors of post-stroke acute heart failure based on mixed-effects models.**

| **Variables** | **Post-stroke heart failure** | | | |
| --- | --- | --- | --- | --- |
|  | Univariable analysis | | Multivariable analysis | |
|  | OR [95% CI] | *p* | Adjusted OR [95% CI] | *p* |
| Age | 1.05 [1.04, 1.06] | <0.001 | 1.03 [1.02, 1.05] | <0.001 |
| Sex | 0.51 [0.40, 0.65] | <0.001 | 0.92 [0.69, 1.24] | 0.598 |
| Baseline NIHSS score | 1.07 [1.04, 1.09] | <0.001 | 1.04 [1.01, 1.07] | 0.003 |
| Medical history |  |  |  |  |
| Pre-existing chronic heart failure | 15.73 [11.52, 21.48] | <0.001 | 10.40 [7.50, 14.42] | <0.001 |
| Atrial fibrillation | 4.51 [3.44, 5.91] | <0.001 | 1.58 [1.09, 2.30] | 0.017 |
| Prestroke modified Rankin scale | 1.52 [1.12, 2.06] | 0.007 | 1.13 [0.80, 1.60] | 0.491 |
| Occlusion site |  |  |  |  |
| Internal carotid artery | Reference | Reference | Reference | Reference |
| M1 segment | 0.79 [0.59, 1.07] | 0.135 | 0.84 [0.60, 1.19] | 0.331 |
| M2 segment | 0.77 [0.48, 1.22] | 0.259 | 0.66 [0.39, 1.10] | 0.113 |
| Basilar artery | 0.94 [0.46, 1.91] | 0.868 | 1.17 [0.66, 2.06] | 0.596 |
| Baseline ASPECTS | 0.90 [0.85, 0.95] | <0.01 | 0.91[0.85, 0.98] | 0.008 |
| Stroke etiology |  |  |  |  |
| Large artery atherosclerosis | Reference | Reference | Reference | Reference |
| Cardioembolism | 4.97 [3.61, 6.86] | <0.001 | 2.10 [1.37, 3.21] | <0.001 |
| Other | 0.46 [0.11, 1.88] | 0.282 | 0.56 [0.13, 2.45] | 0.445 |
| Unknown | 1.45 [0.8, 2.66] | 0.223 | 1.21 [0.64, 2.32] | 0.556 |
| eTICI | 1.01[0.93, 1.11] | 0.787 | 1.02 [0.92, 1.12] | 0.750 |
| Onset to recanalization time | 1.00 [1.00, 1.00] | 0.062 | 1.00 [1.00, 1.00] | 0.998 |

The *p* values were obtained from univariable models and multivariable models. Univariable models included only the variable of interest. Multivariable models were controlled for age, sex, history of atrial fibrillation, pre-existing chronic heart failure, premorbid modified Rankin Scale (mRS) score, occlusion site, baseline ASPECTS, baseline National Institutes of Health Stroke Scale (NIHSS), stroke etiology, expanded Treatment in Cerebral Infarction (eTICI) grade, and onset to recanalization time.

**Abbreviations:** ASPECTS, Alberta Stroke Program Early CT Score; eTICI, expanded Treatment in Cerebral Infarction; NIHSS, National Institutes of Health Stroke Scale; mRS, modified Rankin Scale; M1, M1 segment of the middle cerebral artery; M2, M2 segment of the middle cerebral artery; NIHSS, National Institutes of Health Stroke Scale.

**Table E in S1 Text Interaction models for National Institutes of Health Stroke Scale score and cardioembolism.**

| **Models** | ***p*** | **FDR-**  **adjusted *p*** | **Bonferroni-**  **adjusted *p*** |
| --- | --- | --- | --- |
| **Nonlinear interaction (RCS)^a^** | 0.953 | 0.953 | 1.000 |
| **Categorical interaction^b^** | 0.227 | 0.341 | 0.681 |
| **Linear interaction (continuous)^c^** | 0.016 | 0.048 | 0.048 |

^a^ Restricted cubic spline (RCS) by mixed-effects model with 3 knots for NIHSS × cardioembolism interaction. The *p* value for nonlinear interaction component. Model was adjusted for age, sex, baseline National Institutes of Health Stroke Scale score (NIHSS), Baseline Alberta Stroke Program Early CT Score (ASPECTS), history of atrial fibrillation, pre-existing chronic heart failure, premorbid modified Rankin Scale (mRS) score, occlusion site, expanded Treatment in Cerebral Infarction (eTICI), stroke etiology and onset to recanalization time.

^b^ *p* value for categorical NIHSS × cardioembolism interaction. Mixed-effects model was adjusted for age, sex, baseline NIHSS, baseline ASPECTS, history of atrial fibrillation, pre-existing chronic heart failure, premorbid mRS score, occlusion site, eTICI, Stroke etiology and onset to recanalization time.

^c^ *p* value for linear NIHSS × cardioembolism interaction. Mixed-effects model was adjusted for age, sex, baseline NIHSS, baseline ASPECTS, history of atrial fibrillation, pre-existing chronic heart failure, premorbid mRS score, occlusion site, eTICI, Stroke etiology and onset to recanalization time.

**Abbreviations:** ASPECTS, Alberta Stroke Program Early CT Score; eTICI, expanded Treatment in Cerebral Infarction; FDR, false discovery rate; mRS, modified Rankin Scale; NIHSS, National Institutes of Health Stroke Scale; RCS, Restricted cubic spline.

**Table F in S1 Text Sensitivity analysis adjusting for time-varying confounding in mediation analysis.**

| Models | Proportion  [95% CI] | *p* | FDR-  adjusted *p* | Bonferroni-adjusted *p* |
| --- | --- | --- | --- | --- |
| Model 1 | 7.70%  [2.40%, 12.40%] | 0.006 | 0.008 | 0.024 |
| Model 2 | 8.35% [2.26%, 12.62%] | 0.006 | 0.008 | 0.024 |
| Model 3 | 7.96% [2.37%, 12.16%] | 0.010 | 0.010 | 0.040 |
| Model 4 | 8.29%  [2.36%, 12.50%] | 0.006 | 0.008 | 0.024 |

The *p* values and 95% confidence intervals were estimated using nonparametric bootstrap resampling with 1,000 simulations.

**Model 1** was adjusted for all confounders in T0 stage in Method B of S1 Text.

**Model 2** was adjusted for all confounders in T0 stage plus expanded Treatment in Cerebral Infarction (eTICI) in Method B of S1 Text.

**Model 3** was adjusted for all confounders in T0 stage plus onset to recanalization time in Method B of S1 Text.

**Model 4** was adjusted for all confounders in T0 stage and T1 stage in Method B of S1 Text.

**Abbreviations:** CI, confidence interval; eTICI, expanded Treatment in Cerebral Infarction; FDR, false discovery rate.

**Table G in S1 Text Sensitivity analysis.** Adjusted association between post-stroke acute heart failure and outcomes by fixed-effects models.

| Outcomes | Before matching | | | | | | | PSM | | |
| --- | --- | --- | --- | --- | --- | --- | --- | --- | --- | --- |
|  | **All**  **(N=3415)** | **No-PSHF**  **(N=3137)** | **PSHF**  **(N=278)** | **Treatment effect** | **Effect value**  **[95% CI]** | **Adjusted**  **effect value [95% CI]** | ***p*^a^** | **Effect value**  **[95% CI]** | **Adjusted**  **effect value [95% CI]** | ***p*** |
| Primary outcome |  |  |  |  |  |  |  |  |  |  |
| Modified Rankin scale score of 5-6 at 90 days, n (%) | 2267  (66.38) | 975  (31.08) | 173  (62.23) | Odds  ratio | 3.65  [2.83, 4.72] | 3.01  [2.20, 4.12] | <0.001 | 2.31  [1.65, 3.26] | 3.02  [2.06, 4.47] | <0.001 |
| Secondary outcome |  |  |  |  |  |  |  |  |  |  |
| Score on the modified Rankin scale at 90 days, median [IQR]^b^ | 3.00  [1.00,6.00] | 3.00  [1.00,5.00] | 6.00  [3.00,6.00] | Common odds ratio | 3.52  [2.79, 4.46] | 2.77  [2.12, 3.63] | <0.01 | 2.32  [1.70, 3.17] | 2.96  [2.14, 4.11] | <0.001 |
| Modified Rankin scale score of 0-3 at 90 days, n (%) | 1850  (54.17) | 1772 (56.49) | 78  (28.06) | Odds  ratio | 0.30  [0.23, 0.39] | 0.39  [0.28, 0.54] | <0.001 | 0.40  [0.28, 0.57] | 0.33  [0.21, 0.49] | <0.001 |
| Modified Rankin scale score of 0-2 at 90 days, n (%) | 1401  (41.02) | 1350 (43.03) | 51  (18.35) | Odds  ratio | 0.30  [0.22, 0.40] | 0.38  [0.26, 0.54] | <0.001 | 0.33  [0.22, 0.49] | 0.35  [0.22, 0.54] | <0.001 |
| Mortality within 90 days, n (%) | 922  (27.00) | 771  (24.58) | 151  (54.32) | Hazard Ratio | 2.68  [2.25, 3.19] | 1.99  [1.62, 2.43] | <0.001 | 1.79  [1.39, 2.32] | 2.01  [1.54, 2.62] | <0.001 |
| NIHSS at 5-7 day or at early discharge, median [IQR]^c^ | 12.00  [4.00,26.00] | 11.00  [3.00,23.00] | 23.00  [10.00,36.00] | β coefficient | 8.56  [6.90, 10.22] | 6.38  [4.84, 7.93] | <0.001 | 5.84  [3.39, 8.28] | 5.89  [3.84, 7.95] | <0.001 |

The *p* values for binary outcomes were derived from logistic regression, for mortality from Cox proportional hazards regression, and for continuous outcomes from linear regression.

^a^ Adjusted fixed models were adjusted for age, sex, history of atrial fibrillation, pre-existing chronic heart failure, premorbid modified Rankin Scale, occlusion site, baseline Alberta Stroke Program Early CT Score, baseline National Institutes of Health Stroke Scale, stroke etiology (Trial of Org 10172 in Acute Stroke Treatment), expanded Thrombolysis In Cerebral Infarction, and onset to recanalization time.

^b^ Adjusted ordinal logistic regression was performed as a sensitivity analysis by adjusted for variables as fixed models, noting violation of the proportional odds assumption (*p* from Brant test <0.001).

^c^ were missing for 5 patients.

**Abbreviations:** CI, confidence interval; IQR, interquartile range; NIHSS, National Institutes of Health Stroke Scale; OR, odds ratio; PSM, propensity score matching; PSHF, post-stroke heart failure

**Table H in S1 Text Sensitivity analysis.** Adjusted association between post-stroke acute heart failure and outcomes by incorporating source studies as one of the adjustment covariates into the fixed-effects models.

| **Outcomes** | **Before matching** | | | | | | | **PSM** | | |
| --- | --- | --- | --- | --- | --- | --- | --- | --- | --- | --- |
|  | **All**  **(N=3415)** | **No-PSHF**  **(N=3137)** | **PSHF**  **(N=278)** | **Treatment effect** | **Effect value [95% CI]** | **Adjusted**  **effect value [95% CI]** | ***p*^a^** | **Effect value [95% CI]** | **Adjusted**  **effect value [95% CI]** | ***p*** |
| **Primary outcome** |  |  |  |  |  |  |  |  |  |  |
| **Modified Rankin scale score of 5-6 at 90 days, n (%)** | 2267  (66.38) | 975  (31.08) | 173  (62.23) | Odds  ratio | 3.79  [2.92, 4.95] | 3.00  [2.20, 4.11] | <0.001 | 2.46  [1.73, 3.51] | 3.11  [2.11, 4.53] | <0.001 |
| **Secondary outcome** |  |  |  |  |  |  |  |  |  |  |
| **Score on the modified Rankin scale at 90 days, median [IQR]^b^** | 3.00  [1.00, 6.00] | 3.00  [1.00, 5.00] | 6.00  [3.00, 6.00] | Common odds ratio | 3.49  [2.76, 4.44] | 2.78  [2.13, 3.65] | <0.001 | 2.35  [1.72, 3.23] | 3.02  [2.18, 4.20] | <0.001 |
| **Modified Rankin scale score of 0-3 at 90 days, n (%)** | 1850  (54.17) | 1772 (56.49) | 78  (28.06) | Odds  ratio | 0.29  [0.22, 0.38] | 0.38  [0.27, 0.53] | <0.001 | 0.39  [0.27, 0.55] | 0.32  [0.21, 0.48] | <0.001 |
| **Modified Rankin scale score of 0-2 at 90 days, n (%)** | 1401  (41.02) | 1350 (43.03) | 51  (18.35) | Odds  ratio | 0.30  [0.21, 0.40] | 0.37  [0.26, 0.53] | <0.001 | 0.32  [0.22, 0.48] | 0.34  [0.22, 0.53] | <0.001 |
| **Mortality within 90 days, n (%)** | 922  (27.00) | 771  (24.58) | 151  (54.32) | Hazard Ratio | 2.50  [2.10, 2.98] | 1.99  [1.62, 2.43] | <0.001 | 1.79  [1.39, 2.32] | 2.00  [1.53, 2.61] | <0.001 |
| **NIHSS at 5-7 day or at early discharge, median [IQR]^c^** | 12.00  [4.00, 26.00] | 11.00  [3.00, 23.00] | 23.00  [10.00, 36.00] | β coefficient | 8.26  [6.65, 9.88] | 6.38  [4.83, 7.92] | <0.001 | 5.90  [3.45, 8.33] | 5.94  [3.88, 8.00] | <0.001 |

The *p* values for binary outcomes were derived from logistic regression, for mortality from Cox proportional hazards regression, and for continuous outcomes from linear regression.

^a^ Adjusted models were adjusted for age, sex, history of atrial fibrillation, pre-existing chronic heart failure, premorbid modified Rankin Scale, occlusion site, baseline Alberta Stroke Program Early CT Score, baseline National Institutes of Health Stroke Scale, stroke etiology (Trial of Org 10172 in Acute Stroke Treatment), expanded Thrombolysis In Cerebral Infarction, and onset to recanalization time.

^b^ Adjusted ordinal logistic regression was performed as a sensitivity analysis, noting violation of the proportional odds assumption (P from Brant test < 0.001).

^c^ were missing for 5 patients

**Abbreviations:** CI, confidence interval; IQR, interquartile range; NIHSS, National Institutes of Health Stroke Scale; PSM, propensity score matching; PSHF, post-stroke heart failure.

**Table I in S1 Text. Sensitivity analysis.** Mixed-effects models incorporating study×post-stroke acute heart failure(PSHF) interaction for the association between PSHF and clinical outcomes.

| Outcomes | All  (N=3415) | No-PSHF  (N=3137) | PSHF  (N=278) | Treatment  effect | Effect value^a^ [95% CI] | Adjusted effect value^a^ [95% CI] | *p _for Heterogeneity_^b^* |
| --- | --- | --- | --- | --- | --- | --- | --- |
| Primary outcome |  |  |  |  |  |  |  |
| Modified Rankin scale score of 5-6 at 90 days, n (%) | 2267  (66.38) | 975  (31.08) | 173  (62.23) | Odds  ratio | 2.17  [1.14, 4.14] | 3.01  [2.94, 5.54] | 0.136 |
| Secondary outcome |  |  |  |  |  |  |  |
| Score on the modified Rankin scale at 90 days, median [IQR]^c^ | 3.00  [1.00,6.00] | 3.00  [1.00,5.00] | 6.00  [3.00,6.00] | Common odds ratio | 2.40  [1.35, 4.27] | 2.94  [1.63, 5.29] | 0.108 |
| Modified Rankin scale score of 0-3 at 90 days, n (%) | 1850  (54.17) | 1772  (56.49) | 78  (28.06) | Odds  ratio | 0.47  [0.23, 0.96] | 0.40  [0.29, 0.57] | 0.129 |
| Modified Rankin scale score of 0-2 at 90 days, n (%) | 1401  (41.02) | 1350  (43.03) | 51  (18.35) | Odds  ratio | 0.31  [0.13, 0.74] | 0.36  [0.25, 0.53] | 0.866 |
| Mortality within 90 days, n (%) | 922  (27.00) | 771  (24.58) | 151  (54.32) | Hazard  Ratio | 2.55  [2.13, 3.05] | 2.05  [1.67, 2.52] | 0.087 |
| NIHSS at 5-7 day or at early discharge, median [IQR]^d^ | 12.00  [4.00,26.00] | 11.00  [3.00,23.00] | 23.00  [10.00,36.00] | β  coefficient | 5.15  [1.48, 8.82] | 6.41  [4.84, 7.99] | 0.059 |

The *p* values for binary outcomes were derived from logistic regression, for mortality from Cox proportional hazards regression, and for continuous outcomes from linear regression.

^a^ Mixed-effects models incorporating both study-level and center-level random effects with PSHF×study interactions. Models were adjusted for age, sex, history of atrial fibrillation, pre-existing chronic heart failure, premorbid modified Rankin Scale, occlusion site, baseline Alberta Stroke Program Early CT Score, baseline National Institutes of Health Stroke Scale, stroke etiology (Trial of Org 10172 in Acute Stroke Treatment), expanded Thrombolysis In Cerebral Infarction, and onset to recanalization time. The effect values were estimated using sample size-weighted averages across studies.

^b^ The *p* values for heterogeneity tests were assessed by likelihood ratio tests comparing adjusted mixed-effects models with and without PSHF×study interactions, assessing whether the effect of PSHF varied significantly across studies

^c^ Ordinal logistic regression was performed as a sensitivity analysis, noting that study-level interaction was not assessed for the Win Ratio given its pooled pairwise comparison structure.

^d^ were missing for 5 patients.

**Abbreviations:** CI, confidence interval; IQR, interquartile range; NIHSS, National Institutes of Health Stroke Scale; PSHF, post-stroke heart failure.

**Table J in S1 Text Sensitivity analysis.** Pharmacological interventions in each trial did not significantly influence the incidence of post-stroke acute heart failure.

| **Trials** | **OR [95% CI]** | **aOR [95% CI]^a^** | ***p*** | ***p* _for heterogeneity_** |
| --- | --- | --- | --- | --- |
| RESCUE-BT | 0.58  [0.31, 1.09] | 0.56  [0.29, 1.08] | 0.081 | 0.208 |
| DEVT | 1.67  [0.35, 8.01] | 1.08  [0.20, 5.94] | 0.929 |  |
| MARVEL | 1.06  [0.76, 1.47] | 1.06  [0.73, 1.54] | 0.761 |  |

^a^ Adjusted odds ratios (aOR) and 95% confidence intervals (CI) were derived from mixed-effects logistic regression models. The *p* for heterogeneity was assessed by likelihood ratio tests comparing models with vs. without treatment×study interaction terms. Models were adjusted for age, sex, history of atrial fibrillation, pre-existing chronic heart failure, premorbid modified Rankin Scale, occlusion site, baseline Alberta Stroke Program Early CT Score, baseline National Institutes of Health Stroke Scale, stroke etiology (Trial of Org 10172 in Acute Stroke Treatment), expanded Thrombolysis In Cerebral Infarction, and onset to recanalization time.

The *p* for heterogeneity was assessed using likelihood ratio tests comparing models with and without treatment-by-study interaction terms.

**Abbreviations:** aOR, adjusted odds ratio; CI, confidence interval; OR, odds ratio.

**Table K in S1 Text Sensitivity analysis.** The effects of trial interventions on 90-day functional outcome did not significantly differ between patients with and without post-stroke acute heart failure.

| **Trials** | **PSHF status** | **OR [95% CI]** | **aOR [95% CI]^a^** | ***p* _for interaction_** |
| --- | --- | --- | --- | --- |
| RESCUE-BT | Without PSHF | 1.13  [0.81, 1.57] | 1.18  [0.82, 1.68] | 0.834 |
|  | With PSHF | 1.15  [0.33, 4.05] | 1.38  [0.34, 5.61] |  |
| DEVT | Without PSHF | 0.73  [0.04, 13.45] | 0.44  [0.09, 2.18] | 0.544 |
|  | With PSHF | 1.93  [0.55, 6.76] | 1.38  [0.06, 33.33] |  |
| MARVEL | Without PSHF | 0.78  [0.62, 0.98] | 0.76  [0.59, 0.98] | 0.711 |
|  | With PSHF | 0.68  [0.36, 1.28] | 0.66  [0.33, 1.35] |  |

^a^ Adjusted odds ratios (aOR) and 95% confidence intervals (CI) were derived from logistic regression models. The *p* for interaction indicates whether the treatment effect differed significantly between PSHF groups. Models were adjusted for age, sex, history of atrial fibrillation, pre-existing chronic heart failure, premorbid modified Rankin Scale, occlusion site, baseline Alberta Stroke Program Early CT Score, baseline National Institutes of Health Stroke Scale, stroke etiology (Trial of Org 10172 in Acute Stroke Treatment), expanded Thrombolysis In Cerebral Infarction, and onset to recanalization time.

The *p* values for interaction were derived from logistic regression models including an interaction term between randomized intervention and PSHF status, testing whether the effect of trial intervention on very poor outcome differed between patients with and without PSHF.

**Abbreviations:** aOR, adjusted odds ratio; CI, confidence interval; OR, odds ratio; PSHF, post-stroke acute heart failure.

**Table L in S1 Text** The association between post-stroke acute heart failure and Utility-weighted modified Rankin Scale (UW-mRS).

| **Outcome** | **No-PSHF**  **(N=3137)** | **PSHF**  **(N=278)** | **β**  **[95% CI]** | **Adjusted β**  **[95% CI]^a^** | ***p*** |
| --- | --- | --- | --- | --- | --- |
| **MOST** | 6.50  [0.00,9.10] | 0.00  [0.00,6.50] | -2.45  [-2.91, -1.99] | -1.64  [-2.08, -1.194] | <0.001 |
| **Hong** | 3.50  [0.00,7.90] | 0.00  [0.00,3.50] | -2.06  [-2.49, -1.60] | -1.30  [-1.73, -0.87] | <0.001 |
| **Rivero** | 6.20  [1.10,9.30] | 0.00  [0.00,6.20] | -2.48  [-2.93, -2.04] | -1.73  [-2.16, -1.30] | <0.001 |

^a^ Linear mixed models were used to assess the association between post-stroke acute heart failure (PSHF) and UW-mRS scores. The adjusted models controlled for age, sex, history of atrial fibrillation, pre-existing chronic heart failure, premorbid modified Rankin Scale, occlusion site, baseline Alberta Stroke Program Early CT Score, baseline National Institutes of Health Stroke Scale, stroke etiology (Trial of Org 10172 in Acute Stroke Treatment), expanded Thrombolysis In Cerebral Infarction, and onset to recanalization time. The *p* values were derived from Wald tests based on linear mixed models.

**Abbreviations:** CI, confidence interval; PSHF, post-stroke acute heart failure; UW-mRS, utility-weighted modified Rankin Scale.

**Table M in S1 Text** The association between post-stroke acute heart failure and quality-of-life.

| **Trials** | **Indicator of quality-of-life** | **No-PSHF** | **PSHF** | **β**  **[95% CI]** | **Adjusted β**  **[95% CI]^a^** | ***p*** |
| --- | --- | --- | --- | --- | --- | --- |
| **DEVT & RESCUE-BT** | EQ-5D-5Lscore at 90 days | 0.73  [0.19, 0.96] | 0.00 [0.00,0.62] | -0.30  [-0.40, -0.21] | -0.20  [-0.28, -0.11] | <0.001 |
| **MARVEL** | EQ-5D-VAS score at 90 days | 55.00  [2.50, 80.00] | 0.00 [0.00,50.00] | -25.15  [-31.09, -19.20] | -18.84  [-24.74, -12.93] | <0.001 |

^a^ Adjusted linear models were used to assess the association between post-stroke acute heart failure (PSHF) and UW-mRS scores, adjusted for age, sex, history of atrial fibrillation, pre-existing chronic heart failure, premorbid modified Rankin Scale, occlusion site, baseline Alberta Stroke Program Early CT Score, baseline National Institutes of Health Stroke Scale, stroke etiology (Trial of Org 10172 in Acute Stroke Treatment), expanded Thrombolysis In Cerebral Infarction, and onset to recanalization time. The *p* values were derived from linear mixed models. Detailed information on the quality-of-life indicator is provided in Method D of S1 Text.

**Abbreviations:** PSHF, post-stroke acute heart failure; CI, confidence interval.

**Table N in S1 Text** Effects of adjunctive application of methylprednisolone on the incidence of post-stroke acute heart failure among patients in the MARVEL trial.

| Patients | Without-  Methylprednisolone  , n (%) | Methylpred  nisolone  , n (%) | HR  [95%CI] | aHR  [95%CI]^a^ | *p* | FDR-adjusted  *p* | Bonferroni-adjusted *p* |
| --- | --- | --- | --- | --- | --- | --- | --- |
| Cardioembolism  and NIHSS>16 | 42/297  (14.14) | 60/328  (18.29) | 0.95  [0.55, 1.63] | 0.92  [0.53,1.62] | 0.785 | 0.785 | 1.000 |
| Cardioembolism  and NIHSS≤16 | 8/88  (9.09) | 11/87  (12.64) | 0.81  [0.26, 2.54] | 0.82  [0.26,2.61] | 0.734 | 0.785 | 1.000 |
| Non-cardioembolism  and NIHSS > 16 | 21/319  (6.58) | 9/287  (3.14) | 0.53  [0.18, 1.54] | 0.65  [0.19, 2.17] | 0.484 | 0.785 | 1.000 |
| No-cardioembolism  and NIHSS≤16 | 6/127  (4.72) | 1/126  (0.79) |  | - | - | - | - |

^a^Adjusted hazard ratios (HR) and 95% confidence intervals (CI) were derived from time-dependent Cox proportional hazards models. P values were derived from the Cox models. FDR and Bonferroni corrections were applied for multiple comparisons across subgroups.

^a^Time-dependent Cox proportional hazards models were used with time-zero at the randomization time of MARVEL trial. Models were adjusted for age, sex, history of atrial fibrillation, pre-existing chronic heart failure, premorbid mRS score (modified Rankin Scale), occlusion site, baseline ASPECTS (Alberta Stroke Program Early CT Score), eTICI (expanded Thrombolysis In Cerebral Infarction), and onset to recanalization time.

Important caveats: This analysis was conducted post hoc after observing the association between PSHF, cardioembolism, and stroke severity. Results should be considered purely hypothesis-generating with no therapeutic implications.

**Abbreviations:** aHR, adjusted hazard ratio; CI, confidence interval; FDR, false discovery rate; HR, hazard ratio; NIHSS, National Institutes of Health Stroke Scale; PSHF, post-stroke acute heart failure.

**Table O in S1 Text** The impact of methylprednisolone on the frequency of very poor outcomes among post-stroke acute heart failure in MARVEL trial.

| Patients | Without-  Methylprednisolone  , n (%) | Methylpred  nisolone  , n (%) | OR  [95% CI] | aOR  [95% CI]^a^ | *p* | FDR-  adjusted *p* | Bonferroni-adjusted *p* |
| --- | --- | --- | --- | --- | --- | --- | --- |
| Cardioembolism  and NIHSS>16 | 30/42  (71.43) | 32/60  (53.33) | 0.46  [0.19, 1.04] | 0.33  [0.11, 0.85] | 0.027 | 0.054 | 0.054 |
| Cardioembolism  and NIHSS≤16 | 2/8  (25.00) | 5/11  (45.45) | - | - | - | - | - |
| Non-cardioembolism  and NIHSS > 16 | 15/21  (71.43) | 7/9  (77.78) | 1.4  [0.24, 11.23] | 2.02  [0.20, 27.18] | 0.559 | 0.559 | 1.000 |
| Non-cardioembolism  and NIHSS≤16 | 3/6  (50.00) | 1/1  (100.00) | - | - | - | - | - |

^a^ Adjusted models were adjusted for age, sex, history of atrial fibrillation, pre-existing chronic heart failure, premorbid modified Rankin Scale, occlusion site, baseline Alberta Stroke Program Early CT Score, baseline National Institutes of Health Stroke Scale, expanded Thrombolysis In Cerebral Infarction, and onset to recanalization time. The *p* values were derived from the logistic regression model coefficients.

Important caveats: This analysis was conducted post hoc after observing the association between PSHF, cardioembolism, and stroke severity. Results should be considered purely hypothesis-generating with no therapeutic implications. The borderline statistical significance (FDR-corrected P=0.054) and small sample size indicate inadequate evidence for clinical decision-making.

**Abbreviations:** aOR, adjusted odds ratio; CI, confidence interval; FDR, false discovery rate; NIHSS, National Institutes of Health Stroke Scale; OR, odds ratio; PSHF, post-stroke acute heart failure.

**Supplemental Method**

**Method A in S1 Text** Key features distinguishing post-stroke acute heart failure from competing cardiopulmonary conditions.

| **Condition** | **Key Distinguishing Features** | **Natriuretic Peptides** | **Chest Imaging** | **Typical Course** |
| --- | --- | --- | --- | --- |
| PSHF | ESC criteria: elevated NP + ≥3 symptoms/signs of congestion | Elevated above age-adjusted thresholds | Bilateral infiltrates, pleural effusion, vascular congestion | Peaks at 24-72 hours, responds to HF therapy |
| Pneumonia | Fever, productive cough, leukocytosis, focal signs | Normal or mildly elevated | Focal consolidation, air bronchograms | Infectious symptoms, antibiotic response |
| Pulmonary embolism | Abrupt dyspnea, tachycardia, pleuritic pain, no congestion signs | Normal or mildly elevated | Normal, wedge-shaped opacities, or dilated RV | Abrupt onset, CT-PA confirmation |
| Fluid overload | History of excessive intravenous injection fluids, renal dysfunction, minimal cardiac symptoms | Normal or minimally elevated | Variable, often mild | Resolves with fluid restriction |
| Neurogenic pulmonary edema | Severe brain injury, very early onset (within hours), rapid spontaneous resolution | Normal or mildly elevated | Diffuse, central, or "butterfly" pattern, clears rapidly | Self-limited, hours to 48 hours |

**Abbreviations:** CT-PA, computed tomography pulmonary angiography; ESC, European Society of Cardiology; HF, heart failure; NP, natriuretic peptide; PSHF, post-stroke acute heart failure; RV, right ventricle.

**Method B in S1 Text** An overview of the variables and measurements in the study.

| **Variable** | **Measurement instrument** | **Timing of assessment*** | **Measurement level applied in analysis** |
| --- | --- | --- | --- |
| **Confounders** |  |  |  |
| Age | Age in years | T0 | Continuous variable |
| Sex | Female or Male | T0 | Binary variable |
| Atrial fibrillation | Dichotomous: with a history of atrial fibrillation before or not | T0 | Binary variable |
| Pre-existing chronic heart failure | Dichotomous: with a history of heart failure before or not | T0 | Binary variable |
| Premorbid modified Rankin Scale | The modified Rankin Scale (mRS) is the 'gold standard' for assessing long-term functional outcomes in stroke clinical research. The scale ranges from 0 [no symptoms] to 6 [death], where scores represent: 0, no symptoms; 1, no significant disability; 2, slight disability; 3, moderate disability; 4, moderately severe disability; 5, severe disability; and 6, death [1] | T0 | Ordinal variable |
| Occlusion site | Classification of stroke site: Intracranial internal carotid artery, the first or second segment of middle cerebral artery | T0 | Classified variable |
| TOAST | Classification of stroke etiology: large artery atherosclerosis, cardioembolism, other or unknown causes[2] | T0 | Classified variable |
| NIHSS | The NIHSS comprises 11 items that assess neurological deficits, including consciousness, sensation, motor function, etc. Scores range from 0 to 42, with higher scores indicating greater stroke severity[3] | T0 | Continuous variable |
| ASPECTS | The ASPECTS divides the middle cerebral artery territory into 10 specific regions on baseline CT/MRI, assigning one point to each region devoid of early ischemic changes. Scores range from 0 to 10. A higher score indicates a smaller infarct volume[4] | T0 | Continuous variable |
| Onset to recanalization | The time from stroke onset to recanalization of vessels | T1 | Continuous variable |
| eTICI | The extended Treatment in Cerebral Infarction (eTICI) scale is currently the 'gold standard' for assessing post-procedural vascular recanalization in endovascular stroke therapy. The grades are defined as follows: Grade 0, no recanalization; Grade 1, minimal recanalization; Grade 2a, partial recanalization; Grade 2b, suboptimal recanalization; Grade 2c, near-complete recanalization; and Grade 3, complete recanalization[5] | T1 | Ordinal variable |
| **Outcomes** |  |  |  |
| PSHF | PSHF is defined as new-onset or acutely worsening heart failure occurring within 7 days after stroke onset, diagnosed based on the 2021 ESC guidelines requiring simultaneous elevation of natriuretic peptides and the presence of characteristic symptom | T2 | Binary variable |
| 90-day mRS score | The modified Rankin Scale (mRS) is the 'gold standard' for assessing long-term functional outcomes in stroke clinical research. The scale ranges from 0 [no symptoms] to 6 [death], where scores represent: 0, no symptoms; 1, no significant disability; 2, slight disability; 3, moderate disability; 4, moderately severe disability; 5, severe disability; and 6, death [1] | T3 | Ordinal variable |
| 90-day mRS score 0-2/0-3/5-6 | Dichotomous: with 90-day mRS score 0-2/0-3/5-6 or not | T3 | Binary variable |
| Mortality at 90 days | Dichotomous: with 90-day mRS score 6 or not | T3 | Binary variable |
| NIHSS at 5-7 day or early discharge | The NIHSS comprises 11 items that assess neurological deficits, including consciousness, sensation, motor function, etc. Scores range from 0 to 42, with higher scores indicating greater stroke severity[3] | T2 | Continuous variable |

**Abbreviations:** mRS score (modified Rankin Scale), ASPECTS (Alberta Stroke Program Early CT Score), NIHSS (National Institutes of Health Stroke Scale), stroke etiology (Trial of Org 10172 in Acute Stroke Treatment), eTICI (expanded Thrombolysis In Cerebral Infarction)

*T0 = Baseline, T1 = Post-procedure, T2 = 5-7 day or at early discharge, T3 = 3 months

**Short narrative of literature supporting the selection of confounders.**

Confounders were selected supported by previous studies and reasoning connected to clinical course and prognosis in ischemic stroke. The following factors were identified as significant for prognosis in systematic reviews: age[6], sex[7,8], hypertension[9], atrial fibrillation[10], pre-existing chronic heart failure[11], pre-stroke mRS score[12], occlusion site[13], TOAST[14,15], baseline NIHSS[16], baseline ASPECTS[17], onset to recanalization[16], eTICI[18] was also identified as potential confounders based on previous studies.

The selection of covariates was guided by established evidence and clinical rationale regarding outcomes after ischemic stroke. The following factors were identified as established predictors of PSHF and were therefore included in the analysis: age [19], sex [20], atrial fibrillation [21], pre-existing chronic heart failure [22], pre-stroke mRS score [23], occlusion site [24], TOAST classification [25], baseline NIHSS score [26], baseline ASPECTS [27], onset to recanalization time [28], and eTICI score [29], all of which have been consistently reported as independent predictors of PSHF. These variables were therefore incorporated as potential confounders in the analysis.

**Method C in S1 Text** Hierarchical adjustment strategies for sensitivity analysis of covariate selection.

To evaluate the robustness of the pre-specified covariate set, we performed sensitivity analyses using four nested adjustment models. Covariates were temporally classified as pre-exposure (T0) or post-treatment (T1) based on their temporal relationship to stroke onset and endovascular treatment (Method B in S1 Text). T0 variables included age, sex, baseline National Institutes of Health Stroke Scale (NIHSS) score, baseline Alberta Stroke Program Early CT Score (ASPECTS), history of atrial fibrillation, pre-existing chronic heart failure, premorbid modified Rankin Scale (mRS) score, occlusion site, and stroke etiology (TOAST classification). T1 variables comprised expanded Treatment in Cerebral Infarction (eTICI) grade and onset to recanalization time.

The four models were defined as follows:

Model 1: adjusted only for demographic characteristics (age and sex);

Model 2: adjusted for all T0 variables in Method B of S1 Text;

Model 3: adjusted for all T1 variables in Method B of S1 Text;

Model 4: adjusted for all T0 and T1 variables (primary analysis model) in Method B of S1 Text;

| **Outcome** | **No-PSHF**  **(N=3137)** | **PSHF**  **(N=278)** | **Adjusted OR [95% CI]** | ***p*** |
| --- | --- | --- | --- | --- |
| **Model 1** | 975  (31.08) | 173  (62.23) | 3.29  [2.50, 4.33] | <0.001 |
| **Model 2** | 975  (31.08) | 173  (62.23) | 2.94  [2.14, 4.03] | <0.001 |
| **Model 3** | 975  (31.08) | 173  (62.23) | 4.11  [3.14, 5.40] | <0.001 |
| **Model 4** | 975  (31.08) | 173  (62.23) | 3.09  [2.25, 4.24] | <0.001 |

All models were fitted using mixed-effects logistic regression with random intercepts for study and center to account for clustering. The *p* values were from the mixed-effects logistic regression models. The effect of PSHF on very poor outcome remained stable from the model adjusted only for baseline (T0) confounders (adjusted odds ratio (aOR) 2.94, 95% confidence interval (CI), 95% CI [2.14, 4.03]; *p*<0.001) to the primary model adjusting for both T0 and T1 variables (aOR 3.09, 95% CI [2.25, 4.24]; *p*<0.001), with less than 5% relative change when T1 variables were added. Notably, adjustment restricted to T1 variables alone yielded a larger estimate (aOR 4.11, 95% CI [3.14, 5.40]; *p*<0.001), demonstrating omitted variable bias when baseline confounders were excluded.

**Abbreviations:** aOR, odds ratio; CI, confidence interval; PSHF, post-stroke acute heart failure.

**Method D in S1 Text** Additional outcome.

**Utility-weighted modified Rankin Scale (UW-mRS)**

We calculated utility-weighted mRS scores using three established approaches to ensure robustness: (1) weights derived from the HERMES collaboration (MOST method), (2) weights from the MR CLEAN trial (Hong method), and (3) utility weights from the original mRS utility study by Rivero et al. (Rivero method). For each approach, utility weights were applied to the 90-day mRS score to compute UW-mRS scores ranging from 0 (death) to 1 (perfect health) [30-32]. Linear mixed models were used to assess the association between PSHF and UW-mRS scores, adjusted for age, sex, baseline ASPECTS, history of atrial fibrillation, history of previous heart failure, premorbid mRS score, occlusion site, eTICI, and onset to recanalization time.

**Quality-of-life assessment**

Quality-of-life was assessed using trial-specific instruments: EQ-5D-5L in the DEVT and RESCUE-BT trials, and EQ-5D-VAS in the MARVEL trial. The EQ-5D-5L index score (ranging from -0.391 to 1.000, where 1 represents full health) and EQ-5D-VAS score (ranging from 0 to 100, where 100 represents the best imaginable health state) were measured at 90 days after stroke [33, 34]. The quality-of-life was not measured in the BASILAR registry. Given differences in study design, time periods (2014-2023), and assessment tools across trials, we analyzed the association between PSHF and quality-of-life outcomes within each individual study rather than pooling across trials. Linear regression models were adjusted for the same covariates as the primary analysis.

**References**

1. Liu C, Guo C, Li F, Yu N, Huang J, Peng Z, et al. Intra-Arterial Urokinase After Endovascular Reperfusion for Acute Ischemic Stroke: The POST-UK Randomized Clinical Trial. JAMA. 2025;333: 589–598. doi:10.1001/jama.2024.23480

2. Adams HP, Bendixen BH, Kappelle LJ, Biller J, Love BB, Gordon DL, et al. Classification of subtype of acute ischemic stroke. Definitions for use in a multicenter clinical trial. TOAST. Trial of Org 10172 in Acute Stroke Treatment. Stroke. 1993;24: 35–41. doi:10.1161/01.str.24.1.35

3. National Institute of Neurological Disorders and Stroke rt-PA Stroke Study Group. Tissue plasminogen activator for acute ischemic stroke. N Engl J Med. 1995;333: 1581–1587. doi:10.1056/NEJM199512143332401

4. Barber PA, Demchuk AM, Zhang J, Buchan AM. Validity and reliability of a quantitative computed tomography score in predicting outcome of hyperacute stroke before thrombolytic therapy. ASPECTS Study Group. Alberta Stroke Programme Early CT Score. Lancet. 2000;355: 1670–1674. doi:10.1016/s0140-6736(00)02237-6

5. Liebeskind DS, Bracard S, Guillemin F, Jahan R, Jovin TG, Majoie CB, et al. eTICI reperfusion: defining success in endovascular stroke therapy. J Neurointerv Surg. 2019;11: 433–438. doi:10.1136/neurintsurg-2018-014127

6. Kim BJ, Tang S-C, Hsieh Y-C, Chen C-H, Kim YS, Lin C-J, et al. Association Between Age and Endovascular Treatment Outcomes: Binational Registry of 9934 EVT Cases From Korea and Taiwan. Stroke. 2025;56: 2540–2549. doi:10.1161/STROKEAHA.124.050483

7. Ali M, Dekker L, Ali M, Van Zwet EW, Hofmeijer J, Nederkoorn PJ, et al. Sex differences in outcomes after endovascular treatment in posterior circulation stroke: results from the MR CLEAN Registry. J Neurointerv Surg. 2024;17: e74–e82. doi:10.1136/jnis-2023-021086

8. D S, None R, X H, B J, X T, G M, et al. Sex-Related Differences in Outcomes of Endovascular Treatment for Anterior Circulation Large Vessel Occlusion. Stroke. 2023;54. doi:10.1161/STROKEAHA.122.041195

9. Ferrari F, Villa RF. Brain bioenergetics in chronic hypertension: Risk factor for acute ischemic stroke. Biochem Pharmacol. 2022;205: 115260. doi:10.1016/j.bcp.2022.115260

10. Deng G, Xiao J, Yu H, Chen M, Shang K, Qin C, et al. Predictors of futile recanalization after endovascular treatment in acute ischemic stroke: a meta-analysis. J Neurointerv Surg. 2022;14: 881–885. doi:10.1136/neurintsurg-2021-017963

11. D’Anna L, Abu-Rumeileh S, Merlino G, Ornello R, Foschi M, Diana F, et al. Safety and Outcomes of Mechanical Thrombectomy in Acute Ischemic Stroke Attributable to Cardiological Diseases: A Scoping Review. J Am Heart Assoc. 2024;13: e034783. doi:10.1161/JAHA.124.034783

12. Khan MA, Baird GL, Miller D, Patel A, Tsekhan S, Yaghi S, et al. Endovascular treatment of acute ischemic stroke in nonagenarians compared with younger patients in a multicenter cohort. J Neurointerv Surg. 2017;9: 727–731. doi:10.1136/neurintsurg-2016-012427

13. Yang S, Wu L, Shi X, Guo C, Yue C, Fan S, et al. Effect of occlusion site on the effectiveness and safety of endovascular thrombectomy for large ischemic cores: a cohort study. Int J Surg. 2026;112: 762–771. doi:10.1097/JS9.0000000000002102

14. Thut MZ, Howaldt G, Krepuska M, Thurner P, Madjidyar J, Wegener S, et al. Revascularization strategies in acute stroke with tandem occlusions: The Impact of Dissection vs. Atherosclerotic etiology on Clinical Outcomes. J Stroke Cerebrovasc Dis. 2025;34: 108362. doi:10.1016/j.jstrokecerebrovasdis.2025.108362

15. Qiao Y, Zhao M, Wang J, Li S, Yang T, Wang P, et al. Stroke etiology was associated with tirofiban efficacy in acute ischemic stroke without endovascular treatment: A pre-specified subgroup analysis of the TREND trial. Int J Stroke. 2025;20: 977–986. doi:10.1177/17474930251326423

16. Gong C, Jiang S, Huang L, Wang Z, Chen Y, Huang Z, et al. Predicting Futile Recanalization by Cerebral Collateral Recycle Status in Patients with Endovascular Stroke Treatment: The CHANOA Score. Acad Radiol. 2025;32: 2876–2885. doi:10.1016/j.acra.2024.11.032

17. Xiong X, Yang D, Wan J, Yang Y, Fan S, Guo C, et al. Relationship between ASPECTS-region and clinical outcome in patients with large vessel occlusion stroke: a post-hoc analysis of randomized clinical trial. Int J Surg. 2026;112: 999–1006. doi:10.1097/JS9.0000000000003327

18. Chai M, Wan Q, Li L, Chen W, Zhu H, Liu J, et al. Impact of expanded Thrombolysis in Cerebral Infarction (eTICI) grades on clinical outcomes in patients with large ischemic strokes receiving endovascular treatment. J Neurol. 2025;272: 577. doi:10.1007/s00415-025-13327-2

19. Edrissi C, Rathfoot C, Knisely K, et al. Age Stratification in Acute Ischemic Stroke Patients with Heart Failure. J Clin Med 2022;12.

20. Joffe J, Stepp R, Faulkner M, Imeh-Nathaniel S, Imeh-Nathaniel A, Nathaniel TI. Sex differences in comorbidities associated with exclusion from thrombolytic therapy in ischemic stroke patients with heart failure: A retrospective data analysis. JRSM Cardiovasc Dis 2024;13:20480040241265785.

21. Zabierowski J, Hudzik B, Nowak J, Gąsior M. Emerging risk factors for stroke and bleeding in patients with atrial fibrillation and heart failure-a narrative review. Curr Probl Cardiol 2026;51:103231.

22. Tütüncü S, Olma MC, Kunze C, et al. Heart failure, recurrent vascular events and death in patients with ischemic stroke-results of the MonDAFIS study. Intern Emerg Med 2024;19:1247-1254.

23. Jiang X, Wang L, Morgenstern LB, Cigolle CT, Claflin ES, Lisabeth LD. New Index for Multiple Chronic Conditions Predicts Functional Outcome in Ischemic Stroke. Neurology 2021;96:e42-e53.

24. Rincon F, Dhamoon M, Moon Y, et al. Stroke location and association with fatal cardiac outcomes: Northern Manhattan Study (NOMAS). Stroke 2008;39:2425-2431.

25. Ntaios G, Papavasileiou V, Makaritsis K, Milionis H, Michel P, Vemmos K. Association of ischaemic stroke subtype with long-term cardiovascular events. Eur J Neurol 2014;21:1108-1114.

26. Micheli S, Agnelli G, Caso V, et al. Acute myocardial infarction and heart failure in acute stroke patients: frequency and influence on clinical outcome. J Neurol 2012;259:106-110.

27. Hill MD, Demchuk AM, Goyal M, et al. Alberta Stroke Program early computed tomography score to select patients for endovascular treatment: Interventional Management of Stroke (IMS)-III Trial. Stroke 2014;45:444-449.

28. Schnieder M, von Glasenapp A, Hesse A, et al. Heart Failure Is Not Associated with a Poor Outcome after Mechanical Thrombectomy in Large Vessel Occlusion of Cerebral Arteries. Stroke Res Treat 2019;2019:4695414.

29. Gentile L, Pracucci G, Saia V, et al. Mechanical thrombectomy in patients with heart failure: the Italian registry of Endovascular Treatment in Acute Stroke. Neurol Sci 2023;44:3577-3585.

30. Rivero-Arias O, Ouellet M, Gray A, Wolstenholme J, Rothwell PM, Luengo-Fernandez R. Mapping the modified Rankin scale (mRS) measurement into the generic EuroQol (EQ-5D) health outcome. Med Decis Making 2010;30:341-354.

31. Hong KS, Saver JL. Quantifying the value of stroke disability outcomes: WHO global burden ofdisease project disability weights for each level of the modified Rankin Scale. Stroke 2009;40:3828-3833.

32. Saver JL, Yafeh B. Confirmation of tPA treatment effect by baseline severity-adjusted end point reanalysis of the NINDS-tPA stroke trials. Stroke 2007;38:414-416.

33. Chen, P, Lin, K. C, Liing, R. J, Wu, C. Y, Chen, C. L, Chang, K. C. Validity, responsiveness, and minimal clinically important difference of EQ-5D-5L in stroke patients undergoing rehabilitation. Qual Life Res 2016;25:1585-1596.

34. Cheng, L. J, Chen, L. A, Cheng, J. Y, Herdman, M, Luo, N. Systematic review reveals that EQ-5D minimally important differences vary with treatment type and may decrease with increasing baseline score. J Clin Epidemiol 2024;174:111487.
