# Supplementary material for: Post-stroke acute heart failure in patients with large vessel occlusion undergoing endovascular treatment: A pooled analysis of individual patient data from multicenter studies with mediation analysis
Source: PLoS Med. 2026 Jul 7;23(7):e1004752. doi: 10.1371/journal.pmed.1004752 (PMC13340808; doi:10.1371/journal.pmed.1004752)
Supplement: S2 Text — (DOCX) [file pmed.1004752.s004.docx]

# S2 Text Analysis plan and updates

Below is the original analysis plan. In response to reviewer comments, several changes were made which are detailed here:

## Changes to primary analysis methods:

- Replaced traditional regression models with mixed-effects models as the primary analytical framework to account for potential heterogeneity across the four component studies and clustering within centers
- Mixed-effects logistic regression used for binary outcomes (very poor outcome, functional independence, independent ambulation)
- Mixed-effects ordinal logistic regression used for 90-day mRS distribution, with the proportional odds assumption tested by the Brant test. Once violation of this assumption, stratified inverse probability of treatment weighting (IPTW) with the Win Ratio will be implemented
- Linear mixed-effects models used for continuous outcomes (NIHSS at 5-7 days)
- Replaced Cox proportional hazards regression for logistical model
- Mixed-effects Cox proportional hazards regression used for mortality

## Changes to causal mediation analysis:

- Added detailed causal mediation analysis following AGReMA (A Guideline for Reporting Mediation Analyses) guidelines
- Added multiplicity correction using both Bonferroni and False Discovery Rate (FDR) methods for mediation tests

## Changes to interaction analysis:

- Added complementary tests for interaction: nonlinear interaction using restricted cubic splines (RCS) with three knots, and categorical interaction using NIHSS categorized into 5-point increments
- Added multiplicity correction using both Bonferroni and False Discovery Rate (FDR) methods for interaction tests

## Changes to subgroup and sensitivity analyses:

- Added pre-existing chronic heart failure as a subgroup variable (with vs. without)
- Added both Bonferroni and FDR corrections for multiple comparisons in subgroup
- analyses
- Added study-by-PSHF interaction terms in mixed-effects models with likelihood ratio tests
- Added examination of trial-specific intervention effects within each individual trial

## Changes to methylprednisolone analysis:

- Revised from focusing on global dataset to population in the MARVEL trial and added both Bonferroni and FDR corrections for multiple comparisons

-----------------------------------------------------------------------------------------

*Created: 2025-01-29 by Chang Liu*

*Updated: 2026-03-19 by Chang Liu*

# OBJECTIVE AND HYPOTHESES

Cardiac complications are common after stroke, and acute heart failure ranks among one of the most frequent cardiovascular emergencies[1]. However, evidence is lacking regarding the incidence, predictors, and clinical impacts of post-stroke acute heart failure (PSHF) in patients with large vessel occlusion stroke receiving endovascular treatment (EVT)[2]. Hence, the aim of this study is to assess the incidence, predictors, and clinical impact of post-stroke acute heart failure (PSHF) in patients with large vessel occlusion stroke (LVO) receiving endovascular treatment (EVT). The hypothesis is that PSHF is significantly associated with very poor outcomes in LVO patients undergoing EVT.

Cardioembolic stroke is often associated with underlying cardiac conditions, and greater stroke severity imposes stronger pathological stress on the heart. These two factors may interact to increase the risk of post‑stroke acute heart failure[3–5]. We therefore examined whether the effect of stroke severity on PSHF differs by cardioembolic etiology and whether PSHF mediates the effect of stroke severity on functional outcome. We hypothesized that stroke severity and cardioembolic etiology have a synergistic effect on PSHF risk and that PSHF partially mediates the impact of stroke severity on poor outcomes in cardioembolic patients[6].

# NOTATION AND ABBREVIATIONS

aOR adjusted odds ratio

ASPECTS Alberta Stroke Program Early CT Score

BNP B-type natriuretic peptide

cOR common odds ratio

CI confidence interval

eTICI expanded Treatment in Cerebral Infarction

EVT Endovascular treatment

HF Heart Failure

LVO Large vessel occlusion

mRS modified Rankin Scale

NIHSS National Institutes of Health Stroke Scale

NT-proBNP N-terminal pro-B-type natriuretic peptide

PSHF Post-stroke acute heart failure

PSM Propensity score matching

TOAST Trial of Org 10172 in Acute Stroke Treatment

# STUDY POPULATION

## INCLUSION CRITERIA

Individual patient data pooled analysis from four multicenter prospective studies: the BASILAR registry[7], the DEVT trial[8], the RESCUE-BT trial[9], and the MARVEL trial[10]. Patients meeting the following criteria were enrolled:

- LVO confirmed by digital subtraction angiography, magnetic resonance angiography, or computed tomography angiography;
- Received EVT;
- premorbid modified Rankin Scale (mRS) less than 3.

## EXCLUSION CRITERIA

- Minor stroke (NIHSS at admission <=5)
- Missing 90-day follow-up
- Absence of key baseline clinical and procedural characteristics

# MEASUREMENTS AND VARIABLES

## Outcome

Primary outcome: Very poor outcome at 90 days, defined as mRS score of 5-6[11]. Secondary outcomes: functional independence (mRS 0-2), independent ambulation (mRS 0-3), 90-day mRS distribution, and NIHSS score at 5-7 days (or discharge if earlier).

## Exposure

Post-stroke acute heart failure (PSHF) diagnosed according to the 2021 ESC guideline on heart failure and ESC Practical Guidance on Natriuretic Peptide Concentrations. Diagnostic criteria require simultaneously elevated natriuretic peptides (BNP >400 pg/mL or NT-proBNP exceeding age-specific thresholds: 450 pg/mL for age <50 years, 900 pg/mL for 50-75 years, and 1800 pg/mL for >75 years) and a new-onset or severe worsening of heart failure symptoms (at least 3 out of 6 symptoms/signs: orthopnoea, paroxysmal nocturnal dyspnoea, fatigue, pulmonary rales, peripheral oedema, and gut congestion)[12–14]. PSHF cases occurring within the first week after stroke onset or last known well were included[15,16].

## Covariates

### Pre-exposure variables:

- Age
- Sex
- Baseline NIHSS score,
- Baseline ASPECTS
- History of atrial fibrillation
- Chronic heart failure
- Premorbid mRS score
- Occlusion site
- Stroke etiology (TOAST classification)

### Post-treatment variables:

- eTICI grade
- Onset to recanalization time

# DATA MANAGEMENT

Data from one registry and three trials were combined into a harmonized dataset. The studies were reviewed to identify common variables (baseline and outcomes), and a harmonized dataset was assembled. Outcome definitions in each trial were reviewed to ensure consistency. The retained cohort was incorporated into baseline characterization, covariate adjustment, and propensity score matching analyses.

# STATISTICAL ANALYSES

## Primary analysis

Demographic factors, medical history, and baseline clinical characteristics were compared using the Chi-squared test or Fisher's exact test for categorical variables and the Mann Whitney U test for continuous variables. The clinical outcomes between groups with and without PSHF were compared using binary logistic regression, ordinal logistic regression, and linear regression, as appropriate. Adjusted odds ratio (aOR), common odds ratio (cOR), and beta coefficient were reported with 95% confidence interval (CI).

## Interaction analysis

A multiplicative term was added to the regression models test the pre-specified interaction between baseline NIHSS and cardioembolic etiology in relation to PSHF, following the methodology established by the HERMES collaboration[17].

## Mediation analysis

Mediation analysis was performed to test the pre-specified hypothesis that PSHF mediates the effect of stroke severity on poor outcomes (mRS 5-6) among patients with cardioembolic stroke. The proportion mediated was estimated using nonparametric bootstrap with 1000 simulations. We also conducted mediation analyses in non-cardioembolic patients, as an exploratory analysis to assess the specificity of the observed mediation effect.

## Propensity score matching

Propensity score matching (PSM) was applied to match subjects with a similar distribution of confounders to create novel cohorts with different status of PSHF. Based on the "MatchIt" package, PSM was performed with 1:1 matching based on the nearest neighbor matching algorithm with a caliper width of 0.2 of the propensity score. After PSM, multivariable regression analyses were applied to investigate the roles of PSHF on outcomes.

## Subgroup analysis

Heterogeneity in treatment effect size for the primary outcome was investigated within the following subgroups: age (<65 vs. >=65 years old), sex (female vs. male), baseline NIHSS score (<=16 vs. >16), ASPECTS (<6 vs. >=6), stroke causative mechanism, and time from last known well to recanalization (<=360 vs. >360 min).

## Effect of methylprednisolone

The roles of early administration of methylprednisolone after stroke onset on the incidence of PSHF among LVO patients were explored. The effect of methylprednisolone on outcomes among PSHF patients was also examined.

## Statistical software

All statistical analyses were performed using R software (version 4.4.2). Results were considered statistically significant at two-tailed P<0.05.

# STAFF LIST

Liyuan Chen, co-first author; Jiaxin Song, co-first author; Changwei Guo, co-first author; Linyu Li, co-first author; Tao Xu, co-author; Chen Gong, co-author; Liping Huang, co-author; Shuyu Jiang, co-author; Lin Gao, co-author; Xinyu Li, co-author; Gang Wu, co-author; Xue Wang, co-author; Thanh N. Nguyen, co-author; Jeffrey L. Saver, co-author; Yangmei Chen, corresponding author; Wenjie Zi, corresponding author; Chang Liu, corresponding author and principal investigator.

**Reference:**

1. Sposato LA, Hilz MJ, Aspberg S, Murthy SB, Bahit MC, Hsieh C-Y, et al. Post-Stroke Cardiovascular Complications and Neurogenic Cardiac Injury: JACC State-of-the-Art Review. J Am Coll Cardiol. 2020;76: 2768–2785. doi:10.1016/j.jacc.2020.10.009

2. Roger VL. Epidemiology of Heart Failure: A Contemporary Perspective. Circ Res. 2021;128: 1421–1434. doi:10.1161/CIRCRESAHA.121.318172

3. Arboix A, Alió J. Cardioembolic stroke: clinical features, specific cardiac disorders and prognosis. Curr Cardiol Rev. 2010;6: 150–161. doi:10.2174/157340310791658730

4. Fan X, Cao J, Li M, Zhang D, El-Battrawy I, Chen G, et al. Stroke Related Brain-Heart Crosstalk: Pathophysiology, Clinical Implications, and Underlying Mechanisms. Adv Sci (Weinh). 2024;11: e2307698. doi:10.1002/advs.202307698

5. Scheitz JF, Sposato LA, Schulz-Menger J, Nolte CH, Backs J, Endres M. Stroke-Heart Syndrome: Recent Advances and Challenges. J Am Heart Assoc. 2022;11: e026528. doi:10.1161/JAHA.122.026528

6. Sposato LA, Hilz MJ, Aspberg S, Murthy SB, Bahit MC, Hsieh C-Y, et al. Post-Stroke Cardiovascular Complications and Neurogenic Cardiac Injury: JACC State-of-the-Art Review. J Am Coll Cardiol. 2020;76: 2768–2785. doi:10.1016/j.jacc.2020.10.009

7. Writing Group for the BASILAR Group, Zi W, Qiu Z, Wu D, Li F, Liu H, et al. Assessment of Endovascular Treatment for Acute Basilar Artery Occlusion via a Nationwide Prospective Registry. JAMA Neurol. 2020;77: 561–573. doi:10.1001/jamaneurol.2020.0156

8. Zi W, Qiu Z, Li F, Sang H, Wu D, Luo W, et al. Effect of Endovascular Treatment Alone vs Intravenous Alteplase Plus Endovascular Treatment on Functional Independence in Patients With Acute Ischemic Stroke: The DEVT Randomized Clinical Trial. JAMA. 2021;325: 234–243. doi:10.1001/jama.2020.23523

9. RESCUE BT Trial Investigators, Qiu Z, Li F, Sang H, Luo W, Liu S, et al. Effect of Intravenous Tirofiban vs Placebo Before Endovascular Thrombectomy on Functional Outcomes in Large Vessel Occlusion Stroke: The RESCUE BT Randomized Clinical Trial. JAMA. 2022;328: 543–553. doi:10.1001/jama.2022.12584

10. MARVEL Trial Authors for the MARVEL Investigators, Yang Q, Guo C, Yue C, Song J, Yang J, et al. Methylprednisolone as Adjunct to Endovascular Thrombectomy for Large-Vessel Occlusion Stroke: The MARVEL Randomized Clinical Trial. JAMA. 2024;331: 840–849. doi:10.1001/jama.2024.0626

11. Yedavalli V, Salim H, Musmar B, Adeeb N, El Naamani K, Henninger N, et al. Pretreatment predictors of very poor clinical outcomes in medium vessel occlusion stroke patients treated with mechanical thrombectomy. Int J Stroke. 2024;19: 1123–1133. doi:10.1177/17474930241270524

12. Beer BN, Keshtkaran S, Kellner C, Besch L, Sundermeyer J, Dettling A, et al. Pro-adrenomedullin associates with congestion in acute heart failure patients. ESC Heart Fail. 2024;11: 3598–3606. doi:10.1002/ehf2.15007

13. McDonagh TA, Metra M, Adamo M, Gardner RS, Baumbach A, Böhm M, et al. 2021 ESC Guidelines for the diagnosis and treatment of acute and chronic heart failure. Eur Heart J. 2021;42: 3599–3726. doi:10.1093/eurheartj/ehab368

14. Mueller C, McDonald K, de Boer RA, Maisel A, Cleland JGF, Kozhuharov N, et al. Heart Failure Association of the European Society of Cardiology practical guidance on the use of natriuretic peptide concentrations. Eur J Heart Fail. 2019;21: 715–731. doi:10.1002/ejhf.1494

15. Prosser J, MacGregor L, Lees KR, Diener H-C, Hacke W, Davis S, et al. Predictors of early cardiac morbidity and mortality after ischemic stroke. Stroke. 2007;38: 2295–2302. doi:10.1161/STROKEAHA.106.471813

16. Shima S, Shinoda M, Takahashi O, Unaki A, Kimura T, Okada Y, et al. Risk Factors for Acute Heart Failure and Impact on In-Hospital Mortality after Stroke. J Stroke Cerebrovasc Dis. 2019;28: 1629–1635. doi:10.1016/j.jstrokecerebrovasdis.2019.02.030

17. Saver JL, Goyal M, van der Lugt A, Menon BK, Majoie CBLM, Dippel DW, et al. Time to Treatment With Endovascular Thrombectomy and Outcomes From Ischemic Stroke: A Meta-analysis. JAMA. 2016;316: 1279–1288. doi:10.1001/jama.2016.13647
