# Supplementary material for: Post-stroke acute heart failure in patients with large vessel occlusion undergoing endovascular treatment: A pooled analysis of individual patient data from multicenter studies with mediation analysis
Source: PLoS Med. 2026 Jul 7;23(7):e1004752. doi: 10.1371/journal.pmed.1004752 (PMC13340808; doi:10.1371/journal.pmed.1004752)
Supplement: S1 File — This compressed file contains the study protocols for the four multicenter studies included in this pooled analysis: BASILAR registry, DEVT trial, RESCUE-BT trial, and MARVEL trial. Each protocol document is provided in PDF format. (ZIP) [file pmed.1004752.s005.zip › BASILAR_no_logo.pdf]

Endovascular Treatment for Acute Basilar Artery Occlusion: a Nationwide Prospective Registry (BASILAR study)

Study Protocol

1.Background

The basilar artery is the main blood supply artery of the brainstem, occipital lobe, thalamus and part of cerebellum. Due to the degree of brain impairment, the clinical symptoms of acute basilar artery occlusion (BAO) are of great diversity. The mild degree may only have single cranial nerve palsy and slight hemiplegia and the severe cases may have quadriplegia, lock-in syndrome and even coma. Acute BAO is a rare but devastating medical conditions accounting for 1% of all ischemic strokes and 5% of large vessel occlusion strokes[1,2].

In recent years, there are several randomized clinical trials have proved the efficacy of endovascular treatment (EVT) for acute ischemic stroke with larger vessel occlusion in the anterior circulation, the benefit of EVT remains uncertain for patients with acute BAO. Since 1996, there have been four randomized control trials, (1) A multicenter, Randomized Controlled Trial of Intra-Arterial Urokinase in the Treatment of Acute Posterior Circulation Ischemic Stroke, (2) BASICS (The Basilar Artery International Cooperation Study), (3) BEST (The Acute Basilar Artery Occlusion: Endovascular Interventions vs. Standard Medical Treatment Trial) and (4) BAOCHE (Basilar Artery Occlusion: Chinese Endovascular Trial), aiming to investigate the benefit of EVT plus usual care versus usual care alone in acute BAO[3-5]. To our knowledge, the first trial has been terminated prematurely because of poor recruitment. The other trials are facing the question of whether these trials will achieve its inclusion target, mainly because some comprehensive stroke centers are unwilling to include cases after the many positive results of trials for endovascular therapy in patients with anterior circulation stroke. The Basilar Artery International Cooperation Study (BASICS) was a prospective registry which enrolled 619 patients range from Nov. 2002 to Oct. 2007, which did not demonstrate superiority of EVT over usual care (25.7% vs. 38.2%)[6]. In 2015, the

ENDOSTROKE study suggested that 42% of the acute BAO patients can achieve good functional outcomes from EVT and a recent retrospective study shown that proportion was 50% which indicated EVT is an efficacy treatment for acute BAO[7,8]. However, these studies have several limitations. First, BASICS study was conducted a decade ago and used outdated EVT techniques and devices. Second, recent studies were single arm, small sample size retrospective studies. In conclusion, these findings are unable to clarify the role of EVT for acute BAO.

We conducted this nationwide, prospective registry (BASILAR study) to investigate the efficacy and safety of EVT for acute BAO. This study had been registered on the website, Chinese Clinical Trial Registry (http://www.chictr.org.cn/index.aspx) (Registration No. ChiCTR1800014759).

2.Methods

2.1.Study Design

The BASILAR study is a nationwide, prospective registry of consecutive patients presenting with a symptomatic and radiologically confirmed acute BAO. The main purpose of the BASILAR study is to evaluate the efficacy and safety of modern EVT plus standard medical treatment (SMT) versus SMT alone in acute BAO within 24 hours of estimated occlusion time. The target number of patients included in the registry is about 800. The study was designed by the principal investigators and experts in cerebrovascular diseases and interventional neuroradiology. The BASILAR study protocol has been approved by the ethics committee of the Xinqiao hospital, Army Medical University, Chongqing, China and each participating center. The study flow chart was shown in Figure 1.

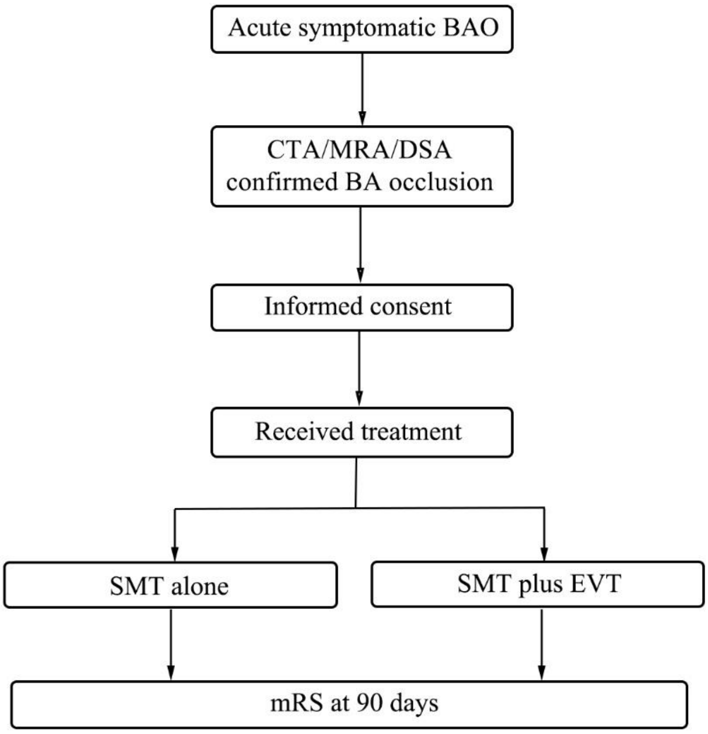

Figure 1. The BASILAR study flowchart. SMT denotes standard medical treatment, EVT endovascular treatment.

2.2.Subjects selection

2.2.1.Inclusion criteria

Patients can be enrolled in the study if they fulfilled the following criteria:

1) age ≥18 years;

2) within 24h of estimated time of BAO. Estimated time of BAO is defined as time of onset of acute symptoms consistent with the clinical diagnosis of BAO or if not known last time patient was seen normal prior to onset of these symptoms;

3) BAO confirmed by computed tomographic angiography (CTA) /magnetic resonance angiography (MRA) /digital subtraction angiography (DSA);

4) initiation of intravenous (IV) recombinant tissue plasminogen activator (rt-PA) within 4.5 hours or IV urokinase within 6 hours of estimated time of BAO;

5) initiation of EVT for patients within 24 hours of estimated time of BAO;

6) informed consent.

2.2.2.Exclusion criteria

Patients will be excluded from the study in case of:

1) pre-existing dependency with a modified Rankin Scale (mRS) ≥ 3;

2) neuroimaging evidence of cerebral hemorrhage on presentation;

3) patients without follow-up information;

4) currently in pregnant or lactating;

5) serious, advanced or terminal illness;

6) incomplete baseline critical data (e.g., imaging and time points).

2.3.Participating center eligibility

To avoid selection bias, all centers were obliged to enter all consecutive patients in the study. To be fully eligible for participation in this study, study site selection had to meet the following minimum criteria: (1) all study centers were required to have performed at least 30 endovascular procedures annually, including at least 15 thrombectomy procedures with the stent–retrieval devices; (2) all intervention teams were certified interventionists for EVT on large artery occlusion.

2.4.Treatments

Patients were divided into SMT alone group (control group) or SMT plus EVT group (EVT group) according to the treatment they received.

The control group received SMT alone, e.g. intravenous thrombolysis (IVT) with rt-PA or urokinase, antiplatelet drugs, systematic anticoagulation, or combinations of these medical treatments, as described in the guidelines for the management of AIS[9].

Patients in the EVT group underwent SMT as described previously plus EVTs which comprised mechanical thrombectomy, thromboaspiration, balloon dilation, stenting, IAT, or various combinations of these approaches. The mechanical thrombectomy may consist of thrombus retraction, aspiration or use of a stent retriever device. Generally, solitaire FR

(Medtronic Neurovascular, Irvine, CA), Trevo (Stryker neurovascular, Fremont, CA) or other newly developed devices approved by National Medical Products Administration (NMPA) were considered in the present study. Both intravenous sedation and general anesthesia can be considered to ensure the safety of patients. Re-occlusion often occurred after thrombectomy in atherosclerotic disease, therefore, rescue therapy including balloon dilation, stenting, intra-arterial thrombolysis, and glycoprotein IIb/IIIa inhibitor (GPI) might be utilized to retrieve recanalization. After recanalization of the target artery, most of the patients were transferred to the neuro-intensive care unit for at least 24 hours with their systolic blood pressure maintained at 120-140mmHg. Additionally, the patients who underwent extracranial or intracranial stent implantation were prescribed antithrombotic medication to prevent acute stent thrombosis. For the patients without prior IVT, loading doses of clopidogrel (300 mg) and aspirin (300 mg) were given, or a low dose of GPI was bolus-injected intra-arterially and maintained for at least 24 hours, while for those with prior IVT, clopidogrel (75 mg) and aspirin (100 mg) were given after 24 hours of IVT, then all the patients were given clopidogrel (75 mg/d) and aspirin (100 mg/d) for 1-3 months.

2.5.Follow-up

At 24 hours, a clinical examination including the National nstitutes of Health Stroke Scale (NIHSS) and Glasgow Coma Scale (GCS) will be carried out. At 48 hours, patients will undergo CTA or MRA imaging to evaluate the recanalization rate and CT to assess the cerebral hemorrhage. At one-week, clinical status, NIHSS score, GCS score, and adverse events will be reported. At 90 days, mRS will be recorded.

2.6.Endpoints

2.6.1.Primary efficacy outcome

The primary efficacy outcome is the score on the mRS at 90 days.

2.6.2.Secondary efficacy outcomes

The secondary clinical efficacy outcomes were the rate of favorable functional outcomes (defined as mRS ≤ 3) at 90 days, and the change of the NIHSS score at 24 hours and at 5-7 days or discharge if earlier from baseline. The technical efficacy outcomes regarding recanalization were substantial reperfusion, as assessed by means of catheter angiography in the EVT group and defined as a modified Treatment in Cerebral Infarction score of 2b (50 to 99% reperfusion) or 3 (complete reperfusion).

2.6.3.Safety outcomes

Safety outcomes were the mortality at 90 days, symptomatic intracerebral hemorrhage (sICH) at 48 hours, procedure-related complications (e.g., arterial perforation, arterial dissection, and embolization in a previously uninvolved vascular territory), and serious adverse events.

2.7.Blinding

All those involved in the subsequent clinical and imaging assessment of outcomes will be blinded to treatment allocation.

2.8.Imaging core laboratory

Centralized imaging core labs will be used in this study to provide consistent evaluation of images. The imaging core laboratory evaluated the findings on baseline non-contrast CT for the posterior circulation- Alberta Stroke Program Early CT Score (pc-ASPECTS), baseline vessel imaging (CTA, MRA, or DSA) for the location of the occlusion, angiographic outcomes on DSA imaging for technical efficacy outcomes regarding recanalization, follow-up CTA or MRA within 48 hours for vessel recanalization, and the follow-up CT for the presence of intracerebral hemorrhage. All neuroimaging studies were evaluated by two neuroradiologists independently with blindness of the treatment allocation, clinical data, and outcomes. For cases with disagreement, decisions were made by the third experienced neuroradiologist.

2.9.Clinical events committee

The clinical events committee will be comprised of three expert physicians who are independent of the investigational sites. This committee will be responsible for the review and validation of all complications that occur over the course of the study and the subsequent classification of these complications as related to the device or procedure.

Members of the clinical events committee will review all complications and adjudicate them as defined in the adverse event section in the clinical events committee manual of operations. The clinical events committee can request additional source documentation and any imaging obtained in support of the adverse event to assist with adjudication.

2.10.Statistical analyses

Statistical analysis is performed using SPSS 23 (IBM SPSS Statistics). Baseline characteristics will be summarized by means of simple descriptive statistics. The main analysis of this study consists of a comparison of the primary outcome after 90 days between two treatment groups. The primary effect parameter takes the whole range of the mRS into account and is defined as the relative risk for improvement on the mRS. It is estimated as a common odds ratio with ordinal logistic regression. In this primary analysis, multivariable regression analysis will be used to adjust for main prognostic variables between EVT and control group, such as age, baseline NIHSS, baseline pc-ASPECTS, onset to imaging diagnosis time, sex, diabetes mellitus, ischemic stroke, intravenous thrombolysis, and location of occlusion. Accordingly, treatment effect modification will be explored in subgroups defined by (tertiles of) age, sex, NIHSS score at the time of treatment, pc-ASPECTS, site of occlusion, time from onset to imaging diagnosis, and intravenous thrombolysis. Secondary effect parameters will be the improvement according to the classic dichotomizations of the mRS scale at 0-1 vs. 2-6, 0-2 vs. 3-6, and 0-3 vs. 4-6, the change of the NIHSS score at 24 hours and at 5-7 days or discharge if earlier from baseline. For the analysis of the secondary outcomes, simple 2 * 2 tables, two-group t-tests, Mann-Whitney U tests, and multivariable

linear and logistic regression models will be used, where appropriate. In all analyses, statistical uncertainty will be expressed by means of 95% CI.

We perform a 1:1 and/or 1:2 propensity score matching (PSM) analysis based on the nearest-neighbor matching algorithm with a caliper width of 0.2 of the propensity score with main prognostic variables as covariates[10]. After matching, we use property methods to test the differences between two groups. Furthermore, supportive analyses use the propensity score, compute based on an ordinal logistic regression model accounting for additional explanatory variables. Significance level is set to $P < 0{\cdot}05$, and all tests of hypotheses are two-sided.

3.Study personnel

3.1. Principal Investigator

Qingwu Yang, Raul Gomes Nogueira

3.2.Executive committee

Chengsong Yue, Jun Yang, Weidong Luo, Shuai Liu, Pengxiao Yu, Luming Chen, Dongjing Xie, Fei Gao, Jiaxing Song, Chenhao Zhao, Junjie Yuan, Guoqiang Yang, Jiacheng Huang, Hongfei Sang, Meng Zuo; Yonggang Hao, Ting Hu, Meng Liang, Feng Peng, Xiaoyun Liu, Shun Li

3.3.Steering committee

Qingwu Yang, Raul Gomes Nogueira, Wenjie Zi, Zhongming Qiu, Deping Wu, Fengli Li, Hansheng Liu, Zili Gong, Jie Shuai

3.4.Clinical events committee

Fan Deng, Dan Liu, Shengya Zeng

3.5.Imaging assessment committee

Dong Zhang, Hua Yang, Fajin Lv

3.6.Outcome assessment committee

Zhangbao Guo, Min Zhang, Jiaming Cao, Zhizhong Yan, Shuai Zhang, Zhiguo Li, Dongzhang Xue, Fanghui Bai, Hongxing Han, Xianjun Wang, Guozhong Niu, Hao Zhang, Kuihua Wang, Wei He, Tieqiao Feng, Wentong Ling, Kui Lu, Yi Zhang, Sizhi Tang, Hongliang Zeng, Jianzhou Wu, Ling Tian, Xuan Liu, Zetao Shao, Yong Liu, Shu Yang, Jun Zhang, Lin Chen, Haitao Guan, Qian Yang, Qiang Shi, Renliang Meng, Xianhui Ding, and Zili Gong.

3.7.PhD-students and study coordinators

Chengsong Yue, Jun Yang, Weidong Luo, Shuai Liu, Pengxiao Yu, Luming Chen, Dongjing Xie, Fei Gao, Jiaxing Song, Chenhao Zhao, Junjie Yuan, Guoqiang Yang, Jiacheng Huang, Hongfei Sang, Meng Zuo, Yonggang Hao, Ting Hu, Meng Liang, Feng Peng, Xiaoyun Liu, and Shun Li.

All these students' affiliation is Department of Neurology, Xinqiao Hospital and The Second Affiliated Hospital, Army Medical University (Third Military Medical University), Chongqing, China.

3.8.Study statisticians

Duolao Wang, Dong Yi

4.References

1. Smith WS, Lev MH, English JD, et al. Significance of large vessel intracranial occlusion causing acute ischemic stroke and TIA. *Stroke.* 2009;40(12):3834-3840.
2. Mattle HP, Arnold M, Lindsberg PJ, Schonewille WJ, Schroth G. Basilar artery occlusion. *The Lancet Neurology.* 2011;10(11):1002-1014.
3. Macleod MR, Davis SM, Mitchell PJ, et al. Results of a multicentre, randomised controlled trial of intra-arterial urokinase in the treatment of acute posterior circulation ischaemic stroke. *Cerebrovascular diseases (Basel, Switzerland).* 2005;20(1):12-17.

4. Liu X, Xu G, Liu Y, et al. Acute basilar artery occlusion: Endovascular Interventions versus Standard Medical Treatment (BEST) Trial-Design and protocol for a randomized, controlled, multicenter study. *Int J Stroke*. 2017;12(7):779-785.
5. van der Hoeven EJ, Schonewille WJ, Vos JA, et al. The Basilar Artery International Cooperation Study (BASICS): study protocol for a randomised controlled trial. *Trials.* 2013;14:200.
6. Schonewille WJ, Wijman CA, Michel P, et al. Treatment and outcomes of acute basilar artery occlusion in the Basilar Artery International Cooperation Study (BASICS): a prospective registry study. *The Lancet Neurology.* 2009;8(8):724-730.
7. Singer OC, Berkefeld J, Nolte CH, et al. Mechanical recanalization in basilar artery occlusion: the ENDOSTROKE study. *Annals of neurology.* 2015;77(3):415-424.
8. van Houwelingen RC, Luijckx GJ, Mazuri A, Bokkers RP, Eshghi OS, Uyttenboogaart M. Safety and Outcome of Intra-Arterial Treatment for Basilar Artery Occlusion. *JAMA neurology.* 2016;73(10):1225-1230.
9. Powers Wj Fau - Derdeyn CP, Derdeyn Cp Fau - Biller J, Biller J Fau - Coffey CS, et al. 2015 American Heart Association/American Stroke Association Focused Update of the 2013 Guidelines for the Early Management of Patients With Acute Ischemic Stroke Regarding Endovascular Treatment: A Guideline for Healthcare Professionals From the American Heart Association/American Stroke Association. *Stroke.* 2015;46(1524-4628 (Electronic)):16.
10. Kurth T, Walker AM, Glynn RJ, et al. Results of multivariable logistic regression, propensity matching, propensity adjustment, and propensity-based weighting under conditions of nonuniform effect. *Am J Epidemiol.* 2006;163(3):262-270.
