# Supplementary material for: Post-stroke acute heart failure in patients with large vessel occlusion undergoing endovascular treatment: A pooled analysis of individual patient data from multicenter studies with mediation analysis
Source: PLoS Med. 2026 Jul 7;23(7):e1004752. doi: 10.1371/journal.pmed.1004752 (PMC13340808; doi:10.1371/journal.pmed.1004752)
Supplement: S1 File — This compressed file contains the study protocols for the four multicenter studies included in this pooled analysis: BASILAR registry, DEVT trial, RESCUE-BT trial, and MARVEL trial. Each protocol document is provided in PDF format. (ZIP) [file pmed.1004752.s005.zip › DEVT_no_logo.pdf]

Effect of Endovascular Treatment Alone Versus Intravenous Alteplase Plus Endovascular Treatment on Functional Independence in Patients with Acute Ischemic Stroke: The DEVT Randomized Clinical Trial

Trial Protocol

This supplement contains the following items:

1. Original Trial Protocol (page 2 to 33)
2. Final Trial Protocol (page 34 to 65)
3. Summary of changes (page 66 to 68)

Note: personal identifying information has been redacted from the protocol documents to comply with international privacy legislation.

Sponsor

Army Medical University

No. 30 Gaotanyan Main Street, Chongqing, China

Principle Investigators

Professor Qingwu Yang, Jie Shuai

Professor Raul Gomes Nogueira

DEVT: A randomized, controlled, multicenter trial of direct endovascular treatment versus standard bridging therapy for acute stroke patients with large vessel occlusion in the anterior circulation

Protocol Version: 1.0

Issue Date: 30th March 2018

CONTENTS

List of Abbreviations ... **5**
Study Synopsis ... **7**
Schedule of Assessments ... **10**
1. BACKGROUND INFORMATION ... **11**
2. TRIAL OBJECTIVES ... **11**
3. TRIAL DESIGN ... **11**
4. PATIENT POPULATION ... **12**
  4.1. Inclusion criteria ... 12
  4.2. Exclusion criteria ... 12
5. PARTICIPATING CENTER ELIGIBILITY ... **13**
6. RANDOMIZATION ... **13**
7. TREATMENTS ... **13**
8. OUTCOMES ... **13**
  8.1. Primary Efficacy Outcome ... 13
  8.2. Secondary Efficacy Outcomes ... 14
  8.3 Safety Outcomes ... 14
9. BLINDING AND MASKING ... **14**
10. ASSESSMENT OF EFFICACY ... **15**
  10.1. The Modified Rankin Scale ... 15
  10.2. The National Institutes of Health Stroke Scale ... 15
  10.3. EQ-5D ... 15
11. ASSESSMENT OF SAFETY ... **15**
  11.1. Adverse Event Definitions ... 15
    11.1.1. Adverse Event ... 15
    11.1.2. Serious Adverse Event ... 16
  11.2. Definitions of AE-Related Terms ... 16
12. CLINICAL MANAGEMENT OF ADVERSE EVENTS ... **16**
  12.1. Identification of Adverse Events by the Investigator ... 16
  12.2. Reporting of Adverse Events ... 17
  12.3. Reporting of Serious Adverse Events ... 17
  12.4. Reporting by the Investigator ... 17
  12.5. Reporting SAEs to the Health Authorities and Ethics Committees ... 17
13. DATA SAFETY MONITORING BOARD ... **17**
14. IMAGING CORE LABORATORY ... **18**
15. CLINICAL EVENTS COMMITTEE ... **18**
16. STATISTICS ... **18**
  16.1. Sample size estimates ... 18
  16.2. Analysis Populations ... 18
    16.2.1. Intention-to-treat Population ... 18
    16.2.2. Per Protocol Population ... 18
  16.3. Analysis of Primary Efficacy Outcome ... 18
  16.4. Analysis of secondary efficacy outcomes ... 19
  16.5. Adjustment for covariates and subgroup analyses ... 19

16.6. Handling of Missing Data 19
16.7. Analyses of Safety 19
17. DIRECT ACCESS TO SOURCE DATA/DOCUMENTS 19
18. QUALITY CONTROL AND QUALITY ASSURANCE 20
18.1. Audits and Inspections 20
18.2. Protocol Amendments and Revisions 21
19. ETHICAL CONSIDERATION 21
20. DATA HANDLING AND RECORD KEEPING 21
20.1. Data Handling 21
20.2. Investigator Files/Retention of Documents 21
20.3. Source Documents and Background Data 22
20.4. Case Report Forms 22
20.5. Confidentiality 22
21. PUBLICATION AND PRESENTATION POLICY 22
22. DATA-SHARING PLAN 23
23. STUDY ORGANIZATION AND FUNDING 23
Appendix 1 – Classification of Subtype of Acute Ischemic Stroke 24
Appendix 2 - ASITN/SIR Collateral Vessel Grading System 27
Appendix 3 - The Alberta Stroke Program Early Computed Tomography Score (ASPECTS) 28
Appendix 4 - Modified Rankin Scale (MRS) 29
Appendix 5 - Modified Treatment In Cerebral Infarction (mTICI) Score 30
Investigator's Agreement 31
REFERENCES 32

List of Abbreviations

AE	Adverse Event
AIS	Acute Ischemia Stroke
ASITN/SIR	American Society of Interventional and Therapeutic Neuroradiology/Society of Interventional Radiology
ASPECTS	Alberta Stroke Program Early Computed Tomography Score
BP	Blood Pressure
CEC	Clinical Events Committee
CRF	Case Report Form
CTA	Computed Tomographic Angiography
DEVT	Direct Endovascular Treatment Versus Standard Bridging Therapy in Large Artery Anterior Circulation Stroke
DSA	Digital Subtraction Angiography
DSMB	Data Safety Monitoring Board
ECG	Electrocardiogram
EQ-5D	European Quality Five Dimensions
EVT	Endovascular Treatment
GCP	Good Clinical Practice
HbA1c	Hemoglobin A1c
HCG	Human Chorionic Gonadotropin
HR	Heart Rate
ICA	Internal Carotid Artery
ICH-GCP	International Conference on Harmonization-Good Clinical Practice
INR	International Normalized Ratio
IRB	Institutional Review Board
ITT	Intention-To-Treat
IVT	Intravenous Thrombolysis
LAR	Legally Authorized Representative
LVO	Large Vessel Occlusion
MCA	Middle Cerebral Artery
MR	Magnetic Resonance
MRA	Magnetic Resonance Angiography

mRS	Modified Rankin Scale
NCCT	Non-Contrast Computed Tomography
NIHSS	National Institutes of Health Stroke Scale
PP	Per-Protocol
QA	Quality Assurance
RCT	Randomized Controlled Trial
REB	Research Ethics Board
rt-PA	Recombinant Tissue-type Plasminogen Activator
SAE	Serious Adverse Event
SICH	Symptomatic Intracranial Hemorrhage
SOPs	Standard Operating Procedures
Temp	Temperature
TIA	Transient Ischemic Attack
TOAST	Trial of Org 10172 in Acute Stroke Treatment

111 **Study Synopsis**

**Trial Objectives**	The objective is to determine whether endovascular treatment alone is non-inferior to intravenous thrombolysis bridging endovascular treatment in acute anterior circulation large vessel occlusive patients who are eligible for intravenous rt-PA.
**Trial Design**	This study is a randomized, controlled, multicenter trial with blinded outcome assessment. This trial uses a five-look group-sequential non-inferiority design. Up to 194 patients in each interim analysis will be consecutively randomized to endovascular treatment alone or rt-PA plus endovascular treatment group in 1:1 ratio over three years from about 35 hospitals in China.
**Subjects**	<u>Inclusion criteria</u><br>1) Aged 18 years or older;<br>2) Presenting with acute ischemic stroke (AIS) symptom within 4.5 hours;<br>3) Eligible for IV rt-PA;<br>4) Occlusion of the intracranial internal carotid artery (ICA) or M1 segment of the middle cerebral artery (MCA) confirmed by CT or MR angiography (CTA or MRA);<br>5) Randomization no later than 4 hours 15 minutes after stroke symptom onset;<br>6) Informed consent obtained from patients or their legal representatives.<br><u>Exclusion criteria</u><br>1) CT or MR evidence of hemorrhage (the presence of micro-bleeds is allowed);<br>2) Contraindications of IV rt-PA;<br>3) Pre-morbidity with a modified Rankin scale (mRS) score of ≥ 2;<br>4) Currently in pregnant or lactating or serum beta human chorionic gonadotrophin (HCG) test is positive on admission;<br>5) Contraindication to radiographic contrast agents, nickel, titanium metals or their alloys;<br>6) Arterial tortuosity and/or other arterial disease that would prevent the device from reaching the target vessel;<br>7) Patients with a preexisting neurological or psychiatric disease that would confound the neurological functional evaluations;<br>8) Subjects with occlusions in multiple vascular territories (e.g. bilateral anterior circulation, or anterior/posterior circulation);<br>9) CT or MRI evidence of mass effect or intracranial tumor (except small meningioma);<br>10) CT or MRI evidence of cerebral vasculitis;<br>11) CTA or MRA evidence of intracranial arteriovenous malformations or aneurysms;<br>12) Any terminal illness with life expectancy less than 6 months;<br>13) Unlikely to be available for 90-day follow-up;<br>14) Current participation in another clinical trial.
**Treatments**	Patients are assigned to receive either endovascular treatment (EVT) alone (primary-thrombectomy group) or rt-PA plus EVT (bridging-therapy group). In the bridging-therapy group, subjects will receive a single rt-PA dose of 0.9 mg/kg IV (maximum dose: 90 mg), with 10% given as a bolus, followed by continuous IV

	infusion of the rest dose within 1 hour. Simultaneously, EVT preparation should be initiated with or as soon as IV rt-PA administration. While in the primary-thrombectomy group, subjects will receive EVT directly without prior IV rt-PA. Subjects in both groups will undergo rapid EVT. EVT consisted of mechanical thrombectomy, thromboaspiration, balloon dilation, stenting, intra-arterial thrombolysis, or various combinations of these approaches.
**Consent**	Explicit written, signed informed consent from the subject or legally authorized representative will be obtained prior to any protocol specific procedures.
**Randomization Method**	Subjects will be randomly assigned in a 1:1 fashion to receive EVT alone or IV rt-PA plus EVT. Randomization occurs immediately after baseline (at the EVT institution) CT/MR brain imaging and CT/MR angiography via a real-time, internet-based randomization method. The randomization was stratified by participating centers.
**Duration of Treatment**	This study consists of one 90-day study period for each subject.<br>Subjects will be hospitalized for care after their acute stroke according to the current standard of care. Subjects are required to return to clinic on Day 90 for end-of-study procedures.
**Laboratory Tests**	In order to support the assessment of patient safety baseline, chemistry laboratory tests will be completed. At baseline, blood work will be evaluated which includes: Blood cell counts, triglyceride, cholesterol, low density lipoprotein, high density lipoprotein, homocysteine, glucose, procalcitonin, HbA1C, prothrombin time, activated partial thromboplastin time, thrombin time, fibrinogen, D-dimer, international normalized ratio.<br>If the subject is female and is of childbearing potential, a pregnancy test (urine or serum point-of-care pregnancy test) must be completed and a negative test result obtained prior to inclusion in the trial.<br>Electrocardiograms will also be collected and reviewed at baseline.
**Assessment of Efficacy**	The primary efficacy outcome is the overall proportion of subjects experiencing a functional independence 90 days post randomization, defined as a score of 0 to 2 on the mRS.<br>The secondary efficacy outcomes include:<br>1) Proportion of mRS score 0 to 1 at 90 days;<br>2) Shift in the distribution of mRS scores at 90 days in EVT alone versus rt-PA plus EVT (ordinal shift analysis);<br>3) Successful recanalization proportion immediate after EVT. Successful recanalization is defined as a modified Treatment in Cerebral Infarction score of 2b (50 to 99% reperfusion) or 3 (complete reperfusion) in the post-procedure angiography;<br>4) Vessel recanalization rate evaluated by CTA or MRA within 48 hours;<br>5) The change of the National Institutes of Health Stroke Scale (NIHSS) score at 24 hours from baseline;<br>6) The change of the NIHSS score at 5-7 days or discharge if earlier from baseline;

	7) European Quality Five Dimensions (EQ-5D) scale score at 90 days.
**Assessment of Safety**	1) Symptomatic intracerebral hemorrhage (sICH) rate within 48 hours;<br>2) Mortality at 90 days;<br>3) Procedure-related complications such as arterial perforation, iatrogenic arterial dissection, arterial access site hematoma, and retroperitoneal hematoma;<br>4) Incidence of serious adverse events.

Schedule of Assessments

	Baseline	Day 1 (24 ± 12 h from randomization)	Day 2 (48 ± 8 h from randomization)	Day 5 or discharge (±1 d)	Day 90 (±14 d)
Informed consent	X				
History and examination	X				
Weight	X				
Vital Signs (BP, HR, Temp)	X	X	X	X	
Randomization	X				
NIHSS	X	X		X	
mRS	X*				X
ASPECTS	X				
EQ-5D					X
CBC, electrolytes, INR, aPTT, serum creatinine and serum glucose	X	X			
Pregnancy test‡	X				
NCCT/MR head	X		X**		
CTA/MRA	X		X		
ECG	X				
Endovascular Procedure	X				
sICH			X		
Mortality				X	X
AE assessment	Collected to Day 30 visit				
SAE assessment	Collected to Day 90 visit				
Prior medications	X				
Concomitant medications	Collected to Day 30 visit				

***** Historical (pre-stroke) score.

** MR head may be supplanted by an NCCT head if MR is unavailable.

‡ If the subject is female and is of childbearing potential a pregnancy test (urine or serum point-of-care pregnancy test) must be completed and the result must be negative; this is the only mandatory laboratory test prior to randomization

1. BACKGROUND INFORMATION

Intravenous thrombolysis with recombinant tissue plasminogen activator (rt-PA) within 4.5 hours of symptom onset is the first-line treatment for acute ischemic stroke(AIS)[1,2]. Several randomized controlled trials have consistently demonstrated that intravenous thrombolysis bridging with endovascular treatment (namely bridging therapy) is superior to intravenous thrombolysis alone for acute anterior large vessel occlusion(LVO)[3-9]. Intravenous thrombolysis prior to endovascular treatment can be initiated earlier, help eliminate thrombi in distal or small arteries which are inaccessible for revascularization devices, facilitate mechanical thrombectomy, and thereby increasing the rate of reperfusion[10,11]. However, intravenous thrombolysis also has some drawbacks. For instance, it may increase the risk of intracranial or systemic hemorrhage[12], especially when anti-thrombotic therapy is administrated after angioplasty and/or stenting. It may also postpone endovascular treatment and increase medical expenditures[13]. The therapeutic time window of intravenous thrombolysis is very narrow, which has largely limited its application.

It remains uncertain whether pretreated with intravenous rt-PA provides any additional benefits to the acute anterior large vessel occlusive patients experiencing endovascular treatment. A meta-analysis revealed that patients treated with bridging therapy have higher recanalization rates, fewer device passes, equal probabilities of symptomatic intracerebral hemorrhage, better clinical neurological outcomes, and lower mortality rates compared with patients treated with direct endovascular treatment[14]. Whereas, a propensity score matching analysis based on the Chinese population suggested that direct endovascular treatment can achieve similar efficacy to that of bridging therapy, and a lower proportion of asymptomatic intracranial hemorrhage[12]. However, the baseline characteristics for the direct endovascular treatment group and bridging-therapy group of these studies are lack of equipoise, which may have significant influence on the results. Prospective data on direct endovascular treatment for acute anterior large vessel occlusion remains scarce. Thus, we propose the hypothesis that EVT alone initiated within 4.5 h of stroke onset is not inferior to rt-PA plus EVT in acute stroke patients with a proximal LVO in the anterior circulation.

2. TRIAL OBJECTIVES

Direct Endovascular Treatment Versus Standard Bridging Therapy in Large Artery Anterior Circulation Stroke (DEVT) Trial aims to investigate whether EVT alone is non-inferior to rt-PA plus EVT in acute anterior circulation large vessel occlusive patients who are eligible for intravenous rt-PA.

3. TRIAL DESIGN

DEVT trial is a multicenter, prospective, randomized, open-label controlled clinical trial with blinded endpoint evaluation. It is an academic trial designed by the principal investigators and a steering committee consisting of experts in cerebrovascular diseases and interventional neuroradiology. The study patient flow outline was shown in Figure 1.

Figure 1 Study flowchart of DEVT trial.

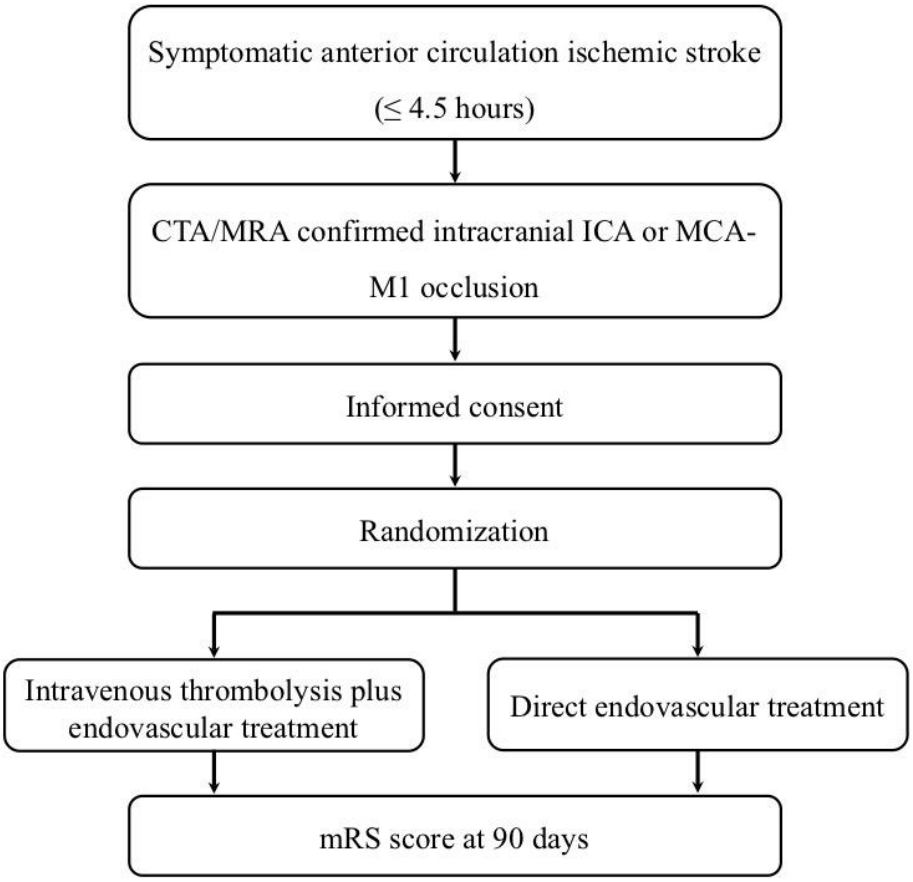

4. PATIENT POPULATION

4.1. Inclusion criteria

(1) Aged 18 years or older;
(2) Presenting with AIS symptom within 4.5 hours;
(3) Eligible for IV rt-PA;
(4) Occlusion of the intracranial internal carotid artery (ICA) or M1 segment of the middle cerebral artery (MCA) confirmed by CT or MR angiography (CTA or MRA);
(5) Randomization no later than 4 hours 15 minutes after stroke symptom onset;
(6) Informed consent obtained from patients or their legal representatives.

4.2. Exclusion criteria

(1) CT or MR evidence of hemorrhage (the presence of micro-bleeds is allowed);
(2) Contraindications of IV rt-PA;
(3) Pre-morbidity with a modified Rankin scale (mRS) score of ≥ 2;
(4) Currently in pregnant or lactating or serum beta HCG test is positive on admission;
(5) Contraindication to radiographic contrast agents, nickel, titanium metals or their alloys;
(6) Arterial tortuosity and/or other arterial disease that would prevent the device from reaching the target vessel;
(7) Patients with a preexisting neurological or psychiatric disease that would confound the neurological functional evaluations;
(8) Subjects with occlusions in multiple vascular territories (e.g. bilateral anterior circulation, or anterior/posterior circulation);
(9) CT or MRI evidence of mass effect or intracranial tumor (except small meningioma);
(10) CT or MRI evidence of cerebral vasculitis;

(11) CTA or MRA evidence of intracranial arteriovenous malformations or aneurysms;
(12) Any terminal illness with life expectancy less than 6 months;
(13) Unlikely to be available for 90-day follow-up;
(14) Current participation in another clinical trial.

5. PARTICIPATING CENTER ELIGIBILITY

To be fully eligible for participation in this trial, study centers were required have performed at least 80 endovascular procedures annually, including at least 50 thrombectomy procedures with the stent-retriever devices. Moreover, all neurointerventionists with more than five years' experience in cerebrovascular intervention and at least 10 cases of mechanical thrombectomy with stent retriever devices annually.

6. RANDOMIZATION

Subjects will be randomly assigned in a 1:1 fashion to receive EVT alone or IV rt-PA plus EVT. Randomization occurs immediately after baseline (at the EVT institution) CT/MR brain imaging and CT/MR angiography via a real-time, internet-based randomization method. The randomization was stratified by participating centers. The time of randomization is defined as the time randomization occurred on the central server and this time is considered time zero for the study. IV rt-PA will be infused immediately after randomization.
All subjects, investigators, their clinical staff, the clinical coordinating center, the data management group, and the sponsor staff and delegates will be blinded to the randomization codes. The local laboratories will also be blinded.

7. TREATMENTS

Patients are assigned to receive either EVT alone (primary-thrombectomy group) or rt-PA plus EVT (bridging-therapy group). In the bridging-therapy group, subjects will receive a single rt-PA dose of 0.9 mg/kg IV (maximum dose: 90 mg), with 10% given as a bolus, followed by continuous IV infusion of the rest dose within 1 hour. Simultaneously, EVT preparation should be initiated with or as soon as IV rt-PA administration. While in the primary-thrombectomy group, subjects will receive EVT alone without prior IV rt-PA. Subjects in both groups will undergo rapid EVT. EVT consisted of mechanical thrombectomy, thromboaspiration, balloon dilation, stenting, intra-arterial thrombolysis, or various combinations of these approaches. The choice of technique is left to the discretion of the treating neurointerventionist. Additionally, stenting of the extracranial or intracranial artery is permitted when absolutely necessary to obtain access to distal occlusion or to prevent acute re-occlusion. This may require the use of thrombolytic agents to prevent acute stent thrombosis. After recanalization of the target artery, all patients will get stroke unit care and postoperative management follows the current American Heart Association/American Stroke Association guidelines[15].
The use of conscious sedation or general anesthesia for the procedure to ensure the comfort and safety of patients is at the discretion of the individual site neurointerventionalist. The steering committee will make recommendations for dosages of thrombolytic agents, procedures, and for devices that will be considered in the trial based on proposals by the executive committee or local investigators. The requirements for a device to be considered in the trial should be approved by the China Food and Drug Administration or National Medical Products Administration.

8. OUTCOMES

8.1. Primary Efficacy Outcome

The primary end-point is the overall proportion of subjects experiencing a functional independence 90 days post randomization, defined as a score of 0 to 2 on the mRS. To ensure the reliability, evaluability, and traceability of

the mRS score, we keep patients' video or voice version of follow-up at 90 days except those who die or refuse to take a video. The primary functional outcome is centrally assessed by two independent certified neurologists in a blinded manner by the use of the video or voice recording. Disagreements are resolved by consensus.

8.2. Secondary Efficacy Outcomes

(1) Shift in the distribution of mRS scores at 90 days in EVT alone versus rt-PA plus EVT (ordinal shift analysis);

(2) Proportion of mRS score 0 to 1 at 90 days;

(3) Successful recanalization proportion immediate after EVT. Successful recanalization is defined as a modified Treatment in Cerebral Infarction score of 2b (50 to 99% reperfusion) or 3 (complete reperfusion) in the post-procedure angiography[16];

(4) Vessel recanalization rate evaluated by CTA or MRA within 48 hours;

(5) The change of the National Institutes of Health Stroke Scale (NIHSS) score at 24 hours from baseline[17];

(6) The change of the NIHSS score at 5-7 days or discharge if earlier from baseline;

(7) European Quality Five Dimensions (EQ-5D) scale score at 90 days.

8.3 Safety Outcomes

(1) Symptomatic intracerebral hemorrhage (sICH) rate within 48 hours. ICH will be evaluated according to the Heidelberg Bleeding Classification[18]. sICH was diagnosed if the new observed ICH was associated with any of the following conditions: 1) NIHSS score increased more than 4 points than that immediately before worsening; 2) NIHSS score increased more than 2 points in one category; 3) Deterioration led to intubation, hemicraniectomy, external ventricular drain placement or any other major interventions. Additionally, the symptom deteriorations could not be explained by causes other than the observed ICH. Hemicraniectomy will be defined as that surgical procedure used to decompress the swollen hemisphere;

(2) Mortality at 90 days. Mortality rates are defined as the number of deaths observed divided by the number of subject observed over the 90-day study period.

(3) Procedure-related complications such as arterial perforation, iatrogenic arterial dissection, embolization in previously uninvolved vascular territory, arterial access site hematoma, and retroperitoneal hematoma. Arterial perforation will be defined at angiography by the operator and associated with subarachnoid hemorrhage. Iatrogenic arterial dissection will be defined at angiography by the operator. Arterial access site hematoma will be assessed as a complication of arterial access puncture and defined by clinical examination and anatomic imaging. Retroperitoneal hematoma will be assessed as a complication of groin puncture and defined by imaging (ultrasound or CTA or MR). The definition of embolization in previously uninvolved vascular territory is noted after recanalization of the primary occlusion site, any vessel occlusions distal from the primary occlusion site were considered emboli due to periprocedural thrombus fragmentation.

(4) Incidence of serious adverse events.

9. BLINDING AND MASKING

Each site will designate one or more physician(s) to perform the follow-up evaluation at 24 hours, 5-7 days or discharge if earlier and at 90 days who cannot be involved in care of the subjects and must remain blinded to treatment assignment of each subject.

Regarding the NIHSS examination at baseline, 24 hours, 5-7 days or discharge if earlier and the primary end-point, first, a local independent neurologist, not involved in the trial patient management, will evaluate the scores in a face to face clinical visit, recording the examination by video with the consent of patient or the legal representative; and second, two experienced and certified physicians will centrally evaluate the score by using

the video recording. For cases with disagreement between the two assessors, decisions were made by the third experienced neurologist.

All neuroimaging end-points including baseline Alberta Stroke Program Early Computed Tomography Score (ASPECTS) score, recanalization within 48 hours, collateral circulation classification and hemorrhage will be determined by the CT/MR core laboratory, which will be also blinded to treatment allocation. Another independent angiographic core lab will review angiographic images from the procedure to determine clot location and recanalization. Serious adverse events (SAEs) and procedure-related complications will be reviewed and adjudicated by two individuals of the independent clinical events committee who will be blinded to treatment allocation.

10. ASSESSMENT OF EFFICACY

10.1. The Modified Rankin Scale

The mRS is a valid and reliable clinician-reported measure of global disability that has been widely applied for evaluating recovery from stroke. It is a scale used to measure functional recovery (the degree of disability or dependence in daily activities) of people who have suffered a stroke[19,20]. mRS scores range from 0 to 6, with 0 indicating no residual symptoms; 5 indicating bedbound, requiring constant care; and 6 indicating death. The mRS will be obtained at Day 90. Premorbid mRS status will also be obtained retrospectively at 24 Hours. The mRS will only be scored by those trained and certified in the use of this scale.

10.2. The National Institutes of Health Stroke Scale

The NIHSS is a standardized neurological examination score that is a valid and reliable measure of disability and recovery after acute stroke[17]. Scores range from 0 to 42, with higher scores indicating increasing severity. The scale includes measures of level of consciousness, extra ocular movements, motor and sensory tests, coordination, language and speech evaluations. The NIHSS will be administered at Baseline, at 24 hours from baseline, Day 5-7or discharge. The NIHSS will only be scored by those trained and certified in the use of this scale.

10.3. EQ-5D

The EQ-5D is a generic instrument for describing and valuing health. It is based on a descriptive system that defines health in terms of five dimensions: Mobility, Self-Care, Usual Activities, Pain/Discomfort, and Anxiety/Depression[21]. Each dimension has five response categories corresponding to: no problems, slight, moderate, severe and extreme problems[22]. The instrument is designed for self-completion, and respondents also rate their overall health on the.day of the interview on a 0-100 hash-marked, vertical visual analogue scale. The EQ- 5D will be administered on Day 90 by those trained in the use of this scale.

11. ASSESSMENT OF SAFETY

11.1. Adverse Event Definitions

11.1.1. Adverse Event

An adverse event (AE) is any untoward medical occurrence in a patient or clinical investigation subject administered a pharmaceutical product and which does not necessarily have to have a causal relationship with this treatment. An AE can therefore be any unfavorable and unintended sign (including an abnormal laboratory finding, for example), symptom or disease temporally associated with the use of a medicinal product, whether or not considered related to the medicinal product.

Therefore, an AE may be: A new illness; The worsening of a concomitant illness; An effect of vaccination, including the comparator; A combination of the above.

Pre-existing medical conditions are not to be reported as AEs. However, if a pre-existing condition worsens in frequency or intensity, or if in the assessment of the treating physician there is a change in its clinical

significance, this change should be reported as an AE (exacerbation). This applies equally to recurring episodes of pre-existing conditions (e.g., asthma) if the frequency or intensity increases post-randomization.

11.1.2. Serious Adverse Event

A serious adverse event (SAE) is any untoward medical occurrence that at any dose: Result in death; Are life-threatening; Require or prolong inpatient hospitalization; Result in persistent or significant disability/incapacity, or; Are a congenital/birth defect.

A SAE can also be an important medical event that may not result in death, be life-threatening, or require hospitalization, but may jeopardize the subject and may require medical or surgical intervention to prevent one of the outcomes listed in this definition. For example, any new diagnosis of cancer (made after study enrollment) is considered an important medical event. Because our primary safety outcomes for the trial are also SAEs by definition, they will be reported dually as SAEs and as outcomes. SAEs should be managed according to the best current standard of care.

All deaths occurring during the follow up to Day 90 will be reported as an SAE. When reporting a death, the event or condition that caused or contributed to the fatal outcome should be reported as a single medical concept.

AE occurring within 30 days of randomization and all SAEs will be reported in the CRF. Severity and relationship definitions are presented below.

11.2. Definitions of AE-Related Terms

**AE Severity**	
Mild	Awareness of sign or symptom but easily tolerated
Moderate	Discomfort sufficient to cause interference with normal activities.
Severe	Incapacitating, with inability to perform normal activities.
**AE Relationship**	
Related	A clinical event, including laboratory test abnormality, where there is a “reasonable possibility” that the SAE was caused by the study drug, meaning that there is evidence or arguments to suggest a causal relationship.
Probably	A clinical event, including laboratory test abnormality, with a reasonable time sequence to drug administration, unlikely to be attributed to concurrent disease or other drugs or chemicals, and which follows a clinically reasonable response on withdrawal.
Possibly	A clinical event, including laboratory test abnormality, with a reasonable time sequence to drug administration, but which could also be explained by concurrent disease or other drugs or chemicals. Information on drug withdrawal may be lacking or unclear.
Unrelated	This category is applicable to AEs which are judged to be clearly and incontrovertibly due to extraneous causes (diseases, environment, etc.) and do not meet the criteria for drug relationship listed for the above-mentioned conditions.

12. CLINICAL MANAGEMENT OF ADVERSE EVENTS

12.1. Identification of Adverse Events by the Investigator

AE monitoring and reporting will be followed-up until Day 30. SAEs will be followed through the final study exit visit (Day 90 Visit or death or end of study whichever is sooner) or until the subject is deemed “lost to follow-up”.

AE identification while the subject is admitted to the acute stroke hospital will be collected via acute stroke hospital patient records and verbal histories from the subject or legally authorized representative (LAR). For

follow up visits after discharge from the acute stroke hospital the subject (or LAR if the subject is not able to respond to the questions) will be asked about the occurrence of AEs since the last contact, and if available, from records at the acute stroke hospital. AEs that were ongoing at the last contact will be updated with a stop date or confirmed as ongoing. AE collection will continue until Day 30, and SAE to Day 90 or the final contact.

A consistent methodology of eliciting AEs at all subject evaluation timepoints will be used. Non-directive questions include: How have you felt since your last clinical visit/hospital discharge? Have you had any new or changed health problems since you were last here? Have you had any unusual or unexpected worsening of your underlying medical condition or overall health? Have there been any changes in the medicines you take since your last clinical visit/hospital discharge?

Diagnosis versus signs and symptoms for the purpose of AE reporting: if known at the time of reporting, a diagnosis should be reported rather than individual signs and symptoms. However, if a constellation of signs and/or symptoms cannot be medically characterized as a single diagnosis it is acceptable to report the information that is ultimately available.

12.2. Reporting of Adverse Events

AEs should be reported as they occur on the electronic Case Report Form (e-CRF). Documentation must be supported by an entry in the subject’s file. Each event should be described in detail along with start and stop dates, severity, relationship to investigational product as judged by the investigator, action taken and outcome.

12.3. Reporting of Serious Adverse Events

In order to comply with current regulations on SAE reporting to health authorities, the investigator must document all SAEs regardless of causal relationship and notify the Sponsor. The Investigator will give access and provide the Sponsor with all necessary information to allow the Sponsor to conduct a detailed analysis of the safety of the investigational product. It is the responsibility of the Investigator to request all necessary documentation (e.g., medical records, discharge summary, autopsy) in order to provide comprehensive safety information. All relevant information must then be transcribed into the e-SAE Form.

12.4. Reporting by the Investigator

All SAEs must be reported to the Sponsor within 24 hours of the local Investigator’s first awareness of its occurrence. SAEs will be reviewed by the trial medical monitor.

The investigator will report the SAEs using the e-SAE form in the e-CRF, which will send an immediate alert to the Sponsor. If the e-CRF system is not available, a paper SAE form should be directed within 24 hours.

12.5. Reporting SAEs to the Health Authorities and Ethics Committees

The Sponsor will inform the relevant health authorities of any reportable SAEs according to the local regulatory requirements. Reporting to the health authorities will be according to the Sponsor’s standard operating procedures.

SAEs that are assessed by the Sponsor to be unexpected and related to study drug (expedited reporting SAEs) will be reported to the regulatory agencies as per country requirements. All other SAEs will be reported to regulatory agencies based upon local reporting requirements.

The Sponsor’s medical monitor or designee will notify the investigators in writing of the occurrence of any reportable SAEs. The Sponsor or delegate will be responsible for reporting suspected unexpected serious adverse reaction to any Central Ethics Committees in compliance with local current legislation. The investigators will be responsible for informing their local ethics committees of any reportable SAEs as per their local requirements.

13. DATA SAFETY MONITORING BOARD

The independent Data safety monitoring board (DSMB) will be composed of an experienced neurologist, an interventionalist, and a biostatistician, which are not involved in the trial. The DSMB will meet at least once a year, and is provided with structured unmasked reports, prepared by the trial statistician, for their reference only.

DSMB is responsible for recommendations to the executive committee regarding stopping or extending the trial. In addition, the DSMB will review the occurrence of SAEs and make recommendations to the executive committee regarding safety of the trial.

14. IMAGING CORE LABORATORY

Centralized imaging core laboratories will be used in this trial to provide consistent assessment of all the images. CT/MR and angiographic images will be independently reviewed by two independent central imaging core laboratories respectively. CT/MR core laboratory will review CT/MR images obtained at baseline and within 24 hours for confirmation of inclusion criteria, ASPECTS score, collateral circulation classification, and presence/absence of hemorrhage. Angiographic core laboratory will review angiographic images from the procedure to determine clot location and recanalization. CT/MR core laboratory will be independent from the angiographic core laboratory to ensure the CT/MR core laboratory is blinded to the treatment allocation.

15. CLINICAL EVENTS COMMITTEE

The Clinical events committee (CEC) will be comprised of three expert physicians independent of the investigational sites. This committee will validate all the complications that occur over the course of the study and categorized for severity and relatedness according to the definition in the Adverse Event section in the CEC Manual of Operations. The CEC can request any additional source information and images supporting the adverse events to assist with the adjudication.

16. STATISTICS

16.1. Sample size estimates

According to the previous study data[12,23-25], we hypothesis that the 90-day follow-up proportion of independent functional outcome is 43% both in the primary-thrombectomy group and bridging-therapy group. The clinically relevant non-inferiority margin Δ was -10.0%. To maintain the alpha, Pocock Analog Alpha Spending Function is used. Sample size and power are computed incorporating a five-look group-sequential analysis plan with a one-sided α at 0.025, 918 cases provide 80% power for testing the primary hypothesis of this trial; assuming the attrition rate is 5% for the primary end-point, the total sample size is up to 970. The evaluable sample size is 194 at each interim analysis. Therefore, in each interim analysis, 97 cases should be enrolled in each treatment group.

16.2. Analysis Populations

16.2.1. Intention-to-treat Population

The primary efficacy analysis will be conducted in the intention-to-treat (ITT) population, defined as all subjects randomized into the trial with grouping by randomized treatment, regardless of treatment actually received. Deceased subject will be included in the ITT population with a mRS score of 6.

16.2.2. Per Protocol Population

The primary analysis will be repeated on the Per Protocol (PP) population, defined to be all subjects randomized and treated, with no major protocol deviations. This population will be determined via blinded review of protocol deviations at the end of the trial before database lock and unblinding. Prior to unblinding, the imaging from each subject at the time of inclusion will be adjudicated to determine whether they have met the criteria for endovascular intervention, and hence for the trial. This will include review of baseline NCCT and CTA. Subjects who do not meet the imaging criteria outlined in the trial inclusion/exclusion criteria, will not be included in the Per Protocol (PP) population.

Patients who withdraw informed consent immediately after randomization and are not to receive any treatment should be excluded from all analysis populations.

16.3. Analysis of Primary Efficacy Outcome

Non-inferiority test will be used to test the primary hypothesis that the proportion of patients with independent functional outcome will be non-inferior in the primary-thrombectomy group compared to the bridging-therapy group. We desired a maximum of 5 looks when approximately 20, 40, 60, 80, and 100% of the total sample size finish the follow-up, monitoring and data cleaning processes. A group-sequential test strategy was designed to have reasonable chances of stopping as early as possible, either because of efficacy or safety reasons. The independent DSMB may recommend stopping the trial either for effectiveness, or safety in case the stopping boundaries are crossed at interim analysis. For shedding cases, follow-up will be performed until the end of the study, and the results will be included in the final analysis. Statistical analysis will be performed on the SAS 9.3 system. Details of these are provided in the Statistical Analysis Plan.

16.4. Analysis of secondary efficacy outcomes

The key secondary outcomes will be tested in the following order:

1.The Proportion of mRS score 0 to 1 at 90 days;

2.Shift in the distribution of mRS scores at 90 days in EVT alone versus rt-PA plus EVT (ordinal shift analysis);

3.Successful recanalization proportion immediate after EVT.

4.Vessel recanalization rate evaluated by CTA or MRA within 48 hours;

5.The change of the National Institutes of Health Stroke Scale (NIHSS) score at 24 hours from baseline;

6.The change of the NIHSS score at 5-7 days or discharge if earlier from baseline;

7.European Quality Five Dimensions (EQ-5D) scale score at 90 days.

16.5. Adjustment for covariates and subgroup analyses

In addition to the primary and secondary analyses adjusting for age, sex, baseline NIHSS score, baseline ASPECTS score, occlusion location, exploratory analyses will be conducted to determine the potential roles of common baseline characteristics and assess potential heterogeneity of treatment effect across subgroups. Specific subgroups of interest include the age >= 70 vs. < 70 years old, male vs. female, subject with different baseline stroke severity (on NIHSS and measured radiologically on ASPECTS), baseline occlusion location (ICA occlusion: no vs. yes), cause of stroke, onset to randomization time. Full details will be specified in detail in the Statistical Analysis Plan.

16.6. Handling of Missing Data

Every effort will be made to keep missing data, particularly the Day 90 outcome assessments, to a minimum. However, some missing data may be inevitable due to, for example, loss to follow-up. Deceased subject will score 6 on the mRS and be counted as non-responders. For the primary analysis for regulatory submission, we will assume that subject missing the primary endpoint data will be considered to be non-responders. Sensitivity analyses using various imputation techniques will be specified prospectively in the Statistical Analysis Plan before the database lock for the interim analysis if more than 5% of subject randomized are missing the primary endpoint.

16.7. Analyses of Safety

The main analyses will be frequency of sICH and 90-day mortality. It is expected that the safety population and the ITT population will be near-identical. Full details will be specified in detail in the Statistical Analysis Plan.

17. DIRECT ACCESS TO SOURCE DATA/DOCUMENTS

The sponsor or delegate will be permitted to visit the study facilities at any reasonable time in order to maintain current, detailed knowledge of the study through review of the records, source documents, observation, and discussion of the conduct and progress of the study. In addition, the sponsor will maintain regular telephone and written communication with all investigators through the coordinating center. The sponsor (or delegate) will be given complete access to all components of the study facility that pertain to the conduct of this study, and may be present to observe any aspect of the conduct of the study by medical and paramedical staff, including but not

limited to drug preparations, dosing, sample collections, and clinical observations. E-CRFs will be monitored with sufficient frequency to assess the following: Subject randomization, compliance with protocol procedures, the completeness and accuracy of data entered into the e-CRFs, verification of e-CRF data against original source documents, and occurrence of AEs. Adequate time and all documents for these monitoring visits must be made available by the investigators. The investigators will permit trial-related monitoring, audits, REB/IRB review, and regulatory inspections, providing direct access to source data/documents.

18. QUALITY CONTROL AND QUALITY ASSURANCE

To ensure monitoring responsibilities are performed to the fullest extent possible, industry experienced study monitors will perform on site data verification for the trial. All data monitored on site are verified for accuracy and completeness using source documents for all subjects. In addition, 100% of subjects enrolled are monitored for the presence of signed consent.

Monitoring of the investigational sites will be conducted by the sponsor or contracted to a qualified clinical research organization. The sponsor will determine the extent, nature, and frequency of on-site visits that are needed to ensure that the study is being conducted in accordance with the approved protocol (and any amendments), Good Clinical Practice (GCP), and all applicable regulatory requirements. At site visits, the monitor will, as required, assess the progress of the study; check that the study data chosen for verification are authentic, accurate, and complete; verify that the safety and rights of patients are being protected; compare original documents with data entered into the study database; and identify any issues and address their resolution.

The investigator agrees to allow the monitor(s) direct access to all relevant documents, and to allocate his/her time and the time of staff to discuss findings, corrective actions and any relevant issues. In addition to contacts during the study, the monitor may also contact the site prior to the start of the study to discuss the protocol and data collection procedures with site personnel.

Additional on-site monitoring verification includes: ongoing evaluation of the adequacy of site facilities and staff, site recruitment, subject randomization, the presence of regulatory documents, and specific review of documents and data. The initial performance-monitoring visit to a site takes place after the initial subject(s) are enrolled and will continue according to enrolment for the duration of the trial.

During the monitoring visit, any omissions and corrections to data submitted to the database will be noted and queries will be generated by the monitor and resolved by the site.

The close-out monitoring visit by the monitor will take place at the completion of subject enrollment and protocol required follow-up visits at the performance site. At that visit, the monitor will again review the presence of a regulatory file and verify documents for currency and completion as directed by the clinical research unit. Sites will be instructed in the record retention of all trial documents. Principal Investigators are directed to close the trial and issue a final report to the institutional review board. Finally, any additional special considerations for the auditing of any additional safety issues are made during this final monitoring visit.

Except for an emergency situation in which proper care for the protection, safety and well- being of the study subjects requires medical treatment, the study will be conducted as described in the approved protocol, International Conference on Harmonization-Good Clinical Practice (ICH-GCP), Standard Operating Procedures (SOPs) and regulatory requirements. All medical treatments will be recorded. Any deviation(s) from the protocol will be recorded and presented in the final clinical study report.

18.1. Audits and Inspections

In accordance with the principles of ICH-GCP, the study site may be inspected by regulatory authorities. Quality Assurance (QA) or their designates. The investigator and relevant clinical support staff will be required to be

actively involved in audits and inspections, including staff interviews, and to make all necessary documentation and data available upon request.

During the course of the study and/or after it has been completed, one or more investigator site audits may be undertaken by auditors. The purpose of these audits is to determine whether or not the study is being/has been conducted and monitored in compliance with recognized ICH-GCP, protocol and approved amendment requirements, applicable local SOPs, and local laws and regulations. It is the responsibility of the investigator and site staff to promptly address any deficiencies stemming out of regulatory inspections and delegate audits, and to ensure that agreed-upon corrective and preventive actions are implemented as soon as possible. An inspection by any regulatory authority may occur at any time during or after completion of the study.

18.2. Protocol Amendments and Revisions

Should amendments and/or revisions to the protocol be required, they will be originated and documented by the sponsor. All amendments and/or revisions will be made in compliance with sponsor SOPs. All amendments will be submitted to the research ethics board/Institutional Review Board (REB/IRB) for approval prior to implementation. It is the sponsor's responsibility to submit all revisions and amendments to regulatory authorities when necessary.

19. ETHICAL CONSIDERATION

This research followed the ethical principles of the Helsinki Declaration. This protocol and the consent forms will be submitted to each hospital's REB/IRB. Before initiation of the study, a copy of the REB/IRBs' approval letters will be provided to the sponsor and the membership list of the REB/IRB will be kept on file. To make sure the subjects fully understand about this trial, the investigators must provide the patients or their legal representatives with detailed information about the clinical trial, including the purpose of the trial, possible benefits and risks, and the rights/obligations. Subjects have the right to withdraw from the study at any time if they wish to do so. The privacy protection of subjects has to be ensured. The patients or their legal representatives give their written informed consent prior to the study. Each patient must leave contact information to the investigator of the participating center. At the same time, the investigator must leave his own phone number to the patient so that the patient can find the investigator at any time. Ethical approval for the study was obtained by the Ethics Committee of the participating centers. SAEs will be reported to the REB/IRB according to their requirements.

20. DATA HANDLING AND RECORD KEEPING

20.1. Data Handling

During the trial, clinical data reported in the e-CRFs will be integrated into the clinical database under the responsibility of the Sponsor or their qualified representative. Quality control in the form of computerized logic and/or consistency checks will be systematically applied in order to detect errors or omissions. In addition, safety reviews may be performed several times by the Sponsor's staff in the course of the trial. Any questions pertaining to the reported clinical data will be submitted to the investigator for resolution. Each step of this process will be monitored through the implementation of individual passwords to maintain appropriate database access and to ensure database integrity.

After integration of all corrections in the complete set of data, the database will be released for statistical analysis.

20.2. Investigator Files/Retention of Documents

The investigator must maintain adequate and accurate records to enable the conduct of the study to be fully documented and the study data to be subsequently verified. These documents should be classified into two different separate categories: Investigator's Study File; and Subject Clinical Source Documents.

The Investigator's Study File will contain the Protocol/Amendments, CRFs, REB/IRB and governmental approval with correspondence, all versions of ethics approved informed consent forms, staff curriculum vitae and authorization forms and other appropriate documents/correspondence, etc.

Subject clinical source documents (usually defined by the project in advance to record efficacy/safety parameters independent of the CRFs) would include subject hospital/clinic records, physician's and nurse's notes, appointment book, original laboratory reports, ECG, image data, signed consent forms, consultant letters, and source worksheets. The investigator must keep these two categories of documents on file according to local clinical trial regulation.

The Investigator and the sponsor will maintain the records of disposition of the drug and the clinic records in accordance with ICH-GCP and each applicable regulatory agency. Clinic records will be retained at the site until informed by the sponsor to destroy the documents. If the clinical study must be terminated for any reason, the investigator will return all study materials to the sponsor and provide a written statement as to why the termination has taken place and notify the REB/IRB.

20.3. Source Documents and Background Data

Any investigators shall supply the sponsor, upon request, with any required background data from the study documentation or clinic records. This is particularly important when e-CRFs are illegible or when errors in data transcription are suspected. In case of special problems and/or governmental queries or requests for audit inspections, it is also necessary to have access to the complete study records, provided that subject confidentiality is protected.

20.4. Case Report Forms

For each subject randomized, an e-CRF must be completed and signed by the investigator. If a subject withdraws from the study, the reason must be noted on the CRF. All forms should be completed within five business days of subject visit. All corrections will be tracked in the e-CRF audit trail. The Investigator should ensure the accuracy, completeness, legibility, and timeliness of the data reported to the sponsor in the CRFs and in all required reports.

20.5. Confidentiality

All imaging, evaluation forms, reports, and other records that leave the site are identified only by the site and subject number to maintain subject confidentiality. All records are kept in a locked file cabinet. Clinical information is not released without written permission of the subject, except as necessary for monitoring by REB/IRB, health authorities, the sponsor, or the sponsor’s designee.

All study investigators at the clinical sites must ensure that the confidentiality of personal identity and all personal medical information of study subjects are maintained at all times. clinical sites must conform to local privacy and confidentiality law and custom. On the CRFs and other study documents or image materials submitted to the CRU, the subjects are identified only by study identification codes.

Personal medical information may be reviewed for the purpose of verifying data recorded in the CRF by the site monitors. Other properly authorized persons, such as the regulatory authorities, may also have access to these records. Personal medical information is always treated as confidential.

21. PUBLICATION AND PRESENTATION POLICY

A trial executive committee shall be formed, and include at least the trial principal investigator and co-principal investigator, the statistical consultant, and representatives of the Sponsor. The trial executive committee will be co-authors on all publications and presentations. The primary author list for the primary publication will consist of the executive committee and the site principal/qualified investigator at each of the sites. A formal publication policy will be presented and developed by the trial executive.

22. DATA-SHARING PLAN

The sponsor will permit any and all academic publications arising from the trial data provided that no publication containing unblinded trial data precedes publication of the overall trial results in a peer-review journal, and are (1) approved by the trial executive committee and (2) the publication authors notify the sponsor at least 30 days prior to submittal for publication with a copy of such proposed publication for the sponsor's review and comment. Employees or consultants of the sponsor will only be named as authors in any such publication if the parties agree that it is appropriate under the usual conventions used by academic institutions for naming authors in scientific publications. Upon request of the sponsor the publication or disclosure shall be delayed for up to 60 days in order to allow for the filing of a patent application. The Executive Committee will make the trial results available as free-access using PubMed and on Chinese Clinical Trials Registry. (www.chictr.org.cn).

23. STUDY ORGANIZATION AND FUNDING

DEVT trial is an investigator-initiated study which is organized by the second affiliated hospital of the Third Military Medical University and conducted in about 30 comprehensive stroke centers in China. The authors disclosed receipt of the following financial support: (1) National Science Fund for Distinguished Young Scholars (No. 81525008), and (2) Major clinical innovation technology project of the Second Affiliated Hospital of the Army Military Medical University (No. 2018JSLC0017). The funders had no involvement in the study design, data collection, analysis and interpretation, writing or decision to submit the paper.

Appendix 1 – Classification of Subtype of Acute Ischemic Stroke

The TOAST classification system includes five categories: 1) large-artery atherosclerosis, 2) cardioembolism, 3) small-artery occlusion (lacunae), 4) stroke of other determined etiology, and 5) stroke of undetermined etiology (Table 1)[26]. Diagnoses are based on clinical features and on data collected by tests such as brain imaging (CT/MRI), cardiac imaging (echocardiography, etc.), duplex imaging of extracranial arteries, arteriography, and laboratory assessments for a pro-thrombotic state.

The physician can apply the clinical and imaging findings when first assessing the patient and then consider the results of other diagnostic tests later. An important part of the classification is the ability of the physician to categorize a specific subtype diagnosis as probable or possible based on the degree of certainty. A "probable" diagnosis is made if the clinical findings, neuroimaging data, and results of diagnostic studies are consistent with one subtype and other etiologies have been excluded. A "possible" diagnosis is made when the clinical findings and neuroimaging data suggest a specific subtype but other studies are not done. Because many patients will have a limited number of diagnostic tests, the probable and possible subcategorizations allow the physician to make as precise a subgroup diagnosis as can be achieved.

Large artery atherosclerosis

These patients will have clinical and brain imaging findings of either significant (>50%) stenosis or occlusion of a major brain artery or branch cortical artery, presumably due to atherosclerosis (Table 2). Clinical findings include those of cerebral cortical impairment (aphasia, neglect, restricted motor involvement, etc.) or brain stem or cerebellar dysfunction. A history of intermittent claudication, transient ischemic attacks (TIAs) in the same vascular territory, a carotid bruit, or diminished pulses helps support the clinical diagnosis. Cortical or cerebellar lesions and brain stem or subcortical hemispheric infarcts greater than 1.5 cm in diameter on CT or MRI are considered to be of potential large-artery atherosclerotic origin. Supportive evidence by duplex imaging or arteriography of a stenosis of greater than 50% of an appropriate intracranial or extracranial artery is needed. Diagnostic studies should exclude potential sources of cardiogenic embolism. The diagnosis of stroke secondary to large artery atherosclerosis cannot be made if duplex or arteriographic studies are normal or show only minimal changes.

Cardioembolism

This category includes patients with arterial occlusions presumably due to an embolus arising in the heart (Table 2). Cardiac sources are divided into high-risk and medium-risk groups based on the evidence of their relative propensities for embolism (Table 3). At least one cardiac source for an embolus must be identified for a possible or probable diagnosis of cardioembolic stroke. Clinical and brain imaging findings are similar to those described for large-artery atherosclerosis. Evidence of a previous TIA or stroke in more than one vascular territory or systemic embolism supports a clinical diagnosis of cardiogenic stroke. Potential large-artery atherosclerotic sources of thrombosis or embolism should be eliminated. A stroke in a patient with a medium-risk cardiac source of embolism and no other cause of stroke is classified as a possible cardioembolic stroke.

Small artery occlusion (lacunae)

This category includes patients whose strokes are often labeled as lacunar infarcts in other classifications (Table 2). The patient should have one of the traditional clinical lacunar syndromes and should not have evidence of cerebral cortical dysfunction. A history of diabetes mellitus or hypertension supports the clinical diagnosis. The patient should also have a normal CT/MRI examination or a relevant brain stem or subcortical hemispheric lesion with a diameter of less than 1.5cm demonstrated. Potential cardiac sources for embolism should be absent, and evaluation of the large extracranial arteries should not demonstrate a stenosis of greater than 50% in an ipsilateral artery.

Acute stroke of other determined etiology

This category includes patients with rare causes of stroke, such as nonatherosclerotic vasculopathies, hypercoagulable states, or hematologic disorders. Patients in this group should have clinical and CT or MRI findings of an acute ischemic stroke, regardless of the size or location. Diagnostic studies such as blood tests or arteriography should reveal one of these unusual causes of stroke. Cardiac sources of embolism and large-artery atherosclerosis should be excluded by other studies.

Stroke of undetermined etiology

In several instances, the cause of a stroke cannot be determined with any degree of confidence. Some patients will have no likely etiology determined despite an extensive evaluation. In others, no cause is found but the evaluation was cursory. This category also includes patients with two or more potential causes of stroke so that the physician is unable to make a final diagnosis. For example, a patient with a medium-risk cardiac source of embolism who also has another possible cause of stroke identified would be classified as having a stroke of undetermined etiology. Other examples would be a patient who has atrial fibrillation and an ipsilateral stenosis of 50%, or the patient with a traditional lacunar syndrome and an ipsilateral carotid stenosis of 50%.

TABLE 1. TOAST Classification of Subtypes of Acute Ischemic Stroke

Large artery atherosclerosis (embolus/thrombosis)*
Cardioembolism (high-risk/medium-risk)*
Small-vessel occlusion (lacunae)*
Stroke of other determined etiology*
Stroke of undetermined etiology
a. Two or more causes identified
b. Negative evaluation
c. Incomplete evaluation

TOAST denotes Trial of Org 10172 in Acute Stroke Treatment.

*Possible or probable depending on results of ancillary studies.

Table 2. Features of TOAST Classification of Subtypes of Ischemic Stroke

	Subtype			
Features	Large artery atherosclerosis	Cardioembolism	Small artery occlusion (lacunae)	Other cause
**Clinical**				
Cortical or cerebellar dysfunction	+	+	-	+/-
Lacunar syndrome	-	-	+	+/-
**Imaging**				
Cortical, cerebellar, brain stem, or subcortical infarct > 1.5 cm	+	+	-	+/-

Subcortical or brain stem infarct < 1.5 cm	-	-	+/-	+/-
**Tests**				
Stenosis of extracranial internal carotid artery	+	-	-	-
Cardiac source of emboli	-	+	-	-
Other abnormality on tests	-	-	-	+

TABLE 3. TOAST Classification of High- and Medium-Risk Sources of Cardioembolism

**High-risk sources**
Mechanical prosthetic valve
Mitral stenosis with atrial fibrillation
Atrial fibrillation (other than lone atrial fibrillation)
Left atrial/atrial appendage thrombus
Sick sinus syndrome
Recent myocardial infarction (<4 weeks)
Left ventricular thrombus
Dilated cardiomyopathy
Akinetic left ventricular segment
Atrial myxoma
Infective endocarditis
**Medium-risk sources**
Mitral valve prolapse
Mitral annulus calcification
Mitral stenosis without atrial fibrillation
Left atrial turbulence (smoke)
Atrial septal aneurysm
Patent foramen ovale
Atrial flutter
Lone atrial fibrillation
Bioprosthetic cardiac valve
Nonbacterial thrombotic endocarditis
Congestive heart failure
Hypokinetic left ventricular segment
Myocardial infarction (> 4 weeks, < 6 months)

Appendix 2 - ASITN/SIR Collateral Vessel Grading System

Collateral vessel status was evaluated by using the American Society of Interventional and Therapeutic Neuroradiology/Society of Interventional Radiology (ASITN/SIR) collateral vessel grading system[27]. Collateral vessel scores were categorized into ASITN/SIR grades 0 or 1, 2, and 3 or 4. The following scoring system provides a guide.

Grade	Description
0	No collateral vessels visible to the ischemic site
1	Slow collateral vessels to the periphery of the ischemic site with persistence of some of the defect
2	Rapid collateral vessels to periphery of ischemic site with persistence of some of the defect and to only a portion of the ischemic territory
3	Collateral vessels with slow but complete angiographic blood flow of the ischemic bed by the late venous phase
4	Complete and rapid collateral blood flow to the vascular bed in the entire ischemic territory by retrograde perfusion

Appendix 3 - The Alberta Stroke Program Early Computed Tomography Score (ASPECTS)

NCCT shall be scored using ASPECTS, a 10-point score derived by examining each of 10 regions on the middle cerebral artery territory[28]. Ischemic change present is scored as 0; ischemic change absent is score as 1. Adding up the score gives a maximum of 10 (favorable scan) and minimum of 0 (unfavorable scan). The score is highly reliable when trichotomized into 0-4 (severe ischemic change, large core), 5-7 (moderate ischemic change) and 8-10 (minimal ischemic change, small core). ASPECTS may be less reliable early in stroke (i.e. within 90 minutes of onset); however, at later time windows it should be easy to recognize large areas of irreversible damage. Having a good quality scan and optimization of scanner is key to successful interpretation. Further information is available at: ww.aspectsinstroke.com.

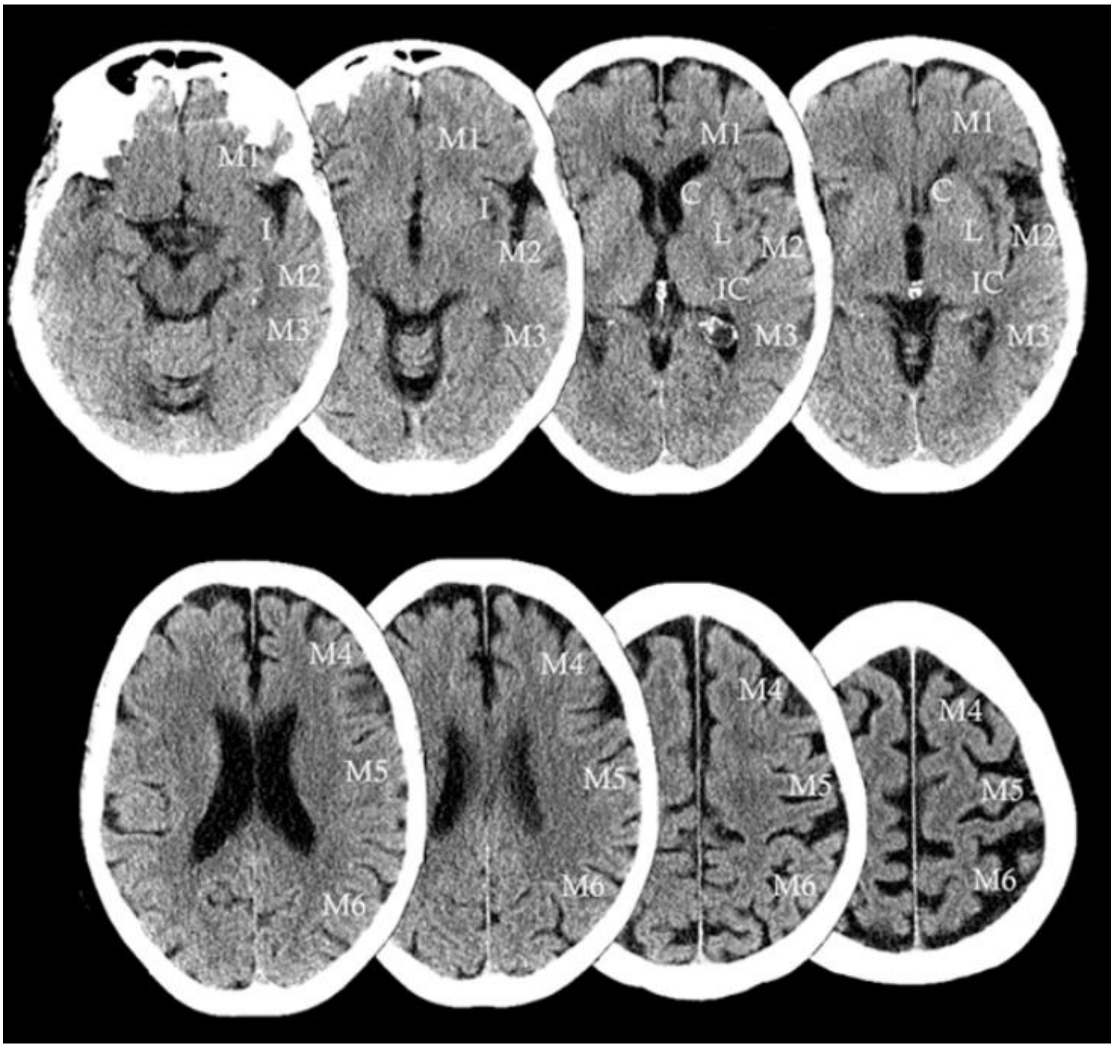

Appendix 4 - Modified Rankin Scale (MRS)

Grade	Description[29]
0	No symptoms at all
1	No significant disability despite symptoms: able to carry out all usual duties and activities
2	Slight disability: unable to carry out all previous activities but able to look after own affairs without assistance
3	Moderate disability: requiring some help, but able to walk without assistance
4	Moderately severe disability: unable to walk without assistance, and unable to attend to own bodily needs without assistance
5	Severe disability: bedridden, incontinent, and requiring constant nursing care and attention
6	Death

Appendix 5 - Modified Treatment In Cerebral Infarction (mTICI) Score

Grade	Description[16]
0	No perfusion
1	Antegrade reperfusion past the initial occlusion, but limited distal branch filling with little or slow distal reperfusion
2a	Antegrade reperfusion of less than half of the occluded target artery previously ischemic territory (e.g. in one major division of the middle cerebral artery (MCA) and its territory)
2b	Antegrade reperfusion of more than half of the previously occluded target artery ischemic territory (e.g. in two major divisions of the MCA and their territories)
3	Complete antegrade reperfusion of the previously occluded target artery ischemic territory, with absence of visualized occlusion in all distal branches

Investigator's Agreement

I have read the attached protocol: a randomized, controlled, multicenter trial of Direct Endovascular treatment Versus standard bridging Therapy for acute stroke patients with large vessel occlusion in the anterior circulation (DEVT Trial), Version 1.0 dated 30th March 2018 and agree to abide by all provisions set forth therein. I agree to comply with the current International Conference on Harmonization Guidelines for Good Clinical Practice and the laws, rules, regulations and guidelines of the community, country, state or locality relating to the conduct of the clinical study. I also agree that persons debarred from conducting or working on clinical studies by any court or regulatory agency will not be allowed to conduct or work on studies for the sponsor.

Name Site Principal Investigator Signature

Name of Clinical Site Date

REFERENCES

1. Hacke W, Kaste M, Bluhmki E, et al. Thrombolysis with alteplase 3 to 4.5 hours after acute ischemic stroke. The New England journal of medicine 2008;359:1317-29.
2. National Institute of Neurological D, Stroke rt PASSG. Tissue plasminogen activator for acute ischemic stroke. The New England journal of medicine 1995;333:1581-7.
3. Muir KW, Ford GA, Messow C-M, et al. Endovascular therapy for acute ischaemic stroke: the Pragmatic Ischaemic Stroke Thrombectomy Evaluation (PISTE) randomised, controlled trial. Journal of neurology, neurosurgery, and psychiatry 2017;88:38-44.
4. Bracard S, Ducrocq X, Mas JL, et al. Mechanical thrombectomy after intravenous alteplase versus alteplase alone after stroke (THRACE): a randomised controlled trial. The Lancet Neurology 2016;15:1138-47.
5. Saver JL, Goyal M, Bonafe A, et al. Stent-retriever thrombectomy after intravenous t-PA vs. t-PA alone in stroke. The New England journal of medicine 2015;372:2285-95.
6. Jovin TG, Chamorro A, Cobo E, et al. Thrombectomy within 8 hours after symptom onset in ischemic stroke. The New England journal of medicine 2015;372:2296-306.
7. Goyal M, Demchuk AM, Menon BK, et al. Randomized assessment of rapid endovascular treatment of ischemic stroke. The New England journal of medicine 2015;372:1019-30.
8. Campbell BC, Mitchell PJ, Kleinig TJ, et al. Endovascular therapy for ischemic stroke with perfusion-imaging selection. The New England journal of medicine 2015;372:1009-18.
9. Berkhemer OA, Fransen PS, Beumer D, et al. A randomized trial of intraarterial treatment for acute ischemic stroke. The New England journal of medicine 2015;372:11-20.
10. Chandra RV, Lesliemazwi TM, Mehta BP, et al. Does the use of IV tPA in the current era of rapid and predictable recanalization by mechanical embolectomy represent good value. Journal of neurointerventional surgery 2016;8:443-6.
11. Grotta JC, Hacke W. Stroke Neurologist's Perspective on the New Endovascular Trials. Stroke; a journal of cerebral circulation 2015;46:1447-52.
12. Wang H, Zi W, Hao Y, et al. Direct endovascular treatment: an alternative for bridging therapy in anterior circulation large-vessel occlusion stroke. European journal of neurology : the official journal of the European Federation of Neurological Societies 2017;24:935-43.
13. Rai AT, Boo S, Buseman C, et al. Intravenous thrombolysis before endovascular therapy for large vessel strokes can lead to significantly higher hospital costs without improving outcomes. Journal of neurointerventional surgery 2018;10:17-21.
14. Mistry EA, Mistry AM, Nakawah MO, et al. Mechanical Thrombectomy Outcomes With and Without Intravenous Thrombolysis in Stroke Patients: A Meta-Analysis. Stroke; a journal of cerebral circulation 2017;48:2450-6.
15. Powers WJ, Derdeyn CP, Biller J, et al. 2015 American Heart Association/American Stroke Association Focused Update of the 2013 Guidelines for the Early Management of Patients With Acute Ischemic Stroke Regarding Endovascular Treatment: A Guideline for Healthcare Professionals From the American Heart Association/American Stroke Association. Stroke; a journal of cerebral circulation 2015;46:3020-35.
16. Zaidat OO, Yoo AJ, Khatri P, et al. Recommendations on angiographic revascularization grading standards for acute ischemic stroke: a consensus statement. Stroke; a journal of cerebral circulation 2013;44:2650-63.
17. Brott T, Adams HP, Jr., Olinger CP, et al. Measurements of acute cerebral infarction: a clinical examination scale. Stroke; a journal of cerebral circulation 1989;20:864-70.
18. von Kummer R, Broderick JP, Campbell BC, et al. The Heidelberg Bleeding Classification: Classification of Bleeding Events After Ischemic Stroke and Reperfusion Therapy. Stroke; a journal of cerebral circulation

2015;46:2981-6.
19. Banks JL, Marotta CA. Outcomes validity and reliability of the modified Rankin scale: implications for stroke clinical trials: a literature review and synthesis. Stroke; a journal of cerebral circulation 2007;38:1091-6.
20. Quinn TJ, Dawson J, Walters MR, Lees KR. Reliability of the modified Rankin Scale: a systematic review. Stroke; a journal of cerebral circulation 2009;40:3393-5.
21. Brooks R. EuroQol: the current state of play. Health Policy 1996;37:53-72.
22. Herdman M, Gudex C, Lloyd A, et al. Development and preliminary testing of the new five-level version of EQ-5D (EQ-5D-5L). Quality of life research : an international journal of quality of life aspects of treatment, care and rehabilitation 2011;20:1727-36.
23. Broeg-Morvay A, Mordasini P, Bernasconi C, et al. Direct Mechanical Intervention Versus Combined Intravenous and Mechanical Intervention in Large Artery Anterior Circulation Stroke: A Matched-Pairs Analysis. Stroke; a journal of cerebral circulation 2016;47:1037-44.
24. Bellwald S, Weber R, Dobrocky T, et al. Direct Mechanical Intervention Versus Bridging Therapy in Stroke Patients Eligible for Intravenous Thrombolysis: A Pooled Analysis of 2 Registries. Stroke; a journal of cerebral circulation 2017;48:3282-8.
25. Berkhemer OA, Fransen PSS, Beumer D, et al. A randomized trial of intraarterial treatment for acute ischemic stroke. The New England journal of medicine 2015;372:11-20.
26. Adams HP, Jr., Bendixen BH, Kappelle LJ, et al. Classification of subtype of acute ischemic stroke. Definitions for use in a multicenter clinical trial. TOAST. Trial of Org 10172 in Acute Stroke Treatment. Stroke; a journal of cerebral circulation 1993;24:35-41.
27. Zaidat OO, Yoo AJ, Khatri P, et al. Recommendations on angiographic revascularization grading standards for acute ischemic stroke: a consensus statement. Stroke; a journal of cerebral circulation 2013;44:2650-63.
28. Pexman JH, Barber PA, Hill MD, et al. Use of the Alberta Stroke Program Early CT Score (ASPECTS) for assessing CT scans in patients with acute stroke. AJNR American journal of neuroradiology 2001;22:1534-42.
29. Bonita R, Beaglehole R. Recovery of motor function after stroke. Stroke; a journal of cerebral circulation 1988;19:1497-500.

Sponsor

Army Medical University

No. 30 Gaotanyan Main Street, Chongqing, China

Principle Investigators

Professor Qingwu Yang, Jie Shuai

Professor Raul Gomes Nogueira

DEVT: A randomized, controlled, multicenter trial of direct endovascular treatment versus standard bridging therapy for acute stroke patients with large vessel occlusion in the anterior circulation

Protocol Version: 2.0

Issue Date: 1st August 2019

CONTENTS

List of Abbreviations **37**
Study Synopsis **39**
Schedule of Assessments **42**
1. BACKGROUND INFORMATION **43**
2. TRIAL OBJECTIVES **43**
3. TRIAL DESIGN **43**
4. PATIENT POPULATION **44**
  4.1. Inclusion criteria 44
  4.2. Exclusion criteria 44
5. PARTICIPATING CENTER ELIGIBILITY **45**
6. RANDOMIZATION **45**
7. TREATMENTS **45**
8. OUTCOMES **45**
  8.1. Primary Efficacy Outcome 45
  8.2. Secondary Efficacy Outcomes 46
  8.3 Safety Outcomes 46
9. BLINDING AND MASKING **46**
10. ASSESSMENT OF EFFICACY **47**
  10.1. The Modified Rankin Scale 47
  10.2. The National Institutes of Health Stroke Scale 47
  10.3. EQ-5D-5L 47
11. ASSESSMENT OF SAFETY **47**
  11.1. Adverse Event Definitions 47
    11.1.1. Adverse Event 47
    11.1.2. Serious Adverse Event 48
  11.2. Definitions of AE-Related Terms 48
12. CLINICAL MANAGEMENT OF ADVERSE EVENTS **48**
  12.1. Identification of Adverse Events by the Investigator 48
  12.2. Reporting of Adverse Events 49
  12.3. Reporting of Serious Adverse Events 49
  12.4. Reporting by the Investigator 49
  12.5. Reporting SAEs to the Health Authorities and Ethics Committees 49
13. DATA SAFETY MONITORING BOARD **49**
14. IMAGING CORE LABORATORY **50**
15. CLINICAL EVENTS COMMITTEE **50**
16. STATISTICS **50**
  16.1. Sample size estimates 50
  16.2. Analysis Populations 50
    16.2.1. Intention-to-treat Population 50
    16.2.2. Per-Protocol Population 50
  16.3. Analysis of Primary Efficacy Outcome 50
  16.4. Analysis of secondary efficacy outcomes 51
  16.5. Adjustment for covariates and subgroup analyses 51

16.6. Handling of Missing Data 51
16.7. Analyses of Safety 51
16.8. SAEs 51
16.9. AEs 52
17. DIRECT ACCESS TO SOURCE DATA/DOCUMENTS 52
18. QUALITY CONTROL AND QUALITY ASSURANCE 52
18.1. Audits and Inspections 53
18.2. Protocol Amendments and Revisions 53
19. ETHICAL CONSIDERATION 53
20. DATA HANDLING AND RECORD KEEPING 53
20.1. Data Handling 54
20.2. Investigator Files/Retention of Documents 54
20.3. Source Documents and Background Data 54
20.4. Case Report Forms 54
20.5. Confidentiality 54
21. PUBLICATION AND PRESENTATION POLICY 55
22. DATA-SHARING PLAN 55
23. STUDY ORGANIZATION AND FUNDING 55
Appendix 1 - Classification of Subtype of Acute Ischemic Stroke 56
Appendix 2 - ASITN/SIR Collateral Vessel Grading System 59
Appendix 3 - The Alberta Stroke Program Early Computed Tomography Score (ASPECTS) 60
Appendix 4 - Modified Rankin Scale (MRS) 61
Appendix 5 - Expanded Thrombolysis In Cerebral Infarction (eTICI) Scale 62
Investigator's Agreement 63
REFERENCES 64

List of Abbreviations

AE	Adverse Event
AIS	Acute Ischemia Stroke
ASITN/SIR	American Society of Interventional and Therapeutic Neuroradiology/Society of Interventional Radiology
ASPECTS	Alberta Stroke Program Early Computed Tomography Score
BP	Blood Pressure
CEC	Clinical Events Committee
CRF	Case Report Form
CTA	Computed Tomographic Angiography
DEVT	Direct Endovascular Treatment Versus Standard Bridging Therapy in Large Artery Anterior Circulation Stroke
DSA	Digital Subtraction Angiography
DSMB	Data Safety Monitoring Board
ECG	Electrocardiogram
EQ-5D-5L	European Quality Five-Dimension Five-Level
EVT	Endovascular Treatment
GCP	Good Clinical Practice
HbA1c	Hemoglobin A1c
HCG	Human Chorionic Gonadotropin
HR	Heart Rate
ICA	Internal Carotid Artery
ICH-GCP	International Conference on Harmonization-Good Clinical Practice
INR	International Normalized Ratio
IRB	Institutional Review Board
ITT	Intention-To-Treat
IVT	Intravenous Thrombolysis
LAR	Legally Authorized Representative
LVO	Large Vessel Occlusion
MCA	Middle Cerebral Artery
MedDRA	Medical Dictionary for Regulatory Activities
MR	Magnetic Resonance

MRA	Magnetic Resonance Angiography
mRS	Modified Rankin Scale
NCCT	Non-Contrast Computed Tomography
NIHSS	National Institutes of Health Stroke Scale
PP	Per-Protocol
QA	Quality Assurance
RCT	Randomized Controlled Trial
REB	Research Ethics Board
rt-PA	Recombinant Tissue-type Plasminogen Activator
SAE	Serious Adverse Event
SICH	Symptomatic Intracranial Hemorrhage
SOC	System Organ Class
SOPs	Standard Operating Procedures
Temp	Temperature
TIA	Transient Ischemic Attack
TOAST	Trial of Org 10172 in Acute Stroke Treatment

898 **Study Synopsis**

**Trial Objectives**	The objective is to determine whether endovascular treatment alone is non-inferior to intravenous thrombolysis bridging endovascular treatment in acute anterior circulation large vessel occlusive patients who are eligible for intravenous rt-PA.
**Trial Design**	This study is a randomized, controlled, multicenter trial with blinded outcome assessment. This trial uses a five-look group-sequential non-inferiority design. Up to 194 patients in each interim analysis will be consecutively randomized to endovascular treatment alone or rt-PA plus endovascular treatment group in 1:1 ratio over three years from about 35 hospitals in China.
**Subjects**	<u>Inclusion criteria</u><br>1) Aged 18 years or older;<br>2) Presenting with acute ischemic stroke (AIS) symptom within 4.5 hours;<br>3) Eligible for IV rt-PA;<br>4) Occlusion of the intracranial internal carotid artery (ICA) or M1 segment of the middle cerebral artery (MCA) confirmed by CT or MR angiography (CTA or MRA);<br>5) Randomization no later than 4 hours 15 minutes after stroke symptom onset;<br>6) Informed consent obtained from patients or their legal representatives.<br><u>Exclusion criteria</u><br>1) CT or MR evidence of hemorrhage (the presence of micro-bleeds is allowed);<br>2) Contraindications of IV rt-PA;<br>3) Pre-morbidity with a modified Rankin scale (mRS) score of ≥ 2;<br>4) Currently in pregnant or lactating or serum beta human chorionic gonadotrophin (HCG) test is positive on admission;<br>5) Contraindication to radiographic contrast agents, nickel, titanium metals or their alloys;<br>6) Arterial tortuosity and/or other arterial disease that would prevent the device from reaching the target vessel;<br>7) Patients with a preexisting neurological or psychiatric disease that would confound the neurological functional evaluations;<br>8) Subjects with occlusions in multiple vascular territories (e.g. bilateral anterior circulation, or anterior/posterior circulation);<br>9) CT or MRI evidence of mass effect or intracranial tumor (except small meningioma);<br>10) CT or MRI evidence of cerebral vasculitis;<br>11) CTA or MRA evidence of intracranial arteriovenous malformations or aneurysms;<br>12) Any terminal illness with life expectancy less than 6 months;<br>13) Unlikely to be available for 90-day follow-up;<br>14) Current participation in another clinical trial.
**Treatments**	Patients are assigned to receive either endovascular treatment (EVT) alone (primary-thrombectomy group) or rt-PA plus EVT (bridging-therapy group). In the bridging-therapy group, subjects will receive a single rt-PA dose of 0.9 mg/kg IV (maximum dose: 90 mg), with 10% given as a bolus, followed by continuous IV

	infusion of the rest dose within 1 hour. Simultaneously, EVT preparation should be initiated with or as soon as IV rt-PA administration. While in the primary-thrombectomy group, subjects will receive EVT directly without prior IV rt-PA. Subjects in both groups will undergo rapid EVT. EVT consisted of mechanical thrombectomy, thromboaspiration, balloon dilation, stenting, intra-arterial thrombolysis, or various combinations of these approaches.
**Consent**	Explicit written, signed informed consent from the subject or legally authorized representative will be obtained prior to any protocol specific procedures.
**Randomization Method**	Subjects will be randomly assigned in a 1:1 fashion to receive EVT alone or rt-PA plus EVT. Randomization occurs immediately after baseline (at the EVT institution) CT/MR brain imaging and CT/MR angiography via a real-time, internet-based randomization method. The randomization was stratified by participating centers.
**Duration of Treatment**	This study consists of one 90-day study period for each subject.<br>Subjects will be hospitalized for care after their acute stroke according to the current standard of care. Subjects are required to return to clinic on Day 90 for end-of-study procedures.
**Laboratory Tests**	In order to support the assessment of patient safety baseline, chemistry laboratory tests will be completed. At baseline, blood work will be evaluated which includes: Blood cell counts, triglyceride, cholesterol, low density lipoprotein, high density lipoprotein, homocysteine, glucose, procalcitonin, HbA1C, prothrombin time, activated partial thromboplastin time, thrombin time, fibrinogen, D-dimer, international normalized ratio.<br>If the subject is female and is of childbearing potential, a pregnancy test (urine or serum point-of-care pregnancy test) must be completed and a negative test result obtained prior to inclusion in the trial.<br>Electrocardiograms will also be collected and reviewed at baseline.
**Assessment of Efficacy**	The primary efficacy outcome is the overall proportion of subjects experiencing a functional independence 90 days post randomization, defined as a score of 0 to 2 on the mRS.<br>The secondary efficacy outcomes include:<br>1) Proportion of mRS score 0 to 1 at 90 days;<br>2) Shift in the distribution of mRS scores at 90 days in EVT alone versus rt-PA plus EVT (ordinal shift analysis);<br>3) Successful recanalization proportion immediate after EVT. Successful recanalization is defined as a modified Treatment in Cerebral Infarction score of 2b (substantial perfusion),2c (near-complete perfusion) or 3 (complete reperfusion) in the post-procedure angiography;<br>4) Vessel recanalization rate evaluated by CTA or MRA within 48 hours;<br>5) The change of the National Institutes of Health Stroke Scale (NIHSS) score at 24 hours from baseline;<br>6) The change of the NIHSS score at 5-7 days or discharge if earlier from baseline;<br>7) European Quality Five-Dimension Five-Level (EQ-5D-5L) scale score at 90

	days.
**Assessment of Safety**	1) Symptomatic intracerebral hemorrhage (sICH) rate within 48 hours;<br>2) Mortality at 90 days;<br>3) Procedure-related complications such as arterial perforation, iatrogenic arterial dissection, arterial access site hematoma, and retroperitoneal hematoma;<br>4) Incidence of serious adverse events.

Schedule of Assessments

	Baseline	Day 1 (24 ± 12 h from randomization)	Day 2 (48 ± 8 h from randomization)	Day 5 or discharge (±1 d)	Day 90 (±14 d)
Informed consent	X				
History and examination	X				
Weight[★]	X				
Vital Signs (BP, HR, Temp)	X	X	X	X	
Randomization	X				
NIHSS	X	X		X	
mRS	X[*]				X
ASPECTS	X				
EQ-5D-5L					X
CBC, electrolytes, INR, aPTT, serum creatinine and serum glucose	X	X			
Pregnancy test[‡]	X				
NCCT/MR head	X		X[**]		
CTA/MRA	X		X		
ECG	X				
Endovascular Procedure	X				
sICH			X		
Mortality				X	X
AE assessment	Collected to Day 30 visit				
SAE assessment	Collected to Day 90 visit				
Prior medications[§]	X				
Concomitant medications[§]	Collected to Day 30 visit				

★ The subject's actual weight will be measured in hospital using standard hospital scales (i.e., stand up or in-bed scales if the subject is not ambulatory). If actual weight cannot be measured for any reason (due to, for example severe illness or unavailability of in-bed scales at the site), weight will be determined by first asking the subject, second asking a family member or third by estimation.

* Historical (pre-stroke) score.

** MR head may be supplanted by an NCCT head if MR is unavailable.

‡ If the subject is female and is of childbearing potential a pregnancy test (urine or serum point-of-care pregnancy test) must be completed and the result must be negative; this is the only mandatory laboratory test prior to randomization

§ Prior and concomitant medications will be listed per patient, with the listings separated within treatment group.

1. BACKGROUND INFORMATION

Intravenous thrombolysis with recombinant tissue plasminogen activator (rt-PA) within 4.5 hours of symptom onset is the first-line treatment for acute ischemic stroke(AIS)[1,2]. Several randomized controlled trials have consistently demonstrated that intravenous thrombolysis bridging with endovascular treatment (namely bridging therapy) is superior to intravenous thrombolysis alone for acute anterior large vessel occlusion(LVO)[3-9]. Intravenous thrombolysis prior to endovascular treatment can be initiated earlier, help eliminate thrombi in distal or small arteries which are inaccessible for revascularization devices, facilitate mechanical thrombectomy, and thereby increasing the rate of reperfusion[10,11]. However, intravenous thrombolysis also has some drawbacks. For instance, it may increase the risk of intracranial or systemic hemorrhage[12], especially when anti-thrombotic therapy is administrated after angioplasty and/or stenting. It may also postpone endovascular treatment and increase medical expenditures[13]. The therapeutic time window of intravenous thrombolysis is very narrow, which has largely limited its application. In addition, IVT before EVT is associated with an increased incidence of clot migration, resulting in an increased rate of clots inaccessibility by mechanical thrombectomy[14].

It remains uncertain whether pretreated with intravenous rt-PA provides any additional benefits to the acute anterior large vessel occlusive patients experiencing endovascular treatment. A meta-analysis revealed that patients treated with bridging therapy have higher recanalization rates, fewer device passes, equal probabilities of symptomatic intracerebral hemorrhage, better clinical neurological outcomes, and lower mortality rates compared with patients treated with direct endovascular treatment[15]. Whereas, a propensity score matching analysis based on the Chinese population suggested that direct endovascular treatment can achieve similar efficacy to that of bridging therapy, and a lower proportion of asymptomatic intracranial hemorrhage[12]. Another meta-analysis showed that direct endovascular treatment may carries comparable effectiveness and safety as compared with bridging therapy by pooling studies with lower selection bias[16]. However, the baseline characteristics for the direct endovascular treatment group and bridging-therapy group of these studies are lack of equipoise, which may have significant influence on the results. Prospective data on direct endovascular treatment for acute anterior large vessel occlusion remains scarce. Thus, we propose the hypothesis that EVT alone initiated within 4.5 h of stroke onset is not inferior to rt-PA plus EVT in acute stroke patients with a proximal LVO in the anterior circulation.

2. TRIAL OBJECTIVES

Direct Endovascular Treatment Versus Standard Bridging Therapy in Large Artery Anterior Circulation Stroke (DEVT) Trial aims to investigate whether EVT alone is non-inferior to rt-PA plus EVT in acute anterior circulation large vessel occlusive patients who are eligible for intravenous rt-PA.

3. TRIAL DESIGN

DEVT trial is a multicenter, prospective, randomized, open-label controlled clinical trial with blinded endpoint evaluation. It is an academic trial designed by the principal investigators and a steering committee consisting of experts in cerebrovascular diseases and interventional neuroradiology. The study patient flow outline was shown in Figure 1.

Figure 1 Study flowchart of DEVT trial.

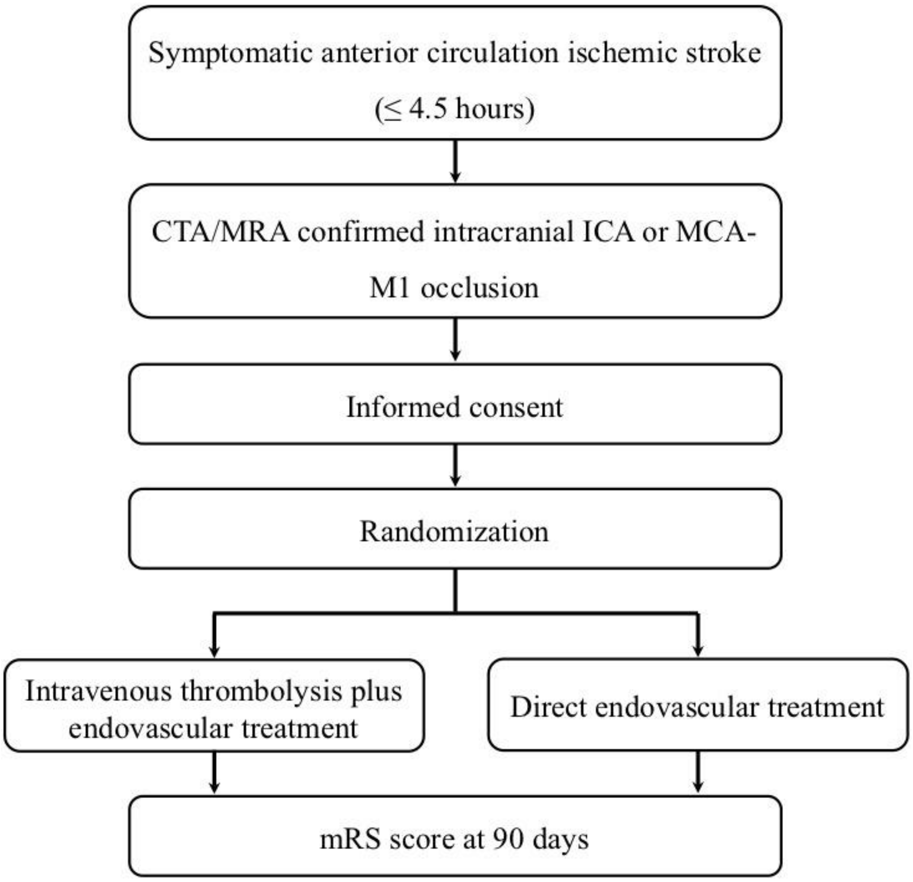

4. PATIENT POPULATION

4.1. Inclusion criteria

(1) Aged 18 years or older;
(2) Presenting with AIS symptom within 4.5 hours;
(3) Eligible for IV rt-PA;
(4) Occlusion of the intracranial internal carotid artery (ICA) or M1 segment of the middle cerebral artery (MCA) confirmed by CT or MR angiography (CTA or MRA);
(5) Randomization no later than 4 hours 15 minutes after stroke symptom onset;
(6) Informed consent obtained from patients or their legal representatives.

4.2. Exclusion criteria

(1) CT or MR evidence of hemorrhage (the presence of micro-bleeds is allowed);
(2) Contraindications of IV rt-PA;
(3) Pre-morbidity with a modified Rankin scale (mRS) score of ≥ 2;
(4) Currently in pregnant or lactating or serum beta HCG test is positive on admission;
(5) Contraindication to radiographic contrast agents, nickel, titanium metals or their alloys;
(6) Arterial tortuosity and/or other arterial disease that would prevent the device from reaching the target vessel;
(7) Patients with a preexisting neurological or psychiatric disease that would confound the neurological functional evaluations;
(8) Subjects with occlusions in multiple vascular territories (e.g. bilateral anterior circulation, or anterior/posterior circulation);
(9) CT or MRI evidence of mass effect or intracranial tumor (except small meningioma);
(10) CT or MRI evidence of cerebral vasculitis;

(11) CTA or MRA evidence of intracranial arteriovenous malformations or aneurysms;
(12) Any terminal illness with life expectancy less than 6 months;
(13) Unlikely to be available for 90-day follow-up;
(14) Current participation in another clinical trial.

5. PARTICIPATING CENTER ELIGIBILITY

To be fully eligible for participation in this trial, study centers are required have performed at least 80 endovascular procedures annually, including at least 50 thrombectomy procedures with the stent-retriever devices. Moreover, all neurointerventionists with more than five years' experience in cerebrovascular intervention and at least 10 cases of mechanical thrombectomy with stent retriever devices annually.

6. RANDOMIZATION

Subjects will be randomly assigned in a 1:1 fashion to receive EVT alone or IV rt-PA plus EVT. Randomization occurs immediately after baseline (at the EVT institution) CT/MR brain imaging and CT/MR angiography via a real-time, internet-based randomization method. The randomization was stratified by participating centers. The time of randomization is defined as the time randomization occurred on the central server and this time is considered time zero for the study. IV rt-PA will be infused immediately after randomization.
All subjects, investigators, their clinical staff, the clinical coordinating center, the data management group, and the sponsor staff and delegates will be blinded to the randomization codes. The local laboratories will also be blinded.

7. TREATMENTS

Patients are assigned to receive either EVT alone (primary-thrombectomy group) or rt-PA plus EVT (bridging-therapy group). In the bridging-therapy group, subjects will receive a single rt-PA dose of 0.9 mg/kg IV (maximum dose: 90 mg), with 10% given as a bolus, followed by continuous IV infusion of the rest dose within 1 hour. Simultaneously, EVT preparation should be initiated with or as soon as IV rt-PA administration. While in the primary-thrombectomy group, subjects will receive EVT alone without prior IV rt-PA. Subjects in both groups will undergo rapid EVT. EVT consisted of mechanical thrombectomy, thromboaspiration, balloon dilation, stenting, intra-arterial thrombolysis, or various combinations of these approaches. The choice of technique is left to the discretion of the treating neurointerventionist. Additionally, stenting of the extracranial or intracranial artery is permitted when absolutely necessary to obtain access to distal occlusion or to prevent acute re-occlusion. This may require the use of thrombolytic agents to prevent acute stent thrombosis. After recanalization of the target artery, all patients will get stroke unit care and postoperative management follows the current American Heart Association/American Stroke Association guidelines[17].
The use of conscious sedation or general anesthesia for the procedure to ensure the comfort and safety of patients is at the discretion of the individual site neurointerventionalist. The steering committee will make recommendations for dosages of thrombolytic agents, procedures, and for devices that will be considered in the trial based on proposals by the executive committee or local investigators. The requirements for a device to be considered in the trial should be approved by the China Food and Drug Administration or National Medical Products Administration.

8. OUTCOMES

8.1. Primary Efficacy Outcome

The primary end-point is the overall proportion of subjects experiencing a functional independence 90 days post randomization, defined as a score of 0 to 2 on the mRS. To ensure the reliability, evaluability, and traceability of

the mRS score, we keep patients' video or voice version of follow-up at 90 days except those who die or refuse to take a video. The primary functional outcome is centrally assessed by two independent certified neurologists in a blinded manner by the use of the video or voice recording. Disagreements are resolved by consensus.

8.2. Secondary Efficacy Outcomes

(1) Shift in the distribution of mRS scores at 90 days in EVT alone versus rt-PA plus EVT (ordinal shift analysis);

(2) Proportion of mRS score 0 to 1 at 90 days;

(3) Successful recanalization proportion immediate after EVT. Successful recanalization is defined as an expanded Thrombolysis In Cerebral Infarction score of 2b (substantial perfusion),2c (near-complete perfusion) or 3 (complete reperfusion) in the post-procedure angiography[18];

(4) Vessel recanalization rate evaluated by CTA or MRA within 48 hours;

(5) The change of the National Institutes of Health Stroke Scale (NIHSS) score at 24 hours from baseline[19];

(6) The change of the NIHSS score at 5-7 days or discharge if earlier from baseline;

(7) European Quality Five-Dimension Five-Level (EQ-5D-5L) scale score at 90 days.

8.3 Safety Outcomes

(1) Symptomatic intracerebral hemorrhage (sICH) rate within 48 hours. ICH will be evaluated according to the Heidelberg Bleeding Classification[20]. sICH was diagnosed if the new observed ICH was associated with any of the following conditions: 1) NIHSS score increased more than 4 points than that immediately before worsening; 2) NIHSS score increased more than 2 points in one category; 3) Deterioration led to intubation, hemicraniectomy, external ventricular drain placement or any other major interventions. Additionally, the symptom deteriorations could not be explained by causes other than the observed ICH. Hemicraniectomy will be defined as that surgical procedure used to decompress the swollen hemisphere;

(2) Mortality at 90 days. Mortality rates are defined as the number of deaths observed divided by the number of subject observed over the 90-day study period;

(3) Procedure-related complications such as arterial perforation, iatrogenic arterial dissection, embolization in previously uninvolved vascular territory, arterial access site hematoma, and retroperitoneal hematoma. Arterial perforation will be defined at angiography by the operator and associated with subarachnoid hemorrhage. Iatrogenic arterial dissection will be defined at angiography by the operator. Arterial access site hematoma will be assessed as a complication of arterial access puncture and defined by clinical examination and anatomic imaging. Retroperitoneal hematoma will be assessed as a complication of groin puncture and defined by imaging (ultrasound or CTA or MR). The definition of embolization in previously uninvolved vascular territory is noted after recanalization of the primary occlusion site, any vessel occlusions distal from the primary occlusion site are considered emboli due to periprocedural thrombus fragmentation.

(4) Incidence of serious adverse events.

9. BLINDING AND MASKING

Each site will designate one or more physician(s) to perform the follow-up evaluation at 24 hours, 5-7 days or discharge if earlier and at 90 days who cannot be involved in care of the subjects and must remain blinded to treatment assignment of each subject.

Regarding the NIHSS examination at baseline, 24 hours, 5-7 days or discharge if earlier and the primary end-point, first, a local independent neurologist, not involved in the trial patient management, will evaluate the scores in a face to face clinical visit, recording the examination by video with the consent of patient or the legal representative; and second, two experienced and certified physicians will centrally evaluate the score by using

the video recording. For cases with disagreement between the two assessors, decisions are made by the third experienced neurologist.

All neuroimaging end-points including baseline Alberta Stroke Program Early Computed Tomography Score (ASPECTS) score, recanalization within 48 hours, collateral circulation classification and hemorrhage will be determined by the CT/MR core laboratory, which will be also blinded to treatment allocation. Another independent angiographic core lab will review angiographic images from the procedure to determine clot location and recanalization. Serious adverse events (SAEs) and procedure-related complications will be reviewed and adjudicated by two individuals of the independent clinical events committee who will be blinded to treatment allocation.

10. ASSESSMENT OF EFFICACY

10.1. The Modified Rankin Scale

The mRS is a valid and reliable clinician-reported measure of global disability that has been widely applied for evaluating recovery from stroke. It is a scale used to measure functional recovery (the degree of disability or dependence in daily activities) of people who have suffered a stroke[21,22]. mRS scores range from 0 to 6, with 0 indicating no residual symptoms; 5 indicating bedbound, requiring constant care; and 6 indicating death. The mRS will be obtained at Day 90. Premorbid mRS status will also be obtained retrospectively and reported on the 24h CRF page. The mRS will only be scored by those trained and certified in the use of this scale.

10.2. The National Institutes of Health Stroke Scale

The NIHSS is a standardized neurological examination score that is a valid and reliable measure of disability and recovery after acute stroke[19]. Scores range from 0 to 42, with higher scores indicating increasing severity. The scale includes measures of level of consciousness, extra ocular movements, motor and sensory tests, coordination, language and speech evaluations. The NIHSS will be administered at Baseline, at 24 hours from baseline, Day 5-7or discharge. The NIHSS will only be scored by those trained and certified in the use of this scale.

10.3. EQ-5D-5L

The EQ-5D-5L is a generic instrument for describing and valuing health. It is based on a descriptive system that defines health in terms of five dimensions: Mobility, Self-Care, Usual Activities, Pain/Discomfort, and Anxiety/Depression[23]. Each dimension has five response categories corresponding to: no problems, slight, moderate, severe and extreme problems. The instrument is designed for self-completion, and respondents also rate their overall health on the day of the interview on a 0-100 hash-marked, vertical visual analogue scale. The EQ-5D-5L will be administered on Day 90 by those trained in the use of this scale.

11. ASSESSMENT OF SAFETY

11.1. Adverse Event Definitions

11.1.1. Adverse Event

An adverse event (AE) is any untoward medical occurrence in a patient or clinical investigation subject administered a pharmaceutical product and which does not necessarily have to have a causal relationship with this treatment. An AE can therefore be any unfavorable and unintended sign (including an abnormal laboratory finding, for example), symptom or disease temporally associated with the use of a medicinal product, whether or not considered related to the medicinal product.

Therefore, an AE may be: A new illness; The worsening of a concomitant illness; An effect of vaccination, including the comparator; A combination of the above.

Pre-existing medical conditions are not to be reported as AEs. However, if a pre-existing condition worsens in frequency or intensity, or if in the assessment of the treating physician there is a change in its clinical

significance, this change should be reported as an AE (exacerbation). This applies equally to recurring episodes of pre-existing conditions (e.g., asthma) if the frequency or intensity increases post-randomization.

11.1.2. Serious Adverse Event

A serious adverse event (SAE) is any untoward medical occurrence that at any dose: Result in death; Are life-threatening; Require or prolong inpatient hospitalization; Result in persistent or significant disability/incapacity, or; Are a congenital/birth defect.

A SAE can also be an important medical event that may not result in death, be life-threatening, or require hospitalization, but may jeopardize the subject and may require medical or surgical intervention to prevent one of the outcomes listed in this definition. For example, any new diagnosis of cancer (made after study enrollment) is considered an important medical event. Because our primary safety outcomes for the trial are also SAEs by definition, they will be reported dually as SAEs and as outcomes. SAEs should be managed according to the best current standard of care.

All deaths occurring during the follow up to Day 90 will be reported as an SAE. When reporting a death, the event or condition that caused or contributed to the fatal outcome should be reported as a single medical concept.

AE occurring within 30 days of randomization and all SAEs will be reported in the CRF. Severity and relationship definitions are presented below.

11.2. Definitions of AE-Related Terms

**AE Severity**	
Mild	Awareness of sign or symptom but easily tolerated
Moderate	Discomfort sufficient to cause interference with normal activities.
Severe	Incapacitating, with inability to perform normal activities.
**AE Relationship**	
Related	A clinical event, including laboratory test abnormality, where there is a “reasonable possibility” that the SAE was caused by the study drug, meaning that there is evidence or arguments to suggest a causal relationship.
Probably	A clinical event, including laboratory test abnormality, with a reasonable time sequence to drug administration, unlikely to be attributed to concurrent disease or other drugs or chemicals, and which follows a clinically reasonable response on withdrawal.
Possibly	A clinical event, including laboratory test abnormality, with a reasonable time sequence to drug administration, but which could also be explained by concurrent disease or other drugs or chemicals. Information on drug withdrawal may be lacking or unclear.
Unrelated	This category is applicable to AEs which are judged to be clearly and incontrovertibly due to extraneous causes (diseases, environment, etc.) and do not meet the criteria for drug relationship listed for the above-mentioned conditions.

12. CLINICAL MANAGEMENT OF ADVERSE EVENTS

12.1. Identification of Adverse Events by the Investigator

AE monitoring and reporting will be followed-up until Day 30. SAEs will be followed through the final study exit visit (Day 90 Visit or death or end of study whichever is sooner) or until the subject is deemed “lost to follow-up”.

AE identification while the subject is admitted to the acute stroke hospital will be collected via acute stroke hospital patient records and verbal histories from the subject or legally authorized representative (LAR). For

follow up visits after discharge from the acute stroke hospital the subject (or LAR if the subject is not able to respond to the questions) will be asked about the occurrence of AEs since the last contact, and if available, from records at the acute stroke hospital. AEs that were ongoing at the last contact will be updated with a stop date or confirmed as ongoing. AE collection will continue until Day 30, and SAE to Day 90 or the final contact.

A consistent methodology of eliciting AEs at all subject evaluation timepoints will be used. Non-directive questions include: How have you felt since your last clinical visit/hospital discharge? Have you had any new or changed health problems since you were last here? Have you had any unusual or unexpected worsening of your underlying medical condition or overall health? Have there been any changes in the medicines you take since your last clinical visit/hospital discharge?

Diagnosis versus signs and symptoms for the purpose of AE reporting: if known at the time of reporting, a diagnosis should be reported rather than individual signs and symptoms. However, if a constellation of signs and/or symptoms cannot be medically characterized as a single diagnosis it is acceptable to report the information that is ultimately available.

12.2. Reporting of Adverse Events

AEs should be reported as they occur on the electronic Case Report Form (e-CRF). Documentation must be supported by an entry in the subject’s file. Each event should be described in detail along with start and stop dates, severity, relationship to investigational product as judged by the investigator, action taken and outcome.

12.3. Reporting of Serious Adverse Events

In order to comply with current regulations on SAE reporting to health authorities, the investigator must document all SAEs regardless of causal relationship and notify the Sponsor. The Investigator will give access and provide the Sponsor with all necessary information to allow the Sponsor to conduct a detailed analysis of the safety of the investigational product. It is the responsibility of the Investigator to request all necessary documentation (e.g., medical records, discharge summary, autopsy) in order to provide comprehensive safety information. All relevant information must then be transcribed into the e-SAE Form.

12.4. Reporting by the Investigator

All SAEs must be reported to the Sponsor within 24 hours of the local Investigator’s first awareness of its occurrence. SAEs will be reviewed by the trial medical monitor.

The investigator will report the SAEs using the e-SAE form in the e-CRF, which will send an immediate alert to the Sponsor. If the e-CRF system is not available, a paper SAE form should be directed within 24 hours.

12.5. Reporting SAEs to the Health Authorities and Ethics Committees

The Sponsor will inform the relevant health authorities of any reportable SAEs according to the local regulatory requirements. Reporting to the health authorities will be according to the Sponsor’s standard operating procedures.

SAEs that are assessed by the Sponsor to be unexpected and related to study drug (expedited reporting SAEs) will be reported to the regulatory agencies as per country requirements. All other SAEs will be reported to regulatory agencies based upon local reporting requirements.

The Sponsor’s medical monitor or designee will notify the investigators in writing of the occurrence of any reportable SAEs. The Sponsor or delegate will be responsible for reporting suspected unexpected serious adverse reaction to any Central Ethics Committees in compliance with local current legislation. The investigators will be responsible for informing their local ethics committees of any reportable SAEs as per their local requirements.

13. DATA SAFETY MONITORING BOARD

The independent Data safety monitoring board (DSMB) will be composed of an experienced neurologist, an interventionalist, and a biostatistician, which are not involved in the trial. The DSMB will meet at least once a year, and is provided with structured unmasked reports, prepared by the trial statistician, for their reference only.

DSMB is responsible for recommendations to the executive committee regarding stopping or extending the trial. In addition, the DSMB will review the occurrence of SAEs and make recommendations to the executive committee regarding safety of the trial.

14. IMAGING CORE LABORATORY

Centralized imaging core laboratories will be used in this trial to provide consistent assessment of all the images. CT/MR and angiographic images will be independently reviewed by two independent central imaging core laboratories respectively. CT/MR core laboratory will review CT/MR images obtained at baseline and within 24 hours for confirmation of inclusion criteria, ASPECTS score, collateral circulation classification, and presence/absence of hemorrhage. Angiographic core laboratory will review angiographic images from the procedure to determine clot location and recanalization. CT/MR core laboratory will be independent from the angiographic core laboratory to ensure the CT/MR core laboratory is blinded to the treatment allocation.

15. CLINICAL EVENTS COMMITTEE

The Clinical events committee (CEC) will be comprised of three expert physicians independent of the investigational sites. This committee will validate all the complications that occur over the course of the study and categorized for severity and relatedness according to the definition in the Adverse Event section in the CEC Manual of Operations. The CEC can request any additional source information and images supporting the adverse events to assist with the adjudication.

16. STATISTICS

16.1. Sample size estimates

According to the previous study data[12,24-26], we hypothesis that the 90-day follow-up proportion of independent functional outcome is 43% both in the primary-thrombectomy group and bridging-therapy group. The clinically relevant non-inferiority margin Δ was -10.0%. To maintain the alpha, Pocock Analog Alpha Spending Function is used. Sample size and power are computed incorporating a five-look group-sequential analysis plan with a one-sided α at 0.025, 918 cases provide 80% power for testing the primary hypothesis of this trial; assuming the attrition rate is 5% for the primary end-point, the total sample size is up to 970. The evaluable sample size is 194 at each interim analysis. Therefore, in each interim analysis, 97 cases should be enrolled in each treatment group.

16.2. Analysis Populations

16.2.1. Intention-to-treat Population

The primary efficacy analysis will be conducted in the intention-to-treat (ITT) population, defined as all subjects randomized into the trial with grouping by randomized treatment, regardless of treatment actually received. Deceased subject will be included in the ITT population with a mRS score of 6.

16.2.2. Per-Protocol Population

The primary analysis will be repeated on the Per-Protocol (PP) population, defined to be all subjects randomized and treated, with no major protocol deviations. This population will be determined via blinded review of protocol deviations at the end of the trial before database lock and unblinding. Prior to unblinding, the imaging from each subject at the time of inclusion will be adjudicated to determine whether they have met the criteria for endovascular intervention, and hence for the trial. This will include review of baseline NCCT and CTA. Subjects who do not meet the imaging criteria outlined in the trial inclusion/exclusion criteria, will not be included in the PP population.

Patients who withdraw informed consent immediately after randomization and are not to receive any treatment should be excluded from all analysis populations.

16.3. Analysis of Primary Efficacy Outcome

Non-inferiority test will be used to test the primary hypothesis that the proportion of patients with independent functional outcome will be non-inferior in the primary-thrombectomy group compared to the bridging-therapy group. We desired a maximum of 5 looks when approximately 20, 40, 60, 80, and 100% of the total sample size finish the follow-up, monitoring and data cleaning processes. A group-sequential test strategy was designed to have reasonable chances of stopping as early as possible, either because of efficacy or safety reasons. The independent DSMB may recommend stopping the trial either for effectiveness, or safety in case the stopping boundaries are crossed at interim analysis. For shedding cases, follow-up will be performed until the end of the study, and the results will be included in the final analysis. Statistical analysis will be performed on the SAS 9.3 system. Details of these are provided in the Statistical Analysis Plan.

16.4. Analysis of secondary efficacy outcomes

The key secondary outcomes will be tested in the following order:

1.The Proportion of mRS score 0 to 1 at 90 days;

2.Shift in the distribution of mRS scores at 90 days in EVT alone versus rt-PA plus EVT (ordinal shift analysis);

3.Successful recanalization proportion immediate after EVT.

4.Vessel recanalization rate evaluated by CTA or MRA within 48 hours;

5.The change of the National Institutes of Health Stroke Scale (NIHSS) score at 24 hours from baseline;

6.The change of the NIHSS score at 5-7 days or discharge if earlier from baseline;

7.European Quality Five-Dimension Five-Level (EQ-5D-5L) scale score at 90 days.

16.5. Adjustment for covariates and subgroup analyses

In addition to the primary and secondary analyses adjusting for age, sex, baseline NIHSS score, baseline ASPECTS score, occlusion location, exploratory analyses will be conducted to determine the potential roles of common baseline characteristics and assess potential heterogeneity of treatment effect across subgroups. Specific subgroups of interest include the age >= 70 vs. < 70 years old, male vs. female, subject with different baseline stroke severity (on NIHSS and measured radiologically on ASPECTS), baseline occlusion location (ICA occlusion: no vs. yes), cause of stroke, onset to randomization time. Full details will be specified in detail in the Statistical Analysis Plan.

16.6. Handling of Missing Data

Every effort will be made to keep missing data, particularly the Day 90 outcome assessments, to a minimum. However, some missing data may be inevitable due to, for example, loss to follow-up. Deceased subject will score 6 on the mRS and be counted as non-responders. For the primary analysis for regulatory submission, we will assume that subject missing the primary endpoint data will be considered to be non-responders. Sensitivity analyses using various imputation techniques will be specified prospectively in the Statistical Analysis Plan before the database lock for the interim analysis if more than 5% of subject randomized are missing the primary endpoint.

16.7. Analyses of Safety

The main analyses will be frequency of sICH and 90-day mortality. It is expected that the safety population and the ITT population will be near-identical. Full details will be specified in detail in the Statistical Analysis Plan.

16.8. SAEs

SAEs over the 90-day study period will be summarized by presenting, for each treatment group, the number and percentage of subjects having at least one SAE, having an SAE in each body system and preferred term, by severity and relatedness to study medication. The frequencies and incidences of SAEs occurring in subjects in the active and control groups will be summarized within treatment group by the Medical Dictionary for Regulatory Activities (MedDRA) System Organ Class (SOC). The frequencies and incidences of SAEs and discontinuations due to SAEs occurring in subjects in the active and control groups will be summarized within treatment group.

16.9. AEs

Additional analyses will consider the frequency of AEs and discontinuations due to AEs. AEs will be summarized by presenting, for each treatment group, the number and percentage of subjects having any AE, having an AE in each body system and preferred term. Severity and relatedness to study medication will be recorded. The frequencies and incidences of AEs occurring in subjects in the active and control groups will be summarized within treatment group by the Medical Dictionary for Regulatory Activities (MedDRA) System Organ Class (SOC).

17. DIRECT ACCESS TO SOURCE DATA/DOCUMENTS

The sponsor or delegate will be permitted to visit the study facilities at any reasonable time in order to maintain current, detailed knowledge of the study through review of the records, source documents, observation, and discussion of the conduct and progress of the study. In addition, the sponsor will maintain regular telephone and written communication with all investigators through the coordinating center. The sponsor (or delegate) will be given complete access to all components of the study facility that pertain to the conduct of this study, and may be present to observe any aspect of the conduct of the study by medical and paramedical staff, including but not limited to drug preparations, dosing, sample collections, and clinical observations. E-CRFs will be monitored with sufficient frequency to assess the following: Subject randomization, compliance with protocol procedures, the completeness and accuracy of data entered into the e-CRFs, verification of e-CRF data against original source documents, and occurrence of AEs. Adequate time and all documents for these monitoring visits must be made available by the investigators. The investigators will permit trial-related monitoring, audits, REB/IRB review, and regulatory inspections, providing direct access to source data/documents.

18. QUALITY CONTROL AND QUALITY ASSURANCE

To ensure monitoring responsibilities are performed to the fullest extent possible, industry experienced study monitors will perform on site data verification for the trial. All data monitored on site are verified for accuracy and completeness using source documents for all subjects. In addition, 100% of subjects enrolled are monitored for the presence of signed consent.

Monitoring of the investigational sites will be conducted by the sponsor or contracted to a qualified clinical research organization. The sponsor will determine the extent, nature, and frequency of on-site visits that are needed to ensure that the study is being conducted in accordance with the approved protocol (and any amendments), Good Clinical Practice (GCP), and all applicable regulatory requirements. At site visits, the monitor will, as required, assess the progress of the study; check that the study data chosen for verification are authentic, accurate, and complete; verify that the safety and rights of patients are being protected; compare original documents with data entered into the study database; and identify any issues and address their resolution.

The investigator agrees to allow the monitor(s) direct access to all relevant documents, and to allocate his/her time and the time of staff to discuss findings, corrective actions and any relevant issues. In addition to contacts during the study, the monitor may also contact the site prior to the start of the study to discuss the protocol and data collection procedures with site personnel.

Additional on-site monitoring verification includes: ongoing evaluation of the adequacy of site facilities and staff, site recruitment, subject randomization, the presence of regulatory documents, and specific review of documents and data. The initial performance-monitoring visit to a site takes place after the initial subject(s) are enrolled and will continue according to enrolment for the duration of the trial.

During the monitoring visit, any omissions and corrections to data submitted to the database will be noted and queries will be generated by the monitor and resolved by the site.

The close-out monitoring visit by the monitor will take place at the completion of subject enrollment and protocol required follow-up visits at the performance site. At that visit, the monitor will again review the presence of a regulatory file and verify documents for currency and completion as directed by the clinical research unit. Sites will be instructed in the record retention of all trial documents. Principal Investigators are directed to close the trial and issue a final report to the institutional review board. Finally, any additional special considerations for the auditing of any additional safety issues are made during this final monitoring visit.

Except for an emergency situation in which proper care for the protection, safety and well- being of the study subjects requires medical treatment, the study will be conducted as described in the approved protocol, International Conference on Harmonization-Good Clinical Practice (ICH-GCP), Standard Operating Procedures (SOPs) and regulatory requirements. All medical treatments will be recorded. Any deviation(s) from the protocol will be recorded and presented in the final clinical study report.

18.1. Audits and Inspections

In accordance with the principles of ICH-GCP, the study site may be inspected by regulatory authorities. Quality Assurance (QA) or their designates. The investigator and relevant clinical support staff will be required to be actively involved in audits and inspections, including staff interviews, and to make all necessary documentation and data available upon request.

During the course of the study and/or after it has been completed, one or more investigator site audits may be undertaken by auditors. The purpose of these audits is to determine whether or not the study is being/has been conducted and monitored in compliance with recognized ICH-GCP, protocol and approved amendment requirements, applicable local SOPs, and local laws and regulations. It is the responsibility of the investigator and site staff to promptly address any deficiencies stemming out of regulatory inspections and delegate audits, and to ensure that agreed-upon corrective and preventive actions are implemented as soon as possible.

An inspection by any regulatory authority may occur at any time during or after completion of the study.

18.2. Protocol Amendments and Revisions

Should amendments and/or revisions to the protocol be required, they will be originated and documented by the sponsor. All amendments and/or revisions will be made in compliance with sponsor SOPs. All amendments will be submitted to the research ethics board/Institutional Review Board (REB/IRB) for approval prior to implementation. It is the sponsor’s responsibility to submit all revisions and amendments to regulatory authorities when necessary.

19. ETHICAL CONSIDERATION

This research followed the ethical principles of the Helsinki Declaration. This protocol and the consent forms will be submitted to each hospital’s REB/IRB. Before initiation of the study, a copy of the REB/IRBs’ approval letters will be provided to the sponsor and the membership list of the REB/IRB will be kept on file. To make sure the subjects fully understand about this trial, the investigators must provide the patients or their legal representatives with detailed information about the clinical trial, including the purpose of the trial, possible benefits and risks, and the rights/obligations. Subjects have the right to withdraw from the study at any time if they wish to do so. The privacy protection of subjects has to be ensured. The patients or their legal representatives give their written informed consent prior to the study. Each patient must leave contact information to the investigator of the participating center. At the same time, the investigator must leave his own phone number to the patient so that the patient can find the investigator at any time. Ethical approval for the study was obtained by the Ethics Committee of the participating centers. SAEs will be reported to the REB/IRB according to their requirements.

20. DATA HANDLING AND RECORD KEEPING

20.1. Data Handling

During the trial, clinical data reported in the e-CRFs will be integrated into the clinical database under the responsibility of the Sponsor or their qualified representative. Quality control in the form of computerized logic and/or consistency checks will be systematically applied in order to detect errors or omissions. In addition, safety reviews may be performed several times by the Sponsor’s staff in the course of the trial. Any questions pertaining to the reported clinical data will be submitted to the investigator for resolution. Each step of this process will be monitored through the implementation of individual passwords to maintain appropriate database access and to ensure database integrity.

After integration of all corrections in the complete set of data, the database will be released for statistical analysis.

20.2. Investigator Files/Retention of Documents

The investigator must maintain adequate and accurate records to enable the conduct of the study to be fully documented and the study data to be subsequently verified. These documents should be classified into two different separate categories: Investigator's Study File; and Subject Clinical Source Documents.

The Investigator's Study File will contain the Protocol/Amendments, CRFs, REB/IRB and governmental approval with correspondence, all versions of ethics approved informed consent forms, staff curriculum vitae and authorization forms and other appropriate documents/correspondence, etc.

Subject clinical source documents (usually defined by the project in advance to record efficacy/safety parameters independent of the CRFs) would include subject hospital/clinic records, physician's and nurse's notes, appointment book, original laboratory reports, ECG, image data, signed consent forms, consultant letters, and source worksheets. The investigator must keep these two categories of documents on file according to local clinical trial regulation.

The Investigator and the sponsor will maintain the records of disposition of the drug and the clinic records in accordance with ICH-GCP and each applicable regulatory agency. Clinic records will be retained at the site until informed by the sponsor to destroy the documents. If the clinical study must be terminated for any reason, the investigator will return all study materials to the sponsor and provide a written statement as to why the termination has taken place and notify the REB/IRB.

20.3. Source Documents and Background Data

Any investigators shall supply the sponsor, upon request, with any required background data from the study documentation or clinic records. This is particularly important when e-CRFs are illegible or when errors in data transcription are suspected. In case of special problems and/or governmental queries or requests for audit inspections, it is also necessary to have access to the complete study records, provided that subject confidentiality is protected.

20.4. Case Report Forms

For each subject randomized, an e-CRF must be completed and signed by the investigator. If a subject withdraws from the study, the reason must be noted on the CRF. All forms should be completed within five business days of subject visit. All corrections will be tracked in the e-CRF audit trail. The Investigator should ensure the accuracy, completeness, legibility, and timeliness of the data reported to the sponsor in the CRFs and in all required reports.

20.5. Confidentiality

All imaging, evaluation forms, reports, and other records that leave the site are identified only by the site and subject number to maintain subject confidentiality. All records are kept in a locked file cabinet. Clinical information is not released without written permission of the subject, except as necessary for monitoring by REB/IRB, health authorities, the sponsor, or the sponsor’s designee.

All study investigators at the clinical sites must ensure that the confidentiality of personal identity and all personal medical information of study subjects are maintained at all times. clinical sites must conform to local privacy and confidentiality law and custom. On the CRFs and other study documents or image materials submitted to the CRU, the subjects are identified only by study identification codes.

Personal medical information may be reviewed for the purpose of verifying data recorded in the CRF by the site monitors. Other properly authorized persons, such as the regulatory authorities, may also have access to these records. Personal medical information is always treated as confidential.

21. PUBLICATION AND PRESENTATION POLICY

A trial executive committee shall be formed, and include at least the trial principal investigator and co-principal investigator, the statistical consultant, and representatives of the Sponsor. The trial executive committee will be co-authors on all publications and presentations. The primary author list for the primary publication will consist of the executive committee and the site principal/qualified investigator at each of the sites. A formal publication policy will be presented and developed by the trial executive.

22. DATA-SHARING PLAN

The sponsor will permit any and all academic publications arising from the trial data provided that no publication containing unblinded trial data precedes publication of the overall trial results in a peer-review journal, and are (1) approved by the trial executive committee and (2) the publication authors notify the sponsor at least 30 days prior to submittal for publication with a copy of such proposed publication for the sponsor’s review and comment. Employees or consultants of the sponsor will only be named as authors in any such publication if the parties agree that it is appropriate under the usual conventions used by academic institutions for naming authors in scientific publications. Upon request of the sponsor the publication or disclosure shall be delayed for up to 60 days in order to allow for the filing of a patent application. The Executive Committee will make the trial results available as free-access using PubMed and on Chinese Clinical Trials Registry. (www.chictr.org.cn).

23. STUDY ORGANIZATION AND FUNDING

DEVT trial is an investigator-initiated study which is organized by the second affiliated hospital of the Third Military Medical University and conducted in about 30 comprehensive stroke centers in China. The authors disclosed receipt of the following financial support: (1) National Natural Science Foundation of China (Nos. 81525008, 81901236, 81801157), (2) Chongqing Major Disease Prevention and Control Technology Research Project (No. 2019ZX001), (3) Major clinical innovation technology project of the Second Affiliated Hospital of the Army Military Medical University (No. 2018JSLC0017), and (4) Clinical Medical Research Talent Training Program of Army Medical University (2019XLC2008, 2019XLC3016). The funders had no involvement in the study design, data collection, analysis and interpretation, writing or decision to submit the paper.

Appendix 1 - Classification of Subtype of Acute Ischemic Stroke

The TOAST classification system includes five categories: 1) large-artery atherosclerosis, 2) cardioembolism, 3) small-artery occlusion (lacunae), 4) stroke of other determined etiology, and 5) stroke of undetermined etiology (Table 1)[27]. Diagnoses are based on clinical features and on data collected by tests such as brain imaging (CT/MRI), cardiac imaging (echocardiography, etc.), duplex imaging of extracranial arteries, arteriography, and laboratory assessments for a pro-thrombotic state.

The physician can apply the clinical and imaging findings when first assessing the patient and then consider the results of other diagnostic tests later. An important part of the classification is the ability of the physician to categorize a specific subtype diagnosis as probable or possible based on the degree of certainty. A "probable" diagnosis is made if the clinical findings, neuroimaging data, and results of diagnostic studies are consistent with one subtype and other etiologies have been excluded. A "possible" diagnosis is made when the clinical findings and neuroimaging data suggest a specific subtype but other studies are not done. Because many patients will have a limited number of diagnostic tests, the probable and possible subcategorizations allow the physician to make as precise a subgroup diagnosis as can be achieved.

Large artery atherosclerosis

These patients will have clinical and brain imaging findings of either significant (>50%) stenosis or occlusion of a major brain artery or branch cortical artery, presumably due to atherosclerosis (Table 2). Clinical findings include those of cerebral cortical impairment (aphasia, neglect, restricted motor involvement, etc.) or brain stem or cerebellar dysfunction. A history of intermittent claudication, transient ischemic attacks (TIAs) in the same vascular territory, a carotid bruit, or diminished pulses helps support the clinical diagnosis. Cortical or cerebellar lesions and brain stem or subcortical hemispheric infarcts greater than 1.5 cm in diameter on CT or MRI are considered to be of potential large-artery atherosclerotic origin. Supportive evidence by duplex imaging or arteriography of a stenosis of greater than 50% of an appropriate intracranial or extracranial artery is needed. Diagnostic studies should exclude potential sources of cardiogenic embolism. The diagnosis of stroke secondary to large artery atherosclerosis cannot be made if duplex or arteriographic studies are normal or show only minimal changes.

Cardioembolism

This category includes patients with arterial occlusions presumably due to an embolus arising in the heart (Table 2). Cardiac sources are divided into high-risk and medium-risk groups based on the evidence of their relative propensities for embolism (Table 3). At least one cardiac source for an embolus must be identified for a possible or probable diagnosis of cardioembolic stroke. Clinical and brain imaging findings are similar to those described for large-artery atherosclerosis. Evidence of a previous TIA or stroke in more than one vascular territory or systemic embolism supports a clinical diagnosis of cardiogenic stroke. Potential large-artery atherosclerotic sources of thrombosis or embolism should be eliminated. A stroke in a patient with a medium-risk cardiac source of embolism and no other cause of stroke is classified as a possible cardioembolic stroke.

Small artery occlusion (lacunae)

This category includes patients whose strokes are often labeled as lacunar infarcts in other classifications (Table 2). The patient should have one of the traditional clinical lacunar syndromes and should not have evidence of cerebral cortical dysfunction. A history of diabetes mellitus or hypertension supports the clinical diagnosis. The patient should also have a normal CT/MRI examination or a relevant brain stem or subcortical hemispheric lesion with a diameter of less than 1.5cm demonstrated. Potential cardiac sources for embolism should be absent, and evaluation of the large extracranial arteries should not demonstrate a stenosis of greater than 50% in an ipsilateral artery.

Acute stroke of other determined etiology

This category includes patients with rare causes of stroke, such as nonatherosclerotic vasculopathies, hypercoagulable states, or hematologic disorders. Patients in this group should have clinical and CT or MRI findings of an acute ischemic stroke, regardless of the size or location. Diagnostic studies such as blood tests or arteriography should reveal one of these unusual causes of stroke. Cardiac sources of embolism and large-artery atherosclerosis should be excluded by other studies.

Stroke of undetermined etiology

In several instances, the cause of a stroke cannot be determined with any degree of confidence. Some patients will have no likely etiology determined despite an extensive evaluation. In others, no cause is found but the evaluation was cursory. This category also includes patients with two or more potential causes of stroke so that the physician is unable to make a final diagnosis. For example, a patient with a medium-risk cardiac source of embolism who also has another possible cause of stroke identified would be classified as having a stroke of undetermined etiology. Other examples would be a patient who has atrial fibrillation and an ipsilateral stenosis of 50%, or the patient with a traditional lacunar syndrome and an ipsilateral carotid stenosis of 50%.

Table 1. TOAST Classification of Subtypes of Acute Ischemic Stroke

Large artery atherosclerosis (embolus/thrombosis)*
Cardioembolism (high-risk/medium-risk)*
Small-vessel occlusion (lacunae)*
Stroke of other determined etiology*
Stroke of undetermined etiology
a. Two or more causes identified
b. Negative evaluation
c. Incomplete evaluation

TOAST denotes Trial of Org 10172 in Acute Stroke Treatment.

*Possible or probable depending on results of ancillary studies.

Table 2. Features of TOAST Classification of Subtypes of Ischemic Stroke

	Subtype			
Features	Large artery atherosclerosis	Cardioembolism	Small artery occlusion (lacunae)	Other cause
**Clinical**				
Cortical or cerebellar dysfunction	+	+	-	+/-
Lacunar syndrome	-	-	+	+/-
**Imaging**				
Cortical, cerebellar, brain stem, or subcortical infarct > 1.5 cm	+	+	-	+/-

Subcortical or brain stem infarct < 1.5 cm	-	-	+/-	+/-
**Tests**				
Stenosis of extracranial internal carotid artery	+	-	-	-
Cardiac source of emboli	-	+	-	-
Other abnormality on tests	-	-	-	+

Table 3. TOAST Classification of High- and Medium-Risk Sources of Cardioembolism

High-risk sources

Mechanical prosthetic valve
Mitral stenosis with atrial fibrillation
Atrial fibrillation (other than lone atrial fibrillation)
Left atrial/atrial appendage thrombus
Sick sinus syndrome
Recent myocardial infarction (<4 weeks)
Left ventricular thrombus
Dilated cardiomyopathy
Akinetic left ventricular segment
Atrial myxoma
Infective endocarditis

Medium-risk sources

Mitral valve prolapse
Mitral annulus calcification
Mitral stenosis without atrial fibrillation
Left atrial turbulence (smoke)
Atrial septal aneurysm
Patent foramen ovale
Atrial flutter
Lone atrial fibrillation
Bioprosthetic cardiac valve
Nonbacterial thrombotic endocarditis
Congestive heart failure
Hypokinetic left ventricular segment
Myocardial infarction (> 4 weeks, < 6 months)

Appendix 2 - ASITN/SIR Collateral Vessel Grading System

Collateral vessel status was evaluated by using the American Society of Interventional and Therapeutic Neuroradiology/Society of Interventional Radiology (ASITN/SIR) collateral vessel grading system[28]. Collateral vessel scores are categorized into ASITN/SIR grades 0 or 1, 2, and 3 or 4. The following scoring system provides a guide.

Grade	Description
0	No collateral vessels visible to the ischemic site
1	Slow collateral vessels to the periphery of the ischemic site with persistence of some of the defect
2	Rapid collateral vessels to periphery of ischemic site with persistence of some of the defect and to only a portion of the ischemic territory
3	Collateral vessels with slow but complete angiographic blood flow of the ischemic bed by the late venous phase
4	Complete and rapid collateral blood flow to the vascular bed in the entire ischemic territory by retrograde perfusion

Appendix 3 - The Alberta Stroke Program Early Computed Tomography Score (ASPECTS)

NCCT shall be scored using ASPECTS, a 10-point score derived by examining each of 10 regions on the middle cerebral artery territory[29]. Ischemic change present is scored as 0; ischemic change absent is score as 1. Adding up the score gives a maximum of 10 (favorable scan) and minimum of 0 (unfavorable scan). The score is highly reliable when trichotomized into 0-4 (severe ischemic change, large core), 5-7 (moderate ischemic change) and 8-10 (minimal ischemic change, small core). ASPECTS may be less reliable early in stroke (i.e. within 90 minutes of onset); however, at later time windows it should be easy to recognize large areas of irreversible damage. Having a good quality scan and optimization of scanner is key to successful interpretation. Further information is available at: www.aspectsinstroke.com.

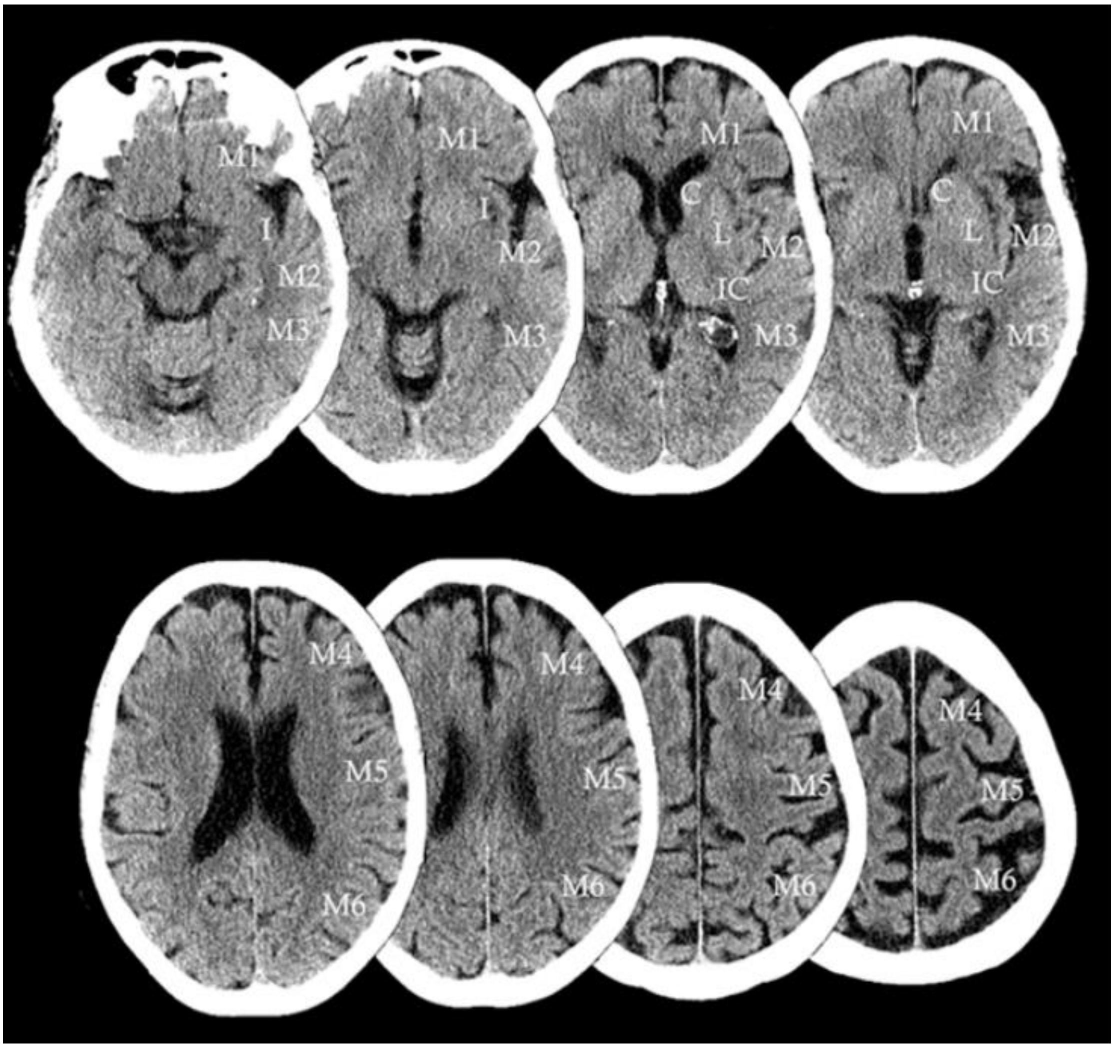

Appendix 4 - Modified Rankin Scale (MRS)

Grade	Description[30]
0	No symptoms at all
1	No significant disability despite symptoms: able to carry out all usual duties and activities
2	Slight disability: unable to carry out all previous activities but able to look after own affairs without assistance
3	Moderate disability: requiring some help, but able to walk without assistance
4	Moderately severe disability: unable to walk without assistance, and unable to attend to own bodily needs without assistance
5	Severe disability: bedridden, incontinent, and requiring constant nursing care and attention
6	Death

Appendix 5 - Expanded Thrombolysis In Cerebral Infarction (eTICI) Scale

Score	Discription[18]
0	No perfusion or anterograde flow beyond site of occlusion
1	Penetration but not perfusion. Contrast penetration exists past the initial obstruction but with minimal filling of the normal territory
2	Incomplete perfusion wherein the contrast passes the occlusion and opacifies the distal arterial bed but rate of entry or clearance from the bed is slower or incomplete when compared with non-involved territories
2a	Some perfusion with distal branch filling of < 50% of territory visualized
2b	Substantial perfusion with distal branch filling of ≥ 50% of territory visualized
2c	Near-complete perfusion except for slow flow in a few distal cortical vessels or presence of small distal cortical emboli
3	Complete perfusion with normal filling of all distal branches

Investigator's Agreement

I have read the attached protocol: a randomized, controlled, multicenter trial of Direct Endovascular treatment Versus standard bridging Therapy for acute stroke patients with large vessel occlusion in the anterior circulation (DEVT Trial), Version 2.0 dated 1st August 2019 and agree to abide by all provisions set forth therein. I agree to comply with the current International Conference on Harmonization Guidelines for Good Clinical Practice and the laws, rules, regulations and guidelines of the community, country, state or locality relating to the conduct of the clinical study. I also agree that persons debarred from conducting or working on clinical studies by any court or regulatory agency will not be allowed to conduct or work on studies for the sponsor.

Name Site Principal Investigator Signature

Name of Clinical Site Date

REFERENCES

1. Hacke W, Kaste M, Bluhmki E, et al. Thrombolysis with alteplase 3 to 4.5 hours after acute ischemic stroke. The New England journal of medicine 2008;359:1317-29.

2. National Institute of Neurological D, Stroke rt PASSG. Tissue plasminogen activator for acute ischemic stroke. The New England journal of medicine 1995;333:1581-7.

3. Muir KW, Ford GA, Messow C-M, et al. Endovascular therapy for acute ischaemic stroke: the Pragmatic Ischaemic Stroke Thrombectomy Evaluation (PISTE) randomised, controlled trial. Journal of neurology, neurosurgery, and psychiatry 2017;88:38-44.

4. Bracard S, Ducrocq X, Mas JL, et al. Mechanical thrombectomy after intravenous alteplase versus alteplase alone after stroke (THRACE): a randomised controlled trial. The Lancet Neurology 2016;15:1138-47.

5. Saver JL, Goyal M, Bonafe A, et al. Stent-retriever thrombectomy after intravenous t-PA vs. t-PA alone in stroke. The New England journal of medicine 2015;372:2285-95.

6. Jovin TG, Chamorro A, Cobo E, et al. Thrombectomy within 8 hours after symptom onset in ischemic stroke. The New England journal of medicine 2015;372:2296-306.

7. Goyal M, Demchuk AM, Menon BK, et al. Randomized assessment of rapid endovascular treatment of ischemic stroke. The New England journal of medicine 2015;372:1019-30.

8. Campbell BC, Mitchell PJ, Kleinig TJ, et al. Endovascular therapy for ischemic stroke with perfusion-imaging selection. The New England journal of medicine 2015;372:1009-18.

9. Berkhemer OA, Fransen PS, Beumer D, et al. A randomized trial of intraarterial treatment for acute ischemic stroke. The New England journal of medicine 2015;372:11-20.

10. Chandra RV, Lesliemazwi TM, Mehta BP, et al. Does the use of IV tPA in the current era of rapid and predictable recanalization by mechanical embolectomy represent good value. Journal of neurointerventional surgery 2016;8:443-6.

11. Grotta JC, Hacke W. Stroke Neurologist's Perspective on the New Endovascular Trials. Stroke; a journal of cerebral circulation 2015;46:1447-52.

12. Wang H, Zi W, Hao Y, et al. Direct endovascular treatment: an alternative for bridging therapy in anterior circulation large-vessel occlusion stroke. European journal of neurology : the official journal of the European Federation of Neurological Societies 2017;24:935-43.

13. Rai AT, Boo S, Buseman C, et al. Intravenous thrombolysis before endovascular therapy for large vessel strokes can lead to significantly higher hospital costs without improving outcomes. Journal of neurointerventional surgery 2018;10:17-21.

14. Ren Y, Churilov L, Mitchell P, Dowling R, Bush S, Yan B. Clot Migration Is Associated With Intravenous Thrombolysis in the Setting of Acute Ischemic Stroke. Stroke; a journal of cerebral circulation 2018;49:3060-2.

15. Mistry EA, Mistry AM, Nakawah MO, et al. Mechanical Thrombectomy Outcomes With and Without Intravenous Thrombolysis in Stroke Patients: A Meta-Analysis. Stroke; a journal of cerebral circulation 2017;48:2450-6.

16. Kaesmacher J, Mordasini P, Arnold M, et al. Direct mechanical thrombectomy in tPA-ineligible and -eligible patients versus the bridging approach: a meta-analysis. Journal of neurointerventional surgery 2019;11:20-7.

17. Powers Wj Fau - Rabinstein AA, Rabinstein Aa Fau - Ackerson T, Ackerson T Fau - Adeoye OM, et al. 2018 Guidelines for the Early Management of Patients With Acute Ischemic Stroke: A Guideline for Healthcare Professionals From the American Heart Association/American Stroke Association. stroke 2018;49.

18. Liebeskind DS, Bracard S, Guillemin F, et al. eTICI reperfusion: defining success in endovascular stroke therapy. Journal of neurointerventional surgery 2019;11:433-8.

19. Brott T, Adams HP, Jr., Olinger CP, et al. Measurements of acute cerebral infarction: a clinical examination

scale. Stroke; a journal of cerebral circulation 1989;20:864-70.

20. von Kummer R, Broderick JP, Campbell BC, et al. The Heidelberg Bleeding Classification: Classification of Bleeding Events After Ischemic Stroke and Reperfusion Therapy. Stroke; a journal of cerebral circulation 2015;46:2981-6.

21. Banks JL, Marotta CA. Outcomes validity and reliability of the modified Rankin scale: implications for stroke clinical trials: a literature review and synthesis. Stroke; a journal of cerebral circulation 2007;38:1091-6.

22. Quinn TJ, Dawson J, Walters MR, Lees KR. Reliability of the modified Rankin Scale: a systematic review. Stroke; a journal of cerebral circulation 2009;40:3393-5.

23. Herdman M, Gudex C, Lloyd A, et al. Development and preliminary testing of the new five-level version of EQ-5D (EQ-5D-5L). Quality of life research : an international journal of quality of life aspects of treatment, care and rehabilitation 2011;20:1727-36.

24. Broeg-Morvay A, Mordasini P, Bernasconi C, et al. Direct Mechanical Intervention Versus Combined Intravenous and Mechanical Intervention in Large Artery Anterior Circulation Stroke: A Matched-Pairs Analysis. Stroke; a journal of cerebral circulation 2016;47:1037-44.

25. Bellwald S, Weber R, Dobrocky T, et al. Direct Mechanical Intervention Versus Bridging Therapy in Stroke Patients Eligible for Intravenous Thrombolysis: A Pooled Analysis of 2 Registries. Stroke; a journal of cerebral circulation 2017;48:3282-8.

26. Berkhemer OA, Fransen PSS, Beumer D, et al. A randomized trial of intraarterial treatment for acute ischemic stroke. The New England journal of medicine 2015;372:11-20.

27. Adams HP, Jr., Bendixen BH, Kappelle LJ, et al. Classification of subtype of acute ischemic stroke. Definitions for use in a multicenter clinical trial. TOAST. Trial of Org 10172 in Acute Stroke Treatment. Stroke; a journal of cerebral circulation 1993;24:35-41.

28. Zaidat OO, Yoo AJ, Khatri P, et al. Recommendations on angiographic revascularization grading standards for acute ischemic stroke: a consensus statement. Stroke; a journal of cerebral circulation 2013;44:2650-63.

29. Pexman JH, Barber PA, Hill MD, et al. Use of the Alberta Stroke Program Early CT Score (ASPECTS) for assessing CT scans in patients with acute stroke. AJNR American journal of neuroradiology 2001;22:1534-42.

30. Bonita R, Beaglehole R. Recovery of motor function after stroke. Stroke; a journal of cerebral circulation 1988;19:1497-500.

Summary of Changes - Protocol DEVT Version 1.0 to Version 2.0

Below is the table of changes. Deleted items are identified with Strikethrough font. Additional wording is in bold font

Section(s)	Protocol Version 1.0<br>Change From:	Protocol Version 2.0<br>Change To:	Rationale
**List of Abbreviations**		**MedDRA-Medical Dictionary for Regulatory Activities**<br>**SOC-System Organ Class**	Addition
**Schedule of Assessments**	Weight	Weight[★]<br>[★]**The subject's actual weight will be measured in hospital using standard hospital scales (i.e., stand up or in-bed scales if the subject is not ambulatory). If actual weight cannot be measured for any reason (due to, for example severe illness or unavailability of in-bed scales at the site), weight will be determined by first asking the subject, second asking a family member or third by estimation.**	Clarification
**Schedule of Assessments**	Prior medications<br>Concomitant medications	Prior medications[§]<br>Concomitant medications[§]<br>[§]**Prior and concomitant medications will be listed per patient, with the listings separated within treatment group.**	Clarification
**Study Synopsis - Assessment of Efficacy**<br>**&**<br>**Section 8.2 Secondary Efficacy Outcomes**	Successful recanalization proportion immediate after EVT. Successful recanalization is defined as a modified Treatment in Cerebral Infarction score of 2b (50 to 99% reperfusion) or 3 (complete reperfusion) in the post-procedure angiography	Successful recanalization proportion immediate after EVT. Successful recanalization is defined as a **expanded Thrombolysis In Cerebral Infarction score** of 2b (**substantial perfusion**), **2c (near-complete perfusion)** or 3 (complete reperfusion) in the post-procedure angiography	Updated definition, in response to a comment received from the Research Committee
**Section 1 BACKGROUND INFORMATION**		**In addition, IVT before EVT is associated with an increased incidence of clot migration, resulting in an**	Addition

		**increased rate of clots inaccessibility by mechanical thrombectomy. [reference: *Stroke* 2017;48:2450-6.]**<br><br>**Another meta-analysis showed that direct endovascular treatment may carries comparable effectiveness and safety as compared with bridging therapy by pooling studies with lower selection bias. [reference: *Journal of neurointerventional surgery* 2019;11:20-7.]**	
**Section 10.1 The Modified Rankin Scale**	Premorbid mRS status will also be obtained retrospectively ~~at 24 Hours~~	Premorbid mRS status will also be obtained retrospectively **and reported on the 24h CRF page.**	Clarification of the premorbid mRS collection time and reporting on the CRF
**Section 16.8 SAEs**		**SAEs over the 90-day study period will be summarized by presenting, for each treatment group, the number and percentage of subjects having at least one SAE, having an SAE in each body system and preferred term, by severity and relatedness to study medication. The frequencies and incidences of SAEs occurring in subjects in the active and control groups will be summarized within treatment group by the Medical Dictionary for Regulatory Activities (MedDRA) System Organ Class (SOC). The frequencies and incidences of SAEs and discontinuations due to SAEs occurring in subjects in the active and control groups will be summarized within treatment group**	Addition,<br>To be consistent with the Statistical Analysis Plan
**Section 16.9 AEs**		**Additional analyses will consider the frequency of AEs and discontinuations due to AEs. AEs will be summarized by presenting, for each treatment group, the number and percentage of subjects having any AE, having an AE in each body system and preferred term. Severity and**	Addition,<br>To be consistent with the Statistical Analysis Plan

		**relatedness to study medication will be recorded. The frequencies and incidences of AEs occurring in subjects in the active and control groups will be summarized within treatment group by the Medical Dictionary for Regulatory Activities (MedDRA) System Organ Class (SOC).**	
**Section 23. STUDY ORGANIZATION AND FUNDING**	DEVT trial is an investigator-initiated study which is organized by the second affiliated hospital of the Third Military Medical University and conducted in about 30 comprehensive stroke centers in China. The authors disclosed receipt of the following financial support: (1) National Science Fund for Distinguished Young Scholars (No. 81525008), and (2) Major clinical innovation technology project of the Second Affiliated Hospital of the Army Military Medical University (No. 2018JSLC0017). The funders had no involvement in the study design, data collection, analysis and interpretation, writing or decision to submit the paper.	DEVT trial is an investigator-initiated study which is organized by the second affiliated hospital of the Third Military Medical University and conducted in about 30 comprehensive stroke centers in China. The authors disclosed receipt of the following financial support: (1) National Natural Science Foundation of China (Nos. 81525008, **81901236, 81801157**), (2) **Chongqing Major Disease Prevention and Control Technology Research Project (No. 2019ZX001),** (3) Major clinical innovation technology project of the Second Affiliated Hospital of the Army Military Medical University (No. 2018JSLC0017), and (4) **Clinical Medical Research Talent Training Program of Army Medical University (2019XLC2008, 2019XLC3016)**. The funders had no involvement in the study design, data collection, analysis and interpretation, writing or decision to submit the paper.	Addition
**Appendix 5 - Modified Treatment In Cerebral Infarction (mTICI) Score**	~~Modified Treatment In Cerebral Infarction (mTICI) Score~~	**Expanded Thrombolysis in Cerebral Infarction Scale**	Updated definition, in response to a comment received from the Research Committee
