# Supplementary material for: Post-stroke acute heart failure in patients with large vessel occlusion undergoing endovascular treatment: A pooled analysis of individual patient data from multicenter studies with mediation analysis
Source: PLoS Med. 2026 Jul 7;23(7):e1004752. doi: 10.1371/journal.pmed.1004752 (PMC13340808; doi:10.1371/journal.pmed.1004752)
Supplement: S1 File — This compressed file contains the study protocols for the four multicenter studies included in this pooled analysis: BASILAR registry, DEVT trial, RESCUE-BT trial, and MARVEL trial. Each protocol document is provided in PDF format. (ZIP) [file pmed.1004752.s005.zip › MARVEL_no_logo.pdf]

MARVEL： Methylprednisolone for acute large vessel occlusion: a randomized double-blind, placebo-controlled trial in revascularization patients

This supplement contains the following items:

1. Original protocol (page1 to 95), final protocol (page96 to 187), summary of changes (page188 to 190).

Note: personal identifying information has been redacted from the protocol and SAP documents to comply with international privacy legislation.

Clinical study protocol

Efficacy and safety of intravenous methylprednisolone sodium succinate in acute ischemic stroke patients underwent endovascular treatment: a multicenter, randomized, double-blind trial.

Principal Investigators

Wenjie Zi, MD. and Qingwu Yang, Ph.D.

Xinqiao Hospital, Army Medical University, Chongqing, China

Prepared by:

Wenjie Zi, MD, Xinqiao Hospital, Army Medical University, Chongqing, China

Qingwu Yang, Ph.D, Xinqiao Hospital, Army Medical University, Chongqing, China

Protocol Version: 1.0

Date 2021 September 29

Table of Contents

1. Study Synopsis 8

2. Flow chart and schedule of assessment 15

2.1 Flow Chart 15

2.2. Schedule of Assessments 16

3. List of Abbreviations 18

4. Background 20

4.1 The burden of stroke 20

4.2 Treatment in acute ischemic stroke 20

4.3 Glucocorticoid in Stroke 22

5. Study Objectives 24

6. Product characteristics of methylprednisolone 25

6.1 Description of the investigational medicinal product 25

6.2 Indications and Usage 26

6.3 Contraindications 29

6.4 Warning 29

7. Organizational Structure 36

7.1 Funding 37

7.2 Trials Boards and Committees 37

7.2.1 Steering Committee 37

7.2.2 Data Safety and Monitoring Board 37

7.2.3 Ethics Advisory Board 38

7.2.4 Imaging Core Laboratory 38

7.2.5 Clinical Events Committee 38

8. Design 39

9. Population 39

9.1 Inclusion criteria 39

9.2 Exclusion Criteria 40

9.3 Sample Size 41

10. Imaging Training 42

11. Randomization 42

12. Blinding/Unblinding 43

13. Study Treatment 44

13.1 Formulation, Packaging, and Handling 44

13.2 Test Product 44

13.3 Comparator 44

13.4 Dosage, Administration, and Compliance 45

13.4.1 Methylprednisolone and its Placebo 45
13.4.2 Concomitant therapy 45
13.4.4 Drug Accountability 46

14. Study Schedule 46

14.1 Screening period (Visit V0, Randomization) 47
14.1.1 Visit V0 47
14.1.2 Randomization (R) 48
14.2 Treatment period (Visits V1， V2) 48
14.2.1 Visit 1 48
14.2.2 Visit 2 49
14.3 Follow Up period (Visits V3, V4) 49
14.3.1 Visit 3 50
14.3.3 Visit 4 50

15. Endpoints of the Clinical Trial 50

15.1 Efficacy Analysis-Primary Endpoint 50
15.2 Efficacy Analysis-Secondary Endpoints 51
15.3 Safety Analysis-Primary Endpoint 51
15.3.1 Primary Safety Endpoints 51
15.3.2 Secondary Safety Endpoints 52

16. Assessment 52

16.1 Assessment of efficacy 53
16.1.1 The modified Rankin Scale (mRS) 53
16.1.2 The National Institutes of Health Stroke Scale (NIHSS) 54
16.1.3 European Quality Five Dimensions Five Level scale 55
16.2 Assessment of safety 55
16.2.1 Mortality at 90±7 days 55
16.2.2 Symptomatic Intracranial Haemorrhage 55
16.2.3 Any ICH within 48 hours 56
16.2.4 Proportion of patients with pneumonia 57
16.2.4 Proportion of patients with gastrointestinal bleeding 57
16.2.5 Adverse events 57
16.2.6 Serious Adverse Event 60
16.2.7 Adverse Event Recording and Follow Up 61
16.2.8 Serious Adverse Events Reporting 62

17. Study Discontinuation Criteria 63

17.1 Patient Discontinuation 63
17.2 Study Treatment Discontinuation 64
17.3 Study and Site Discontinuation 64

18. Statistical Analysis 65

18.1 Determination of sample size 65
18.2 Analysis population 66
18.2.1 Intention-to-Treat Population 66

18.2.2. Per-Protocol Population ... 67
18.2.3. Safety Population ... 68
18.2 Analysis of Efficacy–primary endpoint ... 68
18.3 Analysis of Efficacy – secondary endpoints ... 69
18.4 Safety analysis ... 69
18.5 Tolerability Analysis (adverse events) ... 70
18.5.1 Adverse Events ... 70
18.5.2 Serious Adverse Events ... 70
18.5.3 All Adverse Events ... 70
18.6 Adjustment for Covariates and Subgroup Analyses ... 71
18.7 Missing data handling ... 72
19. Ethical and regulatory consideration ... 72
19.1 General requirements and Considerations ... 72
19.2 Study monitoring and quality control ... 73
19.3 Informed Consent ... 74
19.4 Confidentiality ... 75
19.5 Liability and Insurance ... 75
20. Administrative procedures ... 75
20.1 Curriculum vitae ... 75
20.2 Secrecy agreement ... 76
20.3 Ownership of data and use of the study results ... 76
20.4 Protocol amendments ... 76
21. Data Retention ... 77
22. Study report and publications ... 77
25. Appendices ... 81
25.1 Appendix 1: mRS ... 81
25.2 Appendix 2: NIHSS ... 82
25.2 Appendix 3: EQ-5D ... 90

1. **Study Synopsis**

**Study Title**	MARVEL: Methylprednisolone for acute large vessel occlusion: a randomized double-blind, placebo-controlled trial in recanalization patients
**Study registry number**	**registry number:** ChiCTR2100051729
**Indication**	Acute Ischemic Stroke
**Study Centres**	About 80 stroke centres in China
**Number of Subjects**	1672 (836 subjects per group)
**Study Estimate Duration**	February 2022 until June 2023
**Study Objectives**	To test the safety and efficacy of adjunctive low-dose methylprednisolone sodium succinate therapy for the treatment of acute ischemic stroke patients within 24 hours of onset who underwent EVT.
**Study Design**	A multicentre, randomized, double-blind, placebo-controlled trial
**Study Population**	Patients with large vessels occlusive stroke within 24 hours that underwent endovascular treatment
**Inclusion Criteria**	● Age ≥ 18 years;<br>● The time from onset to randomization was within 24 hours;<br>● Anterior circulation ischemic stroke was preliminarily determined according to clinical symptoms or imaging examination;<br>● Baseline Alberta Stroke Program Early CT Score (ASPECTS) ≥ 5;<br>● Baseline National Institutes of Health Stroke Scale (NIHSS) ⩾ 6<br>● Computed tomography angiography (CTA) / magnetic resonance angiography (MRA) /digital subtraction angiography (DSA) confirmed occlusion of intracranial segment of

	internal carotid artery and middle cerebral artery who decided to receive endovascular treatment.<br>Written informed consent signed by patients or their family members.
**Exclusion Criteria**	● Intracranial hemorrhage confirmed by cranial computed tomography (CT) or magnetic resonance imaging (MRI);<br>● mRS score ≥ 2 before onset;<br>● Pregnant or lactating women;<br>● Allergic to contrast agents;<br>● Allergic to glucocorticoids;<br>● Participating in other clinical trials;<br>● Systolic blood pressure > 185 mmHg or diastolic pressure > 110 mmHg, and oral antihypertensive drugs can not control;<br>● Genetic or acquired bleeding constitution, lack of anticoagulant factors; Or oral anticoagulants and INR > 1.7;<br>● Blood sugar < 2.8 mmol/L (50 mg/dl) or > 22.2 mmol/L (400 mg/dl), platelet < 90 x 10^9/L;<br>● The artery is tortuous and the thrombectomy device cannot reach the target vessel;<br>● Bleeding history (gastrointestinal and urinary tract bleeding) in recent 1 month;<br>● Chronic hemodialysis and severe renal insufficiency (glomerular filtration rate < 30 ml/min or serum creatinine > 220 umol/L [2.5 mg/dL]);<br>● Life expectancy due to any advanced disease < 6 months;<br>● Follow-up is not expected to be completed;<br>● Intracranial aneurysm and arteriovenous malformation;<br>● Brain tumors with imaging mass effect;

	Systemic infectious diseases.
**Study drug**	Methylprednisolone sodium succinate group: The box of the methylprednisolone group contains 12 bottles, each containing 40mg of methylprednisolone sodium succinate. Both the bottle and box are labelled as "Study Drug".<br><br>Placebo group: The box of the placebo group contains 12 bottles, each containing 40mg of placebo. Both the bottle and box are labelled as "Study Drug".
**Randomization**	Eligible patients will be consecutively randomized to treatment with either Methylprednisolone or Placebo group with a ratio of 1:1 by a web-based APP (Jinlingshu) on mobile phone or computer (https://jinlingshu.com/). Randomization will be stratified by participating centres permutation block size of 4.
**Treatments**	Patients will be assigned to receive either a placebo or methylprednisolone, with a dosage of 2mg/kg (based on estimated or actual weight if known, not exceeding a maximum dose of 160 mg) per day for a duration of 3 days. The initial study drug will be administered as soon as possible after randomisation. It is recommended that the initial study drug administrated before arterial access closure, but it should not be delayed more than 2 hours after arterial access closure.
**Consent**	Explicit written, signed informed consent from the subject or legally authorized representative will be obtained prior to any protocol specific procedures.
**Criteria for Evaluation-Efficacy**	Primary Efficacy Endpoint<br>● Reduced disability level (ordinal shift in mRS score) at 90 ±7 days;<br>Secondary Efficacy Endpoints<br>● Proportion of patients with mRS score 0 to 4 at 90 ±7 days;

	● Proportion of patients with mRS score 0 to 3 at 90 ±7 days;<br>● Proportion of patients with mRS score 0 to 2 at 90 ±7 days;<br>● Proportion of patients with mRS score 0 to 1 or returned to pre-stroke mobidity at 90 ±7 days;<br>● NIHSS score at 5-7 days or at early discharge;<br>Health-related quality of life [European Quality of Life Five-Dimension visual-analogue scale (EQ-5D VAS)] at 90 ±7 days.
**Criteria for Evaluation-Safety**	Primary Safety Endpoints<br>● Mortality at 90 ±7 days;<br>● Proportion of patients with symptomatic intracranial haemorrhage within 48 hours. Symptomatic intracranial haemorrhage will be adjudicated by an independent Imaging Core Laboratory according to the modified Heidelberg Bleeding Classification;<br>Secondary Safety Endpoints<br>● Proportion of patients with any radiologic intracranial haemorrhage within 48 hours;<br>● Proportion of patients with pneumonia;<br>● Proportion of patients with gastrointestinal haemorrhage within 7 days after EVT;<br>● Incidence of serious adverse events.<br>Incidence of any adverse events.
**Sample Size Calculation**	The current trial is designed detect a shift on the modified Rankin Scale score, which represents the global disability, to a lower score. The expected distribution of the modified Rankin Scale score, derived from the EVT for acute anterior circulation ischemic stroke (ACTUAL) registry and the Highly Effective Reperfusion evaluated in Multiple Endovascular Stroke Trials (HERMES) collaboration, is as follows: 7%, 20%, 16%, 11%, 13%, 8% and 25%.<br>The sample size was calculated using the cumulative proportion of patients with mRS 0-2. We assume a moderate effect of 7% absolute increase in the cumulative proportion of patients with mRS 0-2 in the intervention group, compared with controls, indicating an odds ratio

	(OR) of 1.33, which have substantially exceeds the minimal clinically important difference.10<br>In order to demonstrate the expected treatment effect with a type-1 error alpha =0.05 (two-tailed) and a power of 80% (beta = 20%). A sample size of n= 1588 patients (n=794 per treatment group) is required.<br>The intention-to-treat principle will be applied to the primary analysis and, therefore, to safeguard against dilution of the treatment effect associated with an approximate 5% non-adherence rate (due to loss to follow-up, consent withdrawal and other reasons), we initially planned to enrol n=1672 patients (n=836 per treatment group) for this study. This estimation was performed based on PASS (NCSS, LLC. Kaysville, Utah, USA) version 15.0.
**Statistical Methods**	All efficacy analyses will be conducted on data from all randomly assigned patients according to the intention-to-treat principle. All efforts will be made to minimize the amount of missing data. Sensitivity analyses based on different hypotheses about the missingness pattern of the primary outcome will be performed to test for the robustness of the primary analysis. Analyses will also be repeated according the per protocol principle.<br>Analysis of Efficacy - primary endpoint:<br>The primary efficacy endpoint is disability evaluated 90 (±7) days after randomization using the modified Rankin Scale (mRS) score. The primary treatment effect will be estimated using Common Odds Ratio or assumption free method. Covariable were age, baseline NIHSS score, pre-stroke mRS, baseline ASPECTS score, use of intravenous thrombolysis, time from onset to randomization, and occlusion location.<br>Analysis of Efficacy - Secondary endpoint:<br>Proportion of with mRS score 0 to 4 at 90 ±7 days and proportion of mRS score 0 to 3 at 90 ±7 days, proportion of patients with mRS score 0 to 2 at 90 ±7 days, proportion of mRS score 0 to 1 or return to pre-morbid mRS score at 90 days (for patients with mRS >1). Between-group

	differences will be tested using generalized linear models. The risk ratios and corresponding 95%CI will be provided.<br>NIHSS score at 5-7 days or at early discharge and Health-related quality of life (EQ-5D VAS). The results will be compared between two randomized treatment groups using GLM. The mean difference and corresponding 95%CI will be provided. Normality and variance homogeneity of residuals will be accessed graphically. The win ratio method will be used if normality assumption is seriously violated.<br>Analysis of Safety:<br>The safety analysis will be conducted on the Safety Population.<br>Mortality due to any cause at 90 ±7 days. The difference of mortality in two treatment groups will be tested using modified Poisson Regression. In addition, the Kaplan-Meier method will be used to assess the mortality. The log-rank test will be applied to compare the two treatment groups. Cox regression model will be employed to calculate the hazard ratio with 95%CI.<br>In addition to mortality due at 90 ±7 days, SICH within 48 hours is considered to be another primary safety outcome. SICH will be evaluated according to the modified Heidelberg Bleeding Classification. Besides, modified Poisson regression will be fitted to estimate the risk ratio associated with the treatment effect. The proportion of Any radiologic ICH within 48 hours between methylprednisolone and placebo groups will be tested in the same manner as SICH.<br>Proportion of patients with pneumonia and proportion of patients with gastrointestinal haemorrhage within 7 days after EVT will be explore and tested the same as SICH and any ICH.<br>Adverse events (AE) and serious adverse event (SAE) will be summarized and presented by treatment group for all patients in the Safety Population. The incidences will be compared via

	Fisher's exact test or chi-square test as appropriate.

2. Flow chart and schedule of assessment

2.1 Flow Chart

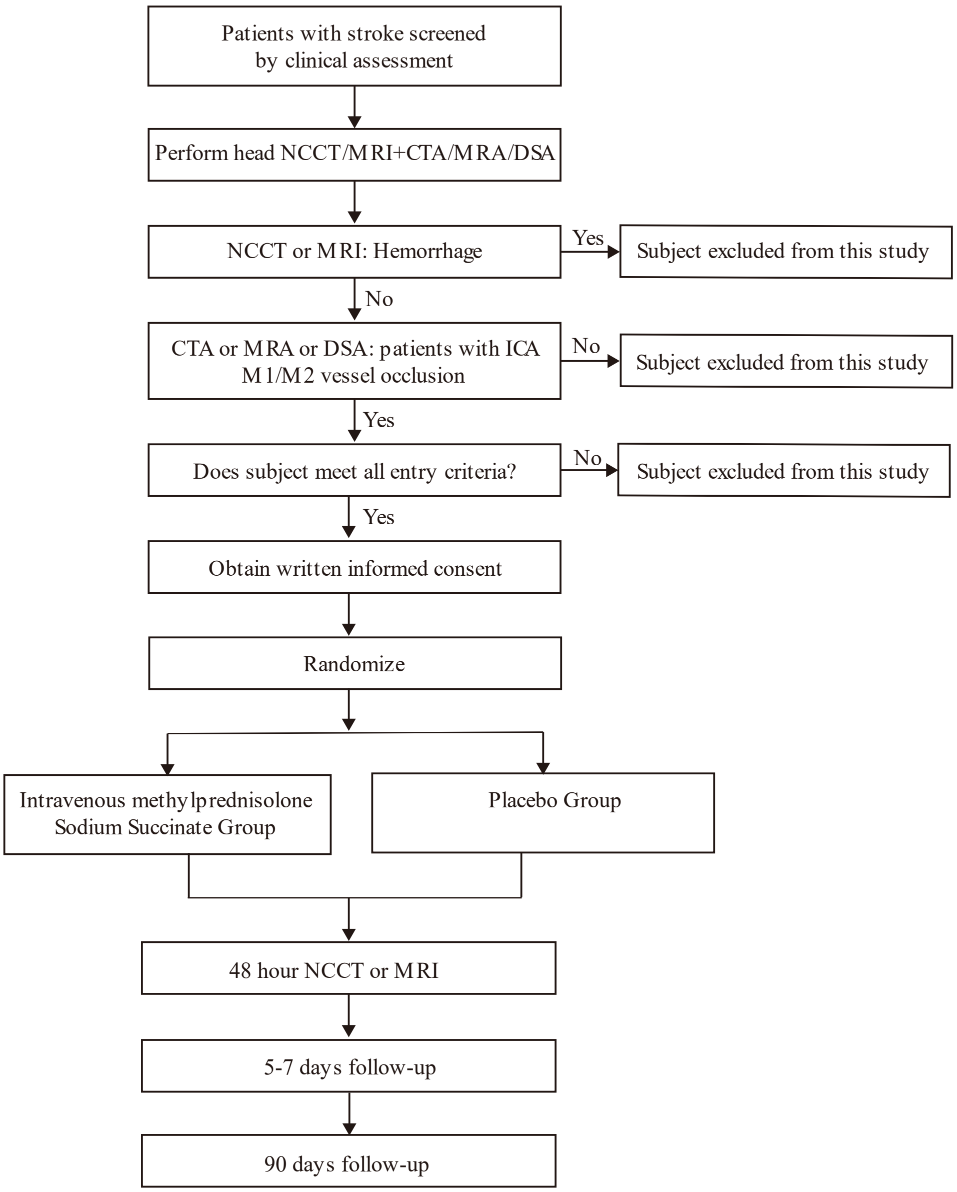

2.2. Schedule of Assessments

	**Visit (Time in hours / days from EVT =V1)**					
	Screening		Treatment		Follow Up	
	**V0**	**R**	**V1**[a] Study medication administration	**V2** (48-96h)	**V3**[b] (5-7d)	**V4** (90±7d)
Informed consent	X					
Demographic data	X					
Medical history	X					
Physical examination	X			X	X	
Pre-stroke mRS	X					
NIHSS	X				X	
mRS						X
EQ-5D						X
Previous medication	X					
Blood pressure and heart rate	X		X	X[e]	X	
Local laboratory result	X			X		
Pregnancy test[c]	X					

Brain CT/MRI plus CTA/MRA/DSA	X					
Brain CT/MRI scan[d]				X		
Inclusion / exclusion criteria	X					
Randomization		X				
Study medication			X			
Concomitant medication	X		X	X	X	X
12-lead ECG	X					
48h monitoring			X[a]			
Adverse events			X	X	X	X

V = Visit; R = Randomization; h = hour; d = day

[a] 48h monitoring in Unit or equivalent unit including repeated measurements of blood pressure, heart rate and body temperature

[b] or hospital discharge if < 5 days

[c] Mandatory for women of childbearing potential

[d] also to be performed in any case of neurological deterioration during the first 96h

[e] every hour

3. List of Abbreviations

AE	Adverse Event
AIS	Acute Ischemia Stroke
BI	Barthel Index
CRF	Case Report Form
CT	Computed Tomography
CTA	Computed Tomography Angiography
DSMB	Data Safety Monitoring Board
EAB	Ethics Advisory Board
ECG	Electrocardiogram
EQ-5D	European Quality Five-Dimension
EVT	Endovascular Therapy
GCP	Good Clinical Practice
GOS	Glasgow outcome scale
HBC	Heidelberg bleeding classification
ICH	Intracerebral Haemorrhage
ICH-GCP	International Conference on Harmonization-Good Clinical Practice
IRB	Institutional Review Board
ITT	Intention-to-Treat
IVT	Intravenous Thrombolysis
LVO	Large Vessel Occlusion
MARVEL	MARVEL: Methylprednisolone for acute large vessel occlusion: a

	randomized double-blind, placebo-controlled trial in revascularization patients
MedDRA	Medical Dictionary for Regulatory Activities
MR	Magnetic Resonance
MRA	Magnetic Resonance Angiography
mRS	modified Rankin Scale
NIHSS	National Institutes of Health Stroke Scale
NINDS	National Institute of Neurological Disorders and Stroke
PI	Principal Investigator
PP	Per-Protocol
REB	Research Ethics Board
RISS	Rapidly improving stroke symptoms
RR	Risk ratio
rt-PA	recombinant tissue Plasminogen Activator
SAB	Scientific Advisory Board
SAE	Serious Adverse Event
SAP	Statistical Analysis Plan
SICH	Symptomatic Intracerebral Haemorrhage
SITS-MOST	Safe Implementation of Thrombolysis in Stroke–Monitoring Study
SOC	System Organ Class

4. Background

4.1 The burden of stroke

Stroke remains the second-leading cause of death and the third-leading cause of disability combined in the world.[1-3] The estimated global cost of stroke is over US $721 billion (0.66% of the global GDP).[3] In the past 20 years, the burden increased substantially, with the bulk of the global stroke burden residing in lower-income and lower-middle-income countries.[3] Recently, the National Epidemiological Survey of Stroke in China (NESS-China) suggested that the current stroke prevalence (1115/100 000 [95%CI, 997–1233]) in China appears to be the highest among other low- to middle-income countries (range from 536 to 1040/100 000), but significantly lower than that observed in high-income countries (range from 2600 to 8000/100 000). [4]

4.2 Treatment in acute ischemic stroke

Stroke is normally classified as either ischemic or haemorrhagic stroke although 62.4% of cases belong to ischemic nature. Ischemic stroke is characterized by a high incidence, high disability rate, and high mortality rate, and has become the leading cause of death in China.[4] Currently, there are about 2.4 million new stroke patients and 1.1 million deaths in China each year, imposing a heavy burden on society and families.[4] Approximately 40% of acute ischemic strokes are caused acute large vessel occlusion (LVO), with anterior circulation LVO accounting for about 80% and posterior circulation LVO accounting for about 20%.[4]

Early restoration of blood flow and salvage of ischemic penumbra are the current theoretical basis of the treatment of AIS.[5,6] The current standard therapy for acute ischemic stroke including the intravenous thrombolysis and endovascular treatment.[6] Currently, the recanalization rate of acute anterior circulation LVO stroke has been improved to 80%~94.0% in the endovascular treatment era.[7]

However, the successful recanalization rates have not been completely translated into clinical benefits for AIS patients, and the proportion of patients with good outcome was less than 50%, which is clinically referred to as "Futile recanalization (FR)" or “Ineffective reperfusion”.[8,9] That is, successful recanalization of occluded vessels (defined as modified treatment in cerebral ischemia (mTICI) blood flow grade ≥2b) does not lead to good functional outcomes (mRS score ≥3 at 90 days). Moreover, reperfusion of the ischaemic tissue is not risk-free, often causes haemorrhagic transformation (HT), blood–brain barrier (BBB) disruption.[10-12] Besides, malignant brain oedema was a common complication that were associated with high mortality.[13,14]

Several mechanisms may contribute futile reperfusion and may be the potential treatment target for the futile reperfusion phenomenon: the non-reflow phenomenon, cerebral oedema, blood-brain barrier damage and inflammatory damage.[12]

Firstly, the "non-reflow" phenomenon refers to tissue hypoperfusion despite the timely or complete recanalization of an occluded artery.[15] This phenomenon is frequently observed in clinical practice, with a prevalence of more than half in cases of coronary

artery occlusion, and it serves as an independent predictor of adverse outcomes.[16] In the cerebral circulation, non-reflow has consistently been demonstrated in preclinical studies since first being described in 1968.[15] Non-reflow in the brain may involve post-ischemic swelling in endothelial cells, microvasculature occluded by platelet and neutrophils, interstitial oedema, and inflammatory reaction.[17] Recent experimental data further suggest that non-reflow is present during the first 24 hours after recanalization.[17] In clinical studies, the prevalence of the no-reflow phenomenon after endovascular treatment (EVT) in acute ischemic stroke was approximately 30%, and this number is expected to increase with the extension of EVT to a 24-hour time window.[17] Secondly, the endovascular treatment has a damage to BBB, and will lead to severe brain edema and haemorrhagic transformation that considerably contribute to neurological deterioration and death.[11] Thirdly, inflammatory mechanisms after stroke are now increasingly considered prime targets for stroke therapy since immune signals and their mediators can have both detrimental and beneficial effects at different stages of the disease process.[18,19]

Thus, due to the multi-faceted nature of the aforementioned mechanisms, single-target agents may not sufficiently intervene. Therefore, combinations of agents or multi-target agents may be necessary to address these mechanisms effectively.

4.3 Glucocorticoid in Stroke

In animal models, corticosteroids have shown the ability to reduce infarct size, regulate cerebral blood flow, enhance non-reflow phenomenon, stabilize the blood-brain barrier,

prevent angiogenic edema, and modulate immune response.[20-28], while none have been adopted through clinical trial success in acute ischemic stroke therapy.[29,30]

Several lessons are learned from prior studies. Firstly, those trials were performed in the era when reperfusion strategies were not well established. The pre-clinical study of corticosteroids has suggested that corticosteroid was effective in the transient middle cerebral artery occlusion(tMCAO) model but was ineffective in the permanent middle cerebral artery occlusion(pMCAO) model.[21] It suggested that reperfusion or not was an important condition for the effect of corticosteroids.[21,31-33] In past corticosteriod treatment trials, conducted before the advent of mechanical thrombectomy as a treatment option, it is likely that many patients failed to reperfuse; the candidate adjuvant treatments were tested in the more challenging setting of permanent rather than transient brain ischemia and thus failed to show benefit in clinical trials.[33] The STAIR (Stroke Treatment Academic Industry Roundtable) suggested testing the neuroprotective agents in the new endovascular treatment era when the reperfusion rate can be up to 90%. [31,34] [35] Secondly, most included patients with "presumed ischemic stroke" were treated in an era where reperfusion was not effective, and the sample size of patients were small in the previous trials. Only 8/24 published trials of corticosteroids in stroke were acceptable for further analysis, and these comprised woefully inadequate numbers of patients (only 466). Davis and Donnan suggested that steroid therapy for stroke be discarded prematurely. Thirdly, most trials used a high-dose and long duration of corticosteroid thus increasing the incidence of major gastrointestinal hemorrhage and infections. [29]

Therefore, there was a call to reignite the study of corticosteroids in stroke, especially in the reperfusion era. [35,36] One small sample size trial in China has studied the use of corticosteroids in the new endovascular treatment era and have yielded promising result that a short course and low-dose corticosteroid can improve the functional outcome, which was consistent with the preclinical study.[37] The surprising result indicated that low-dose and short-term corticosteroids may have an effect for patients with ischemic stroke in the new EVT era. A ray of hope comes to restudy the effect of corticosteroids in the EVT era. Trials conducted on patients who underwent endovascular treatment closely aligned with the primate model of transient middle cerebral artery occlusion, where the corticosteroid has demonstrated its efficacy. This is in contrast to the permanent middle cerebral artery occlusion model, where the corticosteroid has shown its benefit.[21]

As an intermediate-acting corticosteroid with its minimal side effects and its ability to effectively cross the blood-brain barrier, methylprednisolone is widely used in clinical practice. We propose a multicenter, prospective, randomized, double-blind, placebo-controlled trial to investigate the efficacy and safety of adjunctive methylprednisolone for patients underwent endovascular treatment. The main objective of this trial is to determine whether the early administration of methylprednisolone can improve the clinical outcomes of patients with acute anterior circulation large vessel occlusive stroke after successful recanalization.

5. Study Objectives

The purpose of MARVEL trial is to investigate the efficacy and safety of early adjunctive methylprednisolone in AIS patients underwent EVT in a multicenter, randomized, double-blind, placebo-controlled trial. The findings of MARVEL are likely to have a direct impact on clinical practice. In case of a positive result, this will provide evidence for an effective, safe, and affordable treatment in the new EVT era. Additionally, this trial will contribute high-quality evidence on the use of corticosteroids in stroke through a large-scale, multicenter trial, addressing existing gaps in current knowledge.

6. Product characteristics of methylprednisolone

The following is the summary of product characteristics for methylprednisolone provided by the Chongqing Lummy Pharmaceutical Co., Ltd., Chongqing, China.

6.1 Description of the investigational medicinal product

Methylprednisolone sodium succinate, is the sodium succinate ester of methylpred-nisolone, and it occurs as a white, or nearly white, odorless hygroscopic, amorphous solid. It is very soluble in water and in alcohol; it is insoluble in chloroform and is very slightly soluble in acetone.

The chemical name for methylprednisolone sodium succinate is pregna-1,4-diene-3,20dione,21-(3-carboxy-1-oxopropoxy)-11,17-dihydroxy-6-methyl-monosodium salt, (6α, 11β), and the molecular weight is 496.53. The structural formula is represented below:

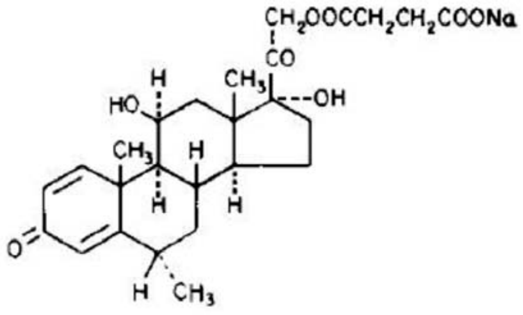

Methylprednisolone sodium succinate is soluble in water; it may be administered in a small volume of diluent and is well suited for intravenous use in situations where high blood levels of methylprednisolone are required rapidly.

CLINICAL PHARMACOLOGY

Glucocorticoids, naturally occurring and synthetic, are adrenocortical steroids that are readily absorbed from the gastrointestinal tract. Naturally occurring glucocorticoids (hydrocortisone and cortisone), which also have salt-retaining properties, are used as replacement therapy in adrenocortical deficiency states. Their synthetic analogs are primarily used for their potent anti-inflammatory effects in disorders of many organ systems. Glucocorticoids cause profound and varied metabolic effects. In addition, they modify the body's immune responses to diverse stimuli. Methylprednisolone is a potent anti-inflammatory steroid with greater anti-inflammatory potency than prednisolone and even less tendency than prednisolone to induce sodium and water retention. Methylprednisolone sodium succinate has the same metabolic and anti-inflammatory actions as methylprednisolone.

6.2 Indications and Usage

When oral therapy is not feasible, and the strength, dosage form, and route of administration of the drug reasonably lend the preparation to the treatment of the condition, the intravenous or intramuscular use of Methylprednisolone is indicated as follows:

Allergic states: Control of severe or incapacitating allergic conditions intractable to adequate trials of conventional treatment in asthma, atopic dermatitis, contact dermatitis, drug hypersensitivity reactions, perennial or seasonal allergic rhinitis, serum sickness, transfusion reactions.

Dermatologic diseases: Bullous dermatitis herpetiformis, exfoliative erythroderma, mycosis fungoides, pemphigus, severe erythema multiforme (Stevens-Johnson syndrome).

Endocrine disorders: Primary or secondary adrenocortical insufficiency (hydrocortisone or cortisone is the drug of choice; synthetic analogs may be used in conjunction with mineralocorticoids where applicable; in infancy, mineralocorticoid supplementation is of particular importance), congenital adrenal hyperplasia, hypercalcemia associated with cancer, nonsuppurative thyroiditis.

Gastrointestinal diseases: To tide the patient over a critical period of the disease in regional enteritis (systemic therapy) and ulcerative colitis. Hematologic disorders: Acquired (autoimmune) hemolytic anemia, congenital (erythroid) hypoplastic anemia (Diamond-Blackfan anemia), idiopathic thrombocytopenic purpura in adults

(intravenous administration only; intramuscular administration is contraindicated), pure red cell aplasia, selected cases of secondary thrombocytopenia.

Miscellaneous: Trichinosis with neurologic or myocardial involvement, tuberculous meningitis with subarachnoid block or impending block when used concurrently with appropriate antituberculous chemotherapy.

Neoplastic diseases: For the palliative management of leukemias and lymphomas.

Nervous System: Acute exacerbations of multiple sclerosis; cerebral edema associated with primary or metastatic brain tumor, or craniotomy.

Ophthalmic diseases: Sympathetic ophthalmia, uveitis and ocular inflammatory conditions unresponsive to topical corticosteroids. Renal diseases: To induce diuresis or remission of proteinuria in idiopathic nephrotic syndrome or that due to lupus erythematosus.

Respiratory diseases: Berylliosis, fulminating or disseminated pulmonary tuberculosis when used concurrently with appropriate antituberculous chemotherapy, idiopathic eosinophilic pneumonias, symptomatic sarcoidosis.

Rheumatic disorders: As adjunctive therapy for short-term administration (to tide the patient over an acute episode or exacerbation) in acute gouty arthritis; acute rheumatic carditis; ankylosing spondylitis; psoriatic arthritis; rheumatoid arthritis, including juvenile rheumatoid arthritis (selected cases may require low-dose maintenance therapy). For the treatment of dermatomyositis, temporal arteritis, polymyositis, and systemic lupus erythematosus.

6.3 Contraindications

The methylprednisolone sodium succinate is contraindicated:

- in systemic fungal infections and patients with known hypersensitivity to the product and its constituents.
- for intrathecal administration. Reports of severe medical events have been associated with this route of administration.

6.4 Warning

Serious Neurologic Adverse Reactions with Epidural Administration

Serious neurologic events, some resulting in death, have been reported with epidural injection of corticosteroids. Specific events reported include, but are not limited to, spinal cord infarction, paraplegia, quadriplegia, cortical blindness, and stroke. These serious neurologic events have been reported with and without use of fluoroscopy. The safety and effectiveness of epidural administration of corticosteroids have not been established, and corticosteroids are not approved for this use.

GENERAL

Increased dosage of rapidly acting corticosteroids is indicated in patients on corticosteroid therapy who are subjected to any unusual stress before, during, and after the stressful situation.

Results from one multicenter, randomized, placebo-controlled study with methylprednisolone hemisuccinate, an intravenous corticosteroid, showed an increase in early (at 2 weeks) and late (at 6 months) mortality in patients with cranial trauma

who were determined not to have other clear indications for corticosteroid treatment. High doses of systemic corticosteroids, should not be used for the treatment of traumatic brain injury.

Cardio-renal

Average and large doses of corticosteroids can cause elevation of blood pressure, salt and water retention, and increased excretion of potassium. These effects are less likely to occur with the synthetic derivatives except when used in large doses. Dietary salt restriction and potassium supplementation may be necessary. All corticosteroids increase calcium excretion. Literature reports suggest an apparent association between the use of corticosteroids and left ventricular free wall rupture after a recent myocardial infarction; therefore, therapy with corticosteroids should be used with great caution in these patients.

Endocrine

Hypothalamic-pituitary adrenal (HPA) axis suppression, Cushing's syndrome, and hyperglycemia. Monitor patients for these conditions with chronic use. Corticosteroids can produce reversible HPA axis suppression with the potential for glucocorticosteroid insufficiency after withdrawal of treatment. Drug induced secondary adrenocortical insufficiency may be minimized by gradual reduction of dosage. This type of relative insufficiency may persist for months after discontinuation of therapy; therefore, in any situation of stress occurring during that period, hormone therapy should be reinstituted.

Drug-Induced Liver Injury

Rarely, high doses of cyclically pulsed intravenous methylprednisolone (usually for the treatment of exacerbations of multiple sclerosis at doses of 1 gram/day) can induce a toxic form of acute hepatitis. The time to onset of this form of steroid-induced liver injury can be several weeks or longer. Resolution has been observed after discontinuation of treatment. However, serious liver injury can occur, sometimes resulting in acute liver failure and death. Discontinue intravenous methylprednisolone if toxic hepatitis occurs. Since recurrence has occurred after re-challenge, avoid use of high dose intravenous methylprednisolone in patients with a history of toxic hepatitis caused by methylprednisolone.

Infections

General

Patients who are on corticosteroids are more susceptible to infections than are healthy individuals. There may be decreased resistance and inability to localize infection when corticosteroids are used. Infections with any pathogen (viral, bacterial, fungal, protozoan, or helminthic) in any location of the body may be associated with the use of corticosteroids alone or in combination with other immunosuppressive agents. These infections may be mild, but can be severe and at times fatal. With increasing doses of corticosteroids, the rate of occurrence of infectious complications increases. Corticosteroids may also mask some signs of current infection. Do not use intraarticularly, intrabursally or for intratendinous administration for local effect in the presence of acute local infection. A study has failed to establish the efficacy of methylprednisolone sodium succinate in the treatment of sepsis syndrome and septic

shock. The study also suggests that treatment of these conditions with methylprednisolone sodium succinate may increase the risk of mortality in certain patients (i.e., patients with elevated serum creatinine levels or patients who develop secondary infections after methylprednisolone sodium succinate).

Fungal infections

Corticosteroids may exacerbate systemic fungal infections and therefore should not be used in the presence of such infections unless they are needed to control drug reactions. There have been cases reported in which concomitant use of amphotericin B and hydrocortisone was followed by cardiac enlargement and congestive heart failure (see CONTRAINDICATIONS and PRECAUTIONS, Drug Interactions, Amphotericin B injection and potassium-depleting agents). Special pathogens Latent disease may be activated or there may be an exacerbation of intercurrent infections due to pathogens, including those caused by Amoeba, Candida, Cryptococcus, Mycobacterium, Nocardia, Pneumocystis, Toxoplasma. It is recommended that latent amebiasis or active amebiasis be ruled out before initiating corticosteroid therapy in any patient who has spent time in the tropics or in any patient with unexplained diarrhea. Similarly, corticosteroids should be used with great care in patients with known or suspected Strongyloides (threadworm) infestation. In such patients, corticosteroid-induced immunosuppression may lead to Strongyloides hyperinfection and dissemination with widespread larval migration, often accompanied by severe enterocolitis and potentially fatal gram-negative septicemia. Corticosteroids should not be used in cerebral malaria. There is currently no evidence of benefit from

steroids in this condition.

Tuberculosis

The use of corticosteroids in active tuberculosis should be restricted to those cases of fulminating or disseminated tuberculosis in which the corticosteroid is used for the management of the disease in conjunction with appropriate antituberculous regimen. If corticosteroids are indicated in patients with latent tuberculosis or tuberculin reactivity, close observation is necessary as reactivation of the disease may occur. During prolonged corticosteroid therapy, these patients should receive chemoprophylaxis.

Vaccination

Administration of live or live, attenuated vaccines is contraindicated in patients receiving immunosuppressive doses of corticosteroids. Killed or inactivated vaccines may be administered. However, the response to such vaccines cannot be predicted. Immunization procedures may be undertaken in patients receiving corticosteroids as replacement therapy, e.g., for Addison’s disease.

Viral infections

Chicken pox and measles can have a more serious or even fatal course in pediatric and adult patients on corticosteroids. In pediatric and adult patients who have not had these diseases, particular care should be taken to avoid exposure. The contribution of the underlying disease and/or prior corticosteroid treatment to the risk is also not known. If exposed to chicken pox, prophylaxis with varicella zoster immune globulin (VZIG) may be indicated. If exposed to measles, prophylaxis with immunoglobulin

(IG) may be indicated. (See the respective package inserts for complete VZIG and IG prescribing information.) If chicken pox develops, treatment with antiviral agents should be considered.

Neurologic

Reports of severe medical events have been associated with the intrathecal route of administration (see ADVERSE REACTIONS, Gastrointestinal and Neurologic/Psychiatric).

Ophthalmic

Use of corticosteroids may produce posterior subcapsular cataracts, glaucoma with possible damage to the optic nerves, and may enhance the establishment of secondary ocular infections due to bacteria, fungi, or viruses. The use of oral corticosteroids is not recommended in the treatment of optineuritis and may lead to an increase in the risk of new episodes. Corticosteroids should be used cautiously in patients with ocular herpes simplex because of corneal perforation. Corticosteroids should not be used in active ocular herpes simplex.

PRECAUTIONS

General

This product, like many other steroid formulations, is sensitive to heat. Therefore, it should not be autoclaved when it is desirable to sterilize the exterior of the vial. The lowest possible dose of corticosteroid should be used to control the condition under treatment. When reduction in dosage is possible, the reduction should be gradual. Since complications of treatment with glucocorticoids are dependent on the size of the

dose and the duration of treatment, a risk/benefit decision must be made in each individual case as to dose and duration of treatment and as to whether daily or intermittent therapy should be used. Kaposi's sarcoma has been reported to occur in patients receiving corticosteroid therapy, most often for chronic conditions. Discontinuation of corticosteroids may result in clinical improvement.

Cardio-renal

As sodium retention with resultant edema and potassium loss may occur in patients receiving corticosteroids, these agents should be used with caution in patients with congestive heart failure, hypertension, or renal insufficiency.

Endocrine

Drug-induced secondary adrenocortical insufficiency may be minimized by gradual reduction of dosage. This type of relative insufficiency may persist for months after discontinuation of therapy; therefore, in any situation of stress occurring during that period, hormone therapy should be reinstituted. Metabolic clearance of corticosteroids is decreased in hypothyroid patients and increased in hyperthyroid patients. Changes in thyroid status of the patient may necessitate adjustment in dosage.

Gastrointestinal

Steroids should be used with caution in active or latent peptic ulcers, diverticulitis, fresh intestinal anastomoses, and nonspecific ulcerative colitis, since they may increase the risk of a perforation. Signs of peritoneal irritation following gastrointestinal perforation in patients receiving corticosteroids may be minimal or absent. There is an enhanced effect due to decreased metabolism of corticosteroids in

patients with cirrhosis.

Musculoskeletal

Corticosteroids decrease bone formation and increase bone resorption both through their effect on calcium regulation (i.e., decreasing absorption and increasing excretion) and inhibition of osteoblast function. This, together with a decrease in the protein matrix of the bone secondary to an increase in protein catabolism, and reduced sex hormone production, may lead to inhibition of bone growth in pediatric patients and the development of osteoporosis at any age. Special consideration should be given to patients at increased risk of osteoporosis (i.e., postmenopausal women) before initiating corticosteroid therapy. Local injection of a steroid into a previously infected site is not usually recommended.

Neurologic-Psychiatric

Although controlled clinical trials have shown corticosteroids to be effective in speeding the resolution of acute exacerbations of multiple sclerosis, they do not show that corticosteroids affect the ultimate outcome or natural history of the disease. The studies do show that relatively high doses of corticosteroids are necessary to demonstrate a significant effect. An acute myopathy has been observed with the use of high doses of corticosteroids, most often occurring in patients with disorders of neuromuscular transmission (e.g., myasthenia gravis), or in patients receiving concomitant therapy with neuromuscular blocking drugs (e.g., pancuronium). This acute myopathy is generalized, may involve ocular and respiratory.

7. Organizational Structure

7.1 Funding

MARVEL trial is an investigator-initiated study which is organized by the second affiliated hospital of the Third Military Medical University and will conduct in about 80 centres in China. MARVEL will be funded by the National Natural Science Foundation of China. Methylprednisolone and its placebo are manufactured and provided by Chongqing Lummy Pharmaceutical Co., Ltd., Chongqing, China. The funder had no involvement in the study design, data collection, analysis and interpretation, writing or decision to submit the paper.

7.2 Trials Boards and Committees

The following boards and institutions will assure the success of the clinical trial:

7.2.1 Steering Committee

The Steering Committee of the clinical trial will decide on the final protocol and oversee the trial.

7.2.2 Data Safety and Monitoring Board

The independent Date and Safety Monitoring Board (DSMB) will regularly monitor the safety of the trial and ensure the safety of patients at all stages of MARVEL. The independent DSMB will be composed of an experienced neurologist, a neuroradiologist, and a biostatistician, who are not participants of the MARVEL consortium and not involved in the clinical trial in any other way. The DSMB may at

any time propose to revise the clinical trial protocol or terminate the trial in case of safety concerns.

7.2.3 Ethics Advisory Board

The Ethics Advisory Board (EAB) will supervise the trial and ensure that the trial is conducted in accordance with the ICH-GCP guidelines and international and China legislation. The EAB may at any time propose to revise the clinical trial protocol or terminate the trial in case of ethical concerns.

7.2.4 Imaging Core Laboratory

Centralized imaging core laboratories will centrally blinded review all images and provide reference judgements for the definition of the per protocol population and intracranial haemorrhages. CT/MR and angiographic images will be independently reviewed by two independent central imaging core laboratories respectively. Imaging Core Laboratory will be independent to ensure it is blinded to the treatment allocation.

7.2.5 Clinical Events Committee

The Clinical Events Committee will be comprised of three expert physicians independent of the investigational sites. This committee will validate all the complications that occur over the course of the study and categorize each for severity and relatedness according to the definition in the AE section. The Clinical Events Committee can request any additional source information and images supporting the AEs to assist with the adjudication.

8. Design

MARVEL is a multicenter, randomized, double-blind, placebo-controlled trial. Patients will be randomized 1:1 to either Methylprednisolone Group or Placebo Group. The study has been registered at Chinese Clinical Trial Registry (www.chictr.org.cn, unique identifier ChiCTR2100051729).

9. Population

The patients for this trial will be recruited from AIS patients with large vessel occlusion within 24 hours from last known well.

9.1 Inclusion criteria

- Age >= 18 years;
- The time from onset to randomization was within 24 hours;
- Anterior circulation ischemic stroke was preliminarily determined according to clinical symptoms or imaging examination;
- Baseline National Institutes of Health Stroke Scale (NIHSS) ≥ 6
- Baseline Alberta Stroke Program Early CT Score (ASPECTS)>= 5;
- Computed tomography angiography (CTA) /magnetic resonance angiography (MRA) /digital subtraction angiography (DSA) confirmed occlusion of intracranial segment of internal carotid artery and middle cerebral artery, and decided to undergo endovascular therapy;

- Written informed consent signed by patients or their family members.

9.2 Exclusion Criteria

Patients meeting any of the following criteria will be excluded from study enrolment.

- Intracranial hemorrhage confirmed by cranial computed tomography (CT) or magnetic resonance imaging (MRI);
- mRS score >= 2 before onset;
- Pregnant or lactating women;
- Allergic to contrast agents;
- Allergic to glucocorticoids;
- Participating in other clinical trials;
- Systolic blood pressure > 185 mmHg or diastolic pressure > 110 mmHg, and oral antihypertensive drugs can not control;
- Genetic or acquired bleeding constitution, lack of anticoagulant factors; Or oral anticoagulants and INR > 1.7;
- Blood sugar < 2.8 mmol/L (50 mg/dl) or > 22.2 mmol/L (400 mg/dl), platelet < 90 x 10^9/L;
- The artery is tortuous so that the thrombectomy device cannot reach the target vessel;
- Bleeding history (gastrointestinal and urinary tract bleeding) in recent 1 month;

- Chronic hemodialysis and severe renal insufficiency (glomerular filtration rate < 30 ml/min or serum creatinine > 220 umol/L [2.5 mg/ dL]);
- Life expectancy due to any advanced disease < 6 months;
- Follow-up is not expected to be completed;
- Intracranial aneurysm and arteriovenous malformation;
- Brain tumors with imaging mass effect;
- Severe systemic infectious diseases.

9.3 Sample Size

The current trial is designed detect a shift on the modified Rankin Scale score, which represents the global disability, to a lower score. The expected distribution of the modified Rankin Scale score, derived from the EVT for acute anterior circulation ischemic stroke (ACTUAL) registry[38] and the Highly Effective Reperfusion evaluated in Multiple Endovascular Stroke Trials (HERMES) collaboration[7], is as follows: 7%, 20%, 16%, 11%, 13%, 8% and 25%.

The sample size was calculated using the cumulative proportion of patients with mRS 0-2. We assume a moderate effect of 7% absolute increase in the cumulative proportion of patients with mRS 0-2 in the intervention group, compared with controls, indicating an odds ratio (OR) of 1.33, which have substantially exceeds the minimal clinically important difference.[39,40] In order to demonstrate the expected

treatment effect with a type-1 error alpha =0.05 (two-tailed) and a power of 80% (beta = 20%). A sample size of n= 1588 patients (n=794 per treatment group) is required.

The intention-to-treat principle will be applied to the primary analysis and, therefore, to safeguard against dilution of the treatment effect associated with an approximate 5% non-adherence rate (due to loss to follow-up, consent withdrawal and other reasons), we initially planned to enrol n=1672 patients (n=836 per treatment group) for this study. This estimation was performed based on PASS (NCSS, LLC. Kaysville, Utah, USA) version 15.0.

10. Imaging Training

Investigators involved in image reading within the trial will participate in a standardized image reading training before study start.

11. Randomization

Randomization will be done by a web-based APP (Jinlingshu) on mobile phone or computer (https://jinlingshu.com/). The automated system will assign an appropriate set of study medication to each patient. Eligible patients are randomized to treatment with either intravenous methylprednisolone or placebo with a ratio of 1:1. Randomization will be stratified by participating centre with permutation block size of 4. Randomization will be completely concealed by having both web-based real-time allocation and identical appearance of methylprednisolone sodium succinate and placebo bottles. (All bottles will have a unique number. Subjects will be assigned a

random serial number according to the time they were enrolled, and corresponding masked medications will be provided).

The randomization list will be prepared by the independent statistical centre using SAS 9.4. It will only be sent to the data coordination centre responsible for central randomization and data management, the pharmacy centre responsible for treatment packaging.

12. Blinding/Unblinding

Both patient and investigator are blinded to treatment assignment. If there is a clinical situation that the investigator believes it is necessary to unblind for the safety of patients, the medical monitor must be immediately notified to discuss the intended unblinding. Premature breaking of the blind should be restricted to the setting where identification of the type of treatment is critical for adequate treatment of the patient. All unblinded patients will remain in the study and complete all follow-up visits.

If the site principal investigator wants to know the identity of the study drug for any other reason, he or she must call the medical monitor. Unless the circumstances are deemed necessary by site principal investigator, treatment codes should not be broken. The site principal investigator should document and make an explanation for any premature unblinding.

For the purpose of regulatory reporting, if required by the local health authorities, treatment codes could be broken for all serious, unexpected suspected adverse reactions that are deemed by the investigator to be related to study drug.

13. Study Treatment

13.1 Formulation, Packaging, and Handling

Methylprednisolone sodium succinate and its placebo are manufactured by Lummy Pharmaceutical Group Co., Ltd., Chongqing, China. All study medication will be manufactured, tested, released, and shipped according to Good Manufacturing Practice guidelines. Labelling and packaging of study medication will be conducted according to Good Clinical Practice and Good Manufacturing Practice guidelines, and any national regulatory requirements.

Methylprednisolone sodium succinate and its corresponding placebos are provided in numbered and are visually identical, except for a unique number. Methylprednisolone sodium succinate and it's placebo will be packed in glass bottle of identical appearance. Each kit has a unique identification number and will be stored in a safe location at room temperature on the clinical site with limited access.

13.2 Test Product

Each kit of the methylprednisolone group contains l2 bottles, and each bottles contains 40mg methylprednisolone sodium succinate, labelled for "Study Drug" and "clinical trial use only"). Each kit will be stored in a safe location at room temperature (25°C with excursions permitted between 15°C−30°C) on the clinical site with restricted access.

13.3 Comparator

Each kit of the placebo group contains l2 bottles, and each bottles contains 40mg placebo, labelled for "Study Drug" and "clinical trial use only"). Each kit will be stored in a safe location at room temperature (25°C with excursions permitted between 15°C−30°C) on the clinical site with restricted access.

13.4 Dosage, Administration, and Compliance

Eligible patients will be randomly assigned a number corresponding to a blinded sealed medication kit that sent to each patient. It is recommended to start using the study drug within 15 minutes after randomization.

13.4.1 Methylprednisolone and its Placebo

Patients will be assigned to receive either a placebo or methylprednisolone, with a dosage of 2mg/kg (based on estimated or actual weight if known, not exceeding a maximum dose of 160 mg) per day for a duration of 3 days. The initial study drug will be administered as soon as possible after randomisation. It is recommended that the initial study drug administrated before arterial access closure, but it should not be delayed more than 2 hours after arterial access closure. All patients will be treated according to the current Chinese Stroke Association guidelines for clinical management of cerebrovascular disorders[41]. Risk factors such as diabetes mellitus, hypertension, hyperlipidaemia, hyperhomocysteinaemia, obesity, drinking and smoking should be managed appropriately.

13.4.2 Concomitant therapy

Administration of any other (intravenous or oral) corticosteroids (E.g.,

Hydrocortisone) is not allowed during the first 72 hours post randomization. Gastrointestinal prophylaxis and hyperglycemia treatment will be administered per standard local protocols. Any concomitant medication within one week prior to screening through the study completion/discontinuation visit should be documented in the Concomitant Medications Case Report Form.

13.4.4 Drug Accountability

The investigational sites will be provided with sufficient amounts of investigational medicinal products. The investigational medicinal products must not be used outside the study protocol. The investigator or authorized staff is obliged to acknowledge receipt of the study medication to confirm the content and shipping temperature. Any damaged shipment of study medications will be replaced. The study drug will be disposed of in accordance with the standard operating procedures at the study site, or returned to the sponsor with appropriate documents. The investigational site's method of study medication destruction must be agreed to by the Sponsor.

Before destroying any study medications, the investigational site must obtain the written authorization from the sponsor and document the destruction of study medications in an appropriate form. Accurate records of receipt, dispensation, return and disposition of all study medication during the study should be documented on the drug inventory log.

14. Study Schedule

The schedule of assessments conducted during the study is shown in Part 2 of Chapter

2. Flow Chart and Schedule of Assessments. The study includes three periods: Screening (V0), treatment (V1, V2) and Follow up (V3, V4).

14.1 Screening period (Visit V0, Randomization)

14.1.1 Visit V0

For each eligible patient, written informed consent for participation in the study will be obtained from the patient or the patient's legal representative prior to the performance of any protocol-related investigation. Once written informed consent is obtained, the results of the following standard of care assessments will be used for the study. The investigator will record the details of all patients consented to confirm eligibility and record the reasons for screening failure.

- Demographics
- Medical history
- Physical examination
- Determination of the pre-stroke mRS via interview of the patient or the patient's kin
- NIHSS score (Note: performed by an NIHSS-certified practitioner)
- Collection of information on previous medications
- Measurement of blood pressure and heart rate
- Pregnancy test (urine test, in non-menopausal women)

- Laboratory tests
- A 12-lead ECG
- Non-contrast CT scan or MRI scan and CTA, MRA, or DSA
- Concomitant medications
- Checking of inclusion and exclusion criteria

14.1.2 Randomization (R)

If all inclusion criteria are met and no exclusion criteria are present, randomization will be performed.

14.2 Treatment period (Visits V1，V2)

- After randomization, the treatment period starts, including study medication (V1) and post-treatment clinical examination (V2).

14.2.1 Visit 1

Before administration of the study medication, measurement of blood pressure and heart rate have to be performed. The study medication will be administered as specified for intravenous methylprednisolone. During the administration of study medication, the following assessments will be made:

- Measurement of blood pressure and heart rate every hour.
- Documentation of any concomitant medication.
- Assessment of Adverse Event.

- In any case of significant neurological deterioration judged by the investigator, an assessment of the neurological deficit and another non-contrast cranial CT scan or MRI scan have to be performed to determine whether intracranial haemorrhage occurs.
- The 48h monitoring should be performed in Stroke Unit or equivalent unit.

14.2.2 Visit 2

- Visit 2 comprises the clinical examination and a second non-contrast cranial CT or MRI scan which is performed to diagnose intracranial haemorrhage. Visit 2 will be done 48-96h after the administration of study medication. The following assessments will be performed:
- Physical examination
- Assessment of the neurological deficit by the NIHSS
- Measurement of blood pressure and heart rate
- Laboratory tests
- Non-contrast cranial CT or MRI scan
- Documentation of any concomitant medication
- Assessment of Adverse Event

14.3 Follow Up period (Visits V3, V4)

The Follow Up period comprises the period of the subacute stage (day 5-7 or hospital

discharge) in Visit 3， and the final follow up examination 90±7 days in Visit 4.

14.3.1 Visit 3

The following assessments will be performed:

- Physical examination
- Assessment of the neurological deficit by the NIHSS
- Measurement of blood pressure and heart rate
- Documentation of any concomitant medication
- Assessment of Adverse Event

14.3.3 Visit 4

The following assessments will be performed:

- Physical examination (If the patients return to local hospital)
- Assessment of the neurological deficit by the NIHSS
- Assessment of functional status by the mRS
- Assessment of functional health status and quality of life by the EQ-5D scale
- Documentation of any concomitant medication
- Assessment of Adverse Event

15. Endpoints of the Clinical Trial

15.1 Efficacy Analysis-Primary Endpoint

The primary outcome was the ordinal mRS score, a global measure of disability, comprises of seven grades ranging from 0 (no symptoms) to 6 (death).

15.2 Efficacy Analysis-Secondary Endpoints

- Proportion of patients with mRS score 0 to 4 at 90±7 days;
- Proportion of patients with mRS score 0 to 3 at 90±7 days;
- Proportion of patients with mRS score 0 to 2 at 90±7 days
- Proportion of patients with mRS score 0 to 1(or returned to pre-stroke modified Rankin scale score) at 90±7 days
- NIHSS score at 5-7 days after EVT or at early discharge;
- European Quality Five-Dimension scale score at 90 days.

15.3 Safety Analysis-Primary Endpoint

15.3.1 Primary Safety Endpoints

- Mortality due to any cause at 90 (±7) days;
- Proportion of patients with symptomatic intracranial haemorrhage within 48 hours after endovascular treatmen. Symptomatic intracranial haemorrhage will be adjudicated by an independent Imaging Core Laboratory and the Clinical Events Committee according to the modified Heidelberg Bleeding Classification, and is diagnosed based on a combination of: 1) clinical deterioration, 2) imaging findings, and 3) causal relatedness assessment. Clinical deterioration is defined as any of the following conditions: 1) NIHSS score increased more than 4 points than that

immediately before worsening; 2) NIHSS score increased more than 2 points in one category; 3) Deterioration led to intubation, hemicraniectomy, external ventricular drain placement or any other major interventions. The imaging criteria are presence of parenchymal hematoma type 2 (PH2), parenchymal hematoma type 1 (PH1), remote intracerebral haemorrhage (RIH), subarachnoid haemorrhage (SAH), intraventricular haemorrhage (IVH), or subdural haemorrhage. When clinical deterioration and PH2 co-occur, the event is automatically categorized as SICH. When clinical deterioration and PH1/RIH/SAH/IVH/SDH co-occur, the event is deemed SICH if it is judged that the haemorrhage contributed substantially to the clinical worsening. When clinical deterioration occurs only with haemorrhagic infarction type 1 or 2 (HI1 or HI2), the event is automatically classified as not SICH. ICH.

15.3.2 Secondary Safety Endpoints

- Proportion of patients with any radiologic intracranial haemorrhage within 48 hours after treatment;
- Proportion of patients with pneumonia;
- Proportion of patients with gastrointestinal haemorrhage within 7 days after EVT;
- Incidence of serious adverse events.
- Incidence of any adverse events.

16. Assessment

16.1 Assessment of efficacy

16.1.1 The modified Rankin Scale (mRS)

The mRS score is a valid and reliable clinician-reported measure of global disability that has been widely applied for evaluating recovery from stroke. It is a scale used to measure functional recovery (the degree of disability or dependence in daily activities) of people who have suffered a stroke[42,43]. mRS scores range from 0 to 6, with 0 indicating no residual symptoms; 5 indicating bedbound, requiring constant care; and 6 indicating death. The mRS score will be obtained at

Day 90. Premorbid mRS status will also be obtained retrospectively and reported on the 24 Hours CRF page. In this trial, we will keep video and voice recording versions of mRS score except those who die, unable, or refuse to take a video. For mRS score at Day 90, the mRS score will be assessed by two independent certified neurologists in a blinded manner with the video combined with voice record. For those who decline to participate in a video recording, the outcomes will be determined in person by site neurologists blinded to the treatment assignment, and keep a chat record with site neurologists. Disagreements are resolved by consensus.

The mRS will only be scored by those who have been trained and certified to use this scale using the table below. (See appendix 1)

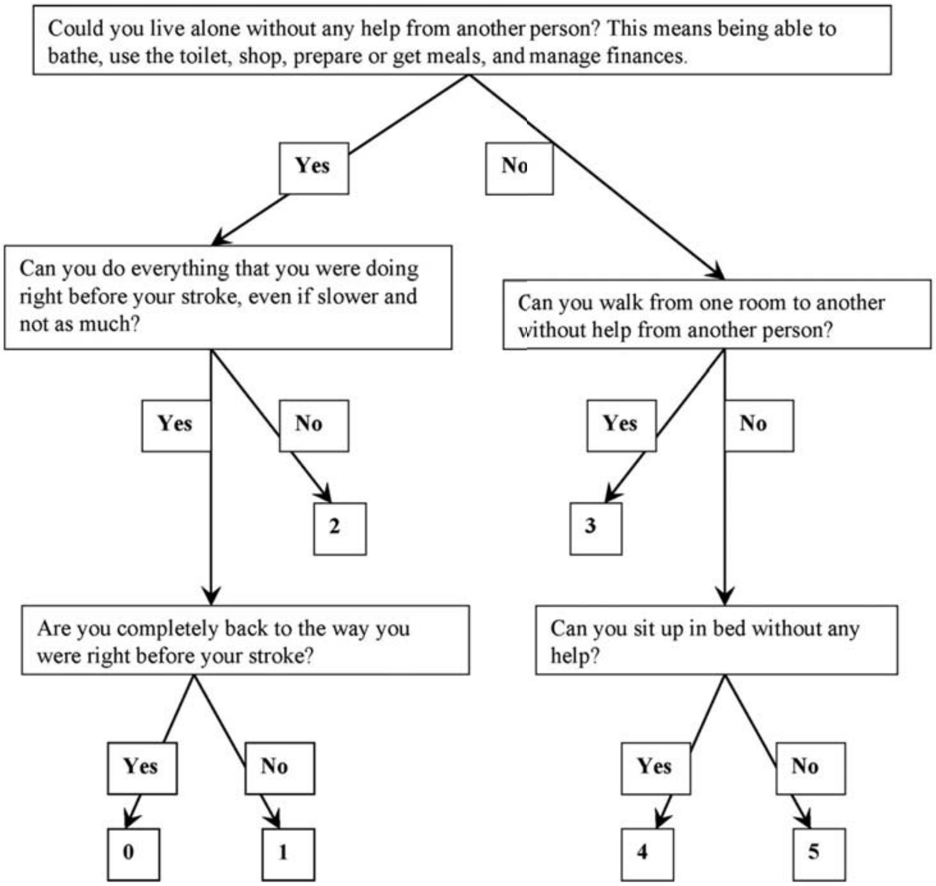

16.1.2 The National Institutes of Health Stroke Scale (NIHSS)

The NIHSS is a standardized neurological examination score that is a valid and reliable measure of disability and recovery after acute stroke[44]. Scores range from 0 to 42, with higher scores indicating more severe disability. The scale includes measures of level of consciousness, extra ocular movements, motor and sensory tests, coordination, language and speech evaluations. The NIHSS will be administered at baseline, Day 5-7 or discharge if earlier. The NIHSS will only be scored by those trained and certified in the use of this scale. In this trial, we will keep a video version

of NIHSS score except in those who die or are unable or refuse to take a video. The NIHSS score will be centrally assessed by two independent certified neurologists in a blinded manner via the video. Disagreements are resolved by consensus.(See appendix 2)

16.1.3 European Quality Five Dimensions Five Level scale

The EQ-5D-VAS is a generic instrument for describing and valuing health. The instrument is designed for self-completion, and respondents also rate their overall health on the day of the interview on a 0-100 hash-marked, vertical visual analogue scale. Patients who died will have a score of 0. The EQ-5D-VAS will be administered on Day 90±7 by those trained in the use of this scale. (See appendix 3)

16.2 Assessment of safety

16.2.1 Mortality at 90±7 days

Mortality at 90±7 days is one of the most important safety endpoints of the MARVEL trial. Mortality rates are defined as the number of deaths observed divided by the number of subjects observed over the 90-day study period.

16.2.2 Symptomatic Intracranial Haemorrhage

In addition to mortality, we will investigate SICH being the most feared complication for acute ischemic stroke. SICH within 48 hours will be adjudicated by an independent Imaging Core Laboratory and the Clinical Events Committee according to the Heidelberg Bleeding Classification.[45] SICH is diagnosed based on a

combination of: 1) clinical deterioration, 2) imaging findings, and 3) causal relatedness assessment. Clinical deterioration is defined as any of the following conditions: 1) NIHSS score increased more than 4 points than that immediately before worsening; 2) NIHSS score increased more than 2 points in one category; 3) Deterioration led to intubation, hemicraniectomy, external ventricular drain placement or any other major interventions. The imaging criteria are presence of parenchymal hematoma type 2 (PH2), parenchymal hematoma type 1 (PH1), remote intracerebral haemorrhage (RIH), subarachnoid haemorrhage (SAH), intraventricular haemorrhage (IVH), or subdural haemorrhage (SDH). When clinical deterioration and PH2 co-occur, the event is automatically categorized as SICH. When clinical deterioration and PH1/RIH/SAH/IVH/SDH co-occur, the event is deemed SICH if it is judged that the haemorrhage contributed substantially to the clinical worsening. When clinical deterioration occurs only with haemorrhagic infarction type 1 or 2 (HI1 or HI2), the event is automatically classified as not SICH.

16.2.3 Any ICH within 48 hours

The statistical analysis of differences in proportions of patients with any radiologic ICH within 48 hours between Methylprednisolone and Placebo groups will be performed.

The central reading will classify ICH according to the following intracerebral subtypes:

- Haemorrhagic infarct type 1 or type 2;

- Parenchymal hematoma type 1 or type 2;
- Remote intraparenchymal haemorrhage;
- In addition, intraventricular haemorrhage, subarachnoid haemorrhage, subdural haemorrhage, or epidural haemorrhage will be noted. ICH subtypes will be tabulated by treatment group.

16.2.4 Proportion of patients with pneumonia

Pneumonia is considered when at least 2 signs (body temperature > 38°C; leukocytosis >12 000/mL, or leukopenia <4000/mL; purulent pulmonary secretions) associated with the appearance of a new infiltrate are present or when changes occur in an existing infiltrate on chest x-ray. If necessary, the diagnosis will be confirmed by a respiratory tract sample using a quantitative culture with a predefined positive threshold of 10^4 colony-forming units per milliliter (CFU/mL) for a bronchoalveolar lavage or non bronchoscopic sample, of 10^3 CFU/mL for a protected specimen brush and of 10^6 CFU/mL for a tracheal sample. The incidence of pneumonia will be reported by local investigators.

16.2.4 Proportion of patients with gastrointestinal bleeding.

The statistical analysis of differences in proportions of patients with gastrointestinal bleeding within 7 days between Methylprednisolone and Placebo groups will be performed. Gastrointestinal bleeding will be reported by local investigators.

16.2.5 Adverse events

The safety assessments will include monitoring and recording adverse events, including serious and non-serious adverse events, measuring vital signs specified in the protocol, and other protocol specified tests that are considered to be critical to the safety evaluation of the study.

Any adverse change in health or the appearance of or worsening of any undesirable sign, symptom or medical condition occurring after enrolment into the trial will be recorded as Adverse Event whether or not it is considered to be related to the study drug. An adverse event also includes any occurrence that is a new illness; aggravated in severity of frequency from the baseline condition, abnormal results of diagnostic procedures, or a combination of the above. The assessment of severity and relationship of adverse events are determined by using the following definitions.

Table 3: Severity categorization and relationship definitions of Adverse Event.

Severity categorization		
Grade1	Mild	It is usually transient and may require only minimal treatment or therapeutic intervention. The event doesn’t generally interfere with usual activities of daily living.
Grade2	Moderate	It is usually alleviated with additional specific therapeutic intervention. The event interferes with usual activities of daily living, causing discomfort but poses no significant or permanent risk of harm to the research subject.
Grade3	Severe	It interrupts usual activities of daily living, or significantly affects clinical status, or may require intensive therapeutic intervention.

Grade4	Life-threatening	Substantial risk of dying at time of event
Grade5	Death	Death
Relationship of adverse events		
Related	A clinical event, including laboratory test abnormality, where there is a “reasonable possibility” that the serious adverse event was caused by the study medication, meaning that there is evidence or arguments to suggest a causal relationship.	
Probably	A clinical event, including laboratory test abnormality, with a reasonable time sequence to drug administration, unlikely to be attributed to concurrent disease or other drugs or chemicals, and which follows a clinically reasonable response on withdrawal.	
Possibly	A clinical event, including laboratory test abnormality, with a reasonable time sequence to drug administration, but which could also be explained by concurrent disease or other drugs or chemicals. Information on drug withdrawal may be lacking or unclear.	
Unrelated	This category is applicable to adverse events which are judged to be clearly and incontrovertibly due to extraneous causes (diseases, environment, etc.) and do not meet the criteria for drug relationship listed for the above-mentioned conditions.	

16.2.6 Serious Adverse Event

A Serious Adverse Event is any untoward medical occurrence (whether deemed to be related to investigational medication or not) that meets any of the following criteria:

- Fatal (i.e., the adverse event actually causes or leads to death)
- Life-threatening (i.e., the adverse event, in the view of the investigator, places the patient at immediate risk of death). This does not include any adverse event that, had it occurred in a more severe form or was allowed to continue, might have caused death.
- Requires or prolongs inpatient hospitalization
- Results in persistent or significant disability/incapacity (i.e., the adverse event results in substantial disruption of the patient's ability to conduct normal life functions)
- Congenital anomaly/birth defect in a neonate/infant born to a mother exposed to study drug
- Significant medical event in the investigator's judgment (e.g., may jeopardize the patient or may require medical/surgical intervention to prevent one of the outcomes listed above)

16.2.7 Adverse Event Recording and Follow Up

The investigator is responsible for evaluating and reporting any adverse events occurring during the study. The investigator will record onset, duration, intensity, any taken action, evolution/outcome, and the causality assessment for any adverse event.

Any adverse events leading to withdrawal from the study and those that persist at the end of the study must be followed up.

16.2.8 Serious Adverse Events Reporting

Although adverse events are recorded in the adverse event form of CRF special reporting requirements apply to the reporting of serious adverse events. Serious adverse events need to be reported immediately. The investigator should immediately notify the principal investigator of any serious adverse events that occurred at the trial site within 24 hours: MARVEL security desk Fax: +86 023-68774270

Serious adverse event reports will be collected centrally at the safety desk. Copies of all serious adverse event reports will be sent to the central trial management. Such preliminary reports will be followed by detailed descriptions, including anonymous copies of hospital case reports or related results, as well as other documents (if required and applicable). All serious adverse events that have not been resolved by the end of the study, or that have not been resolved upon discontinuation of the subject's participation in the study, must be followed until any of the following occurs:

- the event resolves
- the event stabilizes
- the event returns to baseline, if a baseline value is available
- the event can be attributed to agents other than the study drug or to factors unrelated to study conduct

- when it becomes unlikely that any additional information can be obtained

Any additional information known after the event has been initially reported should be sent using a new serious adverse event report form. New information will be noted on the "serious adverse event" form, by ticking the box marked "follow-up" and sending to MARVEL SAFETY DESK.

Patients who withdraw from the study treatment due to any adverse event will be followed up at least until the outcomes are determined, even if it implies that the follow-up continues after the patient has left the trial.

The investigator should assess the seriousness of the adverse events. This is based on the regulatory definitions of seriousness. The investigator should assess the causal relationship of serious adverse events. This is a clinical assessment of whether the adverse events may be related to the investigational medication. The evaluation of the expectedness is based on knowledge of adverse reactions and references.

17. Study Discontinuation Criteria

17.1 Patient Discontinuation

Patients have the right to voluntarily withdraw from the study at any time for any reason. In addition, the investigator has the right to withdraw the patient from the study at any time. Reasons for withdrawal from the study may include, but are not limited to the following:

- Patient withdraws consent at any time

- Any medical condition determined by the investigator or sponsor may endanger the patient's safety if he or she continues the study
- The investigator or sponsor determines that it is in the best interests of the patient
- Patient noncompliance, specifically defined as unwillingness to participate in a 3-month assessment of neurological status and function

Every effort should be made to obtain information about patients who withdrew from the study. The main reasons for withdrawal from the study should be recorded on the corresponding CRF. Patients who withdraw from the study will not be replaced.

17.2 Study Treatment Discontinuation

The treatment with the investigate medication should be terminated immediately if the patients experience any of the following:

- Any serious gastrointestinal bleeding.
- Uncontrollable hyperglycaemia (blood sugar >22.2 mmol/L)
- Anaphylactic reaction
- Continuation of the study drug would be detrimental to the patient’s well-being.
- Withdrawal for personal reasons.

According to the intention to treat (ITT) principle, patients who discontinue study treatment prematurely will continue in the study and be followed up to the 90th day.

17.3 Study and Site Discontinuation

The investigators has the right to terminate this study in any case of concern about the safety of patients caused by new information. The reasons for terminating the study may include, but are not limited to:

- The incidence or severity of adverse events in this or other studies indicates a potential health hazard to patients.
- Patient enrolment is unsatisfactory

The investigators have the right to close a site at any time. Reasons for closing a site may include, but are not limited to, the following:

- Excessively slow recruitment
- Poor protocol adherence
- Inaccurate or incomplete data recording
- Non-compliance with the International Conference on Harmonization guideline for Good Clinical Practice
- No study activity

18. Statistical Analysis

This study will test whether treatment of patients with acute ischemic stroke with intravenous methylprednisolone will lead to a lower mRS score at 90 days.

18.1 Determination of sample size

The current trial is designed detect a shift on the modified Rankin Scale score, which represents the global disability, to a lower score. The expected distribution of the modified Rankin Scale score, derived from the EVT for acute anterior circulation ischemic stroke (ACTUAL) registry[38] and the Highly Effective Reperfusion evaluated in Multiple Endovascular Stroke Trials (HERMES) collaboration[7], is as follows: 7%, 20%, 16%, 11%, 13%, 8% and 25%.

The sample size was calculated using the cumulative proportion of patients with mRS 0-2. We assume a moderate effect of 7% absolute increase in the cumulative proportion of patients with mRS 0-2 in the intervention group, compared with controls, indicating an odds ratio (OR) of 1.33, which have substantially exceeds the minimal clinically important difference.[39,40] In order to demonstrate the expected treatment effect with a type-1 error alpha =0.05 (two-tailed) and a power of 80% (beta = 20%). A sample size of n= 1588 patients (n=794 per treatment group) is required.

The intention-to-treat principle will be applied to the primary analysis and, therefore, to safeguard against dilution of the treatment effect associated with an approximate 5% non-adherence rate (due to loss to follow-up, consent withdrawal and other reasons), we initially planned to enrol n=1672 patients (n=836 per treatment group) for this study. This estimation was performed based on PASS (NCSS, LLC. Kaysville, Utah, USA) version 15.0.

18.2 Analysis population

18.2.1 Intention-to-Treat Population

The ITT population includes all patients randomized into the trial who were recorded as receiving any amount of study drug, even if the subject does not receive the correct treatment, or does not follow the protocol until completion[46]. The ITT population will be the primary analysis population for the efficacy endpoints and subjects will be analysed according to the treatment group to which they were assigned at randomization.

18.2.2. Per-Protocol Population

The PP population is defined as the subset of the ITT population excluding major protocol violators deemed to have the potential to affect patient outcome in terms of efficacy.

The PP population includes patients who actually received the assigned treatment and do not have major protocol violations or deviations. Major protocol violations or deviations will be identified in a blinded fashion prior to database lock. More specifically, patients with anyone of the following criteria will be excluded from the PP population. These deviations will be determined based on the medical monitors' records, as well as programmatically, using the following criteria at a minimum:

- Received but did not complete treatment with study drug, or dose of study drug administered outside recommended dose.
- Received study drug after 2 hours after arterial access closure.
- Violated inclusion or exclusion criteria.

A list of patients to be excluded from the randomized patients to create the PP-Efficacy analysis will be established and validated by the Steering Committee prior to unblinding.

18.2.3. Safety Population

The Safety Population includes all patients who received any amount of study drug. In case of violation of the randomization scheme, patients will be classified according to the treatment they actually received. Patients will be assigned to the different populations prior to unblinding of the database. Patients who withdraw informed consent immediately after randomization and are not to receive any treatment should be excluded from Safety Population.

18.2 Analysis of Efficacy–primary endpoint

The primary efficacy analysis will estimate the effect of treatment with intravenous methylprednisolone and with the placebo in acute ischemic stroke patients.

The primary efficacy outcome is the global disability defined by mRS score at 90 days post-randomization. Adjusted common odds ratio or assumption-free method will be used. The adjusted covariates include age, baseline NIHSS score, pre-stroke mRS, baseline ASPECTS score, use of intravenous thrombolysis, time from onset to randomization, and occlusion location. (Details are described in SAP Section 12.1).

We will analyse the primary outcome in the intention-to-treat (ITT) and per-protocol

(PP) population.

18.3 Analysis of Efficacy – secondary endpoints

The mRS score will be used to evaluate the proportion of patients with a score = 0 to 4 *versus* 5 or 6, 0 to 3 *versus* 4 or higher, 0 to 2 *versus* 3 or higher, 0 to 1(or return to pre stroke morbidity) *versus* 2 or higher and at day 90±7. The adjusted risk ratio will be calculated by fitting the GLM models separately.

NIHSS score at 5-7 days or at early discharge and Functional health status quality of life 90 (±7) days after randomization (EQ-5D-VAS). The EQ-5D-VAS will be compared between the methylprednisolone group and the placebo group via GLM.

Normality and variance homogeneity of residuals will be accessed graphically. The win ratio method will be used if normality assumption is seriously violated.

The significance of each test is determined at the two-sided alpha level of 0.05. The specific statistical model for analysing each of these outcome measures will be detailed in the Statistical Analysis Plan (SAP).

18.4 Safety analysis

Safety outcomes include incidence of overall mortality at 90 (±7) days after randomization, the proportion of patient with SICH within 48 hours after treatment, the proportion of patient with any ICH within 48 hours after treatment, proportion of patients with pneumonia and proportion of patients with gastrointestinal haemorrhage within 7 days after EVT. The safety analysis will be performed on the Safety

Population. The incidences of each outcome will be compared via Chi-square test or Fisher's exact test. The modified Poisson regression models will be fitted to estimate the risk-ratio associated with the treatment effect.[47] Risk ratio with 95% CI will be reported. Patients with missing outcome will not be included in the regression analysis. Additionally, Log-Rank test and the Kaplan-Meier estimates will be plotted over the observation period of 90 days for mortality. Cox regression model will be employed to calculate the hazard ratio with 95%CI.

18.5 Tolerability Analysis (adverse events)

Tolerability analyses will be performed on the Safety Population only.

18.5.1 Adverse Events

Adverse Events will be coded using the Medical Dictionary for Regulatory Activities (MedDRA) central coding dictionary.

18.5.2 Serious Adverse Events

Serious Adverse Event reconciliation will be performed by Data Management, Clinical Research, and Pharmacovigilance via data listings.

A summary of patients with serious adverse events will be presented by treatment group for all patients in the Safety Population. A data listing of SAEs will also be provided, displaying details of the event(s) captured on the CRF. The between group difference will be tested using Chi-square test or Fisher's exact test.

18.5.3 All Adverse Events

Summaries of patients with AEs by System Organ Class and Preferred Term (MedDRA) will be prepared. Each patient will be counted only once within each category (System Organ Class or Preferred Term). If a patient experiences more than one AE within a category, only the AE with the strongest relationship or the greatest intensity, as appropriate, will be included in the summaries. The between group difference will be tested using Chi-square test or Fisher's exact test. Only AEs beginning at or after the beginning of study drug administration will be included.

18.6 Adjustment for Covariates and Subgroup Analyses

In addition to the primary and secondary analyses adjusting for age, baseline NIHSS score, pre-stroke mRS, baseline ASPECTS score, the use of intravenous thrombolysis, time from onset to randomization, and occlusion location, exploratory analyses will be conducted to determine the potential roles of common baseline characteristics and assess potential heterogeneity of treatment effect across subgroups. The primary pre-specified subgroups as follows will be explored.

- age
- sex
- baseline NIHSS score
- pre-stroke mRS
- baseline ASPECTS score
- intravenous thrombolysis

- time from last known well to randomization. (mins)
- occlusion location
- Complete Reperfusion defined as Extended Thrombolysis in Cerebral Infarction grade 2c or 3
- Patients with any radiological haemorrhages
- Patients with symptomatic haemorrhages

18.7 Missing data handling

All efforts will be made to minimize the amount of missing data. However, some missing data may be inevitable due to, for example, loss to follow-up. Missing baseline covariates will be imputed using simple imputation or multiple imputation methods (Details in SAP Section 9.2.2). Sensitivity analyses based on different hypotheses about the missingness pattern of the primary outcome will be performed to test for the robustness of the primary analysis (Details in SAP Section 12.1.5).

19. Ethical and regulatory consideration

19.1 General requirements and Considerations

This study followed the ethical principles of the Helsinki Declaration. Approval of the conduct of the trial will be obtained from the Ethics Committees of all participating centres as well as from the local regulatory authorities. The trial will not start in any centre before written approval and authorization by the respective Ethics Committee and Regulatory Authority. Any subsequent protocol amendment will be submitted to

the Ethics Committee for approval. The involvement of committees in the clinical trial of MARVEL will further ensure that the subjects have the highest priority at any time. In addition to the treatment to be tested, all diagnostic procedures and treatments applied are part of standard management of acute stroke patients and will follow the national guidelines. These procedures are therefore of immediate benefit to the patients. The investigators will assure that every patient participating in the trial will receive best medical treatment.

19.2 Study monitoring and quality control

The design of the MARVEL trial has been carefully reviewed and approved by the Steering Committee before being submitted to the Ethics Committees and Regulatory Authorities for approval. In addition, the independent EAB and DSMB and an external SAB have reviewed and approved the trial protocol and will continuously monitor the conduction of the trial. These committees, composed of well-known independent experts, will ensure sufficient alertness to all ethical and safety issues.

The investigators promise to conduct the MARVEL trial in accordance with this trial protocol, International Conference on Harmonization-GCP (ICH-GCP) Guidelines and applicable regulatory requirements. The investigators agree to provide reliable data and all information required by the trial protocol in an accurate and legible manner according to the instructions provided.

The investigators of this clinical trial is responsible to the health authorities and takes all reasonable measures to ensure the correct implementation of the clinical trial

protocol in terms of ethics, clinical trial protocol compliance, and the completeness and validity of the data recorded on the CRF.

The main responsibility of the monitoring team is to help investigators to ensure that all aspects of clinical trials are highly ethical, scientific, professional and standardized. The monitoring team will regularly contact the centres through field visits, emails or phone calls, and send inspectors to assess the progress of the trial, the compliance of investigators and patients with the trial protocol, and to resolve urgent issues. During these inspection visits, the inspector will work with the on-site investigator. The main aspects of inspection and monitoring are as follows (not exclusive): patient informed consent, patient recruitment and follow-up, serious adverse event recording and reporting, study drug supply, treatment compliance of study drug group participants, study drug count, concomitant treatment and data quality.

19.3 Informed Consent

The written informed consent must be obtained from all participants in the clinical trial prior to inclusion into the study. Informed consent forms must be written to be easily understood by the participants or their legal representatives, enabling them to understand the purpose of the trial, procedures, possible benefits, potential risks, and the rights/obligations of participation. Participants have the right to withdraw from the study at any stage of the trial. Each participant must leave contact information to the investigator of the coordinating centre. At the same time, the investigator must leave

his own phone number to the participant so that the participant can find the investigator at any time.

19.4 Confidentiality

Personal data will be processed in accordance with Chinese data protection directives and regulations, relevant international legislation and good practices. Data will only be processed for the trial's purposes. The investigators encode each patient participating in the study by assigning a unique patient identification number to maintain confidentiality standards. This means that all individual patients' data will be linked to the CRF via a unique identification number throughout the trial. Individual patient's medical information will be recorded only in anonymous form. The clinical monitors may inspect source data in order to ensure the accuracy of the data recorded in the CRF.

19.5 Liability and Insurance

The study investigators provide an appropriate insurance for patients in the event of any trial related damage in accordance with applicable national laws. A certificate of insurance will be provided to the investigator of the coordinating centre in which this document is required.

20. Administrative procedures

20.1 Curriculum vitae

A latest copy of the curriculum vitae of each investigator and co-investigator will be provided to the responsible coordinating centre prior to the start of the study.

20.2 Secrecy agreement

The investigators will take all necessary measures to ensure that there is no violation of confidentiality in respect of all information accumulated, acquired or deduced in the course of the trial, other than that information to be disclosed by law.

20.3 Ownership of data and use of the study results

All materials, information and unpublished documentation supplied to the investigators, inclusive of this study, and the patient case report forms are the exclusive property of the study initiators. Therefore, the study initiators reserve the right to use the data of the present study, either in the form of case report forms, or in the form of a report, with or without comments and with or without analysis, in order to submit them to the health authorities.

20.4 Protocol amendments

Any protocol amendments will be prepared by the investigators. Protocol amendments should be submitted to the REB/IRB for approval prior to implementation in accordance with local regulatory requirements. Approval must be obtained from the REB/IRB and regulatory authorities (as locally required) before implementation of any changes, except for changes necessary to eliminate an immediate hazard to patients or changes that involve logistical or administrative aspects only

21. Data Retention

The double reviewed case report form (CRF) and imaging data will be sent to the data management group. The person in charge of the data management group will check and sign the receipt form. The CRF will be kept by the research centre after data entry is completed.

22. Study report and publications

The results of the trial will be reported to the regulatory authorities and ethics committees. The investigators will provide an annual safety report and the final report.

According to the pre-defined analysis in the clinical trial protocol, the results of the trial will be published in the appropriate journal (for manuscripts) or meeting (for abstracts).

By signing the clinical trial protocol， the investigator agrees that the results of the clinical trial can be used for publication.

The trial will be registered at the Chinese Clinical Trial Registry website.

23. ReferencesUncategorized References

1. Global, regional, and national burden of stroke and its risk factors, 1990-2019: a systematic analysis for the Global Burden of Disease Study 2019. Lancet Neurol 2021;20:795-820.

2. Diseases GBD, Injuries C. Global burden of 369 diseases and injuries in 204 countries and territories, 1990-2019: a systematic analysis for the Global Burden of Disease Study 2019. Lancet 2020;396:1204-22.

3. Lindsay MP, Norrving B, Sacco RL, et al. World Stroke Organization (WSO): Global Stroke Fact Sheet 2019. Int J Stroke 2019;14:806-17.

4. Wang W, Jiang B, Sun H, et al. Prevalence, Incidence, and Mortality of Stroke in China: Results from a Nationwide Population-Based Survey of 480 687 Adults. Circulation 2017;135:759-71.

5. Baron JC. Protecting the ischaemic penumbra as an adjunct to thrombectomy for acute stroke. Nat Rev Neurol 2018;14:325-37.

6. Powers WJ, Rabinstein AA, Ackerson T, et al. Guidelines for the Early Management of Patients With Acute Ischemic Stroke: 2019 Update to the 2018 Guidelines for the Early Management of Acute Ischemic Stroke: A Guideline for Healthcare Professionals From the American Heart Association/American Stroke Association. Stroke 2019;50:e344-e418.

7. Goyal M, Menon BK, van Zwam WH, et al. Endovascular thrombectomy after large-vessel ischaemic stroke: a meta-analysis of individual patient data from five randomised trials. LANCET 2016;387:1723-31.

8. Nie X, Pu Y, Zhang Z, Liu X, Duan W, Liu L. Futile Recanalization after Endovascular Therapy in Acute Ischemic Stroke. Biomed Res Int 2018;2018:5879548.

9. Stoll G, Pham M. Beyond recanalization — a call for action in acute stroke. Nature Reviews Neurology 2020;16:591-2.

10. Whiteley WN, Emberson J, Lees KR, et al. Risk of intracerebral haemorrhage with alteplase after acute ischaemic stroke: a secondary analysis of an individual patient data meta-analysis. Lancet Neurol 2016;15:925-33.

11. Shi ZS, Duckwiler GR, Jahan R, et al. Early Blood-Brain Barrier Disruption after Mechanical Thrombectomy in Acute Ischemic Stroke. J Neuroimaging 2018;28:283-8.

12. Conti E, Piccardi B, Sodero A, et al. Translational Stroke Research Review: Using the Mouse to Model Human Futile Recanalization and Reperfusion Injury in Ischemic Brain Tissue. Cells 2021;10:19.

13. Cheripelli BK, Huang XY, MacIsaac R, Muir KW. Interaction of Recanalization, Intracerebral Hemorrhage, and Cerebral Edema After Intravenous Thrombolysis. Stroke 2016;47:1761-7.

14. Chen XL, Huang Q, Deng QW, et al. A prediction model of brain edema after endovascular treatment in patients with acute ischemic stroke. Journal of the Neurological Sciences 2019;407.

15. Ames A, 3rd, Wright RL, Kowada M, Thurston JM, Majno G. Cerebral ischemia. II. The no-reflow phenomenon. The American journal of pathology 1968;52:437-53.

16. Caiazzo G, Musci RL, Frediani L, et al. State of the Art: No-Reflow Phenomenon. Cardiol Clin 2020;38:563-73.

17. Kloner RA, King KS, Harrington MG. No-reflow phenomenon in the heart and brain. Am J Physiol Heart Circ Physiol 2018;315:H550-H62.

18. Planas AM. Role of Immune Cells Migrating to the Ischemic Brain. Stroke 2018;49:2261-7.

19. Shi KB, Tian DC, Li ZLG, Ducruet AF, Lawton MT, Shi FD. Global brain inflammation in stroke. Lancet Neurology 2019;18:1058-66.

20. Laha RK, Dujovny M, Barrionuevo PJ, DeCastro SC, Hellstrom HR, Maroon JC. Protective effects of

methyl prednisolone and dimethyl sulfoxide in experimental middle cerebral artery embolectomy. J Neurosurg 1978;49:508-16.

21. Slivka AP, Murphy EJ. High-dose methylprednisolone treatment in experimental focal cerebral ischemia. Exp Neurol 2001;167:166-72.

22. de Courten-Myers GM, Kleinholz M, Wagner KR, Xi G, Myers RE. Efficacious experimental stroke treatment with high-dose methylprednisolone. Stroke 1994;25:487-92; discussion 93.

23. Barbosa-Coutinho LM, Hartmann A, Hossmann KA, Rommel T. Effect of dexamethasone on serum protein extravasation in experimental brain infarcts of monkey: an immunohistochemical study. Acta Neuropathol 1985;65:255-60.

24. Espinosa A, Meneses G, Chavarría A, et al. Intranasal Dexamethasone Reduces Mortality and Brain Damage in a Mouse Experimental Ischemic Stroke Model. Neurotherapeutics 2020;17:1907-18.

25. Altamentova S, Rumajogee P, Hong J, et al. Methylprednisolone Reduces Persistent Post-ischemic Inflammation in a Rat Hypoxia-Ischemia Model of Perinatal Stroke. Transl Stroke Res 2020;11:1117-36.

26. Limbourg FP, Huang Z, Plumier JC, et al. Rapid nontranscriptional activation of endothelial nitric oxide synthase mediates increased cerebral blood flow and stroke protection by corticosteroids. J Clin Invest 2002;110:1729-38.

27. Norris JW. Steroids may have a role in stroke therapy. Stroke 2004;35:228-9.

28. Nellis SH, Roberts BH, Kinney EL, Field J, Ummat A, Zelis R. Beneficial effect of dexamethasone on the "no reflow" phenomenon in canine myocardium. Cardiovasc Res 1980;14:137-41.

29. Sandercock PAG, Soane T. Corticosteroids for acute ischaemic stroke. Cochrane Database of Systematic Reviews 2011.

30. Ogun SA, Odusote KA. Effectiveness of high dose dexamethasone in the treatment of acute stroke. West African journal of medicine 2001;20:1-6.

31. Lyden P, Buchan A, Boltze J, et al. Top Priorities for Cerebroprotective Studies—A Paradigm Shift: Report From STAIR XI. Stroke 2021;52:3063-71.

32. Lapchak PA. Emerging Therapies: Pleiotropic Multi-target Drugs to Treat Stroke Victims. Translational Stroke Research 2011;2:129-35.

33. Fraser JF, Pahwa S, Maniskas M, et al. Now that the door is open: an update on ischemic stroke pharmacotherapeutics for the neurointerventionalist. Journal of Neurointerventional Surgery 2023.

34. Savitz SI, Baron JC, Fisher M, Consortium SX. Stroke Treatment Academic Industry Roundtable X: Brain Cytoprotection Therapies in the Reperfusion Era. Stroke 2019;50:1026-31.

35. Savitz SI, Baron J-C, Yenari MA, Sanossian N, Fisher M. Reconsidering Neuroprotection in the Reperfusion Era. 2017;48:3413-9.

36. Davis SM, Donnan GA. Steroids for stroke: another potential therapy discarded prematurely? Stroke 2004;35:230-1.

37. Huting ZHANG SL. Influence of neurological intervention combined with dexamethasone injection on the outcome for the patients suffered from acute ischemic stroke. Journal of Molecular Imaging 2016;39:121-4.

38. Zi W, Wang H, Yang D, et al. Clinical Effectiveness and Safety Outcomes of Endovascular Treatment for Acute Anterior Circulation Ischemic Stroke in China. Cerebrovasc Dis 2017;44:248-58.

39. Cranston JS, Kaplan BD, Saver JL. Minimal Clinically Important Difference for Safe and Simple Novel Acute Ischemic Stroke Therapies. Stroke 2017;48:2946-51.

40. Berkhemer OA, Fransen PSS, Beumer D, et al. A Randomized Trial of Intraarterial Treatment for Acute Ischemic Stroke. New England Journal of Medicine 2015;372:11-20.

41. Wang Y, Han S, Qin H, et al. Chinese Stroke Association guidelines for clinical management of cerebrovascular disorders: executive summary and 2019 update of the management of high-risk population. Stroke Vasc Neurol 2020;5:270-8.
42. Banks JL, Marotta CA. Outcomes validity and reliability of the modified Rankin scale: implications for stroke clinical trials: a literature review and synthesis. Stroke 2007;38:1091-6.
43. Quinn TJ, Dawson J, Walters MR, Lees KR. Reliability of the modified Rankin Scale: a systematic review. Stroke 2009;40:3393-5.
44. Brott T, Adams HP, Jr., Olinger CP, et al. Measurements of acute cerebral infarction: a clinical examination scale. Stroke 1989;20:864-70.
45. von Kummer R, Broderick JP, Campbell BC, et al. The Heidelberg Bleeding Classification: Classification of Bleeding Events After Ischemic Stroke and Reperfusion Therapy. Stroke 2015;46:2981-6.
46. White IR, Horton NJ, Carpenter J, Pocock SJ. Strategy for intention to treat analysis in randomised trials with missing outcome data. Bmj 2011;342:d40.
47. Zou G. A modified poisson regression approach to prospective studies with binary data. Am J Epidemiol 2004;159:702-6.

25. Appendices

25.1 Appendix 1: mRS

Subject ID ___ ___ ___ ___ ___ ___ ___ ___ ___ ___
Subject Date of Birth ___ ___/___ ___/___ ___
Hospital ID ______________________________
Date of Examination ___ ___/___ ___/___ ___

The Modified Rankin Scale (mRS)

(Use web calculator at www.modifiedrankin.com)

0 No symptoms

1 No significant disability; able to carry out all usual activities, despite some symptoms

2 Slight disability; able to look after own affairs without assistance, but unable to carry out all previous activities

3 Moderate disability; requires some help, but able to walk unassisted

4 Moderately severe disability; unable to attend to own bodily needs without assistance, and unable to walk unassisted

5 Severe disability; requires constant nursing care and attention, bedridden, incontinent

6 Dead

References:

Rankin J (May 1957). "Cerebral vascular accidents in patients over the age of 60. II. Prognosis". Scott Med J 2 (5): 200–15

Patel, N., et al. Simple and reliable determination of the modified Rankin Scale in neurosurgical and neurological patients: The mRS-9Q. *Neurosurgery*, published online in advance of print 26 July 2012

25.2 Appendix 2: NIHSS

NIHSS page 1

Subject ID ___ ___ ___ ___ ___ ___ ___ ___ ___ ___
Subject Date of Birth ___ ___/___ ___/___ ___
Hospital ID ______________________________
Date of Examination ___ ___/___ ___/___ ___

NATIONAL INSTITUTES OF HEALTH STROKE SCALE (NIHSS)[1]

Interval: [ ] Baseline
[ ] 7-10 days
[ ] 1 month
[ ] 3 months
[ ] 6 months
[ ] Other ________________________________

Time: ___ ___:___ ___ [ ]am [ ]pm

Person Administering Scale ________________________________

Purpose:
The NIH Stroke Scale (NIHSS) is a standardized neurological examination intended to describe the neurological deficits found in large groups of stroke patients participating in treatment trials.

Administer stroke scale items in the order listed. Record performance in each category after each subscale exam. Do ***not*** go back and change scores. Follow directions provided for each exam technique. Scores should reflect what the patient does, not what the clinician thinks the patient can do. The clinician should record answers while administering the exam and work quickly. Except where indicated, the patient should not be coached (i.e., repeated requests to patient to make a special effort).

Instructions	Scale Definition	Score
**1a. Level of Consciousness:** The investigator must choose a response if a full evaluation is prevented by such obstacles as an endotracheal tube language barrier, orotracheal trauma/bandages. A 3 is scored only if the patient makes no movement (other than reflexive posturing) in response to noxious stimulation.	0 = **Alert**; Keenly responsive.<br>1 = **Not alert, but arousable** by minor stimulation to obey, answer or respond.<br>2 = **Not alert**; requires repeated stimulation to attend, or is obtunded and requires strong or painful. stimulation to make movements (not stereotyped).<br>3 = **Responds only with reflex motor or autonomic effects or totally unresponsive**, flaccid, and areflexic.	______
**1b. LOC Questions**: The patient is asked the month and his/her age. The answer must be correct – there is no partial credit for being close. Aphasic and stuporous patients who do not comprehend the questions will score 2. Patients unable to speak because of endotracheal intubation, orotracheal trauma, severe dysarthria from any cause, language barrier, or any other problem not secondary to aphasia are given a 1. It is important that only the initial answer be graded and that the examiner not "help" the patient with verbal or non-verbal cues.	0 = **Answers both questions correctly**.<br>1 = **Answers one question correctly**.<br>2 = **Answers neither question correctly**.	______

[1] The National Institute of Neurological Diseases and Stroke (NINDS), National Institutes of Health (NIH), Last Revised 01 October 2003 (https://stroke.nih.gov/resources/index.htm).

NIHSS page 2

Instructions	Scale Definition	Score
**6. Motor Leg:** The limb is placed in the appropriate position: hold the leg at 30 degrees (always tested supine). Drift is scored if the leg falls before 5 seconds. The aphasic patient is encouraged using urgency in the voice and pantomime, but not noxious stimulation. Each limb is tested in turn, beginning with the non-paretic leg. Only the case of amputation or joint fusion at the hip, should the examiner record the score as untestable (UN), and clearly write the explanation for this choice.	0 = **No drift**; limb holds 90 (or 45) degrees for full 10 seconds.<br>1 = **Drift**; limb holds 90 (or 45) degrees, but drifts down before full 10 seconds; does not hit bed or other support.<br>2 = **Some effort against gravity**; limb cannot get to or maintain (if cued) 90 (or 45) degrees, drifts down to bed, but has some effort against gravity.<br>3 = **No effort against gravity**; leg falls to bed immediately.<br>4 = **No movement**.<br>UN= **Amputation** or joint fusion; explain: ____________<br>6a= **Left Arm**.<br>6b= **Right Arm**.	______<br>______
**7. Limb Ataxia:** This item is aimed at finding evidence of a unilateral cerebellar lesion. Test with eyes open. In case of visual defect, ensure testing is done in intact visual field. The finger-nose-finger and heel-shin tests are performed on both sides, and ataxia is scored only if present out of proportion to weakness. Ataxia is absent in the patient who cannot understand or is paralyzed. Only in the case of amputation or joint fusion, should the examiner record the score as untestable (UN), and clearly write the explanation for this choice. In case of blindness, test by having the patient touch nose from extended arm position.	0 = **Absent.**<br>1 = **Present in one limb**.<br>2 = **Present in two limbs**.<br>UN= **Amputation** or joint fusion; explain: ____________	______
**8. Sensory:** Sensation or grimace to pinprick when tested, or withdrawal from noxious stimulus in the obtunded or aphasic patient. Only sensory loss attributed to stroke is scored as abnormal and the examiner should test as many body areas [arms (not hands), legs, trunk, face) as needed to accurately check for hemisensory loss. A score of 2, "severe or total sensory loss," should only be given when a severe or total loss of sensation can be clearly demonstrated. Stuporous and aphasic patients will, therefore, probably score 1 or 0. The patient with brainstem stroke who has bilateral loss of sensation is scored 2. If the patient does not respond and is quadriplegic, score 2. Patients in a coma (item 1a=3) are automatically given a 2 on this item.	0 = **Normal**; no sensory loss.<br>1 = **Mild-to-moderate** sensory loss; patient feels pinprick is less sharp or is dull on the affected side, or there is a loss of superficial pain with pinprick, but patient is aware of being touched.<br>2 = **Severe to total** sensory loss; patient is not aware of being touched in the face, arm, and leg.	______
**9. Best Language:** A great deal of information about comprehension will be obtained during the preceding sections of the examination. For this scale item, the patient is asked to describe what is happening in the attached picture, to name the items on the attached naming sheet and to read from the attached list of sentences. Comprehension is judged from responses here, as well as to all of the commands in the preceding general neurological exam. If visual loss interferes with the tests, ask the patient to identify objects placed in the hand, repeat, and produce speech. The intubated patient should be asked to write. The patient in a coma (item 1a=3) will automatically score 3 on this item. The examiner must choose a score for the patient with stupor or limited cooperation, but a score of 3 should be used only if the patient is mute and follows no one-step commands.	0 = **No asphasia**; normal.<br>1 = **Mild-to-moderate aphasia**; some obvious loss of fluency or facility of comprehension, without significant limitation on ideas expressed or form of expression. Reduction of speech and/or comprehension, however, makes conversation about provided materials difficult or impossible. For example, in conversation about provided materials, examiner can identify picture or naming card content from patient's response.<br>2 = **Severe asphasia**; all communication is through fragmentary expression; great need for inference, questioning, and guessing by the listener. Range of information that can be exchanged is limited; listener carries burden of communication. Examiner cannot identify materials provided from patient response.<br>3 = **Mute, global aphasia**; no usable speech or auditory comprehension.	______

NIHSS page 3

Instructions	Scale Definition	Score
**1c. LOC Commands:** The patient is asked to open and close the eyes and then to grip and release the non-paretic hand. Substitute another one step command if the hands cannot be used. Credit is given if an unequivocal attempt is made but not completed due to weakness. If the patient does not respond to command, the task should be demonstrated to him/her (pantomime), and the result scored (i.e., follows none, one or two commands). Patients with trauma, amputation, or other physical impediments should be given suitable one-step commands. Only the first attempt is scored.	0 = **Answers both tasks correctly**.<br>1 = **Answers one task correctly**.<br>2 = **Answers neither task correctly**.	________
**2. Best Gaze**: Only horizontal eye movements will be tested. Voluntary or reflexive (oculocephalic) eye movements will be scored, but caloric testing is not done. If the patient has a conjugate deviation of the eyes that can be overcome by voluntary or reflexive activity, the score will be 1. If a patient has an isolated peripheral nerve paresis (CN III, IV or VI), score a 1. Gaze is testable in all aphasic patients. Patients with ocular trauma, bandages, pre-existing blindness, or other disorder of visual acuity or fields should be tested with reflexive movements, and a choice made by the investigator. Establishing eye contact and then moving about the patient from side to side will occasionally clarify the presence of a partial gaze palsy.	0 = **Normal.**<br>1 = **Partial gaze palsy**; gaze is abnormal in one or both eyes, but forced deviation or total gaze paresis is not present.<br>2 = **Forced deviation**, or total gaze paresis not overcome by the oculocephalic maneuver.	________
**3. Visual:** Visual fields (upper and lower quadrants) are tested by confrontation, using finger counting or visual threat, as appropriate. Patients may be encouraged, but if they look at the side of the moving fingers appropriately, this can be scored as normal. If there is unilateral blindness or enucleation, visual fields in the remaining eye are scored. Score 1 only if a clear-cut asymmetry, including quadrantanopia, is found. If patient is blind from any cause, score 3. Double simultaneous stimulation is performed at this point. If there is extinction, patient receives a 1, and the results are used to respond to item 11.	0 = **No visual loss**.<br>1 = **Partial hemianopia**.<br>2 = **Complete hemianopia**.<br>3 = **Bilateral hemianopia** (blind including cortical blindness).	________
**4. Facial Palsy:** Ask – or use pantomime to encourage – the patient to show teeth or raise eyebrows and close eyes. Score symmetry of grimace in response to noxious stimuli in the poorly responsive or non-comprehending patient. If facial trauma/bandages, orotracheal tube, tape or other physical barriers obscure the face, these should be removed to the extent possible.	0 = **Normal** symmetrical movements.<br>1 = **Minor paralysis** (flattened nasolabial fold, asymmetry on smiling).<br>2 = **Partial paralysis** (total or near-total paralysis of lower face).<br>3 = **Complete paralysis** of one or both sides (absence of facial movement in the upper and lower face	________
**5. Motor Arm:** The limb is placed in the appropriate position: extend the arms (palms down) 90 degrees (if sitting) or 45 degrees (if supine). Drift is scored if the arm falls before 10 sounds. The aphasic patient is encouraged using urgency in the voice and pantomime, but not noxious stimulation. Each limb is tested in turn, beginning with the non-paretic arm. Only in the case of amputation or joint fusion at the shoulder, should the examiner record the score as untestable (UN), and clearly write the explanation for this choice.	0 = **No drift**; limb holds 90 (or 45) degrees for full 10 seconds.<br>1 = **Drift**; limb holds 90 (or 45) degrees, but drifts down before full 10 seconds; does not hit bed or other support.<br>2 = **Some effort against gravity**; limb cannot get to or maintain (if cued) 90 (or 45) degrees, drifts down to bed, but has some effort against gravity.<br>3 = **No effort against gravity**; limb falls.<br>4 = **No movement**.<br>UN= **Amputation** or joint fusion; explain: ____________________<br>5a= **Left Arm**.<br>5b= **Right Arm**.	________<br>________

NIHSS page 4

Instructions	Scale Definition	Score
**10. Dysarthria:** If patient is thought to be normal, an adequate sample of speech must be obtained by asking patient to read or repeat words from the attached list. If the patient has severe aphasia, the clarity of articulation of spontaneous speech can be rated. Only if the patient is intubated or has other physical barriers to producing speech, should the examiner record the score as untestable (UN), and clearly write an explanation for this choice. Do not tell the patient why he or she is being tested.	0 = **Normal**.<br>1 = **Mild-to-moderate dysarthria**; patient slurs at least some words and, at worst, can be understood with some difficulty.<br>2 = **Severe dysarthria**; patient's speech is so slurred as to be unintelligible in the absence of or out of proportion to any dysphasia, or is mute/anarthric.<br>UN= **Intubated** or other physical barrier; explain: ____________	______
**11. Extinction and Inattention (formerly Neglect):** Sufficient information to identify neglect may be obtained during the prior testing. If the patient has a severe visual loss preventing visual double simultaneous stimulation, and the cutaneous stimuli are normal, the score is normal. If the patient has aphasia but does appear to attend to both sides, the score is normal. The presence of visual spatial neglect or anosagnosia may also be taken as evidence of abnormality. Since the abnormality is scored only if present, the item is never untestable.	0 = **No abnormality**.<br>1 = **Visual, tactile, auditory, spatial, or personal inattention** or extinction to bilateral simultaneous stimulation in one of the sensory modalities.<br>2 = **Profound hemi-inattention or extinction to more than one modality**; does not recognize own hand or orients to only one side of space.	______

NIHSS page 5

Example picture-description stimulus.
Adapted from “Banana Theft v2” by Sam Mshiu, via Wikimedia Commons, licensed under CC BY 4.0. This image is used as an illustrative replacement for the original NIHSS Cookie Theft stimulus and is not the official NIHSS stimulus.

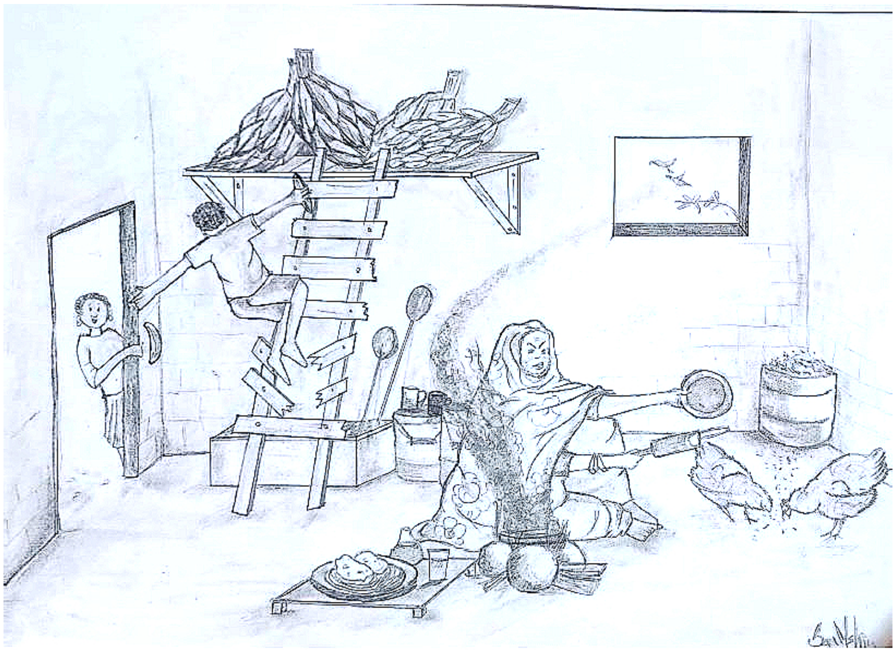

You know how.

Down to earth.

I got home from work.

Near the table in the dining room.

They heard him speak on the radio last night.

请您读出下列句子：

知道

下楼梯

回家做饭

在学校复习

发表精彩演讲

NIHSS page 6

Example picture-description stimulus. Object-naming stimulus assembled from public-domain Openclipart images: Glove, Key, Cactus, Feather, Chair, and Hammock. This is not the official NIHSS stimulus card. The individual source images are public-domain/CC0 Openclipart works, and the assembled/adapted figure is published under CC BY 4.0.

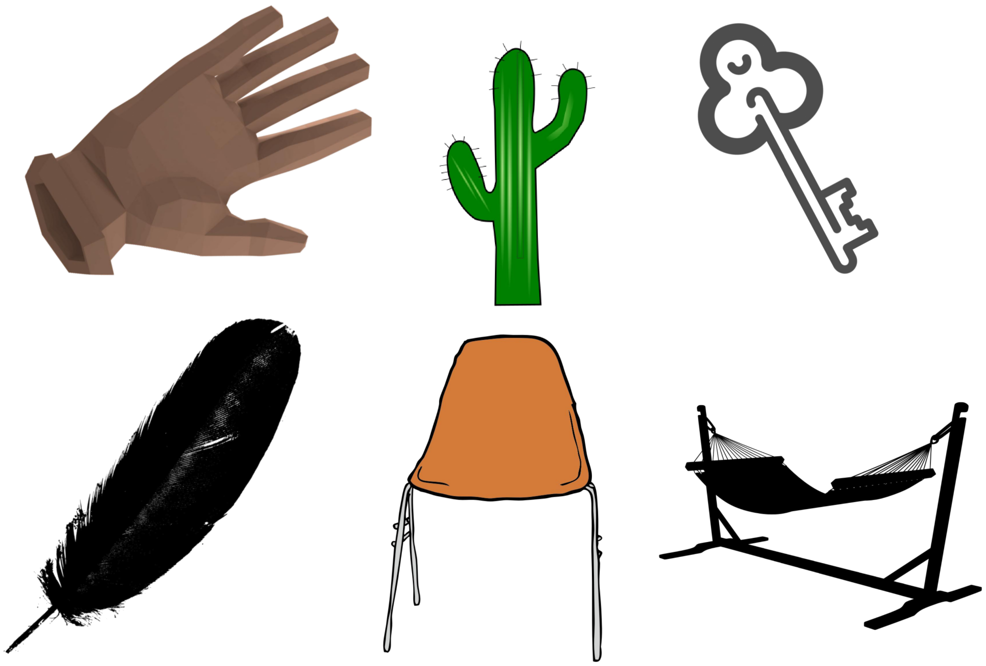

MAMA

TIP – TOP

FIFTY – FIFTY

THANKS

HUCKLEBERRY

BASEBALL PLAYER

请您读出下列单词：

妈妈

大地

飞机飞机

丝绸

按时开工

吃葡萄不吐葡萄皮

25.2 Appendix 3: EQ-5D

Subject ID ___ ___ ___ ___ ___ ___ ___ ___ ___ ___
Subject Date of Birth ___ ___/___ ___/___ ___
Hospital ID ________________________________
Date of Examination ___ ___/___ ___/___ ___

Health Questionnaire (EQ-5D-5L)

Under each heading, please tick the ONE box that best describes your health TODAY.

MOBILITY

- ☐ 1 I have no problems in walking about
- ☐ 2 I have slight problems in walking about
- ☐ 3 I have moderate problems in walking about
- ☐ 4 I have severe problems in walking about
- ☐ 5 I am unable to walk about

SELF-CARE

- ☐ 1 I have no problems washing or dressing myself
- ☐ 2 I have slight problems washing or dressing myself
- ☐ 3 I have moderate problems washing or dressing myself
- ☐ 4 I have severe problems washing or dressing myself
- ☐ 5 I am unable to wash or dress myself

USUAL ACTIVITIES *(e.g. work, study, housework, family or leisure activities)*

- ☐ 1 I have no problems doing my usual activities
- ☐ 2 I have slight problems doing my usual activities
- ☐ 3 I have moderate problems doing my usual activities
- ☐ 4 I have severe problems doing my usual activities
- ☐ 5 I am unable to do my usual activities

PAIN / DISCOMFORT

- ☐ 1 I have no pain or discomfort
- ☐ 2 I have slight pain or discomfort
- ☐ 3 I have moderate pain or discomfort
- ☐ 4 I have severe pain or discomfort
- ☐ 5 I have extreme pain or discomfort

ANXIETY / DEPRESSION

- ☐ 1 I am not anxious or depressed
- ☐ 2 I am slightly anxious or depressed
- ☐ 3 I am moderately anxious or depressed
- ☐ 4 I am severely anxious or depressed
- ☐ 5 I am extremely anxious or depressed

To help people say how good or bad a health state is, we have drawn a scale (rather like a thermometer) on which the best state you can imagine is marked 100 and the worst state you can imagine is marked 0.

We would like you to indicate on this scale how good or bad your own health is today, in your opinion. Please do this by drawing a line from the box below to whichever point on the scale indicates how good or bad your health state is today.

Your own health state today

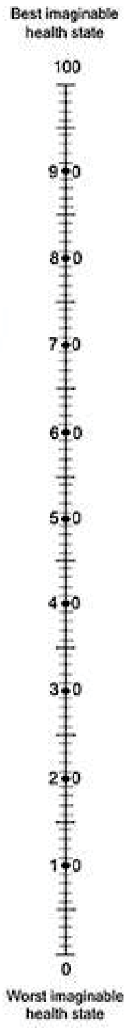

3

UK (English) © 1990 EuroQol Group EQ-5D™ is a trade mark of the EuroQol Group

Clinical study protocol

Efficacy and safety of intravenous methylprednisolone sodium succinate in acute ischemic stroke patients underwent endovascular treatment: a multicenter, randomized, double-blind trial.

Principal Investigators

Wenjie Zi, MD. and Qingwu Yang, Ph.D.

Xinqiao Hospital, Army Medical University, Chongqing, China

Prepared by:

Wenjie Zi, MD, Xinqiao Hospital, Army Medical University, Chongqing, China

Qingwu Yang, Ph.D, Xinqiao Hospital, Army Medical University, Chongqing, China

Protocol Version: 2.0

Table of Contents

1.Study Synopsis **96**

2.Flow chart and schedule of assessment **106**

2.1 Flow Chart 106
2.2. Schedule of Assessments 107

3.List of Abbreviations **109**

4.Background **111**

4.1 The burden of stroke 111
4.2 Treatment in acute ischemic stroke 111
4.3 Glucocorticoid in Stroke 113

5.Study Objectives **115**

6.Product characteristics of methylprednisolone **116**

6.1 Description of the investigational medicinal product 116
6.2 Indications and Usage 117
6.3 Contraindications 120
6.4 Warning 120

7.Organizational Structure **127**

7.1 Funding 128
7.2 Trials Boards and Committees 128
7.2.1 Steering Committee 128
7.2.2 Data Safety and Monitoring Board 128
7.2.3 Ethics Advisory Board 129
7.2.4 Imaging Core Laboratory 129
7.2.5 Clinical Events Committee 129

8.Design **130**

9.Population **130**

9.1 Inclusion criteria 130
9.2 Exclusion Criteria 131
9.3 Sample Size 132
10.Imaging Training 133

11.Randomization **133**

12.Blinding/Unblinding **134**

13.Study Treatment **135**

13.1 Formulation, Packaging, and Handling 135
13.2 Test Product 135
13.3 Comparator 135
13.4 Dosage, Administration, and Compliance 136

13.4.1 Methylprednisolone and its Placebo.......136
13.4.2 Concomitant therapy.......136
13.4.4 Drug Accountability.......137

14.Study Schedule.......137

14.1 Screening period (Visit V0, Randomization).......138
14.1.1 Visit V0.......138
14.1.2 Randomization (R).......139
14.2 Treatment period (Visits V1, V2).......139
14.2.1 Visit 1.......139
14.2.2 Visit 2.......140
14.3 Follow Up period (Visits V3, V4).......140
14.3.1 Visit 3.......141
14.3.3 Visit 4.......141

15.Endpoints of the Clinical Trial.......141

15.1 Efficacy Analysis-Primary Endpoint.......141
15.2 Efficacy Analysis-Secondary Endpoints.......142
15.3 Safety Analysis-Primary Endpoint.......142
15.3.1 Primary Safety Endpoints.......142
15.3.2 Secondary Safety Endpoints.......143

16.Assessment.......143

16.1 Assessment of efficacy.......144
16.1.1 The modified Rankin Scale (mRS).......144
16.1.2 The National Institutes of Health Stroke Scale (NIHSS).......145
16.1.3 European Quality Five Dimensions Five Level scale.......146
16.2 Assessment of safety.......146
16.2.1 Mortality at 90±7 days.......146
16.2.2 Symptomatic Intracranial Haemorrhage.......146
16.2.3 Any ICH within 48 hours.......147
16.2.4 Proportion of patients with pneumonia.......148
16.2.4 Proportion of patients with gastrointestinal bleeding.......148
16.2.5 Adverse events.......148
16.2.6 Serious Adverse Event.......151
16.2.7 Adverse Event Recording and Follow Up.......152
16.2.8 Serious Adverse Events Reporting.......153

17.Study Discontinuation Criteria.......154

17.1 Patient Discontinuation.......154
17.2 Study Treatment Discontinuation.......155
17.3 Study and Site Discontinuation.......155

18.Statistical Analysis.......156

18.1 Determination of sample size.......156
18.2 Analysis population.......157
18.2.1 Intention-to-Treat Population.......157

18.2.2. Per-Protocol Population 158
18.2.3. Safety Population 159
18.2 Analysis of Efficacy–primary endpoint....159
18.3 Analysis of Efficacy – secondary endpoints....160
18.4 Safety analysis 160
18.5 Tolerability Analysis (adverse events)....161
18.5.1 Adverse Events 161
18.5.2 Serious Adverse Events 161
18.5.3 All Adverse Events....161
18.6 Adjustment for Covariates and Subgroup Analyses 162
18.7 Missing data handling....163
19.Ethical and regulatory consideration....163
19.1 General requirements and Considerations 163
19.2 Study monitoring and quality control....164
19.3 Informed Consent....165
19.4 Confidentiality 166
19.5 Liability and Insurance 166
20.Administrative procedures 167
20.1 Curriculum vitae....167
20.2 Secrecy agreement 167
20.3 Ownership of data and use of the study results....167
20.4 Protocol amendments....167
21.Data Retention 168
22.Study report and publications....168
23.References Uncategorized References....169
24.Appendices 172
24.1 Appendix 1: mRS 172
24.2 Appendix 2: NIHSS 174
24.3 Appendix 3: EQ-5D 182

1. **Study Synopsis**

**Study Title**	MARVEL: Methylprednisolone for acute large vessel occlusion: a randomized double-blind, placebo-controlled trial in recanalization patients
**Study registry number**	**registry number:** ChiCTR2100051729
**Indication**	Acute Ischemic Stroke
**Study Centres**	About 80 stroke centres in China
**Number of Subjects**	1672 (836 subjects per group)
**Study Estimate Duration**	February 2022 (first patient in) until July 2023
**Study Objectives**	To test the safety and efficacy of adjunctive low-dose methylprednisolone sodium succinate therapy for the treatment of acute ischemic stroke patients within 24 hours of onset who underwent EVT.
**Study Design**	A multicentre, randomized, double-blind, placebo-controlled trial
**Study Population**	Patients with large vessels occlusive stroke within 24 hours that underwent endovascular treatment
**Inclusion Criteria**	● Age $\geqslant$ 18 years;

	● The time from onset to randomization was within 24 hours;<br>● Anterior circulation ischemic stroke was preliminarily determined according to clinical symptoms or imaging examination;<br>● Baseline Alberta Stroke Program Early CT Score (ASPECTS) ≥ 3;<br>● Baseline National Institutes of Health Stroke Scale (NIHSS) ≥ 6<br>● Computed tomography angiography (CTA) /magnetic resonance angiography (MRA) /digital subtraction angiography (DSA) confirmed occlusion of intracranial segment of internal carotid artery and middle cerebral artery who decided to receive endovascular treatment.<br>● Written informed consent signed by patients or their family members.
**Exclusion Criteria**	● Intracranial hemorrhage confirmed by cranial computed tomography (CT) or magnetic resonance imaging (MRI);<br>● mRS score ≥ 2 before onset;<br>● Pregnant or lactating women;

	● Allergic to contrast agents;<br>● Allergic to glucocorticoids;<br>● Participating in other clinical trials;<br>● Systolic blood pressure > 185 mmHg or diastolic pressure > 110 mmHg, and oral antihypertensive drugs can not control;<br>● Genetic or acquired bleeding constitution, lack of anticoagulant factors; Or oral anticoagulants and INR > 1.7;<br>● Blood sugar < 2.8 mmol/L (50 mg/dl) or > 22.2 mmol/L (400 mg/dl), platelet < 90 x 10^9/L;<br>● The artery is tortuous and the thrombectomy device cannot reach the target vessel;<br>● Bleeding history (gastrointestinal and urinary tract bleeding) in recent 1 month;<br>● Chronic hemodialysis and severe renal insufficiency (glomerular filtration rate < 30 ml/min or serum creatinine > 220 umol/L [2.5 mg/ dL]);<br>● Life expectancy due to any advanced disease < 6 months;<br>● Follow-up is not expected to be completed;

	● Intracranial aneurysm and arteriovenous malformation;<br>● Brain tumors with imaging mass effect;<br>● Severe systemic infectious diseases.
**Study drug**	Methylprednisolone group: The box of the methylprednisolone group contains 12 bottles, each containing 40mg of methylprednisolone sodium succinate. Both the bottle and box are labelled as “Study Drug”.<br>Placebo group: The box of the placebo group contains 12 bottles, each containing 40mg of placebo. Both the bottle and box are labelled as “Study Drug”.
**Randomization**	Eligible patients will be consecutively randomized to treatment with either Methylprednisolone or Placebo group with a ratio of 1:1 by a web-based APP (Jinlingshu) on mobile phone or computer (https://jinlingshu.com/). Randomization will be stratified by participating centres permutation block size of 4.
**Treatments**	Patients will be assigned to receive either a placebo or methylprednisolone, with a dosage of 2mg/kg (based on estimated or actual weight if known, not exceeding a maximum dose of 160 mg) per day for a duration of 3 days. The initial

	study drug will be administered as soon as possible after randomisation. It is recommended that the initial study drug administrated before arterial access closure, but it should not be delayed more than 2 hours after arterial access closure.
**Consent**	Explicit written, signed informed consent from the subject or legally authorized representative will be obtained prior to any protocol specific procedures.
**Criteria for Evaluation-Efficacy**	Primary Efficacy Endpoint<br>● Reduced disability level (ordinal shift in mRS score) at 90 ±7 days;<br>Secondary Efficacy Endpoints<br>● Proportion of patients with mRS score 0 to 4 at 90 ±7 days;<br>● Proportion of patients with mRS score 0 to 3 at 90 ±7 days;<br>● Proportion of patients with mRS score 0 to 2 at 90 ±7 days;<br>● Proportion of patients with mRS score 0 to 1 or returned to pre-stroke mobidity at 90 ±7 days;<br>● NIHSS score at 5-7 days or at early discharge;<br>● Health-related quality of life [European Quality of Life Five-Dimension visual-analogue scale (EQ-5D VAS)] at 90 ±7 days.
**Criteria for Evaluation-Safety**	Primary Safety Endpoints<br>● Mortality at 90 ±7 days;

	● Proportion of patients with symptomatic intracranial haemorrhage within 48 hours. Symptomatic intracranial haemorrhage will be adjudicated by an independent Imaging Core Laboratory according to the modified Heidelberg Bleeding Classification;<br>Secondary Safety Endpoints<br>● Proportion of patients with any radiologic intracranial haemorrhage within 48 hours;<br>● Proportion of patients with pneumonia;<br>● Proportion of patients with gastrointestinal haemorrhage within 7 days after EVT;<br>● Incidence of serious adverse events.<br>● Incidence of any adverse events.
**Sample Size Calculation**	The current trial is designed detect a shift on the modified Rankin Scale score, which represents the global disability, to a lower score. The expected distribution of the modified Rankin Scale score, derived from the EVT for acute anterior circulation ischemic stroke (ACTUAL) registry and the Highly Effective Reperfusion evaluated in Multiple Endovascular Stroke Trials (HERMES) collaboration, is as follows: 7%, 20%, 16%, 11%, 13%, 8% and 25%.

	The sample size was calculated using the cumulative proportion of patients with mRS 0-2. We assume a moderate effect of 7% absolute increase in the cumulative proportion of patients with mRS 0-2 in the intervention group, compared with controls, indicating an odds ratio (OR) of 1.33, which have substantially exceeds the minimal clinically important difference.10 In order to demonstrate the expected treatment effect with a type-1 error alpha =0.05 (two-tailed) and a power of 80% (beta = 20%). A sample size of n= 1588 patients (n=794 per treatment group) is required.<br>The intention-to-treat principle will be applied to the primary analysis and, therefore, to safeguard against dilution of the treatment effect associated with an approximate 5% non-adherence rate (due to loss to follow-up, consent withdrawal and other reasons), we initially planned to enrol n=1672 patients (n=836 per treatment group) for this study. This estimation was performed based on PASS (NCSS, LLC. Kaysville, Utah, USA) version 15.0.
**Statistical Methods**	All efficacy analyses will be conducted on data from all randomly assigned patients according to the intention-to-treat principle. All efforts will be made to minimize the amount of missing data. Sensitivity analyses based on different hypotheses

	about the missingness pattern of the primary outcome will be performed to test for the robustness of the primary analysis. Analyses will also be repeated according the per protocol principle.<br>Analysis of Efficacy - primary endpoint:<br>The primary efficacy endpoint is disability evaluated 90 (±7) days after randomization using the modified Rankin Scale (mRS) score. The primary treatment effect will be estimated as common odds ratio or using assumption-free method.<br>Covariable were age, baseline NIHSS score, pre-stroke mRS, baseline ASPECTS score, use of intravenous thrombolysis, time from onset to randomization, and occlusion location.<br>Analysis of Efficacy - Secondary endpoint:<br>Proportion of with mRS score 0 to 4 at 90 ±7 days and proportion of mRS score 0 to 3 at 90 ±7 days, proportion of patients with mRS score 0 to 2 at 90 ±7 days, proportion of mRS score 0 to 1 or return to pre-morbid mRS score at 90 days (for patients with mRS >1). Between-group differences will be tested using generalized linear models. The risk ratios and corresponding 95%CI will be provided.<br>NIHSS score at 5-7 days or at early discharge and Health-related quality of life (EQ-5D VAS). The results will be

	compared between two randomized treatment groups using GLM. The mean difference and corresponding 95%CI will be provided. Normality and variance homogeneity of residuals will be accessed graphically. The win ratio method will be used if normality assumption is seriously violated.<br>Analysis of Safety:<br>The safety analysis will be conducted on the Safety Population. Mortality due to any cause at 90 ±7 days. The difference of mortality in two treatment groups will be tested using modified Poisson Regression. In addition, the Kaplan-Meier method will be used to assess the mortality. The log-rank test will be applied to compare the two treatment groups. Cox regression model will be employed to calculate the hazard ratio with 95%CI.<br>In addition to mortality due at 90 ±7 days, SICH within 48 hours is considered to be another primary safety outcome. SICH will be evaluated according to the modified Heidelberg Bleeding Classification. Besides, modified Poisson regression will be fitted to estimate the risk ratio associated with the treatment effect. The proportion of Any radiologic ICH within 48 hours between methylprednisolone and placebo groups will be tested in the same manner as SICH.<br>Proportion of patients with pneumonia and proportion of

	patients with gastrointestinal haemorrhage within 7 days after EVT will be explore and tested the same as SICH and any ICH. Adverse events (AE) and serious adverse event (SAE) will be summarized and presented by treatment group for all patients in the Safety Population. The incidences will be compared via Fisher's exact test or chi-square test as appropriate.

2. Flow chart and schedule of assessment

2.1 Flow Chart

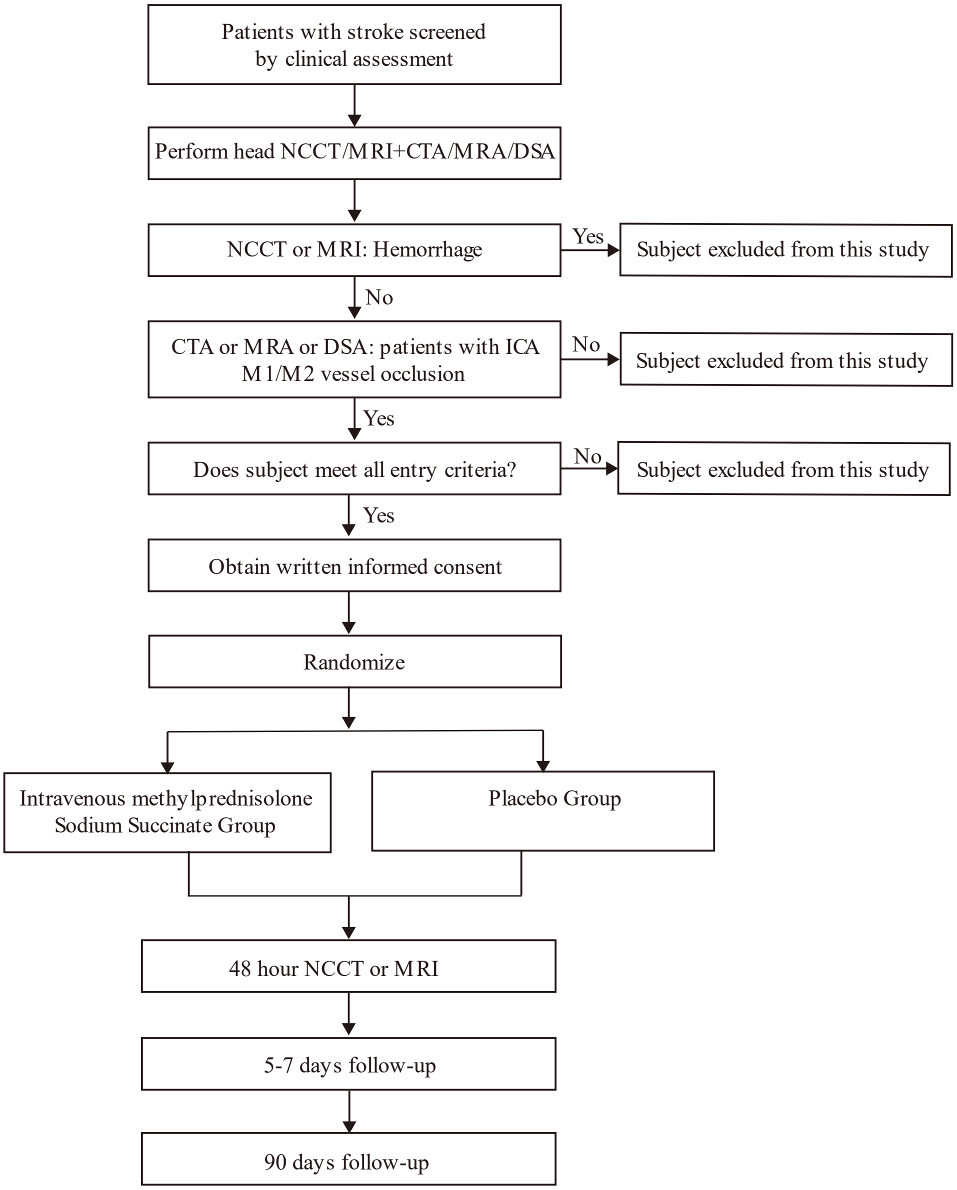

2.2. Schedule of Assessments

	**Visit (Time in hours / days from EVT =V1)**					
	Screening		Treatment		Follow Up	
	**V0**	**R**	**V1**[a] Study medication administration	**V2** (48-96h)	**V3**[b] (5-7d)	**V4** (90±7d)
Informed consent	X					
Demographic data	X					
Medical history	X					
Physical examination	X			X	X	
Pre-stroke mRS	X					
NIHSS	X				X	
mRS						X
EQ-5D						X
Previous medication	X					
Blood pressure and heart rate	X		X	X[e]	X	
Local laboratory result	X			X		
Pregnancy test[c]	X					

Brain CT/MRI plus CTA/MRA/DSA	X					
Brain CT/MRI scan[d]				X		
Inclusion / exclusion criteria	X					
Randomization		X				
Study medication			X			
Concomitant medication	X		X	X	X	X
12-lead ECG	X					
48h monitoring			X[a]			
Adverse events			X	X	X	X

V = Visit; R = Randomization; h = hour; d = day

[a] 48h monitoring in Unit or equivalent unit including repeated measurements of blood pressure, heart rate and body temperature

[b] or hospital discharge if < 5 days

[c] Mandatory for women of childbearing potential

[d] also to be performed in any case of neurological deterioration during the first 96h

[e] every hour

3. List of Abbreviations

AE	Adverse Event
AIS	Acute Ischemia Stroke
BI	Barthel Index
CRF	Case Report Form
CT	Computed Tomography
CTA	Computed Tomography Angiography
DSMB	Data Safety Monitoring Board
EAB	Ethics Advisory Board
ECG	Electrocardiogram
EQ-5D	European Quality Five-Dimension
EVT	Endovascular Therapy
GCP	Good Clinical Practice
GOS	Glasgow outcome scale
HBC	Heidelberg bleeding classification
ICH	Intracerebral Haemorrhage
ICH-GCP	International Conference on Harmonization-Good Clinical Practice
IRB	Institutional Review Board
ITT	Intention-to-Treat
IVT	Intravenous Thrombolysis
LVO	Large Vessel Occlusion
MARVEL	MARVEL: Methylprednisolone for acute large vessel occlusion: a

	randomized double-blind, placebo-controlled trial in revascularization patients
MedDRA	Medical Dictionary for Regulatory Activities
MR	Magnetic Resonance
MRA	Magnetic Resonance Angiography
mRS	modified Rankin Scale
NIHSS	National Institutes of Health Stroke Scale
NINDS	National Institute of Neurological Disorders and Stroke
PI	Principal Investigator
PP	Per-Protocol
REB	Research Ethics Board
RISS	Rapidly improving stroke symptoms
RR	Risk ratio
rt-PA	recombinant tissue Plasminogen Activator
SAB	Scientific Advisory Board
SAE	Serious Adverse Event
SAP	Statistical Analysis Plan
SICH	Symptomatic Intracerebral Haemorrhage
SOC	System Organ Class

4. Background

4.1 The burden of stroke

Stroke remains the second-leading cause of death and the third-leading cause of death and disability combined in the world.[1-3] The estimated global cost of stroke is over US $721 billion (0.66% of the global GDP).[3] In the past 20 years, the burden increased substantially, with the bulk of the global stroke burden residing in lower-income and lower-middle-income countries.[3] Recently, the National Epidemiological Survey of Stroke in China (NESS-China) suggested that the current stroke prevalence (1115/100 000 [95%CI, 997–1233]) in China appears to be the highest among other low- to middle-income countries (range from 536 to 1040/100 000), but significantly lower than that observed in high-income countries (range from 2600 to 8000/100 000). [4]

4.2 Treatment in acute ischemic stroke

Stroke is normally classified as either ischemic or haemorrhagic stroke although 62.4% of cases belong to ischemic nature. Ischemic stroke is characterized by a high incidence, high disability rate, and high mortality rate, and has become the leading cause of death in China.[4] Currently, there are about 2.4 million new stroke patients and 1.1 million deaths in China each year, imposing a heavy burden on society and families.[4] Approximately 40% of acute ischemic strokes are caused acute large vessel occlusion (LVO), with anterior circulation LVO accounting for about 80% and posterior circulation LVO accounting for about 20%.[4]

Early restoration of blood flow and salvage of ischemic penumbra are the current theoretical basis of the treatment of AIS.[5,6] The current standard therapy for acute ischemic stroke including the intravenous thrombolysis and endovascular treatment.[6] Currently, the recanalization rate of acute anterior circulation large vessel occlusive stroke has been improved to 80%~94.0% in the endovascular treatment era.[7]

However, the successful recanalization rates have not been completely translated into clinical benefits for AIS patients, and the proportion of patients with good outcome was less than 50%, which is clinically referred to as "Futile recanalization (FR)" or "Ineffective reperfusion".[8,9] That is, successful recanalization of occluded vessels (defined as modified treatment in cerebral ischemia (mTICI) blood flow grade ≥2b) does not lead to good functional outcomes (mRS score ≥3 at 90 days). Moreover, reperfusion of the ischaemic tissue is not risk-free, often causes haemorrhagic transformation (HT), blood–brain barrier (BBB) disruption.[10-12] Besides, malignant brain oedema was a common complication that were associated with high mortality.[13,14]

Several mechanisms may contribute futile reperfusion and may be the potential treatment target for the futile reperfusion phenomenon: the non-reflow phenomenon, cerebral oedema, blood-brain barrier damage and inflammatory damage.[12]

Firstly, the "non-reflow" phenomenon refers to tissue hypoperfusion despite the timely or complete recanalization of an occluded artery.[15] This phenomenon is frequently observed in clinical practice, with a prevalence of more than half in cases of coronary

artery occlusion, and it serves as an independent predictor of adverse outcomes.[16] In the cerebral circulation, non-reflow has consistently been demonstrated in preclinical studies since first being described in 1968.[15] Non-reflow in the brain may involve post-ischemic swelling in endothelial cells, microvasculature occluded by platelet and neutrophils, interstitial oedema, and inflammatory reaction.[17] Recent experimental data further suggest that non-reflow is present during the first 24 hours after recanalization.[17] In clinical studies, the prevalence of the no-reflow phenomenon after endovascular treatment (EVT) in acute ischemic stroke was approximately 30%, and this number is expected to increase with the extension of EVT to a 24-hour time window.[17] Secondly, the endovascular treatment has a damage to BBB, and will lead to severe brain edema and haemorrhagic transformation that considerably contribute to neurological deterioration and death. [11,18] Thirdly, inflammatory mechanisms after stroke are now increasingly considered prime targets for stroke therapy since immune signals and their mediators can have both detrimental and beneficial effects at different stages of the disease process.[19,20]

Thus, due to the multi-faceted nature of the aforementioned mechanisms, single-target agents may not sufficiently intervene. Therefore, combinations of agents or multi-target agents may be necessary to address these mechanisms effectively.

4.3 Glucocorticoid in Stroke

In animal models, corticosteroids have shown the ability to reduce infarct size, regulate cerebral blood flow, enhance non-reflow phenomenon, stabilize the blood-brain barrier,

prevent angiogenic edema, and modulate immune response.[21-29], while none have been adopted through clinical trial success in acute ischemic stroke therapy.[30-32]

Several lessons are learned from prior studies. Firstly, those trials were performed in the era when reperfusion strategies were not well established. The pre-clinical study of corticosteroids has suggested that corticosteroid was effective in the transient middle cerebral artery occlusion(tMCAO) model but was ineffective in the permanent middle cerebral artery occlusion(pMCAO) model.[22] It suggested that reperfusion or not was an important condition for the effect of corticosteroids.[22,33-35] In past corticosteriod treatment trials, conducted before the advent of mechanical thrombectomy as a treatment option, it is likely that many patients failed to reperfuse; the candidate adjuvant treatments were tested in the more challenging setting of permanent rather than transient brain ischemia and thus failed to show benefit in clinical trials.[35] The STAIR (Stroke Treatment Academic Industry Roundtable) suggested testing the neuroprotective agents in the new endovascular treatment era when the reperfusion rate can be up to 90%. [33,36] [37] Secondly, most included patients with “presumed ischemic stroke” were treated in an era where reperfusion was not effective, and the sample size of patients were small in the previous trials. Only 8/24 published trials of corticosteroids in stroke were acceptable for further analysis, and these comprised woefully inadequate numbers of patients (only 466). Davis and Donnan suggested that steroid therapy for stroke be discarded prematurely. Thirdly, most trials used a high-dose and long duration of corticosteroid thus increasing the incidence of major gastrointestinal hemorrhage and infections. [30]

Therefore, there was a call to reignite the study of corticosteroids in stroke, especially in the reperfusion era. [37,38] One small sample size trial in China has studied the use of corticosteroids in the new endovascular treatment era and have yielded promising result that a short course and low-dose corticosteroid can improve the functional outcome, which was consistent with the preclinical study.[39] The surprising result indicated that low-dose and short-term corticosteroids may have an effect for patients with ischemic stroke in the new EVT era. A ray of hope comes to restudy the effect of corticosteroids in the EVT era. Trials conducted on patients who underwent endovascular treatment closely aligned with the primate model of transient middle cerebral artery occlusion, where the corticosteroid has demonstrated its efficacy. This is in contrast to the permanent middle cerebral artery occlusion model, where the corticosteroid has shown its benefit.[22]

As an intermediate-acting corticosteroid with its minimal side effects and its ability to effectively cross the blood-brain barrier, methylprednisolone is widely used in clinical practice. We propose a multicenter, prospective, randomized, double-blind, placebo-controlled trial to investigate the efficacy and safety of adjunctive methylprednisolone for patients underwent endovascular treatment. The main objective of this trial is to determine whether the early administration of methylprednisolone can improve the clinical outcomes of patients with acute anterior circulation large vessel occlusive stroke after successful recanalization.

5. Study Objectives

The purpose of MARVEL trial is to investigate the efficacy and safety of early adjunctive methylprednisolone in AIS patients underwent EVT in a multicenter, randomized, double-blind, placebo-controlled trial. The findings of MARVEL are likely to have a direct impact on clinical practice. In case of a positive result, this will provide evidence for an effective, safe, and affordable treatment in the new EVT era. Additionally, this trial will contribute high-quality evidence on the use of corticosteroids in stroke through a large-scale, multicenter trial, addressing existing gaps in current knowledge.

6. Product characteristics of methylprednisolone

The following is the summary of product characteristics for methylprednisolone provided by the Chongqing Lummy Pharmaceutical Co., Ltd., Chongqing, China.

6.1 Description of the investigational medicinal product

Methylprednisolone sodium succinate, is the sodium succinate ester of methylprednisolone, and it occurs as a white, or nearly white, odorless hygroscopic, amorphous solid. It is very soluble in water and in alcohol; it is insoluble in chloroform and is very slightly soluble in acetone.

The chemical name for methylprednisolone sodium succinate is pregna-1,4-diene-3,20dione,21-(3-carboxy-1-oxopropoxy)-11,17-dihydroxy-6-methyl-monosodium salt, (6α, 11β), and the molecular weight is 496.53. The structural formula is represented below:

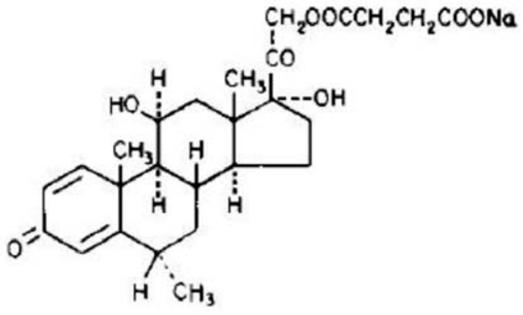

Methylprednisolone sodium succinate is soluble in water; it may be administered in a small volume of diluent and is well suited for intravenous use in situations where high blood levels of methylprednisolone are required rapidly.

CLINICAL PHARMACOLOGY

Glucocorticoids, naturally occurring and synthetic, are adrenocortical steroids that are readily absorbed from the gastrointestinal tract. Naturally occurring glucocorticoids (hydrocortisone and cortisone), which also have salt-retaining properties, are used as replacement therapy in adrenocortical deficiency states. Their synthetic analogs are primarily used for their potent anti-inflammatory effects in disorders of many organ systems. Glucocorticoids cause profound and varied metabolic effects. In addition, they modify the body's immune responses to diverse stimuli. Methylprednisolone is a potent anti-inflammatory steroid with greater anti-inflammatory potency than prednisolone and even less tendency than prednisolone to induce sodium and water retention. Methylprednisolone sodium succinate has the same metabolic and anti-inflammatory actions as methylprednisolone.

6.2 Indications and Usage

When oral therapy is not feasible, and the strength, dosage form, and route of administration of the drug reasonably lend the preparation to the treatment of the condition, the intravenous or intramuscular use of Methylprednisolone is indicated as follows:

Allergic states: Control of severe or incapacitating allergic conditions intractable to adequate trials of conventional treatment in asthma, atopic dermatitis, contact dermatitis, drug hypersensitivity reactions, perennial or seasonal allergic rhinitis, serum sickness, transfusion reactions.

Dermatologic diseases: Bullous dermatitis herpetiformis, exfoliative erythroderma, mycosis fungoides, pemphigus, severe erythema multiforme (Stevens-Johnson syndrome).

Endocrine disorders: Primary or secondary adrenocortical insufficiency (hydrocortisone or cortisone is the drug of choice; synthetic analogs may be used in conjunction with mineralocorticoids where applicable; in infancy, mineralocorticoid supplementation is of particular importance), congenital adrenal hyperplasia, hypercalcemia associated with cancer, nonsuppurative thyroiditis.

Gastrointestinal diseases: To tide the patient over a critical period of the disease in regional enteritis (systemic therapy) and ulcerative colitis. Hematologic disorders: Acquired (autoimmune) hemolytic anemia, congenital (erythroid) hypoplastic anemia (Diamond-Blackfan anemia), idiopathic thrombocytopenic purpura in adults

(intravenous administration only; intramuscular administration is contraindicated), pure red cell aplasia, selected cases of secondary thrombocytopenia.

Miscellaneous: Trichinosis with neurologic or myocardial involvement, tuberculous meningitis with subarachnoid block or impending block when used concurrently with appropriate antituberculous chemotherapy.

Neoplastic diseases: For the palliative management of leukemias and lymphomas.

Nervous System: Acute exacerbations of multiple sclerosis; cerebral edema associated with primary or metastatic brain tumor, or craniotomy.

Ophthalmic diseases: Sympathetic ophthalmia, uveitis and ocular inflammatory conditions unresponsive to topical corticosteroids. Renal diseases: To induce diuresis or remission of proteinuria in idiopathic nephrotic syndrome or that due to lupus erythematosus.

Respiratory diseases: Berylliosis, fulminating or disseminated pulmonary tuberculosis when used concurrently with appropriate antituberculous chemotherapy, idiopathic eosinophilic pneumonias, symptomatic sarcoidosis.

Rheumatic disorders: As adjunctive therapy for short-term administration (to tide the patient over an acute episode or exacerbation) in acute gouty arthritis; acute rheumatic carditis; ankylosing spondylitis; psoriatic arthritis; rheumatoid arthritis, including juvenile rheumatoid arthritis (selected cases may require low-dose maintenance therapy). For the treatment of dermatomyositis, temporal arteritis, polymyositis, and systemic lupus erythematosus.

6.3 Contraindications

The methylprednisolone sodium succinate is contraindicated:

- in systemic fungal infections and patients with known hypersensitivity to the product and its constituents.
- for intrathecal administration. Reports of severe medical events have been associated with this route of administration.

6.4 Warning

Serious Neurologic Adverse Reactions with Epidural Administration

Serious neurologic events, some resulting in death, have been reported with epidural injection of corticosteroids. Specific events reported include, but are not limited to, spinal cord infarction, paraplegia, quadriplegia, cortical blindness, and stroke. These serious neurologic events have been reported with and without use of fluoroscopy. The safety and effectiveness of epidural administration of corticosteroids have not been established, and corticosteroids are not approved for this use.

GENERAL

Increased dosage of rapidly acting corticosteroids is indicated in patients on corticosteroid therapy who are subjected to any unusual stress before, during, and after the stressful situation.

Results from one multicenter, randomized, placebo-controlled study with methylprednisolone hemisuccinate, an intravenous corticosteroid, showed an increase in early (at 2 weeks) and late

(at 6 months) mortality in patients with cranial trauma who were determined not to have other clear indications for corticosteroid treatment. High doses of systemic corticosteroids, should not be used for the treatment of traumatic brain injury.

Cardio-renal

Average and large doses of corticosteroids can cause elevation of blood pressure, salt and water retention, and increased excretion of potassium. These effects are less likely to occur with the synthetic derivatives except when used in large doses. Dietary salt restriction and potassium supplementation may be necessary. All corticosteroids increase calcium excretion. Literature reports suggest an apparent association between the use of corticosteroids and left ventricular free wall rupture after a recent myocardial infarction; therefore, therapy with corticosteroids should be used with great caution in these patients.

Endocrine

Hypothalamic-pituitary adrenal (HPA) axis suppression, Cushing's syndrome, and hyperglycemia. Monitor patients for these conditions with chronic use. Corticosteroids can produce reversible HPA axis suppression with the potential for glucocorticosteroid insufficiency after withdrawal of treatment. Drug induced secondary adrenocortical insufficiency may be minimized by gradual reduction of dosage. This type of relative insufficiency may persist for months after discontinuation of therapy; therefore, in any situation of stress occurring during that period, hormone therapy should be reinstituted.

Drug-Induced Liver Injury

Rarely, high doses of cyclically pulsed intravenous methylprednisolone (usually for the treatment of exacerbations of multiple sclerosis at doses of 1 gram/day) can induce a toxic form of acute hepatitis. The time to onset of this form of steroid-induced liver injury can be several weeks or longer. Resolution has been observed after discontinuation of treatment. However, serious liver injury can occur, sometimes resulting in acute liver failure and death. Discontinue intravenous methylprednisolone if toxic hepatitis occurs. Since recurrence has occurred after re-challenge, avoid use of high dose intravenous methylprednisolone in patients with a history of toxic hepatitis caused by methylprednisolone.

Infections

General

Patients who are on corticosteroids are more susceptible to infections than are healthy individuals. There may be decreased resistance and inability to localize infection when corticosteroids are used. Infections with any pathogen (viral, bacterial, fungal, protozoan, or helminthic) in any location of the body may be associated with the use of corticosteroids alone or in combination with other immunosuppressive agents. These infections may be mild, but can be severe and at times fatal. With increasing doses of corticosteroids, the rate of occurrence of infectious complications increases. Corticosteroids may also mask some signs of current infection. Do not use intraarticularly, intrabursally or for intratendinous administration for local effect in the presence of acute local infection. A study has failed to establish the efficacy of methylprednisolone sodium succinate in the treatment of sepsis syndrome and septic

shock. The study also suggests that treatment of these conditions with methylprednisolone sodium succinate may increase the risk of mortality in certain patients (i.e., patients with elevated serum creatinine levels or patients who develop secondary infections after methylprednisolone sodium succinate).

Fungal infections

Corticosteroids may exacerbate systemic fungal infections and therefore should not be used in the presence of such infections unless they are needed to control drug reactions. There have been cases reported in which concomitant use of amphotericin B and hydrocortisone was followed by cardiac enlargement and congestive heart failure (see CONTRAINDICATIONS and PRECAUTIONS, Drug Interactions, Amphotericin B injection and potassium-depleting agents). Special pathogens Latent disease may be activated or there may be an exacerbation of intercurrent infections due to pathogens, including those caused by Amoeba, Candida, Cryptococcus, Mycobacterium, Nocardia, Pneumocystis, Toxoplasma. It is recommended that latent amebiasis or active amebiasis be ruled out before initiating corticosteroid therapy in any patient who has spent time in the tropics or in any patient with unexplained diarrhea. Similarly, corticosteroids should be used with great care in patients with known or suspected Strongyloides (threadworm) infestation. In such patients, corticosteroid-induced immunosuppression may lead to Strongyloides hyperinfection and dissemination with widespread larval migration, often accompanied by severe enterocolitis and potentially fatal gram-negative septicemia. Corticosteroids should not be used in cerebral malaria. There is currently no evidence of benefit from

steroids in this condition.

Tuberculosis

The use of corticosteroids in active tuberculosis should be restricted to those cases of fulminating or disseminated tuberculosis in which the corticosteroid is used for the management of the disease in conjunction with appropriate antituberculous regimen. If corticosteroids are indicated in patients with latent tuberculosis or tuberculin reactivity, close observation is necessary as reactivation of the disease may occur. During prolonged corticosteroid therapy, these patients should receive chemoprophylaxis.

Vaccination

Administration of live or live, attenuated vaccines is contraindicated in patients receiving immunosuppressive doses of corticosteroids. Killed or inactivated vaccines may be administered. However, the response to such vaccines cannot be predicted. Immunization procedures may be undertaken in patients receiving corticosteroids as replacement therapy, e.g., for Addison’s disease.

Viral infections

Chicken pox and measles can have a more serious or even fatal course in pediatric and adult patients on corticosteroids. In pediatric and adult patients who have not had these diseases, particular care should be taken to avoid exposure. The contribution of the underlying disease and/or prior corticosteroid treatment to the risk is also not known. If exposed to chicken pox, prophylaxis with varicella zoster immune globulin (VZIG) may be indicated. If exposed to measles, prophylaxis with immunoglobulin

(IG) may be indicated. (See the respective package inserts for complete VZIG and IG prescribing information.) If chicken pox develops, treatment with antiviral agents should be considered.

Neurologic

Reports of severe medical events have been associated with the intrathecal route of administration (see ADVERSE REACTIONS, Gastrointestinal and Neurologic/Psychiatric).

Ophthalmic

Use of corticosteroids may produce posterior subcapsular cataracts, glaucoma with possible damage to the optic nerves, and may enhance the establishment of secondary ocular infections due to bacteria, fungi, or viruses. The use of oral corticosteroids is not recommended in the treatment of optineuritis and may lead to an increase in the risk of new episodes. Corticosteroids should be used cautiously in patients with ocular herpes simplex because of corneal perforation. Corticosteroids should not be used in active ocular herpes simplex.

PRECAUTIONS

General

This product, like many other steroid formulations, is sensitive to heat. Therefore, it should not be autoclaved when it is desirable to sterilize the exterior of the vial. The lowest possible dose of corticosteroid should be used to control the condition under treatment. When reduction in dosage is possible, the reduction should be gradual. Since complications of treatment with glucocorticoids are dependent on the size of the

dose and the duration of treatment, a risk/benefit decision must be made in each individual case as to dose and duration of treatment and as to whether daily or intermittent therapy should be used. Kaposi's sarcoma has been reported to occur in patients receiving corticosteroid therapy, most often for chronic conditions. Discontinuation of corticosteroids may result in clinical improvement.

Cardio-renal

As sodium retention with resultant edema and potassium loss may occur in patients receiving corticosteroids, these agents should be used with caution in patients with congestive heart failure, hypertension, or renal insufficiency.

Endocrine

Drug-induced secondary adrenocortical insufficiency may be minimized by gradual reduction of dosage. This type of relative insufficiency may persist for months after discontinuation of therapy; therefore, in any situation of stress occurring during that period, hormone therapy should be reinstituted. Metabolic clearance of corticosteroids is decreased in hypothyroid patients and increased in hyperthyroid patients. Changes in thyroid status of the patient may necessitate adjustment in dosage.

Gastrointestinal

Steroids should be used with caution in active or latent peptic ulcers, diverticulitis, fresh intestinal anastomoses, and nonspecific ulcerative colitis, since they may increase the risk of a perforation. Signs of peritoneal irritation following gastrointestinal perforation in patients receiving corticosteroids may be minimal or absent. There is an enhanced effect due to decreased metabolism of corticosteroids in

patients with cirrhosis.

Musculoskeletal

Corticosteroids decrease bone formation and increase bone resorption both through their effect on calcium regulation (i.e., decreasing absorption and increasing excretion) and inhibition of osteoblast function. This, together with a decrease in the protein matrix of the bone secondary to an increase in protein catabolism, and reduced sex hormone production, may lead to inhibition of bone growth in pediatric patients and the development of osteoporosis at any age. Special consideration should be given to patients at increased risk of osteoporosis (i.e., postmenopausal women) before initiating corticosteroid therapy. Local injection of a steroid into a previously infected site is not usually recommended.

Neurologic-Psychiatric

Although controlled clinical trials have shown corticosteroids to be effective in speeding the resolution of acute exacerbations of multiple sclerosis, they do not show that corticosteroids affect the ultimate outcome or natural history of the disease. The studies do show that relatively high doses of corticosteroids are necessary to demonstrate a significant effect. An acute myopathy has been observed with the use of high doses of corticosteroids, most often occurring in patients with disorders of neuromuscular transmission (e.g., myasthenia gravis), or in patients receiving concomitant therapy with neuromuscular blocking drugs (e.g., pancuronium). This acute myopathy is generalized, may involve ocular and respiratory.

7. Organizational Structure

7.1 Funding

MARVEL trial is an investigator-initiated study which is organized by the second affiliated hospital of the Army Medical University and conducted in 82 centres in China. MARVEL will be funded by the National Natural Science Foundation of China. Methylprednisolone and its placebo are manufactured and provided by Chongqing Lummy Pharmaceutical Co., Ltd., Chongqing, China. The funder had no involvement in the study design, data collection, analysis and interpretation, writing or decision to submit the paper.

7.2 Trials Boards and Committees

The following boards and institutions will assure the success of the clinical trial:

7.2.1 Steering Committee

The Steering Committee of the clinical trial will decide on the final protocol and oversee the trial.

7.2.2 Data Safety and Monitoring Board

The independent Date and Safety Monitoring Board (DSMB) will regularly monitor the safety of the trial and ensure the safety of patients at all stages of MARVEL. The independent DSMB will be composed of an experienced neurologist, a neuroradiologist, and a biostatistician, who are not participants of the MARVEL consortium and not involved in the clinical trial in any other way. The DSMB may at

any time propose to revise the clinical trial protocol or terminate the trial in case of safety concerns.

7.2.3 Ethics Advisory Board

The Ethics Advisory Board (EAB) will supervise the trial and ensure that the trial is conducted in accordance with the ICH-GCP guidelines and international and China legislation. The EAB may at any time propose to revise the clinical trial protocol or terminate the trial in case of ethical concerns.

7.2.4 Imaging Core Laboratory

Centralized imaging core laboratories will centrally blinded review all images and provide reference judgements for the definition of the per protocol population and intracranial haemorrhages. CT/MR and angiographic images will be independently reviewed by two independent central imaging core laboratories respectively. Imaging Core Laboratory will be independent to ensure it is blinded to the treatment allocation.

7.2.5 Clinical Events Committee

The Clinical Events Committee will be comprised of three expert physicians independent of the investigational sites. This committee will validate all the complications that occur over the course of the study and categorize each for severity and relatedness according to the definition in the AE section. The Clinical Events Committee can request any additional source information and images supporting the AEs to assist with the adjudication.

8. Design

MARVEL is a multicenter, randomized, double-blind, placebo-controlled trial. Patients will be randomized 1:1 to either Methylprednisolone Group or Placebo Group. The study has been registered at Chinese Clinical Trial Registry (www.chictr.org.cn, unique identifier ChiCTR2100051729).

9. Population

The patients for this trial will be recruited from AIS patients with large vessel occlusion within 24 hours from last known well.

9.1 Inclusion criteria

- Age ⩾ 18 years;
- The time from onset to randomization was within 24 hours;
- Anterior circulation ischemic stroke was preliminarily determined according to clinical symptoms or imaging examination;
- Baseline National Institutes of Health Stroke Scale (NIHSS) ⩾ 6
- Baseline Alberta Stroke Program Early CT Score (ASPECTS) ⩾ 3;
- Computed tomography angiography (CTA) /magnetic resonance angiography (MRA) /digital subtraction angiography (DSA) confirmed occlusion of intracranial segment of internal carotid artery and middle cerebral artery, and decided to undergo endovascular therapy;

- Written informed consent signed by patients or their family members.

9.2 Exclusion Criteria

Patients meeting any of the following criteria will be excluded from study enrolment.

- Intracranial hemorrhage confirmed by cranial computed tomography (CT) or magnetic resonance imaging (MRI);
- mRS score ≥ 2 before onset;
- Pregnant or lactating women;
- Allergic to contrast agents;
- Allergic to glucocorticoids;
- Participating in other clinical trials;
- Systolic blood pressure > 185 mmHg or diastolic pressure > 110 mmHg, and oral antihypertensive drugs can not control;
- Genetic or acquired bleeding constitution, lack of anticoagulant factors; Or oral anticoagulants and INR > 1.7;
- Blood sugar < 2.8 mmol/L (50 mg/dl) or > 22.2 mmol/L (400 mg/dl), platelet < 90 x 10^9/L;
- The artery is tortuous so that the thrombectomy device cannot reach the target vessel;
- Bleeding history (gastrointestinal and urinary tract bleeding) in recent 1 month;

- Chronic hemodialysis and severe renal insufficiency (glomerular filtration rate < 30 ml/min or serum creatinine > 220 umol/L [2.5 mg/ dL]);
- Life expectancy due to any advanced disease < 6 months;
- Follow-up is not expected to be completed;
- Intracranial aneurysm and arteriovenous malformation;
- Brain tumors with imaging mass effect;
- Severe systemic infectious diseases.

9.3 Sample Size

The current trial is designed detect a shift on the modified Rankin Scale score, which represents the global disability, to a lower score. The expected distribution of the modified Rankin Scale score, derived from the EVT for acute anterior circulation ischemic stroke (ACTUAL) registry[40] and the Highly Effective Reperfusion evaluated in Multiple Endovascular Stroke Trials (HERMES) collaboration[7], is as follows: 7%, 20%, 16%, 11%, 13%, 8% and 25%.

The sample size was calculated using the cumulative proportion of patients with mRS 0-2. We assume a moderate effect of 7% absolute increase in the cumulative proportion of patients with mRS 0-2 in the intervention group, compared with controls, indicating an odds ratio (OR) of 1.33, which have substantially exceeds the minimal clinically important difference.[41,42] In order to demonstrate the expected

treatment effect with a type-1 error alpha =0.05 (two-tailed) and a power of 80% (beta = 20%). A sample size of n= 1588 patients (n=794 per treatment group) is required.

The intention-to-treat principle will be applied to the primary analysis and, therefore, to safeguard against dilution of the treatment effect associated with an approximate 5% non-adherence rate (due to loss to follow-up, consent withdrawal and other reasons), we initially planned to enrol n=1672 patients (n=836 per treatment group) for this study. This estimation was performed based on PASS (NCSS, LLC. Kaysville, Utah, USA) version 15.0.

10. Imaging Training

Investigators involved in image reading within the trial will participate in a standardized image reading training before study start.

11. Randomization

Randomization will be done by a web-based APP (Jinlingshu) on mobile phone or computer (https://jinlingshu.com/). The automated system will assign an appropriate set of study medication to each patient. Eligible patients are randomized to treatment with either intravenous methylprednisolone or placebo with a ratio of 1:1. Randomization will be stratified by participating centre with permutation block size of 4. Randomization will be completely concealed by having both web-based real-time allocation and identical appearance of methylprednisolone sodium succinate and placebo bottles. (All bottles will have a unique number. Subjects will be assigned a

random serial number according to the time they were enrolled, and corresponding masked medications will be provided).

The randomization list will be prepared by the independent statistical centre using SAS 9.4. It will only be sent to the data coordination centre responsible for central randomization and data management, the pharmacy centre responsible for treatment packaging.

12. Blinding/Unblinding

Both patient and investigator are blinded to treatment assignment. If there is a clinical situation that the investigator believes it is necessary to unblind for the safety of patients, the medical monitor must be immediately notified to discuss the intended unblinding. Premature breaking of the blind should be restricted to the setting where identification of the type of treatment is critical for adequate treatment of the patient. All unblinded patients will remain in the study and complete all follow-up visits.

If the site principal investigator wants to know the identity of the study drug for any other reason, he or she must call the medical monitor. Unless the circumstances are deemed necessary by site principal investigator, treatment codes should not be broken. The site principal investigator should document and make an explanation for any premature unblinding.

For the purpose of regulatory reporting, if required by the local health authorities, treatment codes could be broken for all serious, unexpected suspected adverse reactions that are deemed by the investigator to be related to study drug.

13. Study Treatment

13.1 Formulation, Packaging, and Handling

Methylprednisolone sodium succinate and its placebo are manufactured by Lummy Pharmaceutical Group Co., Ltd., Chongqing, China. All study medication will be manufactured, tested, released, and shipped according to Good Manufacturing Practice guidelines. Labelling and packaging of study medication will be conducted according to Good Clinical Practice and Good Manufacturing Practice guidelines, and any national regulatory requirements.

Methylprednisolone sodium succinate and its corresponding placebos are provided in numbered and are visually identical, except for a unique number. Methylprednisolone sodium succinate and it's placebo will be packed in glass bottle of identical appearance. Each kit has a unique identification number and will be stored in a safe location at room temperature on the clinical site with limited access.

13.2 Test Product

Each kit of the methylprednisolone group contains l2 bottles, and each bottles contains 40mg methylprednisolone sodium succinate, labelled for "Study Drug" and "clinical trial use only"). Each kit will be stored in a safe location at room temperature (25°C with excursions permitted between 15°C−30°C) on the clinical site with restricted access.

13.3 Comparator

Each kit of the placebo group contains l2 bottles, and each bottles contains 40mg placebo, labelled for “Study Drug” and “clinical trial use only”). Each kit will be stored in a safe location at room temperature (25°C with excursions permitted between 15°C−30°C) on the clinical site with restricted access.

13.4 Dosage, Administration, and Compliance

Eligible patients will be randomly assigned a number corresponding to a blinded sealed medication kit that sent to each patient. It is recommended to start using the study drug within 15 minutes after randomization.

13.4.1 Methylprednisolone and its Placebo

Patients will be assigned to receive either a placebo or methylprednisolone, with a dosage of 2mg/kg (based on estimated or actual weight if known, not exceeding a maximum dose of 160 mg) per day for a duration of 3 days. The initial study drug will be administered as soon as possible after randomisation. It is recommended that the initial study drug administrated before arterial access closure, but it should not be delayed more than 2 hours after arterial access closure. All patients will be treated according to the current Chinese Stroke Association guidelines for clinical management of cerebrovascular disorders[43]. Risk factors such as diabetes mellitus, hypertension, hyperlipidaemia, hyperhomocysteinaemia, obesity, drinking and smoking should be managed appropriately.

13.4.2 Concomitant therapy

Administration of any other (intravenous or oral) corticosteroids (E.g.,

Hydrocortisone) is not allowed during the first 72 hours post randomization. Gastrointestinal prophylaxis and hyperglycemia treatment will be administered per standard local protocols. Any concomitant medication within one week prior to screening through the study completion/discontinuation visit should be documented in the Concomitant Medications Case Report Form.

13.4.4 Drug Accountability

The investigational sites will be provided with sufficient amounts of investigational medicinal products. The investigational medicinal products must not be used outside the study protocol. The investigator or authorized staff is obliged to acknowledge receipt of the study medication to confirm the content and shipping temperature. Any damaged shipment of study medications will be replaced. The study drug will be disposed of in accordance with the standard operating procedures at the study site, or returned to the sponsor with appropriate documents. The investigational site's method of study medication destruction must be agreed to by the Sponsor.

Before destroying any study medications, the investigational site must obtain the written authorization from the sponsor and document the destruction of study medications in an appropriate form. Accurate records of receipt, dispensation, return and disposition of all study medication during the study should be documented on the drug inventory log.

14. Study Schedule

The schedule of assessments conducted during the study is shown in Part 2 of Chapter

2. Flow Chart and Schedule of Assessments. The study includes three periods: Screening (V0), treatment (V1, V2) and Follow up (V3, V4).

14.1 Screening period (Visit V0, Randomization)

14.1.1 Visit V0

For each eligible patient, written informed consent for participation in the study will be obtained from the patient or the patient's legal representative prior to the performance of any protocol-related investigation. Once written informed consent is obtained, the results of the following standard of care assessments will be used for the study. The investigator will record the details of all patients consented to confirm eligibility and record the reasons for screening failure.

- Demographics
- Medical history
- Physical examination
- Determination of the pre-stroke mRS via interview of the patient or the patient's kin
- NIHSS score (Note: performed by an NIHSS-certified practitioner)
- Collection of information on previous medications
- Measurement of blood pressure and heart rate
- Pregnancy test (urine test, in non-menopausal women)

- Laboratory tests
- A 12-lead ECG
- Non-contrast CT scan or MRI scan and CTA, MRA, or DSA
- Concomitant medications
- Checking of inclusion and exclusion criteria

14.1.2 Randomization (R)

If all inclusion criteria are met and no exclusion criteria are present, randomization will be performed.

14.2 Treatment period (Visits V1，V2)

- After randomization, the treatment period starts, including study medication (V1) and post-treatment clinical examination (V2).

14.2.1 Visit 1

Before administration of the study medication, measurement of blood pressure and heart rate have to be performed. The study medication will be administered as specified for intravenous methylprednisolone. During the administration of study medication, the following assessments will be made:

- Measurement of blood pressure and heart rate every hour.
- Documentation of any concomitant medication.
- Assessment of Adverse Event.

- In any case of significant neurological deterioration judged by the investigator, an assessment of the neurological deficit and another non-contrast cranial CT scan or MRI scan have to be performed to determine whether intracranial haemorrhage occurs.
- The 48h monitoring should be performed in Stroke Unit or equivalent unit.

14.2.2 Visit 2

- Visit 2 comprises the clinical examination and a second non-contrast cranial CT or MRI scan which is performed to diagnose intracranial haemorrhage. Visit 2 will be done 48-96h after the administration of study medication. The following assessments will be performed:
- Physical examination
- Assessment of the neurological deficit by the NIHSS
- Measurement of blood pressure and heart rate
- Laboratory tests
- Non-contrast cranial CT or MRI scan
- Documentation of any concomitant medication
- Assessment of Adverse Event

14.3 Follow Up period (Visits V3, V4)

The Follow Up period comprises the period of the subacute stage (day 5-7 or hospital

discharge) in Visit 3， and the final follow up examination 90±7 days in Visit 4.

14.3.1 Visit 3

The following assessments will be performed:

- Physical examination
- Assessment of the neurological deficit by the NIHSS
- Measurement of blood pressure and heart rate
- Documentation of any concomitant medication
- Assessment of Adverse Event

14.3.3 Visit 4

The following assessments will be performed:

- Physical examination (If the patients return to local hospital)
- Assessment of the neurological deficit by the NIHSS
- Assessment of functional status by the mRS
- Assessment of functional health status and quality of life by the EQ-5D scale
- Documentation of any concomitant medication
- Assessment of Adverse Event

15. Endpoints of the Clinical Trial

15.1 Efficacy Analysis-Primary Endpoint

The primary outcome was the ordinal mRS score, a global measure of disability, comprises of seven grades ranging from 0 (no symptoms) to 6 (death).

15.2 Efficacy Analysis-Secondary Endpoints

- Proportion of patients with mRS score 0 to 4 at 90±7 days;
- Proportion of patients with mRS score 0 to 3 at 90±7 days;
- Proportion of patients with mRS score 0 to 2 at 90±7 days
- Proportion of patients with mRS score 0 to 1(or returned to pre-stroke modified Rankin scale score) at 90±7 days
- NIHSS score at 5-7 days after EVT or at early discharge;
- European Quality Five-Dimension scale score at 90 days.

15.3 Safety Analysis-Primary Endpoint

15.3.1 Primary Safety Endpoints

- Mortality due to any cause at 90 (±7) days;
- Proportion of patients with symptomatic intracranial haemorrhage within 48 hours after endovascular treatmen. Symptomatic intracranial haemorrhage will be adjudicated by an independent Imaging Core Laboratory and the Clinical Events Committee according to the modified Heidelberg Bleeding Classification, and is diagnosed based on a combination of: 1) clinical deterioration, 2) imaging findings, and 3) causal relatedness assessment. Clinical deterioration is defined as any of the following conditions: 1) NIHSS score increased more than 4 points than that

immediately before worsening; 2) NIHSS score increased more than 2 points in one category; 3) Deterioration led to intubation, hemicraniectomy, external ventricular drain placement or any other major interventions. The imaging criteria are presence of parenchymal hematoma type 2 (PH2), parenchymal hematoma type 1 (PH1), remote intracerebral haemorrhage (RIH), subarachnoid haemorrhage (SAH), intraventricular haemorrhage (IVH), or subdural haemorrhage. When clinical deterioration and PH2 co-occur, the event is automatically categorized as SICH. When clinical deterioration and PH1/RIH/SAH/IVH/SDH co-occur, the event is deemed SICH if it is judged that the haemorrhage contributed substantially to the clinical worsening. When clinical deterioration occurs only with haemorrhagic infarction type 1 or 2 (HI1 or HI2), the event is automatically classified as not SICH. ICH.

15.3.2 Secondary Safety Endpoints

- Proportion of patients with any radiologic intracranial haemorrhage within 48 hours after treatment;
- Proportion of patients with pneumonia;
- Proportion of patients with gastrointestinal haemorrhage within 7 days after EVT;
- Incidence of serious adverse events.
- Incidence of any adverse events.

16. Assessment

16.1 Assessment of efficacy

16.1.1 The modified Rankin Scale (mRS)

The mRS score is a valid and reliable clinician-reported measure of global disability that has been widely applied for evaluating recovery from stroke. It is a scale used to measure functional recovery (the degree of disability or dependence in daily activities) of people who have suffered a stroke[44,45]. mRS scores range from 0 to 6, with 0 indicating no residual symptoms; 5 indicating bedbound, requiring constant care; and 6 indicating death. The mRS score will be obtained at

Day 90. Premorbid mRS status will also be obtained retrospectively and reported on the 24 Hours CRF page. In this trial, we will keep video and voice recording versions of mRS score except those who die, unable, or refuse to take a video. For mRS score at Day 90, the mRS score will be assessed by two independent certified neurologists in a blinded manner with the video combined with voice record. For those who decline to participate in a video recording, the outcomes will be determined in person by site neurologists blinded to the treatment assignment, and keep a chat record with site neurologists. Disagreements are resolved by consensus.

The mRS will only be scored by those who have been trained and certified to use this scale using the table below. (See appendix 1)

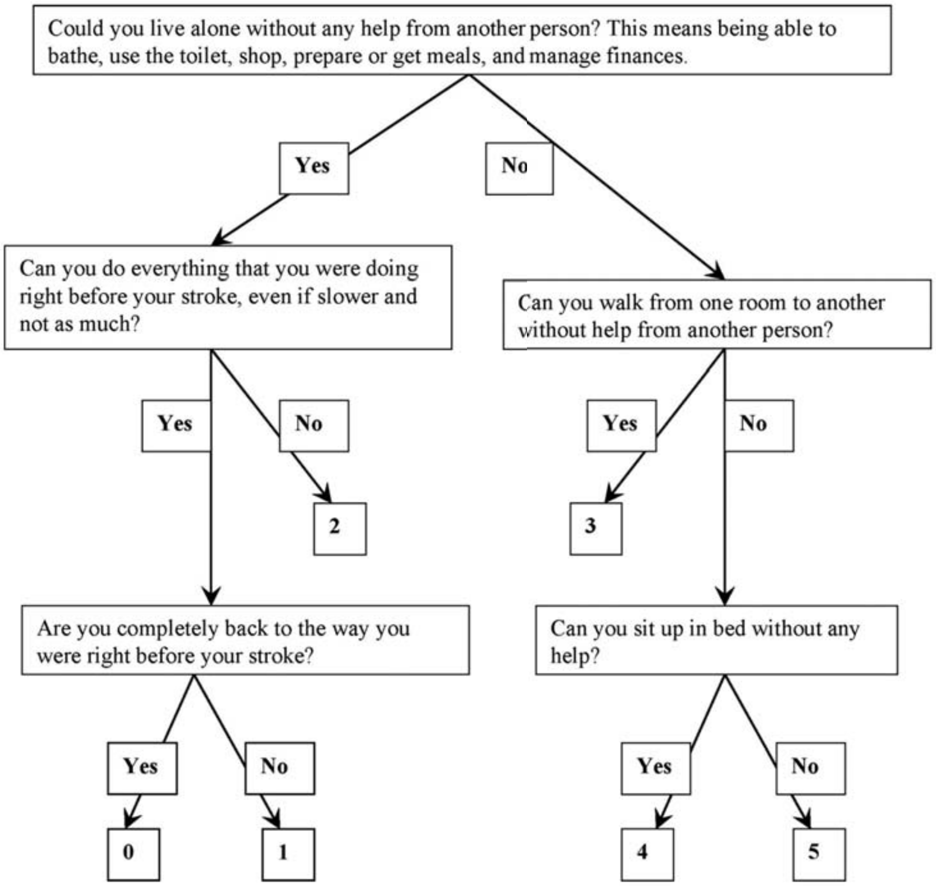

16.1.2 The National Institutes of Health Stroke Scale (NIHSS)

The NIHSS is a standardized neurological examination score that is a valid and reliable measure of disability and recovery after acute stroke[46]. Scores range from 0 to 42, with higher scores indicating more severe disability. The scale includes measures of level of consciousness, extra ocular movements, motor and sensory tests, coordination, language and speech evaluations. The NIHSS will be administered at baseline, Day 5-7 or discharge if earlier. The NIHSS will only be scored by those trained and certified in the use of this scale. In this trial, we will keep a video version of NIHSS score except in those who die or are unable or refuse to take a video. The

NIHSS score will be centrally assessed by two independent certified neurologists in a blinded manner via the video. Disagreements are resolved by consensus.(See appendix 2)

16.1.3 European Quality Five Dimensions Five Level scale

The EQ-5D-VAS is a generic instrument for describing and valuing health. The instrument is designed for self-completion, and respondents also rate their overall health on the day of the interview on a 0-100 hash-marked, vertical visual analogue scale. Patients who died will have a score of 0. The EQ-5D-VAS will be administered on Day 90±7 by those trained in the use of this scale. (See appendix 3)

16.2 Assessment of safety

16.2.1 Mortality at 90±7 days

Mortality at 90±7 days is one of the most important safety endpoints of the MARVEL trial. Mortality rates are defined as the number of deaths observed divided by the number of subjects observed over the 90-day study period.

16.2.2 Symptomatic Intracranial Haemorrhage

In addition to mortality, we will investigate SICH being the most feared complication for acute ischemic stroke. SICH within 48 hours will be adjudicated by an independent Imaging Core Laboratory and the Clinical Events Committee according to the Heidelberg Bleeding Classification.[47] SICH is diagnosed based on a combination of: 1) clinical deterioration, 2) imaging findings, and 3) causal

relatedness assessment. Clinical deterioration is defined as any of the following conditions: 1) NIHSS score increased more than 4 points than that immediately before worsening; 2) NIHSS score increased more than 2 points in one category; 3) Deterioration led to intubation, hemicraniectomy, external ventricular drain placement or any other major interventions. The imaging criteria are presence of parenchymal hematoma type 2 (PH2), parenchymal hematoma type 1 (PH1), remote intracerebral haemorrhage (RIH), subarachnoid haemorrhage (SAH), intraventricular haemorrhage (IVH), or subdural haemorrhage (SDH). When clinical deterioration and PH2 co-occur, the event is automatically categorized as SICH. When clinical deterioration and PH1/RIH/SAH/IVH/SDH co-occur, the event is deemed SICH if it is judged that the haemorrhage contributed substantially to the clinical worsening. When clinical deterioration occurs only with haemorrhagic infarction type 1 or 2 (HI1 or HI2), the event is automatically classified as not SICH.

16.2.3 Any ICH within 48 hours

The statistical analysis of differences in proportions of patients with any radiologic ICH within 48 hours between Methylprednisolone and Placebo groups will be performed.

The central reading will classify ICH according to the following intracerebral subtypes:

- Haemorrhagic infarct type 1 or type 2;
- Parenchymal hematoma type 1 or type 2;

- Remote intraparenchymal haemorrhage;
- In addition, intraventricular haemorrhage, subarachnoid haemorrhage, subdural haemorrhage, or epidural haemorrhage will be noted.

16.2.4 Proportion of patients with pneumonia

Pneumonia is considered when at least 2 signs (body temperature > 38°C; leukocytosis >12 000/mL, or leukopenia <4000/mL; purulent pulmonary secretions) associated with the appearance of a new infiltrate are present or when changes occur in an existing infiltrate on chest x-ray. If necessary, the diagnosis will be confirmed by a respiratory tract sample using a quantitative culture with a predefined positive threshold of 10^4 colony-forming units per milliliter (CFU/mL) for a bronchoalveolar lavage or non bronchoscopic sample, of 10^3 CFU/mL for a protected specimen brush and of 10^6 CFU/mL for a tracheal sample. The incidence of pneumonia will be reported by local investigators.

16.2.4 Proportion of patients with gastrointestinal bleeding.

The statistical analysis of differences in proportions of patients with gastrointestinal bleeding within 7 days between Methylprednisolone and Placebo groups will be performed. Gastrointestinal bleeding will be reported by local investigators.

16.2.5 Adverse events

The safety assessments will include monitoring and recording adverse events, including serious and non-serious adverse events, measuring vital signs specified in

the protocol, and other protocol specified tests that are considered to be critical to the safety evaluation of the study.

Any adverse change in health or the appearance of or worsening of any undesirable sign, symptom or medical condition occurring after enrolment into the trial will be recorded as Adverse Event whether or not it is considered to be related to the study drug. An adverse event also includes any occurrence that is a new illness; aggravated in severity of frequency from the baseline condition, abnormal results of diagnostic procedures, or a combination of the above. The assessment of severity and relationship of adverse events are determined by using the following definitions.

Table 3: Severity categorization and relationship definitions of Adverse Event.

Severity categorization		
Grade1	Mild	It is usually transient and may require only minimal treatment or therapeutic intervention. The event doesn’t generally interfere with usual activities of daily living.
Grade2	Moderate	It is usually alleviated with additional specific therapeutic intervention. The event interferes with usual activities of daily living, causing discomfort but poses no significant or permanent risk of harm to the research subject.
Grade3	Severe	It interrupts usual activities of daily living, or significantly affects clinical status, or may require intensive therapeutic intervention.

Grade4	Life-threatening	Substantial risk of dying at time of event
Grade5	Death	Death
Relationship of adverse events		
Related	A clinical event, including laboratory test abnormality, where there is a "reasonable possibility" that the serious adverse event was caused by the study medication, meaning that there is evidence or arguments to suggest a causal relationship.	
Probably	A clinical event, including laboratory test abnormality, with a reasonable time sequence to drug administration, unlikely to be attributed to concurrent disease or other drugs or chemicals, and which follows a clinically reasonable response on withdrawal.	
Possibly	A clinical event, including laboratory test abnormality, with a reasonable time sequence to drug administration, but which could also be explained by concurrent disease or other drugs or chemicals. Information on drug withdrawal may be lacking or unclear.	
Unrelated	This category is applicable to adverse events which are judged to be clearly and incontrovertibly due to extraneous causes (diseases, environment, etc.) and do not meet the criteria for drug relationship listed for the above-mentioned conditions.	

16.2.6 Serious Adverse Event

A Serious Adverse Event is any untoward medical occurrence (whether deemed to be related to investigational medication or not) that meets any of the following criteria:

- Fatal (i.e., the adverse event actually causes or leads to death)
- Life-threatening (i.e., the adverse event, in the view of the investigator, places the patient at immediate risk of death). This does not include any adverse event that, had it occurred in a more severe form or was allowed to continue, might have caused death.
- Requires or prolongs inpatient hospitalization
- Results in persistent or significant disability/incapacity (i.e., the adverse event results in substantial disruption of the patient's ability to conduct normal life functions)
- Congenital anomaly/birth defect in a neonate/infant born to a mother exposed to study drug
- Significant medical event in the investigator's judgment (e.g., may jeopardize the patient or may require medical/surgical intervention to prevent one of the outcomes listed above)

16.2.7 Adverse Event Recording and Follow Up

The investigator is responsible for evaluating and reporting any adverse events occurring during the study. The investigator will record onset, duration, intensity, any taken action, evolution/outcome, and the causality assessment for any adverse event.

Any adverse events leading to withdrawal from the study and those that persist at the end of the study must be followed up.

16.2.8 Serious Adverse Events Reporting

Although adverse events are recorded in the adverse event form of CRF special reporting requirements apply to the reporting of serious adverse events. Serious adverse events need to be reported immediately. The investigator should immediately notify the principal investigator of any serious adverse events that occurred at the trial site within 24 hours: MARVEL security desk Fax: +86 023-68774270

Serious adverse event reports will be collected centrally at the safety desk. Copies of all serious adverse event reports will be sent to the central trial management. Such preliminary reports will be followed by detailed descriptions, including anonymous copies of hospital case reports or related results, as well as other documents (if required and applicable). All serious adverse events that have not been resolved by the end of the study, or that have not been resolved upon discontinuation of the subject's participation in the study, must be followed until any of the following occurs:

- the event resolves
- the event stabilizes
- the event returns to baseline, if a baseline value is available
- the event can be attributed to agents other than the study drug or to factors unrelated to study conduct

- when it becomes unlikely that any additional information can be obtained

Any additional information known after the event has been initially reported should be sent using a new serious adverse event report form. New information will be noted on the "serious adverse event" form, by ticking the box marked "follow-up" and sending to MARVEL SAFETY DESK.

Patients who withdraw from the study treatment due to any adverse event will be followed up at least until the outcomes are determined, even if it implies that the follow-up continues after the patient has left the trial.

The investigator should assess the seriousness of the adverse events. This is based on the regulatory definitions of seriousness. The investigator should assess the causal relationship of serious adverse events. This is a clinical assessment of whether the adverse events may be related to the investigational medication. The evaluation of the expectedness is based on knowledge of adverse reactions and references.

17. Study Discontinuation Criteria

17.1 Patient Discontinuation

Patients have the right to voluntarily withdraw from the study at any time for any reason. In addition, the investigator has the right to withdraw the patient from the study at any time. Reasons for withdrawal from the study may include, but are not limited to the following:

- Patient withdraws consent at any time

- Any medical condition determined by the investigator may endanger the patient's safety if he or she continues the study
- The investigator determines that it is in the best interests of the patient
- Patient noncompliance, specifically defined as unwillingness to participate in a 3-month assessment of neurological status and function

Every effort should be made to obtain information about patients who withdrew from the study. The main reasons for withdrawal from the study should be recorded on the corresponding CRF. Patients who withdraw from the study will not be replaced.

17.2 Study Treatment Discontinuation

The treatment with the investigate medication should be terminated immediately if the patients experience any of the following:

- Any serious gastrointestinal bleeding.
- Uncontrollable hyperglycaemia (blood sugar >22.2 mmol/L)
- Anaphylactic reaction
- Continuation of the study drug would be detrimental to the patient's well-being.
- Withdrawal for personal reasons.

According to the intention to treat (ITT) principle, patients who discontinue study treatment prematurely will continue in the study and be followed up to the 90th day.

17.3 Study and Site Discontinuation

The investigators has the right to terminate this study in any case of concern about the safety of patients caused by new information. The reasons for terminating the study may include, but are not limited to:

- The incidence or severity of adverse events in this or other studies indicates a potential health hazard to patients.
- Patient enrolment is unsatisfactory

The investigators have the right to close a site at any time. Reasons for closing a site may include, but are not limited to, the following:

- Excessively slow recruitment
- Poor protocol adherence
- Inaccurate or incomplete data recording
- Non-compliance with the International Conference on Harmonization guideline for Good Clinical Practice
- No study activity

18. Statistical Analysis

This study will test whether treatment of patients with acute ischemic stroke with intravenous methylprednisolone will lead to a lower mRS score at 90 days.

18.1 Determination of sample size

The current trial is designed detect a shift on the modified Rankin Scale score, which represents the global disability, to a lower score. The expected distribution of the modified Rankin Scale score, derived from the EVT for acute anterior circulation ischemic stroke (ACTUAL) registry[40] and the Highly Effective Reperfusion evaluated in Multiple Endovascular Stroke Trials (HERMES) collaboration[7], is as follows: 7%, 20%, 16%, 11%, 13%, 8% and 25%.

The sample size was calculated using the cumulative proportion of patients with mRS 0-2. We assume a moderate effect of 7% absolute increase in the cumulative proportion of patients with mRS 0-2 in the intervention group, compared with controls, indicating an odds ratio (OR) of 1.33, which have substantially exceeds the minimal clinically important difference.[41,42] In order to demonstrate the expected treatment effect with a type-1 error alpha =0.05 (two-tailed) and a power of 80% (beta = 20%). A sample size of n= 1588 patients (n=794 per treatment group) is required.

The intention-to-treat principle will be applied to the primary analysis and, therefore, to safeguard against dilution of the treatment effect associated with an approximate 5% non-adherence rate (due to loss to follow-up, consent withdrawal and other reasons), we initially planned to enrol n=1672 patients (n=836 per treatment group) for this study. This estimation was performed based on PASS (NCSS, LLC. Kaysville, Utah, USA) version 15.0.

18.2 Analysis population

18.2.1 Intention-to-Treat Population

The ITT population includes all patients randomized into the trial who were recorded as receiving any amount of study drug, even if the subject does not receive the correct treatment, or does not follow the protocol until completion[48]. The ITT population will be the primary analysis population for the efficacy endpoints and subjects will be analysed according to the treatment group to which they were assigned at randomization.

18.2.2. Per-Protocol Population

The PP population is defined as the subset of the ITT population excluding major protocol violators deemed to have the potential to affect patient outcome in terms of efficacy.

The PP population includes patients who actually received the assigned treatment and do not have major protocol violations or deviations. Major protocol violations or deviations will be identified in a blinded fashion prior to database lock. More specifically, patients with anyone of the following criteria will be excluded from the PP population. These deviations will be determined based on the medical monitors' records, as well as programmatically, using the following criteria at a minimum:

- Received but did not complete treatment with study drug, or dose of study drug administered outside recommended dose.
- Received study drug after 2 hours after arterial access closure.
- Violated inclusion or exclusion criteria.

A list of patients to be excluded from the randomized patients to create the PP-Efficacy analysis will be established and validated by the Steering Committee prior to unblinding.

18.2.3. Safety Population

The Safety Population includes all patients who received any amount of study drug. In case of violation of the randomization scheme, patients will be classified according to the treatment they actually received. Patients will be assigned to the different populations prior to unblinding of the database. Patients who withdraw informed consent immediately after randomization and are not to receive any treatment should be excluded from Safety Population.

18.2 Analysis of Efficacy–primary endpoint

The primary efficacy analysis will estimate the effect of treatment with intravenous methylprednisolone and with the placebo in acute ischemic stroke patients.

The primary efficacy outcome is the global disability defined by mRS score at 90 days post-randomization. Adjusted common odds ratio or assumption free method will be calculated. The adjusted covariates include age, baseline NIHSS score, pre-stroke mRS, baseline ASPECTS score, use of intravenous thrombolysis, time from onset to randomization, and occlusion location. (Details are described in SAP Section 12.1). We will analyse the primary outcome in the intention-to-treat (ITT) and per-

protocol (PP) population.

18.3 Analysis of Efficacy – secondary endpoints

The mRS score will be used to evaluate the proportion of patients with a score = 0 to 4 *versus* 5 or 6, 0 to 3 *versus* 4 or higher, 0 to 2 *versus* 3 or higher, 0 to 1(or return to pre stroke morbidity) *versus* 2 or higher and at day 90±7. The adjusted risk ratio will be calculated by fitting the GLM models separately.

NIHSS score at 5-7 days or at early discharge and Functional health status quality of life 90 (±7) days after randomization (EQ-5D-VAS). The EQ-5D-VAS will be compared between the methylprednisolone group and the placebo group via GLM.

Normality and variance homogeneity of residuals will be accessed graphically. The win ratio method will be used if normality assumption is seriously violated.

The significance of each test is determined at the two-sided alpha level of 0.05. The specific statistical model for analysing each of these outcome measures will be detailed in the Statistical Analysis Plan (SAP).

18.4 Safety analysis

Safety outcomes include incidence of overall mortality at 90 (±7) days after randomization, the proportion of patient with SICH within 48 hours after treatment, the proportion of patient with any ICH within 48 hours after treatment, proportion of patients with pneumonia and proportion of patients with gastrointestinal haemorrhage within 7 days after EVT;. The safety analysis will be performed on the Safety

Population. The incidences of each outcome will be compared via Chi-square test or Fisher's exact test. The modified Poisson regression models will be fitted to estimate the risk-ratio associated with the treatment effect. Risk ratio with 95% CI will be reported. Patients with missing outcome will not be included in the regression analysis. Additionally, Log-Rank test and the Kaplan-Meier estimates will be plotted over the observation period of 90 days for mortality. Cox regression model will be employed to calculate the hazard ratio with 95%CI.

18.5 Tolerability Analysis (adverse events)

Tolerability analyses will be performed on the Safety Population only.

18.5.1 Adverse Events

Adverse Events will be coded using the Medical Dictionary for Regulatory Activities (MedDRA) central coding dictionary.

18.5.2 Serious Adverse Events

Serious Adverse Event reconciliation will be performed by Data Management, Clinical Research, and Pharmacovigilance via data listings.

A summary of patients with serious adverse events will be presented by treatment group for all patients in the Safety Population. A data listing of SAEs will also be provided, displaying details of the event(s) captured on the CRF. The between group difference will be tested using Chi-square test or Fisher's exact test.

18.5.3 All Adverse Events

Summaries of patients with AEs by System Organ Class and Preferred Term (MedDRA) will be prepared. Each patient will be counted only once within each category (System Organ Class or Preferred Term). If a patient experiences more than one AE within a category, only the AE with the strongest relationship or the greatest intensity, as appropriate, will be included in the summaries. The between group difference will be tested using Chi-square test or Fisher's exact test. Only AEs beginning at or after the beginning of study drug administration will be included.

18.6 Adjustment for Covariates and Subgroup Analyses

In addition to the primary and secondary analyses adjusting for age, baseline NIHSS score, pre-stroke mRS, baseline ASPECTS score, the use of intravenous thrombolysis, time from onset to randomization, and occlusion location, exploratory analyses will be conducted to determine the potential roles of common baseline characteristics and assess potential heterogeneity of treatment effect across subgroups. The primary pre-specified subgroups as follows will be explored.

- age
- sex
- baseline NIHSS score
- pre-stroke mRS
- baseline ASPECTS score
- intravenous thrombolysis

- time from last known well to randomization. (mins)
- occlusion location
- Complete Reperfusion defined as Extended Thrombolysis in Cerebral Infarction grade 2c or 3
- Patients with any radiological haemorrhages
- Patients with symptomatic haemorrhages

18.7 Missing data handling

All efforts will be made to minimize the amount of missing data. However, some missing data may be inevitable due to, for example, loss to follow-up. Missing baseline covariates will be imputed using simple imputation or multiple imputation methods (Details in SAP Section 9.2.2). Sensitivity analyses based on different hypotheses about the missingness pattern of the primary outcome will be performed to test for the robustness of the primary analysis (Details in SAP Section 12.1.5).

19. Ethical and regulatory consideration

19.1 General requirements and Considerations

This study followed the ethical principles of the Helsinki Declaration. Approval of the conduct of the trial will be obtained from the Ethics Committees of all participating centres as well as from the local regulatory authorities. The trial will not start in any centre before written approval and authorization by the respective Ethics Committee and Regulatory Authority. Any subsequent protocol amendment will be submitted to

the Ethics Committee for approval. The involvement of committees in the clinical trial of MARVEL will further ensure that the subjects have the highest priority at any time. In addition to the treatment to be tested, all diagnostic procedures and treatments applied are part of standard management of acute stroke patients and will follow the national guidelines. These procedures are therefore of immediate benefit to the patients. The investigators will assure that every patient participating in the trial will receive best medical treatment.

19.2 Study monitoring and quality control

The design of the MARVEL trial has been carefully reviewed and approved by the Steering Committee before being submitted to the Ethics Committees and Regulatory Authorities for approval. In addition, the independent EAB and DSMB and an external SAB have reviewed and approved the trial protocol and will continuously monitor the conduction of the trial. These committees, composed of well-known independent experts, will ensure sufficient alertness to all ethical and safety issues.

The investigators promise to conduct the MARVEL trial in accordance with this trial protocol, International Conference on Harmonization-GCP (ICH-GCP) Guidelines and applicable regulatory requirements. The investigators agree to provide reliable data and all information required by the trial protocol in an accurate and legible manner according to the instructions provided.

The investigators of this clinical trial is responsible to the health authorities and takes all reasonable measures to ensure the correct implementation of the clinical trial

protocol in terms of ethics, clinical trial protocol compliance, and the completeness and validity of the data recorded on the CRF.

The main responsibility of the monitoring team is to help investigators to ensure that all aspects of clinical trials are highly ethical, scientific, professional and standardized. The monitoring team will regularly contact the centres through field visits, emails or phone calls, and send inspectors to assess the progress of the trial, the compliance of investigators and patients with the trial protocol, and to resolve urgent issues. During these inspection visits, the inspector will work with the on-site investigator. The main aspects of inspection and monitoring are as follows (not exclusive): patient informed consent, patient recruitment and follow-up, serious adverse event recording and reporting, study drug supply, treatment compliance of study drug group participants, study drug count, concomitant treatment and data quality.

19.3 Informed Consent

The written informed consent must be obtained from all participants in the clinical trial prior to inclusion into the study. Informed consent forms must be written to be easily understood by the participants or their legal representatives, enabling them to understand the purpose of the trial, procedures, possible benefits, potential risks, and the rights/obligations of participation. Participants have the right to withdraw from the study at any stage of the trial. Each participant must leave contact information to the investigator of the coordinating centre. At the same time, the investigator must leave

his own phone number to the participant so that the participant can find the investigator at any time.

19.4 Confidentiality

Personal data will be processed in accordance with Chinese data protection directives and regulations, relevant international legislation and good practices. Data will only be processed for the trial's purposes. The investigators encode each patient participating in the study by assigning a unique patient identification number to maintain confidentiality standards. This means that all individual patients' data will be linked to the CRF via a unique identification number throughout the trial. Individual patient's medical information will be recorded only in anonymous form. The clinical monitors may inspect source data in order to ensure the accuracy of the data recorded in the CRF.

19.5 Liability and Insurance

The study investigators provide an appropriate insurance for patients in the event of any trial related damage in accordance with applicable national laws. A certificate of insurance will be provided to the investigator of the coordinating centre in which this document is required.

20. Administrative procedures

20.1 Curriculum vitae

A latest copy of the curriculum vitae of each investigator and co-investigator will be provided to the responsible coordinating centre prior to the start of the study.

20.2 Secrecy agreement

The investigators will take all necessary measures to ensure that there is no violation of confidentiality in respect of all information accumulated, acquired or deduced in the course of the trial, other than that information to be disclosed by law.

20.3 Ownership of data and use of the study results

All materials, information and unpublished documentation supplied to the investigators, inclusive of this study, and the patient case report forms are the exclusive property of the study initiators. Therefore, the study initiators reserve the right to use the data of the present study, either in the form of case report forms, or in the form of a report, with or without comments and with or without analysis, in order to submit them to the health authorities.

20.4 Protocol amendments

Any protocol amendments will be prepared by the investigators. Protocol amendments should be submitted to the REB/IRB for approval prior to implementation in accordance with local regulatory requirements. Approval must be obtained from the REB/IRB and regulatory authorities (as locally required) before implementation of any changes, except for changes necessary to eliminate an immediate hazard to patients or changes that involve logistical or administrative aspects only

21. Data Retention

The double reviewed case report form (CRF) and imaging data will be sent to the data management group. The person in charge of the data management group will check and sign the receipt form. The CRF will be kept by the research centre after data entry is completed.

22. Study report and publications

The results of the trial will be reported to the regulatory authorities and ethics committees. The investigators will provide an annual safety report and the final report.

According to the pre-defined analysis in the clinical trial protocol, the results of the trial will be published in the appropriate journal (for manuscripts) or meeting (for abstracts).

By signing the clinical trial protocol，the investigator agrees that the results of the clinical trial can be used for publication.

The trial will be registered at the Chinese Clinical Trial Registry website.

23.?References?Uncategorized References

1. Global, regional, and national burden of stroke and its risk factors, 1990-2019: a systematic analysis for the Global Burden of Disease Study 2019. Lancet Neurol 2021;20:795-820.

2. Diseases GBD, Injuries C. Global burden of 369 diseases and injuries in 204 countries and territories, 1990-2019: a systematic analysis for the Global Burden of Disease Study 2019. Lancet 2020;396:1204-22.

3. Lindsay MP, Norrving B, Sacco RL, et al. World Stroke Organization (WSO): Global Stroke Fact Sheet 2019. Int J Stroke 2019;14:806-17.

4. Wang W, Jiang B, Sun H, et al. Prevalence, Incidence, and Mortality of Stroke in China: Results from a Nationwide Population-Based Survey of 480 687 Adults. Circulation 2017;135:759-71.

5. Baron JC. Protecting the ischaemic penumbra as an adjunct to thrombectomy for acute stroke. Nat Rev Neurol 2018;14:325-37.

6. Powers WJ, Rabinstein AA, Ackerson T, et al. Guidelines for the Early Management of Patients With Acute Ischemic Stroke: 2019 Update to the 2018 Guidelines for the Early Management of Acute Ischemic Stroke: A Guideline for Healthcare Professionals From the American Heart Association/American Stroke Association. Stroke 2019;50:e344-e418.

7. Goyal M, Menon BK, van Zwam WH, et al. Endovascular thrombectomy after large-vessel ischaemic stroke: a meta-analysis of individual patient data from five randomised trials. LANCET 2016;387:1723-31.

8. Nie X, Pu Y, Zhang Z, Liu X, Duan W, Liu L. Futile Recanalization after Endovascular Therapy in Acute Ischemic Stroke. Biomed Res Int 2018;2018:5879548.

9. Stoll G, Pham M. Beyond recanalization — a call for action in acute stroke. Nature Reviews Neurology 2020;16:591-2.

10. Whiteley WN, Emberson J, Lees KR, et al. Risk of intracerebral haemorrhage with alteplase after acute ischaemic stroke: a secondary analysis of an individual patient data meta-analysis. Lancet Neurol 2016;15:925-33.

11. Shi ZS, Duckwiler GR, Jahan R, et al. Early Blood-Brain Barrier Disruption after Mechanical Thrombectomy in Acute Ischemic Stroke. J Neuroimaging 2018;28:283-8.

12. Conti E, Piccardi B, Sodero A, et al. Translational Stroke Research Review: Using the Mouse to Model Human Futile Recanalization and Reperfusion Injury in Ischemic Brain Tissue. Cells 2021;10:19.

13. Cheripelli BK, Huang XY, MacIsaac R, Muir KW. Interaction of Recanalization, Intracerebral Hemorrhage, and Cerebral Edema After Intravenous Thrombolysis. Stroke 2016;47:1761-7.

14. Chen XL, Huang Q, Deng QW, et al. A prediction model of brain edema after endovascular treatment in patients with acute ischemic stroke. Journal of the Neurological Sciences 2019;407.

15. Ames A, 3rd, Wright RL, Kowada M, Thurston JM, Majno G. Cerebral ischemia. II. The no-reflow phenomenon. The American journal of pathology 1968;52:437-53.

16. Caiazzo G, Musci RL, Frediani L, et al. State of the Art: No-Reflow Phenomenon. Cardiol Clin 2020;38:563-73.

17. Kloner RA, King KS, Harrington MG. No-reflow phenomenon in the heart and brain. Am J Physiol Heart Circ Physiol 2018;315:H550-H62.

18. Ng FC, Churilov L, Yassi N, et al. Microvascular Dysfunction in Blood-Brain Barrier Disruption and Hypoperfusion Within the Infarct Posttreatment Are Associated With Cerebral Edema. 2022;53:1597-605.

19. Planas AM. Role of Immune Cells Migrating to the Ischemic Brain. Stroke 2018;49:2261-7.

20. Shi KB, Tian DC, Li ZLG, Ducruet AF, Lawton MT, Shi FD. Global brain inflammation in stroke. Lancet Neurology 2019;18:1058-66.

21. Laha RK, Dujovny M, Barrionuevo PJ, DeCastro SC, Hellstrom HR, Maroon JC. Protective effects of methyl prednisolone and dimethyl sulfoxide in experimental middle cerebral artery embolectomy. J Neurosurg

1978;49:508-16.
22. Slivka AP, Murphy EJ. High-dose methylprednisolone treatment in experimental focal cerebral ischemia. Exp Neurol 2001;167:166-72.
23. de Courten-Myers GM, Kleinholz M, Wagner KR, Xi G, Myers RE. Efficacious experimental stroke treatment with high-dose methylprednisolone. Stroke 1994;25:487-92; discussion 93.
24. Barbosa-Coutinho LM, Hartmann A, Hossmann KA, Rommel T. Effect of dexamethasone on serum protein extravasation in experimental brain infarcts of monkey: an immunohistochemical study. Acta Neuropathol 1985;65:255-60.
25. Espinosa A, Meneses G, Chavarría A, et al. Intranasal Dexamethasone Reduces Mortality and Brain Damage in a Mouse Experimental Ischemic Stroke Model. Neurotherapeutics 2020;17:1907-18.
26. Altamentova S, Rumajogee P, Hong J, et al. Methylprednisolone Reduces Persistent Post-ischemic Inflammation in a Rat Hypoxia-Ischemia Model of Perinatal Stroke. Transl Stroke Res 2020;11:1117-36.
27. Limbourg FP, Huang Z, Plumier JC, et al. Rapid nontranscriptional activation of endothelial nitric oxide synthase mediates increased cerebral blood flow and stroke protection by corticosteroids. J Clin Invest 2002;110:1729-38.
28. Norris JW. Steroids may have a role in stroke therapy. Stroke 2004;35:228-9.
29. Nellis SH, Roberts BH, Kinney EL, Field J, Ummat A, Zelis R. Beneficial effect of dexamethasone on the "no reflow" phenomenon in canine myocardium. Cardiovasc Res 1980;14:137-41.
30. Sandercock PAG, Soane T. Corticosteroids for acute ischaemic stroke. Cochrane Database of Systematic Reviews 2011.
31. Ogun SA, Odusote KA. Effectiveness of high dose dexamethasone in the treatment of acute stroke. West African journal of medicine 2001;20:1-6.
32. Nie X, Leng X, Miao Z, Fisher M, Liu L. Clinically Ineffective Reperfusion After Endovascular Therapy in Acute Ischemic Stroke. 2023;54:873-81.
33. Lyden P, Buchan A, Boltze J, et al. Top Priorities for Cerebroprotective Studies—A Paradigm Shift: Report From STAIR XI. Stroke 2021;52:3063-71.
34. Lapchak PA. Emerging Therapies: Pleiotropic Multi-target Drugs to Treat Stroke Victims. Translational Stroke Research 2011;2:129-35.
35. Fraser JF, Pahwa S, Maniskas M, et al. Now that the door is open: an update on ischemic stroke pharmacotherapeutics for the neurointerventionalist. Journal of Neurointerventional Surgery 2023.
36. Savitz SI, Baron JC, Fisher M, Consortium SX. Stroke Treatment Academic Industry Roundtable X: Brain Cytoprotection Therapies in the Reperfusion Era. Stroke 2019;50:1026-31.
37. Savitz SI, Baron J-C, Yenari MA, Sanossian N, Fisher M. Reconsidering Neuroprotection in the Reperfusion Era. 2017;48:3413-9.
38. Davis SM, Donnan GA. Steroids for stroke: another potential therapy discarded prematurely? Stroke 2004;35:230-1.
39. Huting ZHANG SL. Influence of neurological intervention combined with dexamethasone injection on the outcome for the patients suffered from acute ischemic stroke. Journal of Molecular Imaging 2016;39:121-4.
40. Zi W, Wang H, Yang D, et al. Clinical Effectiveness and Safety Outcomes of Endovascular Treatment for Acute Anterior Circulation Ischemic Stroke in China. Cerebrovasc Dis 2017;44:248-58.
41. Cranston JS, Kaplan BD, Saver JL. Minimal Clinically Important Difference for Safe and Simple Novel Acute Ischemic Stroke Therapies. Stroke 2017;48:2946-51.
42. Berkhemer OA, Fransen PSS, Beumer D, et al. A Randomized Trial of Intraarterial Treatment for Acute Ischemic Stroke. New England Journal of Medicine 2015;372:11-20.

43. Wang Y, Han S, Qin H, et al. Chinese Stroke Association guidelines for clinical management of cerebrovascular disorders: executive summary and 2019 update of the management of high-risk population. Stroke Vasc Neurol 2020;5:270-8.

44. Banks JL, Marotta CA. Outcomes validity and reliability of the modified Rankin scale: implications for stroke clinical trials: a literature review and synthesis. Stroke 2007;38:1091-6.

45. Quinn TJ, Dawson J, Walters MR, Lees KR. Reliability of the modified Rankin Scale: a systematic review. Stroke 2009;40:3393-5.

46. Brott T, Adams HP, Jr., Olinger CP, et al. Measurements of acute cerebral infarction: a clinical examination scale. Stroke 1989;20:864-70.

47. von Kummer R, Broderick JP, Campbell BC, et al. The Heidelberg Bleeding Classification: Classification of Bleeding Events After Ischemic Stroke and Reperfusion Therapy. Stroke 2015;46:2981-6.

48. White IR, Horton NJ, Carpenter J, Pocock SJ. Strategy for intention to treat analysis in randomised trials with missing outcome data. Bmj 2011;342

24. Appendices

24.1 Appendix 1: mRS

Subject ID ___ ___ ___ ___ ___ ___ ___ ___ ___ ___
Subject Date of Birth ___ ___/___ ___/___ ___
Hospital ID ________________________________
Date of Examination ___ ___/___ ___/___ ___

The Modified Rankin Scale (mRS)

(Use web calculator at www.modifiedrankin.com)

0 No symptoms

1 No significant disability; able to carry out all usual activities, despite some symptoms

2 Slight disability; able to look after own affairs without assistance, but unable to carry out all previous activities

3 Moderate disability; requires some help, but able to walk unassisted

4 Moderately severe disability; unable to attend to own bodily needs without assistance, and unable to walk unassisted

5 Severe disability; requires constant nursing care and attention, bedridden, incontinent

6 Dead

References:

Rankin J (May 1957). "Cerebral vascular accidents in patients over the age of 60. II. Prognosis". Scott Med J 2 (5): 200–15

Patel, N., et al. Simple and reliable determination of the modified Rankin Scale in neurosurgical and neurological patients: The mRS-9Q. *Neurosurgery*, published online in advance of print 26 July 2012

24.2 Appendix 2: NIHSS

NIHSS page 1

Subject ID ___ ___ ___ ___ ___ ___ ___ ___ ___ ___
Subject Date of Birth ___ ___/___ ___/___ ___
Hospital ID ______________________________
Date of Examination ___ ___/___ ___/___ ___

NATIONAL INSTITUTES OF HEALTH STROKE SCALE (NIHSS)[1]

Interval: [ ] Baseline
[ ] 7-10 days
[ ] 1 month
[ ] 3 months
[ ] 6 months
[ ] Other ___________________________________

Time: ___ ___:___ ___ [ ]am [ ]pm

Person Administering Scale ___________________________________

Purpose:
The NIH Stroke Scale (NIHSS) is a standardized neurological examination intended to describe the neurological deficits found in large groups of stroke patients participating in treatment trials.

Administer stroke scale items in the order listed. Record performance in each category after each subscale exam. Do ***not*** go back and change scores. Follow directions provided for each exam technique. Scores should reflect what the patient does, not what the clinician thinks the patient can do. The clinician should record answers while administering the exam and work quickly. Except where indicated, the patient should not be coached (i.e., repeated requests to patient to make a special effort).

Instructions	Scale Definition	Score
**1a. Level of Consciousness:** The investigator must choose a response if a full evaluation is prevented by such obstacles as an endotracheal tube language barrier, orotracheal trauma/bandages. A 3 is scored only if the patient makes no movement (other than reflexive posturing) in response to noxious stimulation.	0 = **Alert**; Keenly responsive.<br>1 = **Not alert, but arousable** by minor stimulation to obey, answer or respond.<br>2 = **Not alert**; requires repeated stimulation to attend, or is obtunded and requires strong or painful. stimulation to make movements (not stereotyped).<br>3 = **Responds only with reflex motor or autonomic effects or totally unresponsive**, flaccid, and areflexic.	________
**1b. LOC Questions**: The patient is asked the month and his/her age. The answer must be correct – there is no partial credit for being close. Aphasic and stuporous patients who do not comprehend the questions will score 2. Patients unable to speak because of endotracheal intubation, orotracheal trauma, severe dysarthria from any cause, language barrier, or any other problem not secondary to aphasia are given a 1. It is important that only the initial answer be graded and that the examiner not "help" the patient with verbal or non-verbal cues.	0 = **Answers both questions correctly**.<br>1 = **Answers one question correctly**.<br>2 = **Answers neither question correctly**.	________

[1] The National Institute of Neurological Diseases and Stroke (NINDS), National Institutes of Health (NIH), Last Revised 01 October 2003 (https://stroke.nih.gov/resources/index.htm).

NIHSS page 2

Instructions	Scale Definition	Score
**6. Motor Leg:** The limb is placed in the appropriate position: hold the leg at 30 degrees (always tested supine). Drift is scored if the leg falls before 5 seconds. The aphasic patient is encouraged using urgency in the voice and pantomime, but not noxious stimulation. Each limb is tested in turn, beginning with the non-paretic leg. Only the case of amputation or joint fusion at the hip, should the examiner record the score as untestable (UN), and clearly write the explanation for this choice.	0 = **No drift**; limb holds 90 (or 45) degrees for full 10 seconds.<br>1 = **Drift**; limb holds 90 (or 45) degrees, but drifts down before full 10 seconds; does not hit bed or other support.<br>2 = **Some effort against gravity**; limb cannot get to or maintain (if cued) 90 (or 45) degrees, drifts down to bed, but has some effort against gravity.<br>3 = **No effort against gravity**; leg falls to bed immediately.<br>4 = **No movement**.<br>UN= **Amputation** or joint fusion; explain: ______<br>6a= **Left Arm**.<br>6b= **Right Arm**.	______<br>______
**7. Limb Ataxia:** This item is aimed at finding evidence of a unilateral cerebellar lesion. Test with eyes open. In case of visual defect, ensure testing is done in intact visual field. The finger-nose-finger and heel-shin tests are performed on both sides, and ataxia is scored only if present out of proportion to weakness. Ataxia is absent in the patient who cannot understand or is paralyzed. Only in the case of amputation or joint fusion, should the examiner record the score as untestable (UN), and clearly write the explanation for this choice. In case of blindness, test by having the patient touch nose from extended arm position.	0 = **Absent.**<br>1 = **Present in one limb**.<br>2 = **Present in two limbs**.<br>UN= **Amputation** or joint fusion; explain: ______	______
**8. Sensory:** Sensation or grimace to pinprick when tested, or withdrawal from noxious stimulus in the obtunded or aphasic patient. Only sensory loss attributed to stroke is scored as abnormal and the examiner should test as many body areas [arms (not hands), legs, trunk, face) as needed to accurately check for hemisensory loss. A score of 2, "severe or total sensory loss," should only be given when a severe or total loss of sensation can be clearly demonstrated. Stuporous and aphasic patients will, therefore, probably score 1 or 0. The patient with brainstem stroke who has bilateral loss of sensation is scored 2. If the patient does not respond and is quadriplegic, score 2. Patients in a coma (item 1a=3) are automatically given a 2 on this item.	0 = **Normal**; no sensory loss.<br>1 = **Mild-to-moderate** sensory loss; patient feels pinprick is less sharp or is dull on the affected side, or there is a loss of superficial pain with pinprick, but patient is aware of being touched.<br>2 = **Severe to total** sensory loss; patient is not aware of being touched in the face, arm, and leg.	______
**9. Best Language:** A great deal of information about comprehension will be obtained during the preceding sections of the examination. For this scale item, the patient is asked to describe what is happening in the attached picture, to name the items on the attached naming sheet and to read from the attached list of sentences. Comprehension is judged from responses here, as well as to all of the commands in the preceding general neurological exam. If visual loss interferes with the tests, ask the patient to identify objects placed in the hand, repeat, and produce speech. The intubated patient should be asked to write. The patient in a coma (item 1a=3) will automatically score 3 on this item. The examiner must choose a score for the patient with stupor or limited cooperation, but a score of 3 should be used only if the patient is mute and follows no one-step commands.	0 = **No asphasia**; normal.<br>1 = **Mild-to-moderate aphasia**; some obvious loss of fluency or facility of comprehension, without significant limitation on ideas expressed or form of expression. Reduction of speech and/or comprehension, however, makes conversation about provided materials difficult or impossible. For example, in conversation about provided materials, examiner can identify picture or naming card content from patient's response.<br>2 = **Severe asphasia**; all communication is through fragmentary expression; great need for inference, questioning, and guessing by the listener. Range of information that can be exchanged is limited; listener carries burden of communication. Examiner cannot identify materials provided from patient response.<br>3 = **Mute, global aphasia**; no usable speech or auditory comprehension.	______

NIHSS page 3

Instructions	Scale Definition	Score
**1c. LOC Commands:** The patient is asked to open and close the eyes and then to grip and release the non-paretic hand. Substitute another one step command if the hands cannot be used. Credit is given if an unequivocal attempt is made but not completed due to weakness. If the patient does not respond to command, the task should be demonstrated to him/her (pantomime), and the result scored (i.e., follows none, one or two commands). Patients with trauma, amputation, or other physical impediments should be given suitable one-step commands. Only the first attempt is scored.	0 = **Answers both tasks correctly**.<br>1 = **Answers one task correctly**.<br>2 = **Answers neither task correctly**.	________
**2. Best Gaze**: Only horizontal eye movements will be tested. Voluntary or reflexive (oculocephalic) eye movements will be scored, but caloric testing is not done. If the patient has a conjugate deviation of the eyes that can be overcome by voluntary or reflexive activity, the score will be 1. If a patient has an isolated peripheral nerve paresis (CN III, IV or VI), score a 1. Gaze is testable in all aphasic patients. Patients with ocular trauma, bandages, pre-existing blindness, or other disorder of visual acuity or fields should be tested with reflexive movements, and a choice made by the investigator. Establishing eye contact and then moving about the patient from side to side will occasionally clarify the presence of a partial gaze palsy.	0 = **Normal.**<br>1 = **Partial gaze palsy**; gaze is abnormal in one or both eyes, but forced deviation or total gaze paresis is not present.<br>2 = **Forced deviation**, or total gaze paresis not overcome by the oculocephalic maneuver.	________
**3. Visual:** Visual fields (upper and lower quadrants) are tested by confrontation, using finger counting or visual threat, as appropriate. Patients may be encouraged, but if they look at the side of the moving fingers appropriately, this can be scored as normal. If there is unilateral blindness or enucleation, visual fields in the remaining eye are scored. Score 1 only if a clear-cut asymmetry, including quadrantanopia, is found. If patient is blind from any cause, score 3. Double simultaneous stimulation is performed at this point. If there is extinction, patient receives a 1, and the results are used to respond to item 11.	0 = **No visual loss**.<br>1 = **Partial hemianopia**.<br>2 = **Complete hemianopia**.<br>3 = **Bilateral hemianopia** (blind including cortical blindness).	________
**4. Facial Palsy:** Ask – or use pantomime to encourage – the patient to show teeth or raise eyebrows and close eyes. Score symmetry of grimace in response to noxious stimuli in the poorly responsive or non-comprehending patient. If facial trauma/bandages, orotracheal tube, tape or other physical barriers obscure the face, these should be removed to the extent possible.	0 = **Normal** symmetrical movements.<br>1 = **Minor paralysis** (flattened nasolabial fold, asymmetry on smiling).<br>2 = **Partial paralysis** (total or near-total paralysis of lower face).<br>3 = **Complete paralysis** of one or both sides (absence of facial movement in the upper and lower face	________
**5. Motor Arm:** The limb is placed in the appropriate position: extend the arms (palms down) 90 degrees (if sitting) or 45 degrees (if supine). Drift is scored if the arm falls before 10 sounds. The aphasic patient is encouraged using urgency in the voice and pantomime, but not noxious stimulation. Each limb is tested in turn, beginning with the non-paretic arm. Only in the case of amputation or joint fusion at the shoulder, should the examiner record the score as untestable (UN), and clearly write the explanation for this choice.	0 = **No drift**; limb holds 90 (or 45) degrees for full 10 seconds.<br>1 = **Drift**; limb holds 90 (or 45) degrees, but drifts down before full 10 seconds; does not hit bed or other support.<br>2 = **Some effort against gravity**; limb cannot get to or maintain (if cued) 90 (or 45) degrees, drifts down to bed, but has some effort against gravity.<br>3 = **No effort against gravity**; limb falls.<br>4 = **No movement**.<br>UN= **Amputation** or joint fusion; explain: ______________________<br>5a= **Left Arm**.<br>5b= **Right Arm**.	________<br>________

NIHSS page 4

Instructions	Scale Definition	Score
**10. Dysarthria:** If patient is thought to be normal, an adequate sample of speech must be obtained by asking patient to read or repeat words from the attached list. If the patient has severe aphasia, the clarity of articulation of spontaneous speech can be rated. Only if the patient is intubated or has other physical barriers to producing speech, should the examiner record the score as untestable (UN), and clearly write an explanation for this choice. Do not tell the patient why he or she is being tested.	0 = **Normal**.<br>1 = **Mild-to-moderate dysarthria**; patient slurs at least some words and, at worst, can be understood with some difficulty.<br>2 = **Severe dysarthria**; patient's speech is so slurred as to be unintelligible in the absence of or out of proportion to any dysphasia, or is mute/anarthric.<br>UN= **Intubated** or other physical barrier; explain: ________________	________
**11. Extinction and Inattention (formerly Neglect):** Sufficient information to identify neglect may be obtained during the prior testing. If the patient has a severe visual loss preventing visual double simultaneous stimulation, and the cutaneous stimuli are normal, the score is normal. If the patient has aphasia but does appear to attend to both sides, the score is normal. The presence of visual spatial neglect or anosagnosia may also be taken as evidence of abnormality. Since the abnormality is scored only if present, the item is never untestable.	0 = **No abnormality**.<br>1 = **Visual, tactile, auditory, spatial, or personal inattention** or extinction to bilateral simultaneous stimulation in one of the sensory modalities.<br>2 = **Profound hemi-inattention or extinction to more than one modality**; does not recognize own hand or orients to only one side of space.	________

NIHSS page 5

Example picture-description stimulus.
Adapted from "Banana Theft v2" by Sam Mshiu, via Wikimedia Commons, licensed under CC BY 4.0. This image is used as an illustrative replacement for the original NIHSS Cookie Theft stimulus and is not the official NIHSS stimulus.

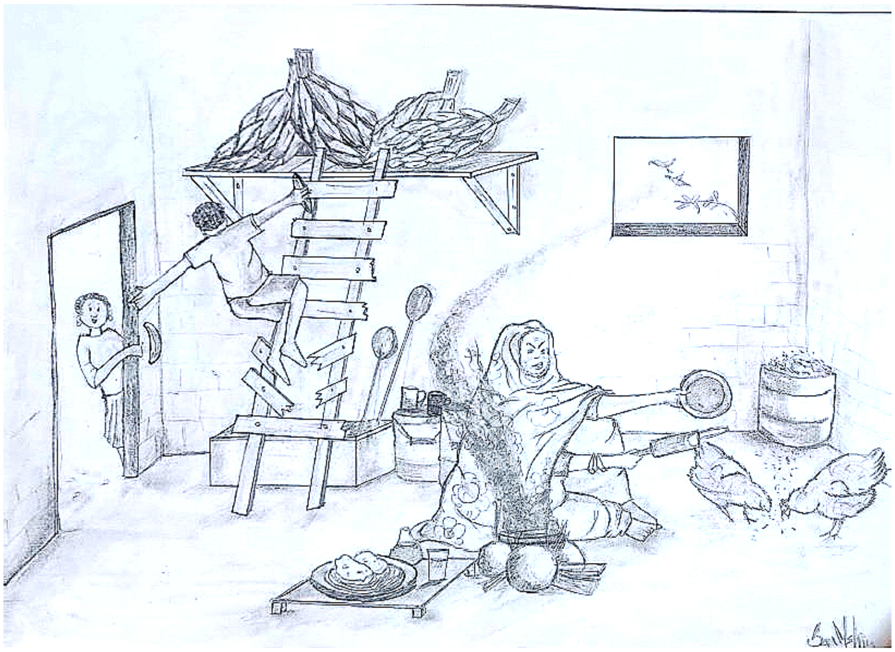

You know how.

Down to earth.

I got home from work.

Near the table in the dining room.

They heard him speak on the radio last night.

请您读出下列句子：
知道
下楼梯
回家做饭
在学校复习
发表精彩演讲

NIHSS page 6

Object-naming stimulus assembled from public-domain Openclipart images: Glove, Key, Cactus, Feather, Chair, and Hammock. This is not the official NIHSS stimulus card. The individual source images are public-domain/CC0 Openclipart works, and the assembled/adapted figure is published under CC BY 4.0.

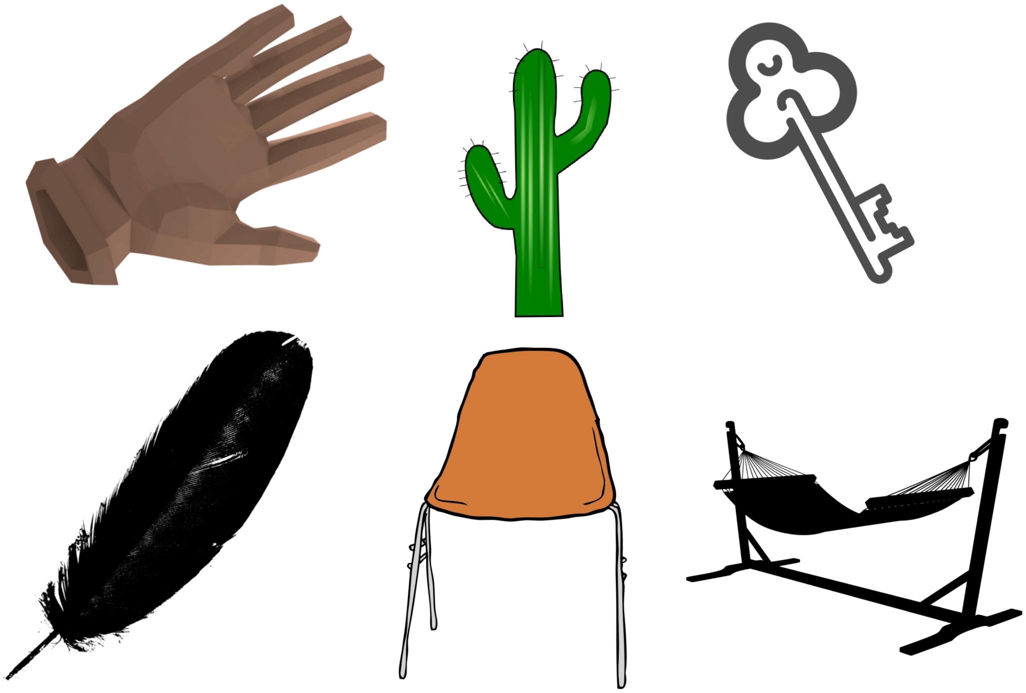

MAMA

TIP – TOP

FIFTY – FIFTY

THANKS

HUCKLEBERRY

BASEBALL PLAYER

**请您读出下列单词：**
**妈妈**
**大地**
**飞机飞机**
**丝绸**
**按时开工**
**吃葡萄不吐葡萄皮**

24.3 Appendix 3: EQ-5D

Subject ID ___ ___ ___ ___ ___ ___ ___ ___ ___ ___
Subject Date of Birth ___ ___/___ ___/___ ___
Hospital ID ________________________________
Date of Examination ___ ___/___ ___/___ ___

Health Questionnaire (EQ-5D-5L)

Under each heading, please tick the ONE box that best describes your health TODAY.

MOBILITY

☐1 I have no problems in walking about
☐2 I have slight problems in walking about
☐3 I have moderate problems in walking about
☐4 I have severe problems in walking about
☐5 I am unable to walk about

SELF-CARE

☐1 I have no problems washing or dressing myself
☐2 I have slight problems washing or dressing myself
☐3 I have moderate problems washing or dressing myself
☐4 I have severe problems washing or dressing myself
☐5 I am unable to wash or dress myself

USUAL ACTIVITIES *(e.g. work, study, housework, family or leisure activities)*

☐1 I have no problems doing my usual activities
☐2 I have slight problems doing my usual activities
☐3 I have moderate problems doing my usual activities
☐4 I have severe problems doing my usual activities
☐5 I am unable to do my usual activities

PAIN / DISCOMFORT

☐1 I have no pain or discomfort
☐2 I have slight pain or discomfort
☐3 I have moderate pain or discomfort
☐4 I have severe pain or discomfort
☐5 I have extreme pain or discomfort

ANXIETY / DEPRESSION

☐1 I am not anxious or depressed
☐2 I am slightly anxious or depressed
☐3 I am moderately anxious or depressed
☐4 I am severely anxious or depressed
☐5 I am extremely anxious or depressed

To help people say how good or bad a health state is, we have drawn a scale (rather like a thermometer) on which the best state you can imagine is marked 100 and the worst state you can imagine is marked 0.

We would like you to indicate on this scale how good or bad your own health is today, in your opinion. Please do this by drawing a line from the box below to whichever point on the scale indicates how good or bad your health state is today.

Your own health state today

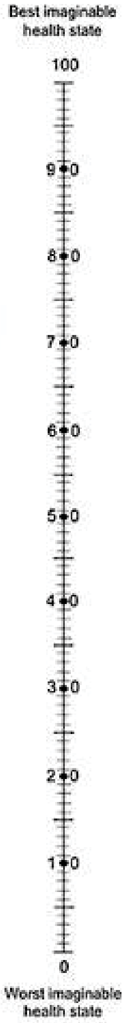

3

UK (English) © 1990 EuroQol Group EQ-5D™ is a trade mark of the EuroQol Group

Summary of Changes - MARVEL Protocol Version 1.0 to Version 2.0

Below is the table of changes. Deleted items are identified with Strikethrough font. Additional wording is in bold font

**Section(s)**	**Protocol Version 1.0**<br>**Change From:**	**Protocol Version 2.0**<br>**Change To:**	**Rationale**
**Section 1. Study Synopsis Inclusion Criteria**	• Baseline Alberta Stroke Program Early CT Score (ASPECTS)>= 5;	Changed:<br>• Baseline Alberta Stroke Program Early CT Score (ASPECTS)>= ~~5~~**3**;	Addition,<br>In light of recent advancements in the management of patients with large infarcts (ASPECTS 3-5), the RESCUE-JAPAN limit trial and several observational studies have demonstrated the superior effectiveness of EVT in treating patients with large infarcts. Additionally, corticosteroids have been suggested to potentially benefit patients with large infarcts.

			To enhance the generalizability and applicability of this study, we have expanded the study population in alignment with the objectives of this research.
**Section 9.1. Study Synopsis Inclusion Criteria**	● Baseline Alberta Stroke Program Early CT Score (ASPECTS)>= 5;	Changed:<br>● Baseline Alberta Stroke Program Early CT Score (ASPECTS)>= ~~5~~***3***;	Addition,<br>In light of recent advancements in the management of patients with large infarcts (ASPECTS 3-5), the RESCUE-JAPAN limit trial and several observational studies have demonstrated the superior effectiveness of EVT in treating patients with large infarcts. Additionally, corticosteroids have been suggested to potentially benefit patients with large infarcts.

			To enhance the generalizability and applicability of this study, we have expanded the study population in alignment with the objectives of this research.
