# Supplementary material for: Post-stroke acute heart failure in patients with large vessel occlusion undergoing endovascular treatment: A pooled analysis of individual patient data from multicenter studies with mediation analysis
Source: PLoS Med. 2026 Jul 7;23(7):e1004752. doi: 10.1371/journal.pmed.1004752 (PMC13340808; doi:10.1371/journal.pmed.1004752)
Supplement: S1 File — This compressed file contains the study protocols for the four multicenter studies included in this pooled analysis: BASILAR registry, DEVT trial, RESCUE-BT trial, and MARVEL trial. Each protocol document is provided in PDF format. (ZIP) [file pmed.1004752.s005.zip › RESCUE-BT1_no_logo.pdf]

RESCUE BT: A multicenter, randomized, placebo-controlled, double-blind trial of endovascular treatment with versus without tirofiban for stroke patients with large vessel occlusion

Supplement – Trial Protocol

This supplement contains the following items:

1. Original Trial Protocol (page 2 to 47)
2. Final Trial Protocol (page 48 to 94)
3. summary of amendments (page 95 to 98)

Note: personal identifying information has been redacted from the protocol and SAP documents to comply with international privacy legislation.

RESCUE BT: A multicenter, randomized, placebo-controlled, double-blind trial of endovascular treatment with versus without tirofiban for stroke patients with large vessel occlusion

TRIAL PROTOCOL

Principle Investigators

Qingwu Yang, MD and Wenjie Zi, MD

Xinqiao Hospital, Army Medical University, Chongqing, China

Prepared by: Zhongming Qiu, MD, Fengli Li, MD, Xinqiao Hospital, Army Medical University, Chongqing, China

Raul G. Nogueira, MD, Department of Neurology, Marcus Stroke & Neuroscience Center, Grady Memorial Hospital, Emory University School of Medicine, Atlanta, GA 30303, USA

Protocol Version: 1.0

Issue Date: 8th July 2018

CONTENTS

List of Abbreviations **6**

Study Synopsis **7**

Schedule of Assessments **12**

1. BACKGROUND INFORMATION **13**

2. TRIAL OBJECTIVES **14**

3. TRIAL DESIGN **15**

4. PATIENT POPULATION **16**

4.1. Inclusion criteria 17

4.2. Exclusion criteria 17

5. BASELINE CLINICAL AND LABORATORY EVALUATIONS **18**

6. PARTICIPATING CENTER ELIGIBILITY **18**

7. RANDOMIZATION **19**

8. BLINDING/UNBLINDING **19**

9. CONTENTS OF INVESTIGATIONAL PRODUCT KIT **20**

9.1. Tirofiban group 20

9.2. Placebo group 20

10. TREATMENTS **21**

11. OUTCOMES **22**

11.1. Primary Efficacy Outcome 22

11.2. Secondary Efficacy Outcomes 22

11.2.1. Secondary Clinical Efficacy Outcomes 22

11.2.2. Secondary Technical Efficacy Outcomes 23

11.3. Safety Outcomes 23

12. ASSESSMENT OF EFFICACY **24**

12.1. The Modified Rankin Scale 24

12.2. The National Institutes of Health Stroke Scale 25

12.3. European Quality Five Dimensions Five Level scale 25

13. ASSESSMENT OF SAFETY **25**

13.1. Symptomatic Intracranial Hemorrhage 26

13.2. Mortality at 90 days ... 26
13.3. Adverse Event Definitions ... 26
13.3.1. Adverse Event ... 26
13.3.2. Serious Adverse Event ... 26
13.4. Clinical Management of Adverse Events ... 28
13.4.1. Early Study Drug Cessation ... 28
13.4.2. Identification of Adverse Events by the Investigator ... 28
13.4.3. Reporting of Adverse Events ... 29
13.4.4. Prompt Reporting of Serious Adverse Events ... 29
14. DATA SAFETY MONITORING BOARD ... 30
15. IMAGING CORE LABORATORY ... 30
16. CLINICAL EVENTS COMMITTEE ... 30
17. STATISTICS ... 31
17.1. Sample size estimates ... 31
17.2. Analysis Populations ... 31
17.2.1. Intention-to-Treat Population ... 31
17.2.2. Per-Protocol Population ... 31
17.2.3. As-Treated Population ... 32
17.2.4. Safety Population ... 32
17.3. Analysis of Primary Efficacy Outcome ... 32
17.4. Analysis of Key Secondary Outcome ... 32
17.5. Analysis of Secondary Outcomes and Safety Outcomes ... 33
17.6. Adjustment for Covariates and Subgroup Analyses ... 33
17.7. Handling of Missing Data ... 34
18. DIRECT ACCESS TO SOURCE DATA/DOCUMENTS ... 34
19. STUDY MONITORING AND QUALITY ASSURANCE ... 35
20. PROTOCOL AMENDMENTS ... 36
21. ETHICAL CONSIDERATION ... 36
22. DATA HANDLING AND RECORD KEEPING ... 37
22.1. Data Handling ... 37

22.2. Data Retention 37
22.3. Case Report Forms 37
22.4. Confidentiality 37
23. USE OF INFORMATION AND PUBLICATION **38**
24. FUNDINGS **38**
Appendix 1 – Classification of Subtype of Acute Ischemic Stroke **40**
Appendix 2 - The Alberta Stroke Program Early Computed Tomography Score **45**
Appendix 3 - Modified Rankin Scale **46**
Investigator's Agreement **47**
REFERENCES **48**

List of Abbreviations

AE	Adverse Event
AIS	Acute Ischemia Stroke
ASPECTS	Alberta Stroke Program Early Computed Tomography Score
CRF	Case Report Form
CT	Computed Tomography
DSMB	Data Safety Monitoring Board
EQ-5D-5L	European Quality Five-Dimension Five-Level
EVT	Endovascular Treatment
GCP	Good Clinical Practice
ICH-GCP	International Conference on Harmonization-Good Clinical Practice
IRB	Institutional Review Board
ITT	Intention-to-Treat
LVO	Large Vessel Occlusion
MR	Magnetic Resonance
mRS	modified Rankin Scale
mTICI	modified Treatment in Cerebral Infarction
NIHSS	National Institutes of Health Stroke Scale
PI	Principal Investigator
REB	Research Ethics Board
RESCUE BT	The Endovascular Treatment With versus Without Tirofiban for Stroke Patients with Large Vessel Occlusion Trial
SAE	Serious Adverse Event
SICH	Symptomatic Intracerebral Hemorrhage
TOAST	Trial of Org 10172 in Acute Stroke Treatment

Study Synopsis

**Study Title**	RESCUE BT: A multicenter, randomized, placebo-controlled, double-blind trial of endovascular treatment with versus without tirofiban for stroke patients with large vessel occlusion
**Study Phase**	Phase 3
**Indication**	Acute Ischemic Stroke
**Study drug**	Active drug: intravenous tirofiban (10μg/kg bolus and then 0.15μg/kg/min maintenance for up to 24 hours)<br>Placebo: saline placebo which is indistinguishable from the active drug
**No. Subjects**	Approximately 930 (465 subjects per arm)
**No. Centers**	Approximately 50 stroke centers in China
**Study Duration**	Total study duration is 36~48 months. Patients will participate in the trial for 12 months
**Recruitment Period**	30~42 months
**Trial Objectives**	To test the hypothesis that acute ischemic stroke patients with large vessel occlusion who receive endovascular treatment within 24 hours of symptom onset will have improved clinical outcomes when given intravenous tirofiban therapy compared to placebo.
**Trial Design**	This will be an investigator-initiated, multicenter, randomized, double-blind, placebo-controlled trial.
**Randomization**	Eligible patients will be consecutively randomized to tirofiban or placebo group with a ratio of 1:1. Randomization is stratified according to stroke severity (National Institutes of Health Stroke Scale (NIHSS) score ≤17 vs. > 17), occlusion site (the intracranial internal carotid artery or not), and participating center.
**Inclusion Criteria**	(1) Aged 18 years or older;<br>(2) Acute ischemic stroke occurs between 4.5 and 12 hours of time

	last known well;<br>(3) Baseline NIHSS score ≤ 30;<br>(4) Baseline Alberta Stroke Program Early CT Score (ASPECTS) ≥ 6;<br>(5) Occlusion of the intracranial internal carotid artery, the first or second segment of the middle cerebral artery confirmed by CT, MR angiography, or digital subtraction angiography;<br>(6) Planned treatment with endovascular treatment by clinical care team;<br>(7) Informed consent obtained from patients or their legal representatives.
**Exclusion Criteria**	(1) CT or MR evidence of hemorrhage;<br>(2) Dual antiplatelet therapy within 1 week of the index stroke;<br>(3) Treated with intravenous thrombolysis after the index stroke;<br>(4) Currently pregnant or lactating (women patients);<br>(5) Allergy to tirofiban, radiographic contrast agents, or nitinol devices;<br>(6) Gastrointestinal or urinary tract hemorrhage within 30 days of the index stroke;<br>(7) Any major surgery within 14 days of the index stroke;<br>(8) History of heparin-induced thrombocytopenia;<br>(9) Any terminal illness with life expectancy less than 6 months;<br>(10) Preexisting neurological or psychiatric disease that would confound the neurological functional evaluations;<br>(11) Platelet count of routine blood test < 100 * 10^9/L;<br>(12) Severe renal insufficiency (glomerular filtration rate < 30ml/min or serum creatinine > 220μmol/L (2.5mg/dl));<br>(13) Arterial tortuosity and/or other arterial disease that would prevent the device from reaching the target vessel;

	(14) Unlikely to be available for 90-day follow-up.
**Treatments**	Tirofiban 10μg/kg bolus and then 0.15μg/kg/min maintenance for up to 24 hours (or matching normal saline placebo volume) will be administered intravenous infusion in the upper or lower extremity using an infusion pump starting after randomization.<br>All subjects will undergo attempted endovascular treatment and receive best medical care according to modern acute stroke care guidelines.
**Consent**	Explicit written, signed informed consent from the subject or legally authorized representative will be obtained prior to any protocol specific procedures.
**Duration of Treatment**	This study consists of one 90-day study period for each subject.<br>Subjects will be hospitalized for care after their acute stroke according to the current standard of care. Subjects are required to return to clinic on Day 90 for end-of-study procedures.
**Laboratory Tests**	In order to support the assessment of patient safety baseline, chemistry laboratory tests will be completed. At baseline, blood work will be evaluated which includes: blood cell counts, triglyceride, cholesterol, low density lipoprotein, high density lipoprotein, homocysteine, glucose, procalcitonin, HbA1C, prothrombin time, activated partial thromboplastin time, thrombin time, fibrinogen, D-dimer, international normalized ratio.<br>If the subject is female and is of childbearing potential, a pregnancy test (urine or serum point-of-care pregnancy test) must be completed and a negative test result obtained prior to inclusion in the trial.<br>Electrocardiograms will also be collected and reviewed at baseline.
**Assessment of Efficacy**	The **primary efficacy outcome** is assessed on the modified Rankin Scale (mRS) score at 90 days from randomization. The mRS, a global measure of disability, comprises of seven grades ranging from 0 (no

	symptoms) to 6 (death).<br>The **secondary clinical efficacy outcomes** include:<br>1) Proportion of patients non-disabled (mRS score 0 to 1) at 90 days or return to pre-morbid mRS score at 90 days (for patients with mRS > 1);<br>2) Proportion of mRS score 0 to 2 at 90 days;<br>3) Proportion of mRS score 0 to 3 at 90 days;<br>4) Change of the NIHSS score at 24 hours from baseline;<br>5) Change of the NIHSS score at 5-7 days or discharge if earlier from baseline;<br>6) European Quality Five Dimensions Five Level scale score at 90 days.<br>The **secondary technical efficacy outcomes** include:<br>1) Proportion of substantial reperfusion before endovascular treatment, as assessed on initial digital subtraction angiography. Substantial reperfusion is defined as a modified Treatment in Cerebral Infarction score of 2b (50 to 99% reperfusion) or 3 (complete reperfusion);<br>2) Substantial reperfusion at final angiogram;<br>3) Proportion of Rescue Drug utilization;<br>4) Recanalization at 48 hours evaluated by CT or MR angiography, assessed with the Arterial Occlusive Lesion scale.
**Assessment of Safety**	1) Symptomatic intracerebral hemorrhage rate within 48 hours;<br>2) Proportion of patients with any intracranial hemorrhage within 48 hours;<br>3) Mortality at 90 days;<br>4) Procedure-related complications such as arterial perforation, iatrogenic arterial dissection, arterial access site hematoma, retroperitoneal hematoma, etc;

	5) Incidence of serious adverse events.

Schedule of Assessments

	Baseline	Day 1~2	Day 5~7	Day 90
Eligibility criteria	X			
Demographics	X			
Medical history	X			
Prior medication	X			
Laboratory results	X			
Electrocardiography	X			
Clinical examination	X	X	X	
Weight	X			
Informed consent	X			
Randomization	X			
mRS score	X			X
NIHSS score	X	X	X	
ASPECTS	X			
Brain CT+CTA or MRI+MRA	X	X		
DSA	X			
Concomitant medication		X	X	X
Adverse event		X	X	X
EQ-5D-5L				X

1. BACKGROUND INFORMATION

The burden of stroke

Stroke is the leading cause of death and the most frequent cause of permanent disability in China[1,2]. The National Epidemiological Survey of Stroke in China (NESS-China) shown that the age-standardized stroke prevalence is 1,115 cases per 100,000 people, annual age-standardized incidence 247 cases per 100,000, and mortality 115 cases per 100,000[3]. Acute ischemic stroke (AIS) accounts for approximate 75% of all strokes, and intracranial large vessel occlusion (LVO) is a common cause of AIS, accounting for about 20%. According to this incidence and percentage, there would be approximate 518,700 LVO patients per year in China. Remarkably, LVO stroke often leads to severe disability and high mortality when comparing non-LVO stroke.

Endovascular treatment in large vessel occlusive stroke

In 1996, alteplase was approved for the treatment of AIS. However, it has several major therapeutic limitations, one of which is the low recanalization rate in LVO stroke[4,5]. To overcome this limitation, endovascular treatments (EVT) have been gradually developed over the last 20 years. Since 2015, at least 7 high-quality multicenter randomized controlled trials shown that for AIS patients caused by anterior LVO, EVT combined with usual medical care can significantly improve clinical outcomes compared with usual medical care alone[4,6-11]. Both the American Heart Association/American Stroke Association and the Chinese stroke early management guidelines strongly recommend EVT as the first line treatment for AIS due to anterior circulation LVO[12,13]. EVT include mechanical thrombectomy using stent-retriever or aspiration devices, angioplasty and/or stenting, intraarterial thrombolysis, and clot disruption using a guidewire or microcatheter (thromborrhexis), all of which may cause traumatic damage of the vascular endothelial with subendothelial matrix exposure, leading to platelet activation, adhesion, and aggregation, and potentially resulting in re-occlusion and thromboembolic complications[14,15].

The platelet glycoprotein IIb/IIIa receptor inhibitor, Tirofiban

The binding of fibrinogen or von Willebrand factor to the platelet glycoprotein IIb/IIIa receptor is the final common pathway for platelet aggregation and subsequent formation of thrombi, which can be reversibly inhibits by tirofiban, a non-peptide selective platelet

glycoprotein IIb/IIIa receptor inhibitor. Tirofiban is the most widely used glycoprotein IIb/IIIa receptor inhibitor due to its pharmacological characteristics including rapid onset of action, short half-life, high selectivity and affinity, and reversible inhibition of fibrinogen-dependent platelet aggregation. It has been proven that the combined use of tirofiban, especially when administered early, can reduce the risk of vascular complications and the need for revascularization during percutaneous coronary intervention[16]. Based on the positive experience and findings in ischemic heart disease patients who receiving percutaneous coronary intervention[17,18], many investigators have evaluated the safety and efficacy of tirofiban as an adjunctive therapy in LVO stroke patients undergoing EVT.

Several studies have suggested that tirofiban may be beneficial to patients with AIS. A randomized, placebo-controlled, open-label treatment, multicenter trial recruited 260 stroke patients who had a National Institutes of Health Stroke Scale (NIHSS) score between 4 and 18 and indicated that tirofiban might decrease mortality in the long-term follow up without increasing the incidence of intracranial hemorrhage (ICH)[19]. However, this study included LVO and non-LVO strokes and predated the era of modern endovascular stroke therapy. A multicenter retrospective study involving 148 LVO stroke patients undergoing rescue stenting who failed in mechanical thrombectomy indicated that adjuvant tirofiban is correlated with stent patency without inducing more symptomatic intracranial hemorrhage (SICH)[20]. An observational study including 180 LVO stroke patients (90 patients in each group with and without tirofiban) suggested that the clinical outcomes of the tirofiban group was better than that of the non-tirofiban group, and the incidence of SICH did not increase significantly[21]. Conversely, one study has suggested that tirofiban might actually increase the risk of fatal ICH and unfavorable clinical outcomes[22]. However, most of the available data is comprised by small, single-center, retrospective studies. There is currently no randomized controlled trial evaluating the effect of tirofiban in EVT of AIS.

2. TRIAL OBJECTIVES

Primary objective

The primary objective is to determine the efficacy of the non-peptide selective platelet glycoprotein IIb/IIIa receptor inhibitor, tirofiban, in reducing severity of disability in patients

with AIS with LVO and within 24 hours of last known well selected for EVT.

Secondary objective

The secondary objectives are to determine the efficacy of tirofiban in:

- Increasing proportion of patients non-disabled (modified Rankin Scale (mRS) score 0 to 1) or return to pre-morbid mRS score (for patients with mRS > 1)
- Improving functional independence (mRS score 0 to 2)
- Reducing re-occlusion

Leading Safety Objectives

The leading safety objectives are to determine the effect of tirofiban to patient with LVO stroke who are selected for EVT on SICH, and 90-day mortality.

3. TRIAL DESIGN

The Endovascular Treatment With versus Without Tirofiban for Stroke Patients with Large Vessel Occlusion (RESCUE BT) Trial is an investigator-initiated, multicenter, prospective, randomized, placebo-controlled, double-blind clinical trial, aiming to specifically assess the efficacy and safety of intravenous tirofiban in anterior circulation LVO stroke patients who undergo EVT within 24 hours of symptom onset. The trial has been registered at Chinese Clinical Trial Registry (www.chictr.org.cn, unique identifier ChiCTR-INR-17014167). The study patient flow outline was shown in Figure 1.

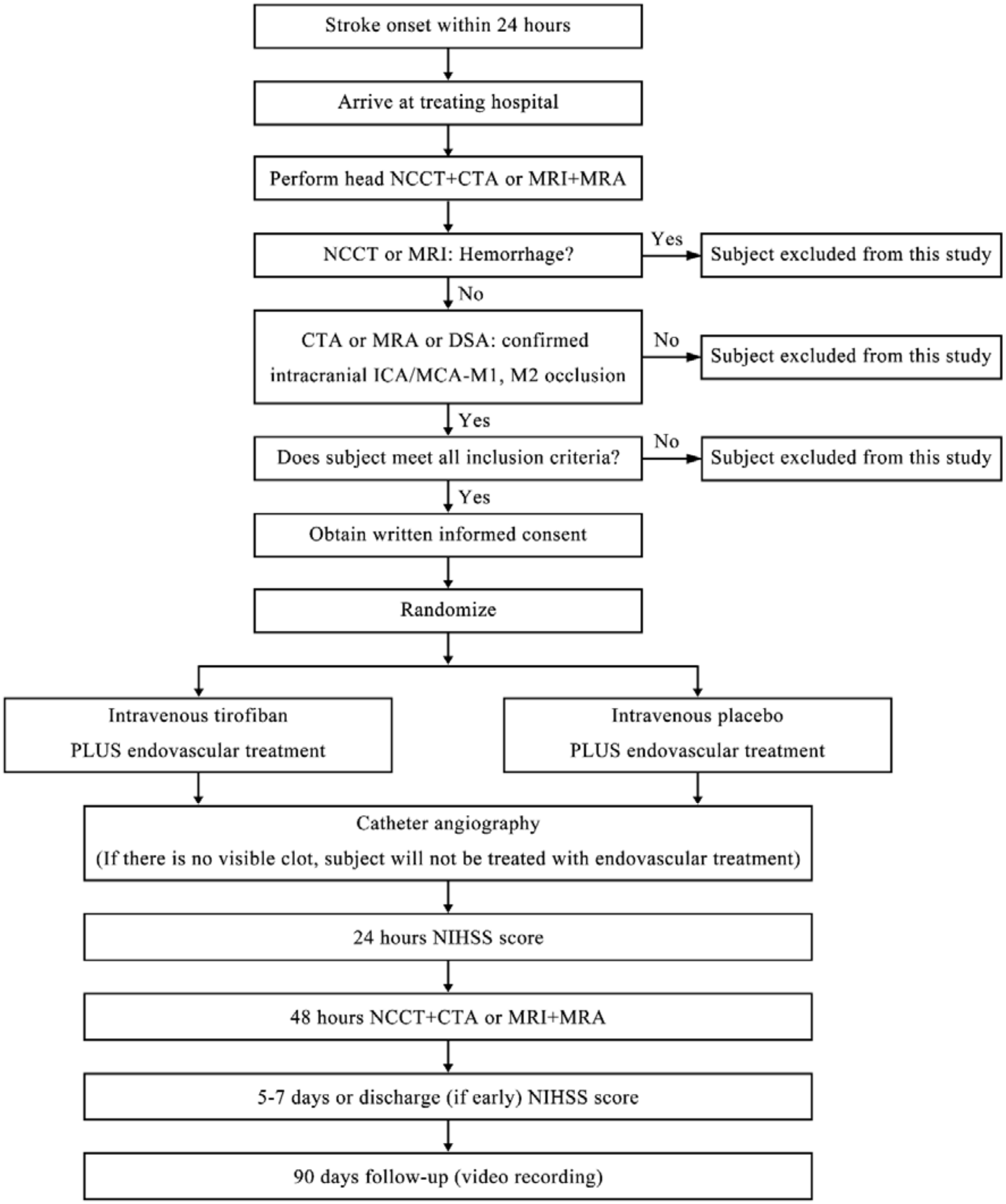

Figure 1 Study flowchart of RESCUE BT trial.

4. PATIENT POPULATION

All subjects will undergo a neurological and clinical assessment, routine laboratory blood test and baseline brain imaging. All these materials will be collected and screened by site physician. This includes standard of care use of non-contrast CT/ CT angiography or diffusion

weighted imaging/MR angiography. In order to track the potential for enrollment, each enrolling site will provide the total number of patients admitted to that site with the diagnosis of AIS-LVO. However, screening log is not required. If the patient is still eligible after completing the routine screening, the patient will be agreed (as needed) and included in the study. Patients who are randomized but do not receive study drug will still be followed up to the 90-day study period.

4.1. Inclusion criteria

(1) Aged 18 years or greater;
(2) Acute ischemic stroke occurs between 4.5 and 12 hours of time last known well;
(3) Baseline NIHSS score ≤ 30;
(4) Baseline Alberta Stroke Program Early Computed Tomography Score (ASPECTS) ≥ 6;
(5) Occlusion of the intracranial internal carotid artery, the first or second segment of the middle cerebral artery confirmed by CT, MR angiography, or digital subtraction angiography;
(6) Planned treatment with EVT by clinical care team;
(7) Informed consent obtained from patients or their legal representatives.

4.2. Exclusion criteria

(1) CT or MR evidence of hemorrhage;
(2) Dual antiplatelet therapy within 1 week of the index stroke;
(3) Treated with intravenous thrombolysis after the index stroke;
(4) Currently in pregnant or lactating on admission;
(5) Contraindication to radiographic contrast agents, nickel, titanium metals or their alloys;
(6) Gastrointestinal or urinary tract hemorrhage within 30 days of the index stroke;
(7) Any major surgery within 14 days of the index stroke;
(8) History of heparin-induced thrombocytopenia;
(9) Any terminal illness with life expectancy less than 6 months;
(10) Patients with a preexisting neurological or psychiatric disease that would confound the neurological functional evaluations;
(11) Platelet count of routine blood test < $100 * 10^9$/L;

(12) Severe renal insufficiency (glomerular filtration rate < 30ml/min or serum creatinine > 220μmol/L (2.5mg/dl));

(13) Arterial tortuosity and/or other arterial disease that would prevent the device from reaching the target vessel;

(14) Unlikely to be available for 90-day follow-up;

Patients can leave the study at any time upon request. Patients who refuse to participate in the study will be treated as usual. Patients who choose to withdraw from the study should attend the final withdrawal visit. The withdrawal date and reason should be documented in the Case Report Form (CRF), and Subject Withdrawal Form.

5. BASELINE CLINICAL AND LABORATORY EVALUATIONS

At Baseline, all subjects will undergo a routine neurological and clinical assessment (including

history, examination and vital signs), and the NIHSS.

Emergency blood work will be drawn including: blood cell counts, triglyceride, cholesterol, low density lipoprotein, high density lipoprotein, homocysteine, glucose, procalcitonin, HbA1C, prothrombin time, activated partial thromboplastin time, thrombin time, fibrinogen, D-dimer, international normalized ratio. The study will use and collect local laboratory results and will not use a central lab. The results of this blood work are not required prior to randomization.

If the subject is female and is of childbearing potential a pregnancy test (urine point-of-care pregnancy test) must be completed and a negative test result obtained prior to inclusion in the trial. Other laboratory or point-of-care testing may be performed at the discretion of the attending physicians and team.

An electrocardiogram will also be collected and reviewed at baseline.

6. PARTICIPATING CENTER ELIGIBILITY

To be fully eligible for participation in this trial, study centers were required have performed at least 80 endovascular procedures annually, including at least 50 thrombectomy procedures with the stent-retriever devices or contact aspiration devices. Moreover, all

neuro-interventionists with more than five years' experience in cerebrovascular intervention and at least 10 cases of mechanical thrombectomy annually.

7. RANDOMIZATION

Eligible patients will be randomly assigned to tirofiban or placebo group with a ratio of 1:1. Randomization will be carried out immediately via a web-based App on mobile phone or computer (www.ratjin.com) after the patient's eligibility status has been confirmed. Randomization is stratified by stroke severity (NIHSS ≤17 vs. > 17), occlusion site (the intracranial internal carotid artery or not), and participating center. The stratified randomization approach helps avoid potential imbalances between the two treatment groups that may affect the clinical outcomes and biased results. Randomization will be completely concealed by having both web-based real-time allocation and identical appearance of tirofiban and placebo bottles. All bottles will have a unique number. Subjects will be assigned a random serial number according to the time they were enrolled, and corresponding masked medications will be provided.

8. BLINDING/UNBLINDING

All trial personnel (investigators, their clinical staff, and the data management group) and patients will be blinded to treatment assignment. The Data Safety Monitoring Board (DSMB) will have access to grouped data, but not unblinded in treatment assignment.

Both the person responsible for investigational product labels and the independent statistical group that prepare reports for the DSMB will be unblinded. In addition, the person in charge of the data management group responsible for managing the programming of the randomized system will be unblinded. If it is necessary to unblind, this person will become the contact person. This person will not participate in data management and will only communicate the unblinded data listed below when contacted by the medical monitor.

If the treatment code needs to be cracked for the safety of the patient, the site principal investigator (PI) will call the medical monitor for all unblinding queries. The medical monitor will discuss with the researcher whether it is necessary to unblind the patient. If the medical monitor determines that the PI should be unblinded for the patient, the medical monitor will

contact with the person in charge of the data management group by e-mail or phone, instructing the unblinding of the specific patient. Members of the unblinding data management team will provide only to the site PI with allocation information, the unblinding date, site number, PI name, and patient number via email. Any cases unblinded in this way will be recorded in the central file. Only the physician who requests the unblinding will receive the unblinding message. Study drugs will be stopped afterwards. It is not expected that there will be any clinical cases that need to be unblinded. The randomization data will be strictly confidential, and only authorized personnel can access it until the data is locked at the end of the study.

9. CONTENTS OF INVESTIGATIONAL PRODUCT KIT

Tirofiban and its placebo (saline) are manufactured and provided by Lunan Pharmaceutical Group Co., Ltd., Linyi, China. Aspirin and Clopidogrel are produced by Bayer HealthCare Manufacturing S.r.l. and Sanofi Winthrop Industrie respectively, and purchased and provided by Lunan Pharmaceutical Group Co., Ltd., Linyi, China. All study medication will be manufactured, tested, released, packaged, labelled, and shipped in accordance with Good Manufacturing Practice, Good Clinical Practice (GCP) guidelines, and any national regulatory requirements.

Tirofiban and its placebo (saline) are prepared in numbered and are visually identical (such as labeling, dosage form, size, and color), except for a unique number. Tirofiban and saline placebo will be packed in sterile, disposable, individually labeled bottles. Each kit has a unique six-digit identification number and will be stored in a safe location at room temperature on the clinical site with restricted access.

9.1. Tirofiban group

The box of tirofiban group contains tirofiban hydrochloride injection (12.5mg/250ml*2 bottles, the bottles are marked as Study Drug) and saline placebo (250ml*2 bottles, Rescue Drug).

9.2. Placebo group

The box of control group contains saline placebo (250ml*2 bottles, Study Drug) and tirofiban hydrochloride injection (12.5mg/250ml*2 bottles, Rescue Drug).

10. TREATMENTS

Eligible patients will be randomly assigned a number corresponding to a blinded sealed medication kit that is given to each patient. It is recommended that the usage of Study Drugs be started within 5 minutes after randomization.

Patients in the tirofiban group and the placebo group will receive intravenous bolus followed by continuous infusion of tirofiban and saline placebo (Study Drug: 10μg/kg bolus and then 0.15μg/kg/min maintenance for up to 24 hours), respectively. Endovascular treatment should be initiated as soon as possible. The treatment technique choice is at the treating neuro-interventionalists' discretion. EVT consisted of mechanical thrombectomy, thromboaspiration, balloon dilation, stenting, intra-arterial thrombolysis, clot disruption using a guidewire or microcatheter (thromborrhexis), or various combinations of these approaches. However, the intra-arterial infusion of thrombolytics (such as alteplase or urokinase), Study Drug, Rescue Drug, or other glycoprotein IIb/IIIa inhibitors is not recommended and will be recorded in the concomitant medications list of CRF. After recanalization of the target artery, all patients will get stroke unit care and postoperative management.

We acknowledge that patients undergoing mechanical thrombectomy might occasionally require rescue treatment with balloon angioplasty and stenting. If the antegrade blood flow cannot be maintained after angioplasty and/or stenting, Rescue Drug can be used. The Rescue Drug is available as a second medication bottle in the study kits and it used in the same manner as the Study Drug. Rescue Drug and Study Drug are injected intravenously through two different infusion pipelines at the same time.

At the 20th hour after using the Study Drug, the two treatment groups will receive oral antiplatelet therapy with aspirin and/or clopidogrel tablets. Patients undergoing angioplasty/stenting will be given dual-antiplatelet therapy with aspirin and clopidogrel. In other cases, mono-antiplatelet therapy is given. If the patient takes aspirin before the index stroke, he will continue to use aspirin, while those who use clopidogrel will use clopidogrel. At the 24th hour, the Study Drug will be discontinued. After that, all patients will be managed according to the current American Heart Association/American Stroke Association guidelines[13]. Risk factors such as hypertension, atrial fibrillation, diabetes mellitus,

hyperlipidemia, decreased physical activity, and smoking should be treated appropriately. The treatment scheme is showed in Figure 2.

Intravenous heparin may be used during the thrombectomy procedure at the discretion of the operator. In addition, the post-procedural use of subcutaneous heparin or low-molecular-weight heparin for deep vein thrombosis prophylaxis is permitted. However, the use of any other (intravenous or oral) anticoagulants or antiplatelet agents is not allowed during the first 24 hours post randomization. Concomitant medications, both within one week before enrollment and during the whole course of the study, will be documented.

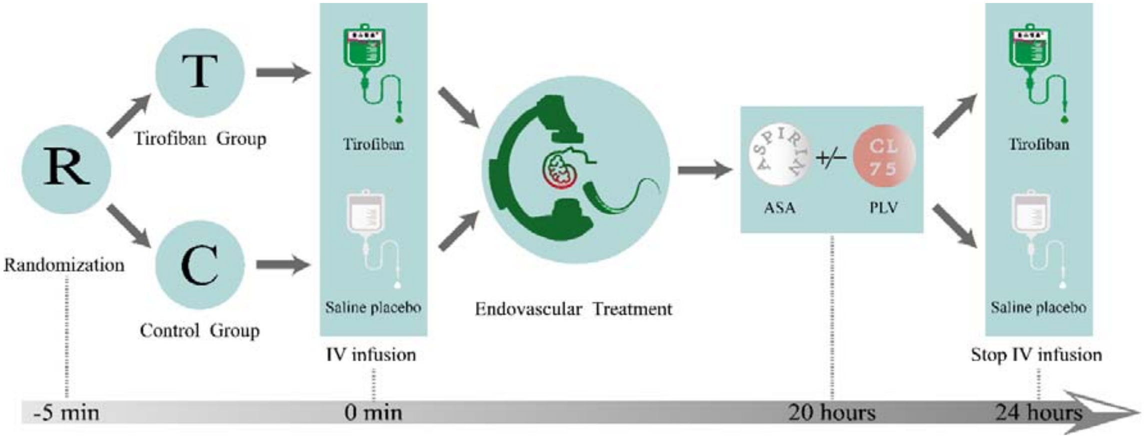

Figure 2 The treatment flow chart of the RESCUE BT trial

11. OUTCOMES

11.1. Primary Efficacy Outcome

The primary end-point is the distribution of global disability on the mRS score at 90 days after randomization. To ensure the reliability, evaluability, and traceability of the mRS score, we will keep patients' video or voice version of follow-up at 90 days except those who die or refuse to take a video. The mRS scores will be centrally assessed by two independent certified neurologists in a blinded manner by the use of the video or voice recording. Disagreements are resolved by consensus. For those who decline to participate in a video or voice recording, the outcomes will be determined in person by site neurologists blinded to the treatment allocation.

11.2. Secondary Efficacy Outcomes

11.2.1. Secondary Clinical Efficacy Outcomes

(1) Proportion of patients non-disabled (mRS score 0 to 1) or return to pre-morbid mRS score at 90 days (for patients with mRS > 1);

(2) Proportion of patients with functional independence at 90 days (mRS score 0 to 2);

(3) Proportion of patients ambulatory or bodily needs-capable or better (mRS score 0 to 3);

(4) Change of the NIHSS score at 24 hours from baseline;

(5) Change of the NIHSS score at 5-7 days or discharge if earlier from baseline;

(6) Health-related quality of life, assessed with the European Quality Five Dimensions Five Level scale (EQ-5D-5L) at 90 days.

11.2.2. Secondary Technical Efficacy Outcomes

(1) Proportion of substantial reperfusion before endovascular treatment, as assessed on initial digital subtraction angiography. Substantial reperfusion is defined as a modified Treatment in Cerebral Infarction score of 2b (50 to 99% reperfusion) or 3 (complete reperfusion)[23];

(2) Proportion of substantial reperfusion at final angiogram;

(3) Proportion of Rescue Drug utilization;

(4) Proportion of vessel recanalization at 48 hours evaluated by CT or MR angiography, assessed with the Arterial Occlusive Lesion scale;

11.3. Safety Outcomes

(1) SICH rate within 48 hours. ICH will be evaluated according to the Heidelberg Bleeding Classification. SICH is diagnosed if the new observed ICH is associated with any of the following conditions: 1) NIHSS score increased more than 4 points than that immediately before worsening; 2) NIHSS score increased more than 2 points in one category; 3) Deterioration led to intubation, hemicraniectomy, external ventricular drain placement or any other major interventions. Additionally, the symptom deteriorations could not be explained by causes other than the observed ICH. Hemicraniectomy will be defined as that surgical procedure used to decompress the swollen hemisphere;

(2) Proportion of patients with any ICH within 48 hours;

(3) Mortality at 90 days. Mortality rates are defined as the number of deaths observed divided by the number of subjects observed over the 90-day study period;

(4) Procedure-related complications such as arterial perforation, iatrogenic arterial dissection, embolization in previously uninvolved vascular territory, arterial access site hematoma, and retroperitoneal hematoma. Arterial perforation will be defined at angiography by the operator and associated with subarachnoid hemorrhage. Iatrogenic arterial dissection will be defined at angiography by the operator. Arterial access site hematoma will be assessed as a complication of arterial access puncture and defined by clinical examination and anatomic imaging. Retroperitoneal hematoma will be assessed as a complication of groin puncture and defined by imaging (ultrasound or CT or MR angiography). The definition of embolization in previously uninvolved vascular territory is noted after recanalization of the primary occlusion site, any vessel occlusions distal from the primary occlusion site are considered emboli due to periprocedural thrombus fragmentation.

(5) Incidence of serious adverse events.

12. ASSESSMENT OF EFFICACY

12.1. The Modified Rankin Scale

The mRS is a valid and reliable clinician-reported measure of global disability that has been widely applied for evaluating recovery from stroke. It is a scale used to measure functional recovery (the degree of disability or dependence in daily activities) of people who have suffered a stroke[24,25]. mRS scores range from 0 to 6, with 0 indicating no residual symptoms; 5 indicating bedbound, requiring constant care; and 6 indicating death. The mRS score will be obtained at Day 90. Premorbid mRS status will also be obtained retrospectively at 24 Hours. The mRS will only be scored by those who have been trained and certified to use this scale using the table below.

Table 1. Level of Function Survey - mRS-9Q

Q 1: Do you have any symptoms that are bothering you?	□Yes □No
Q 2: Are you able to do the same work as before?	□Yes □No
Q 3: Are you able to keep up with your hobbies?	□Yes □No
Q 4: Have you maintained your ties to friends and family?	□Yes □No
Q 5: Do you need help making a simple meal, doing household chores, or	□Yes □No

balancing a checkbook?	
Q 6: Do you need help with shopping or traveling close to home?	□Yes □No
Q 7: Do you need another person to help you walk?	□Yes □No
Q 8: Do you need help with eating, going to the toilet, or bathing?	□Yes □No
Q 9: Do you stay in bed most of the day and need constant nursing care?	□Yes □No

mRS-9Q: the mRS calculator (http://www.modifiedrankin.com/)

12.2. The National Institutes of Health Stroke Scale

The NIHSS is a standardized neurological examination score that is a valid and reliable measure of disability and recovery after acute stroke[26]. Scores range from 0 to 42, with higher scores indicating more severe disability. The scale includes measures of level of consciousness, extra ocular movements, motor and sensory tests, coordination, language and speech evaluations. The NIHSS will be administered at baseline, at 24 hours from baseline, and Day 5-7or discharge. The NIHSS will only be scored by those trained and certified in the use of this scale. In this trial, we will be kept video version of NIHSS score except those who die or refuse to take a video. The NIHSS score will be centrally assessed by two independent certified neurologists in a blinded manner via the video. Disagreements are resolved by consensus. For those who decline to participate in a video recording, the outcomes will be determined in person by site neurologists blinded to the treatment assignment.

12.3. European Quality Five Dimensions Five Level scale

The EQ-5D-5L is a generic instrument for describing and valuing health. It is based on a descriptive system that defines health in terms of five dimensions: Mobility, Self-Care, Usual Activities, Pain/Discomfort, and Anxiety/Depression[27]. Each dimension has five response categories corresponding to: no problems, slight, moderate, severe and extreme problems[28]. The instrument is designed for self-completion, and respondents also rate their overall health on the day of the interview on a 0-100 hash-marked, vertical visual analogue scale. The EQ-5D-5L will be administered on Day 90 by those trained in the use of this scale.

13. ASSESSMENT OF SAFETY

13.1. Symptomatic Intracranial Hemorrhage

In addition to the efficacy endpoints, we will investigate SICH being the most feared complication of intravenous tirofiban for LVO stroke. SICH as defined in Heidelberg Bleeding Classification. SICH is diagnosed if the new observed ICH is associated with any of the following conditions: 1) NIHSS score increased more than 4 points than that immediately before worsening; 2) NIHSS score increased more than 2 points in one category; 3) Deterioration led to intubation, hemicraniectomy, external ventricular drain placement or any other major interventions. Additionally, the symptom deteriorations could not be explained by causes other than the observed ICH.

13.2. Mortality at 90 days

In addition to SICH, mortality at 90 days is also one of the most important safety endpoints of the RESCUE BT trial. Mortality rates are defined as the number of deaths observed divided by the number of subjects observed over the 90-day study period.

The Highly Effective Reperfusion evaluated in Multiple Endovascular Stroke Trial collaboration, a pooled analysis of patient-level data, shown that stroke mortality rate after EVT is 15.3%[29]. For LVO stroke patients treated between 6 hours and 24 hours mortality is 19%[6].

13.3. Adverse Event Definitions

13.3.1. Adverse Event

Any adverse change in health or the appearance of or worsening of any undesirable sign, symptom or medical condition occurring after enrollment into the trial will be recorded as Adverse Event (AE) whether or not it is considered to be related to the study drug. An AE also includes a new illness; aggravated in severity of frequency from the baseline condition, abnormal results of diagnostic procedures, or a combination of the above. Pre-existing medical conditions are not to be reported as AEs.

13.3.2. Serious Adverse Event

A serious adverse event (SAE) is any untoward medical occurrence that at any dose: Result in death; Are life-threatening or fatal; Require or prolong hospitalization; Result in persistent or significant disability/incapacity, Constitutes a congenital anomaly or birth defect, or; Significant medical event.

A SAE can also be an important medical event that may not result in death, be life-threatening, or require hospitalization, but may jeopardize the subject and may require medical or surgical intervention to prevent one of the outcomes listed in this definition. For example, any new diagnosis of cancer (made after study enrollment) is considered an important medical event. Because our primary safety outcomes for the trial are also SAEs by definition, they will be reported dually as SAEs and as outcomes. SAEs should be managed according to the best current standard of care.

All deaths occurring during the follow up to Day 90 will be reported as an SAE. When reporting a death, the event or condition that caused or contributed to the fatal outcome should be reported as a single medical concept.

AE occurring within 30 days of randomization and all SAEs will be reported in the CRF.

Table 2. Severity and relationship definitions of Adverse Event.

**AE Severity**	
Mild	Awareness of sign or symptom but easily tolerated
Moderate	Discomfort sufficient to cause interference with normal activities.
Severe	Incapacitating, with inability to perform normal activities.
**AE Relationship**	
Related	A clinical event, including laboratory test abnormality, where there is a "reasonable possibility" that the SAE was caused by the study drug, meaning that there is evidence or arguments to suggest a causal relationship.
Probably	A clinical event, including laboratory test abnormality, with a reasonable time sequence to drug administration, unlikely to be attributed to concurrent disease or other drugs or chemicals, and which follows a clinically reasonable response on withdrawal.
Possibly	A clinical event, including laboratory test abnormality, with a reasonable time sequence to drug administration, but which could also be explained by concurrent disease or other drugs or chemicals.

	Information on drug withdrawal may be lacking or unclear.
Unrelated	This category is applicable to AEs which are judged to be clearly and incontrovertibly due to extraneous causes (diseases, environment, etc.) and do not meet the criteria for drug relationship listed for the above-mentioned conditions.

13.4. Clinical Management of Adverse Events

13.4.1. Early Study Drug Cessation

The intervention is the intravenous administration of Study Drug over 24 hours to subject undergoing EVT. If any SAE is observed during dosing, dosing shall be immediately terminated. If any moderate or severe AE is observed, the physician may terminate drug administration at his/her discretion.

13.4.2. Identification of Adverse Events by the Investigator

AE monitoring and reporting will be followed-up until Day 30. SAEs will be followed through the final study exit visit (Day 90 Visit or death or end of study whichever is sooner) or until the subject is deemed “lost to follow-up”.

AE identification while the subject is admitted to the acute stroke hospital will be collected via acute stroke hospital patient records and verbal histories from the subject or legally authorized representative. For follow up visits after discharge from the acute stroke hospital the subject (or legally authorized representative if the subject is not able to respond to the questions) will be asked about the occurrence of AEs since the last contact, and if available, from records at the acute stroke hospital. AEs that were ongoing at the last contact will be updated with a stop date or confirmed as ongoing. AE collection will continue until Day 30, and SAE to Day 90 or the final contact.

A consistent methodology of eliciting AEs at all subject evaluation timepoints will be used. Non-directive questions include: How have you felt since your last clinical visit/hospital discharge? Have you had any new or changed health problems since you were last here? Have you had any unusual or unexpected worsening of your underlying medical condition or overall health? Have there been any changes in the medicines you take since your last clinical visit/hospital discharge?

Diagnosis versus signs and symptoms for the purpose of AE reporting: if known at the time of reporting, a diagnosis should be reported rather than individual signs and symptoms. However, if a constellation of signs and/or symptoms cannot be medically characterized as a single diagnosis it is acceptable to report the information that is ultimately available.

13.4.3. Reporting of Adverse Events

AEs should be reported as they occur on the CRF. Documentation must be supported by an entry in the subject's file. Each event should be described in detail along with start and stop dates, severity, relationship to investigational product as judged by the investigator, action taken, and outcome.

13.4.4. Prompt Reporting of Serious Adverse Events

Serious Adverse Events require immediate action.

Once an investigator becomes aware that an SAE has occurred, he/she will immediately notify the clinical coordinator via telephone within one working day. The study SAE form must be completed as thoroughly as possible with all available details of the event, signed by the investigator (or appropriately qualified designee), and faxed to the study manager within one working day of first becoming aware of the event. The equivalent SAE page should be filled in on the CRF.

If the investigator does not have all information regarding an SAE, he/she will not wait to receive additional information before notifying the study monitor of the event and completing the form. The form will be updated when additional information is received.

The investigator will always provide an assessment of causality at the time of the initial report as described previously. If data obtained after reporting indicates that the assessment of causality is incorrect, then the SAE form may be appropriately amended, signed and dated, and resubmitted.

In accordance with local Ethics Committee requirements, the investigator must also notify their Ethics Committee of any SAEs according the guidelines of the Ethics Committee. The investigator and others responsible for subject care should institute any supplementary investigations of SAEs based on their clinical judgement of the likely causative factors. This may include seeking further opinion from a specialist in the field of the adverse event or requesting extra tests. If a subject dies, any post-mortem findings, including histopathology

will be provided if available. No medical help, diagnosis, or advice should be withheld from the subject due to an inability to contact the study manager/medical monitor.

14. DATA SAFETY MONITORING BOARD

The independent DSMB will be composed of an experienced neurologist, an interventionalist, and a biostatistician, which are neither involved in the trial nor affiliated with the sponsor. The DSMB will meet at least once a year, and is provided with structured unmasked reports, prepared by the trial statistician, for their reference only. DSMB is responsible for recommendations to the executive committee regarding stopping or extending the trial. In addition, the DSMB will review the occurrence of SAEs and make recommendations to the executive committee regarding safety of the trial. Interim safety analysis will be conducted when approximate 465 patients have completed their Day 90 assessments. If there are concerns about the safety of patients, this board will make a recommendation to the trial steering committee about continuing, stopping, or modifying the trial. No formal interim efficacy or futility analyses are planned.

15. IMAGING CORE LABORATORY

Centralized imaging core laboratories will be used in this trial to provide consistent assessment of all the images. CT/MR and angiographic images will be independently reviewed by two independent central imaging core laboratories respectively. CT/MR core laboratory will review CT/MR images obtained at baseline and within 24 hours for confirmation of inclusion criteria, ASPECTS score, collateral circulation classification, and presence/absence of hemorrhage. Angiographic core laboratory will review angiographic images from the procedure to determine clot location, collateral compensation, and recanalization. CT/MR core laboratory will be independent from the angiographic core laboratory to ensure the CT/MR core laboratory is blinded to the treatment allocation.

16. CLINICAL EVENTS COMMITTEE

The Clinical Events Committee will be comprised of three expert physicians independent of the investigational sites. This committee will validate all the complications that occur over the

course of the study and categorized for severity and relatedness according to the definition in the AE section. The Clinical Events Committee can request any additional source information and images supporting the AEs to assist with the adjudication.

17. STATISTICS

17.1. Sample size estimates

The distribution of 90-day mRS scores in the placebo group of this trial is derived from the Highly Effective Reperfusion evaluated in Multiple Endovascular Stroke collaboration's intervention population who are ineligible for alteplase as following: mRS 0: 10.2%, mRS 1: 15.7%, mRS 2: 17.6%, mRS 3: 18.5%, mRS 4: 7.4%, mRS 5: 7.4%, and mRS 6: 23.1%[29]. It is assumed that a favorable treatment effect with a common odds ratio of 1.50 would be achieved in the tirofiban group compared with the placebo group, corresponding to 8.5% absolute increase in the proportion of 90-day mRS score 0 to 1. A total sample size of 930 (465 patients per arm) patients would provide 90% power at a 2-sided significance level of 0.05, taking 15% attrition rate into account. This estimation is performed based on PASS (NCSS, LLC. Kaysville, Utah, USA) version 15.0.

17.2. Analysis Populations

17.2.1. Intention-to-Treat Population

The primary efficacy analysis will be conducted in the intention-to-treat (ITT) population, defined as all subjects randomized into the trial with grouping by randomized treatment, regardless of treatment actually received. An ITT analysis will also be conducted for the secondary endpoints, with subject grouped according to the randomized (intended) treatment. Ineligible patients and patients who receive neither Study Drug therapy nor endovascular treatment should be excluded from the ITT population.

17.2.2. Per-Protocol Population

The primary analysis will be repeated on the per-protocol population, defined to be all subjects randomized and treated, with no major protocol deviations. This population will be determined via blinded review of protocol deviations at the end of the trial before database lock and unblinding. Prior to unblinding, the imaging from each subject at the time of inclusion will be adjudicated to determine whether they have met the criteria for endovascular

intervention, and hence for the trial. This will include review of baseline non-contrast CT and CT angiography. Subjects who do not meet the imaging criteria outlined in the trial inclusion/exclusion criteria, will not be included in the per-protocol population.

The following situations are major protocol deviations: (1) Rescue Drug is used, (2) no EVT procedure is conducted, and (3) procedure-related hemorrhage (e.g., artery perforation) resulting in premature termination of Study Drug and EVT and failure of substantial reperfusion.

17.2.3. As-Treated Population

The same as the ITT population except subjects are analyzed based upon actual treatment received. Subjects who receive both Study Drug and Rescue Drug are included in the tirofiban arm, and subjects who receive neither Study Drug nor Rescue Drug are included in the placebo arm.

17.2.4. Safety Population

The safety population will consist of all patients who received any dose of Study Drug. The main analyses will be frequency of SICH and 90-day mortality. It is expected that the safety population and the ITT population will be near-identical. Full details will be specified in detail in the Statistical Analysis Plan.

Patients who withdraw informed consent immediately after randomization and are not to receive any treatment should be excluded from all analysis populations.

17.3. Analysis of Primary Efficacy Outcome

The primary efficacy outcome is the differences in 90-day mRS outcome across the full mRS scale between tirofiban and placebo groups and will be analyzed using ordinal logistic regression unless the proportional odds assumption is violated, in which case the assumption-free Wilcoxon-Mann-Whitney generalized odds ratio approach will be used. The proportional odds assumption will be tested using a Brant test.

17.4. Analysis of Key Secondary Outcome

For the key secondary outcome analysis, the proportions of 90-day mRS 0-1 or return to pre-morbid mRS score (for patients with mRS > 1) outcomes will be compared between tirofiban and placebo arms using binary logistic regression model. Both adjusted and

unadjusted odds ratio their 95% confidence intervals will be reported. Between-group differences will be tested using a chi-square test.

The fixed sequential order for testing in the ITT population is:

1) Primary efficacy outcome

2) Key secondary outcome analysis

3) Secondary and safety endpoints, as specified in the order presented below

17.5. Analysis of Secondary Outcomes and Safety Outcomes

Other secondary outcomes analyses will be carried out in the following order:

(1) Proportion of patients functionally independent (mRS score 0 to 2) at 90 days;

(2) Proportion of patients ambulatory or bodily needs-capable or better (mRS score 0 to 3);

(3) Change of the NIHSS score at 24 hours from baseline;

(4) Change of the NIHSS score at 5-7 days or discharge if earlier from baseline;

(5) Health-related quality of life, assessed with the EQ-5D-5L at 90 days.

(6) Substantial reperfusion before endovascular treatment, as assessed on initial digital subtraction angiography. Substantial reperfusion is defined as modified Treatment in Cerebral Infarction score of 2b (50 to 99% reperfusion) or 3 (complete reperfusion)[23];

(7) Substantial reperfusion at final angiogram;

(8) Proportion of Rescue Drug utilization;

(9) Recanalization at 48 hours evaluated by CT or MR angiography, assessed with the Arterial Occlusive Lesion scale;

(10) SICH rate within 48 hours. SICH will be evaluated according to the Heidelberg Bleeding Classification;

(11) Proportion of patients with any ICH within 48 hours;

(12) Mortality at 90 days;

(13) Procedure-related complications including arterial perforation, vessel dissection, embolization into a new vascular territory, and puncture access complications;

(14) Incidence of serious adverse events.

17.6. Adjustment for Covariates and Subgroup Analyses

In addition to the primary and secondary analyses adjusting for age, baseline NIHSS score, baseline ASPECTS score, occlusion site, and onset-to-randomization time, exploratory

analyses will be conducted to determine the potential roles of common baseline characteristics and assess potential heterogeneity of treatment effect across subgroups. Specific subgroups of interest include the age > median vs. ≤ median years old, sex male vs. female, baseline NIHSS score > median vs. ≤ median, baseline ASPECTS > median vs. ≤ median, occlusion location (intracranial internal carotid artery occlusion: no vs. yes), stroke etiology (large artery atherosclerosis: no vs. yes), rescue therapy (no vs. yes), onset to randomization time > median vs. ≤ median, and other variables of interest. Full details will be specified in detail in the Statistical Analysis Plan.

17.7. Handling of Missing Data

Every effort will be made to keep missing data, particularly the Day 90 outcome assessments, to a minimum. Regular reminders of patient follow-up due dates will be provided to participating centers to facilitate scheduling of follow-up visits. Nevertheless, some missing data may be inevitable due to, for example, loss to follow-up. Since all randomized patients will be included in the primary endpoint analysis (ITT), every patient must have a 90-day mRS score. Patients who die within the study period will be assigned the worst score on all outcome measures and taken into the analysis.

Missing outcome data of mRS scores at 90 days in the lead analysis will be imputed using multiple imputation. Complete case and worst-case analyses will be performed as sensitivity analyses. In the worst-case analysis, if the patient was identified to be alive, we will assign a score of 5. Proportions of missing values for all variables will be reported. Variables that will be used to adjust the primary and secondary effect analyses (age, NIHSS score and ASPECTS at baseline, time to randomization, and site of occlusion) are designated as key variables. Missing values for these variables (if any) will be analyzed for randomness and imputed with standard methods.

18. DIRECT ACCESS TO SOURCE DATA/DOCUMENTS

The sponsor or delegate will be permitted to visit the study facilities at any reasonable time in order to maintain current, detailed knowledge of the study through review of the records, source documents, observation, and discussion of the conduct and progress of the study. In addition, the sponsor will maintain regular telephone and written communication with all

investigators through the coordinating center. The sponsor (or delegate) will be given complete access to all components of the study facility that pertain to the conduct of this study, and may be present to observe any aspect of the conduct of the study by medical and paramedical staff, including but not limited to drug preparations, dosing, sample collections, and clinical observations. CRFs will be monitored with sufficient frequency to assess the following: Subject randomization, compliance with protocol procedures, the completeness and accuracy of data entered into the CRFs, verification of CRF data against original source documents, and occurrence of AEs. Adequate time and all documents for these monitoring visits must be made available by the investigators. The investigators will permit trial-related monitoring, audits, Research Ethics Board/Institutional Review Board (REB/IRB) review, and regulatory inspections, providing direct access to source data/documents.

19. STUDY MONITORING AND QUALITY ASSURANCE

The investigators promise to conduct the RESCUE BT trial in accordance with this trial protocol, International Conference on Harmonization-GCP (ICH-GCP) Guidelines and applicable regulatory requirements.

The investigators must ensure compliance with all procedures required by the trial protocol and all research procedures (including safety rules) provided by the sponsor. The investigators agree to provide reliable data and all information required by the trial protocol in an accurate and legible manner according to the instructions provided, and to ensure direct access to the source documents by the sponsor's representative.

The sponsor of this clinical trial is responsible to the health authorities and takes all reasonable measures to ensure the correct implementation of the clinical trial protocol in terms of ethics, clinical trial protocol compliance, and the completeness and validity of the data recorded on the CRF.

The main responsibility of the monitoring team is to help researchers and sponsors to ensure that all aspects of clinical trials are highly ethical, scientific, professional and standardized. According to the ICH-GCP guidelines, the monitoring team must check CRF entries based on source documents, unless predetermined.

The monitoring team will regularly contact the centers through field visits, emails or phone calls, and send inspectors to assess the progress of the research, the compliance of investigators and patients with the research protocol, and to resolve urgent issues. During these inspection visits, the inspector will work with the on-site investigator. The main aspects of inspection and monitoring are as follows (not exclusive): patient informed consent, patient recruitment and follow-up, serious adverse event recording and reporting, study drug supply, treatment compliance of study drug group participants, study drug count, concomitant treatment and data quality.

20. PROTOCOL AMENDMENTS

The Protocol will not be modified by the Investigator without first obtaining the agreement of the other in writing. During the trial, any amendment or modification to the protocol should be submitted to the REB/IRB for approval prior to implementation. It should also be informed of any event likely to affect the safety of patients or the continued conduct of the trial, in particular any change in safety. If requested, a progress report will be sent to the REB/IRB annually and a summary of the trial's outcome at the end of the trial.

21. ETHICAL CONSIDERATION

This research followed the ethical principles of the Helsinki Declaration. This protocol and the consent forms will be submitted to each hospital's REB/IRB. Before initiation of the study, a copy of the REB/IRBs' approval letters will be provided to the sponsor and the membership list of the REB/IRB will be kept on file. To make sure the subjects fully understand about this trial, the investigators must provide the patients or their legal representatives with detailed information about the clinical trial, including the purpose of the trial, possible benefits and risks, and the rights/obligations. Subjects have the right to withdraw from the study at any time if they wish to do so. The privacy protection of subjects has to be ensured. The patients or their legal representatives give their written informed consent prior to the study. Each patient must leave contact information to the investigator of the participating center. At the same time, the investigator must leave his own phone number to the patient so that the patient can find the investigator at any time. Ethical approval for the study was obtained by the Ethics

Committee of the participating centers. SAEs will be reported to the REB/IRB according to their requirements.

22. DATA HANDLING AND RECORD KEEPING

22.1. Data Handling

During the trial, clinical data reported in the CRFs will be integrated into the clinical database under the responsibility of the Sponsor or their qualified representative. Quality control in the form of computerized logic and/or consistency checks will be systematically applied in order to detect errors or omissions. In addition, safety reviews may be performed several times by the Sponsor's staff in the course of the trial. Any questions pertaining to the reported clinical data will be submitted to the investigator for resolution. Each step of this process will be monitored through the implementation of individual passwords to maintain appropriate database access and to ensure database integrity.

After integration of all corrections in the complete set of data, the database will be released for statistical analysis.

22.2. Data Retention

The double reviewed CRF and imaging data will be sent to the data management group. The person in charge of the data management group will check and sign the receipt form. The CRF will be kept by the research center after data entry is completed.

22.3. Case Report Forms

For each subject randomized, an CRF must be completed and signed by the investigator. If a subject withdraws from the study, the reason must be noted on the CRF. All forms should be completed within five business days of subject visit. All corrections will be tracked in the CRF audit trail. The investigator should ensure the accuracy, completeness, legibility, and timeliness of the data reported to the sponsor in the CRFs and in all required reports.

22.4. Confidentiality

All study investigators at the clinical sites must ensure that the confidentiality of personal identity and all personal medical information of study subjects are maintained at all times. Researchers who use information about the health of their research participants are required, except in specific circumstances, to get written permission to use their participant's Protected

Health Information for the research study. Each participating clinical center is expected to comply with its individual performance site's requirements established for compliance of the local confidentiality policies.

23. USE OF INFORMATION AND PUBLICATION

All information concerning the RESCUE BT trial supplied to the investigators by the Steering Committee and not previously published is considered confidential and shall remain the sole property of the RESCUE BT Steering Committee. The investigator agrees to use this information only in accomplishing the study and will not use it or the data generated from the study for other purposes without first obtaining the written authorization from the RESCUE BT Steering Committee.

It is understood that the RESCUE BT Steering Committee may disclose this information as required to other RESCUE BT clinical investigators or to government regulatory agencies. The investigator understands that she or he has the obligation to provide complete test results and all data collected during this study to the Steering Committee.

A writing committee will be formed to review and publish the data from the study. This committee will consist of the Steering Committee and a subset of investigators. The writing committee will write/review all drafts of abstracts and full-length manuscripts and will choose the appropriate journal (for manuscripts) or meeting (for abstracts) for submission.

The RESCUE BT Steering Committee commits that when the study is completed, the data from this study will be published, regardless of the outcome of the study and the trial will be listed on the Chinese Clinical Trial Registry website.

24. FUNDINGS

RESCUE BT trial is an investigator-initiated study which is organized by the second affiliated hospital of the Third Military Medical University and conducted in about 50 comprehensive stroke centers in China. The authors disclosed receipt of the following financial support: (1) Lunan Pharmaceutical Group Co., Ltd., China, and (2) National Natural Science Foundation of China (No. 81901236, 82071323, and 81801157). The funders had no involvement in the

study design, data collection, analysis and interpretation, writing or decision to submit the paper.

Appendix 1 – Classification of Subtype of Acute Ischemic Stroke

The TOAST classification system includes five categories: 1) large-artery atherosclerosis, 2) cardioembolism, 3) small-artery occlusion (lacunae), 4) stroke of other determined etiology, and 5) stroke of undetermined etiology (Table 3)[31]. Diagnoses are based on clinical features and on data collected by tests such as brain imaging (CT/MR), cardiac imaging (echocardiography, etc.), duplex imaging of extracranial arteries, arteriography, and laboratory assessments for a pro-thrombotic state.

The physician can apply the clinical and imaging findings when first assessing the patient and then consider the results of other diagnostic tests later. An important part of the classification is the ability of the physician to categorize a specific subtype diagnosis as probable or possible based on the degree of certainty. A "probable" diagnosis is made if the clinical findings, neuroimaging data, and results of diagnostic studies are consistent with one subtype and other etiologies have been excluded. A "possible" diagnosis is made when the clinical findings and neuroimaging data suggest a specific subtype but other studies are not done. Because many patients will have a limited number of diagnostic tests, the probable and possible subcategorizations allow the physician to make as precise a subgroup diagnosis as can be achieved.

Large artery atherosclerosis

These patients will have clinical and brain imaging findings of either significant (>50%) stenosis or occlusion of a major brain artery or branch cortical artery, presumably due to atherosclerosis (Table 4). Clinical findings include those of cerebral cortical impairment (aphasia, neglect, restricted motor involvement, etc.) or brain stem or cerebellar dysfunction. A history of intermittent claudication, transient ischemic attacks in the same vascular territory, a carotid bruit, or diminished pulses helps support the clinical diagnosis. Cortical or cerebellar lesions and brain stem or subcortical hemispheric infarcts greater than 1.5 cm in diameter on CT or MR are considered to be of potential large-artery atherosclerotic origin. Supportive evidence by duplex imaging or arteriography of a stenosis of greater than 50% of an appropriate intracranial or extracranial artery is needed. Diagnostic studies should exclude potential sources of cardiogenic embolism. The diagnosis of stroke secondary to large artery

atherosclerosis cannot be made if duplex or arteriographic studies are normal or show only minimal changes.

Cardioembolism

This category includes patients with arterial occlusions presumably due to an embolus arising in the heart (Table 4). Cardiac sources are divided into high-risk and medium-risk groups based on the evidence of their relative propensities for embolism (Table 5). At least one cardiac source for an embolus must be identified for a possible or probable diagnosis of cardioembolic stroke. Clinical and brain imaging findings are similar to those described for large-artery atherosclerosis. Evidence of a previous transient ischemic attack or stroke in more than one vascular territory or systemic embolism supports a clinical diagnosis of cardiogenic stroke. Potential large-artery atherosclerotic sources of thrombosis or embolism should be eliminated. A stroke in a patient with a medium-risk cardiac source of embolism and no other cause of stroke is classified as a possible cardioembolic stroke.

Small artery occlusion (lacunae)

This category includes patients whose strokes are often labeled as lacunar infarcts in other classifications (Table 4). The patient should have one of the traditional clinical lacunar syndromes and should not have evidence of cerebral cortical dysfunction. A history of diabetes mellitus or hypertension supports the clinical diagnosis. The patient should also have a normal CT/MR examination or a relevant brain stem or subcortical hemispheric lesion with a diameter of less than 1.5cm demonstrated. Potential cardiac sources for embolism should be absent, and evaluation of the large extracranial arteries should not demonstrate a stenosis of greater than 50% in an ipsilateral artery.

Acute stroke of other determined etiology

This category includes patients with rare causes of stroke, such as nonatherosclerotic vasculopathies, hypercoagulable states, or hematologic disorders. Patients in this group should have clinical and CT or MR findings of an acute ischemic stroke, regardless of the size or location. Diagnostic studies such as blood tests or arteriography should reveal one of these unusual causes of stroke. Cardiac sources of embolism and large-artery atherosclerosis should be excluded by other studies.

Stroke of undetermined etiology

In several instances, the cause of a stroke cannot be determined with any degree of confidence. Some patients will have no likely etiology determined despite an extensive evaluation. In others, no cause is found but the evaluation was cursory. This category also includes patients with two or more potential causes of stroke so that the physician is unable to make a final diagnosis. For example, a patient with a medium-risk cardiac source of embolism who also has another possible cause of stroke identified would be classified as having a stroke of undetermined etiology. Other examples would be a patient who has atrial fibrillation and an ipsilateral stenosis of 50%, or the patient with a traditional lacunar syndrome and an ipsilateral carotid stenosis of 50%.

Table 3. TOAST Classification of Subtypes of Acute Ischemic Stroke

Large artery atherosclerosis (embolus/thrombosis)*
Cardioembolism (high-risk/medium-risk)*
Small-vessel occlusion (lacunae)*
Stroke of other determined etiology*
Stroke of undetermined etiology
a. Two or more causes identified
b. Negative evaluation
c. Incomplete evaluation

TOAST denotes Trial of Org 10172 in Acute Stroke Treatment.

*Possible or probable depending on results of ancillary studies.

Table 4. Features of TOAST Classification of Subtypes of Ischemic Stroke

	Subtype			
Features	Large artery atherosclerosis	Cardioembolism	Small artery occlusion (lacunae)	Other cause
**Clinical**				
Cortical or	+	+	-	+/-

cerebellar dysfunction				
Lacunar syndrome	-	-	+	+/-
**Imaging**				
Cortical, cerebellar, brain stem, or subcortical infarct > 1.5 cm	+	+	-	+/-
Subcortical or brain stem infarct < 1.5 cm	-	-	+/-	+/-
**Tests**				
Stenosis of extracranial internal carotid artery	+	-	-	-
Cardiac source of emboli	-	+	-	-
Other abnormality on tests	-	-	-	+

Table 5. TOAST Classification of High- and Medium-Risk Sources of Cardioembolism

High-risk sources

Mechanical prosthetic valve

Mitral stenosis with atrial fibrillation

Atrial fibrillation (other than lone atrial fibrillation)

Left atrial/atrial appendage thrombus

Sick sinus syndrome

Recent myocardial infarction (<4 weeks)

Left ventricular thrombus

Dilated cardiomyopathy

Akinetic left ventricular segment

Atrial myxoma

Infective endocarditis

Medium-risk sources

Mitral valve prolapse

Mitral annulus calcification

Mitral stenosis without atrial fibrillation

Left atrial turbulence (smoke)

Atrial septal aneurysm

Patent foramen ovale

Atrial flutter

Lone atrial fibrillation

Bioprosthetic cardiac valve

Nonbacterial thrombotic endocarditis

Congestive heart failure

Hypokinetic left ventricular segment

Myocardial infarction (> 4 weeks, < 6 months)

Appendix 2 - The Alberta Stroke Program Early Computed Tomography Score

Non-contrast CT shall be scored using ASPECTS, a 10-point score derived by examining each of 10 regions on the middle cerebral artery territory[31]. Ischemic change present is scored as 0; ischemic change absent is score as 1. Adding up the score gives a maximum of 10 (favorable scan) and minimum of 0 (unfavorable scan). The score is highly reliable when trichotomized into 0-4 (severe ischemic change, large core), 5-7 (moderate ischemic change) and 8-10 (minimal ischemic change, small core). ASPECTS may be less reliable early in stroke (i.e. within 90 minutes of onset); however, at later time windows it should be easy to recognize large areas of irreversible damage. Having a good quality scan and optimization of scanner is key to successful interpretation. Further information is available at: www.aspectsinstroke.com.

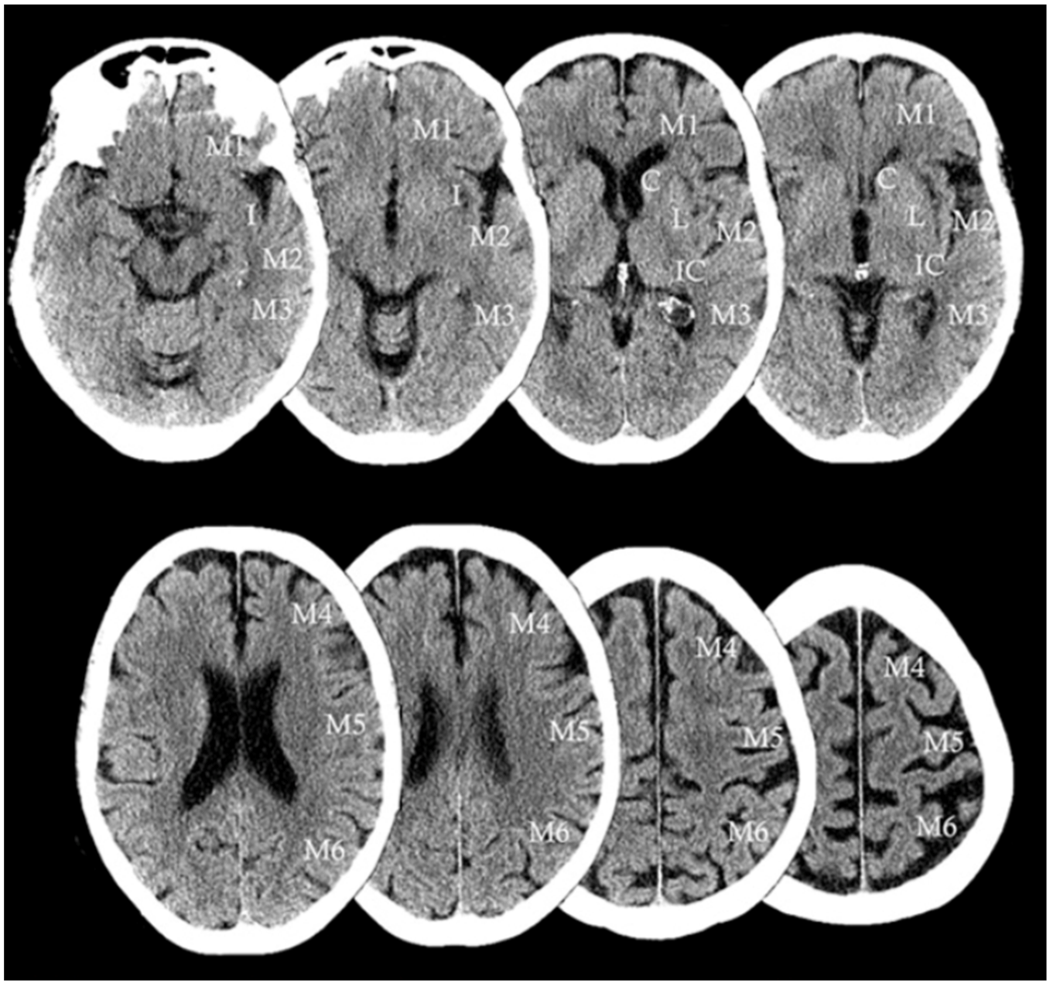

Appendix 3 - Modified Rankin Scale

Grade	Description[32]
0	No symptoms at all
1	No significant disability despite symptoms: able to carry out all usual duties and activities
2	Slight disability: unable to carry out all previous activities but able to look after own affairs without assistance
3	Moderate disability: requiring some help, but able to walk without assistance
4	Moderately severe disability: unable to walk without assistance, and unable to attend to own bodily needs without assistance
5	Severe disability: bedridden, incontinent, and requiring constant nursing care and attention
6	Death

Investigator's Agreement

I have read the attached protocol: **A multicenter, randomized, placebo-controlled, double-blind trial of endovascular treatment with versus without tirofiban for stroke patients with large vessel occlusion (RESCUE BT trial)**, Version 1.0 dated 8th July 2018 and agree to abide by all provisions set forth therein. I agree to comply with the current International Conference on Harmonization Guidelines for Good Clinical Practice and the laws, rules, regulations and guidelines of the community, country, state or locality relating to the conduct of the clinical study. I also agree that persons debarred from conducting or working on clinical studies by any court or regulatory agency will not be allowed to conduct or work on studies for the sponsor.

Name Site Principal Investigator Signature

Name of Clinical Site Date

REFERENCES

1. Global, regional, and national age-sex specific mortality for 264 causes of death, 1980-2016: a systematic analysis for the Global Burden of Disease Study 2016. Lancet (London, England) 2017;390:1151-210.

2. Zhou M, Wang H, Zhu J, et al. Cause-specific mortality for 240 causes in China during 1990-2013: a systematic subnational analysis for the Global Burden of Disease Study 2013. Lancet (London, England) 2016;387:251-72.

3. Wang W, Jiang B, Sun H, et al. Prevalence, Incidence, and Mortality of Stroke in ChinaClinical Perspective. Circulation 2017;135:759-71.

4. Saver JL, Goyal M, Bonafe A, et al. Stent-retriever thrombectomy after intravenous t-PA vs. t-PA alone in stroke. The New England journal of medicine 2015;372:2285-95.

5. Bhatia R, Hill MD, Shobha N, et al. Low rates of acute recanalization with intravenous recombinant tissue plasminogen activator in ischemic stroke: real-world experience and a call for action. Stroke; a journal of cerebral circulation 2010;41:2254-8.

6. Nogueira RG, Jadhav AP, Haussen DC, et al. Thrombectomy 6 to 24 Hours after Stroke with a Mismatch between Deficit and Infarct. The New England journal of medicine 2018;378:11-21.

7. Albers GW, Marks MP, Kemp S, et al. Thrombectomy for Stroke at 6 to 16 Hours with Selection by Perfusion Imaging. The New England journal of medicine 2018;378:708-18.

8. Goyal M, Demchuk AM, Menon BK, et al. Randomized assessment of rapid endovascular treatment of ischemic stroke. The New England journal of medicine 2015;372:1019-30.

9. Campbell BC, Mitchell PJ, Kleinig TJ, et al. Endovascular therapy for ischemic stroke with perfusion-imaging selection. The New England journal of medicine 2015;372:1009-18.

10. Berkhemer OA, Fransen PS, Beumer D, et al. A randomized trial of intraarterial treatment for acute ischemic stroke. The New England journal of medicine 2015;372:11-20.

11. Jovin TG, Chamorro A, Cobo E, et al. Thrombectomy within 8 hours after symptom onset in ischemic stroke. The New England journal of medicine 2015;372:2296-306.

12. Association NSoCM. Chinese guidelines for the endovascular treatment of acute ischemic stroke 2018. Chinese Journal of Neurology 2018;51:683.

13. Powers WJ, Rabinstein AA, Ackerson T, et al. 2018 Guidelines for the Early Management of Patients With Acute Ischemic Stroke: A Guideline for Healthcare Professionals From the American Heart Association/American Stroke Association. Stroke; a journal of cerebral circulation 2018;49.

14. Heo JH, Lee KY, Kim SH, Kim DI. Immediate reocclusion following a successful thrombolysis in acute stroke: a pilot study. Neurology 2003;60:1684-7.

15. Power S, Matouk C, Casaubon LK, et al. Vessel wall magnetic resonance imaging in acute ischemic stroke: effects of embolism and mechanical thrombectomy on the arterial wall. Stroke; a journal of cerebral circulation 2014;45:2330-4.

16. Cura FA, Bhatt DL, Lincoff AM, et al. Pronounced benefit of coronary stenting and adjunctive platelet glycoprotein IIb/IIIa inhibition in complex atherosclerotic lesions. Circulation 2000;102:28-34.

17. Stone GW, Grines CL, Cox DA, et al. Comparison of angioplasty with stenting, with or without abciximab, in acute myocardial infarction. The New England journal of medicine 2002;346:957-66.

18. Steen H, Lehrke S, Wiegand UKH, et al. Very early cardiac magnetic resonance imaging for quantification of myocardial tissue perfusion in patients receiving tirofiban before percutaneous coronary intervention for ST-elevation myocardial infarction. American heart journal 2005;149:564.

19. Siebler M, Hennerici MG, Schneider D, et al. Safety of Tirofiban in acute Ischemic Stroke: the SaTIS trial. Stroke; a journal of cerebral circulation 2011;42:2388-92.

20. Chang Y, Kim BM, Bang OY, et al. Rescue Stenting for Failed Mechanical Thrombectomy in Acute Ischemic Stroke: A Multicenter Experience. Stroke; a journal of cerebral circulation 2018;49:958-64.

21. Zhao W, Che R, Shang S, et al. Low-Dose Tirofiban Improves Functional Outcome in Acute Ischemic Stroke Patients Treated With Endovascular Thrombectomy. Stroke; a journal of cerebral circulation 2017;48:3289-94.

22. Kellert L, Hametner C, Rohde S, et al. Endovascular stroke therapy: tirofiban is associated with risk of fatal intracerebral hemorrhage and poor outcome. Stroke; a journal of cerebral circulation 2013;44:1453-5.

23. Zaidat OO, Yoo AJ, Khatri P, et al. Recommendations on angiographic revascularization grading standards for acute ischemic stroke: a consensus statement. Stroke; a journal of cerebral circulation 2013;44:2650-63.

24. Banks JL, Marotta CA. Outcomes validity and reliability of the modified Rankin scale: implications for stroke clinical trials: a literature review and synthesis. Stroke; a journal of cerebral circulation 2007;38:1091-6.

25. Quinn TJ, Dawson J, Walters MR, Lees KR. Reliability of the modified Rankin Scale: a systematic review. Stroke; a journal of cerebral circulation 2009;40:3393-5.

26. Brott T, Adams HP, Jr., Olinger CP, et al. Measurements of acute cerebral infarction: a clinical examination scale. Stroke; a journal of cerebral circulation 1989;20:864-70.

27. Brooks R. EuroQol: the current state of play. Health Policy 1996;37:53-72.

28. Herdman M, Gudex C, Lloyd A, et al. Development and preliminary testing of the new five-level version of EQ-5D (EQ-5D-5L). Quality of life research : an international journal of quality of life aspects of treatment, care and rehabilitation 2011;20:1727-36.

29. Goyal M, Menon BK, van Zwam WH, et al. Endovascular thrombectomy after large-vessel ischaemic stroke: a meta-analysis of individual patient data from five randomised trials. The Lancet 2016;387:1723-31.

30. Adams HP, Jr., Bendixen BH, Kappelle LJ, et al. Classification of subtype of acute ischemic stroke. Definitions for use in a multicenter clinical trial. TOAST. Trial of Org 10172 in Acute Stroke Treatment. Stroke; a journal of cerebral circulation 1993;24:35-41.

31. Pexman JH, Barber PA, Hill MD, et al. Use of the Alberta Stroke Program Early CT Score (ASPECTS) for assessing CT scans in patients with acute stroke. AJNR American journal of neuroradiology 2001;22:1534-42.

32. Bonita R, Beaglehole R. Recovery of motor function after stroke. Stroke; a journal of cerebral circulation 1988;19:1497-500.

RESCUE BT: A multicenter, randomized, placebo-controlled, double-blind trial of endovascular treatment with versus without tirofiban for stroke patients with large vessel occlusion

TRIAL PROTOCOL

Principle Investigators

Qingwu Yang, MD and Wenjie Zi, MD

Xinqiao Hospital, Army Medical University, Chongqing, China

Prepared by: Zhongming Qiu, MD, Fengli Li, MD, Xinqiao Hospital, Army Medical University, Chongqing, China

Raul G. Nogueira, MD, Department of Neurology, Marcus Stroke & Neuroscience Center, Grady Memorial Hospital, Emory University School of Medicine, Atlanta, GA 30303, USA

Protocol Version: 2.0

Issue Date: 5th August 2020

CONTENTS

List of Abbreviations **54**
Study Synopsis **55**
Schedule of Assessments **60**
1. BACKGROUND INFORMATION **61**
2. TRIAL OBJECTIVES **62**
3. TRIAL DESIGN **63**
4. PATIENT POPULATION **64**
4.1. Inclusion criteria 65
4.2. Exclusion criteria 65
5. BASELINE CLINICAL AND LABORATORY EVALUATIONS **66**
6. PARTICIPATING CENTER ELIGIBILITY **66**
7. RANDOMIZATION **67**
8. BLINDING/UNBLINDING **67**
9. CONTENTS OF INVESTIGATIONAL PRODUCT KIT **68**
9.1. Tirofiban group 68
9.2. Placebo group 68
10. TREATMENTS **69**
11. OUTCOMES **70**
11.1. Primary Efficacy Outcome 70
11.2. Secondary Efficacy Outcomes 71
11.2.1. Secondary Clinical Efficacy Outcomes 71
11.2.2. Secondary Technical Efficacy Outcomes 71
11.3. Safety Outcomes 71
12. ASSESSMENT OF EFFICACY **72**
12.1. The Modified Rankin Scale 72
12.2. The National Institutes of Health Stroke Scale 73
12.3. European Quality Five Dimensions Five Level scale 73
13. ASSESSMENT OF SAFETY **74**
13.1. Symptomatic Intracranial Hemorrhage 74

13.2. Mortality at 90 days 74
13.3. Adverse Event Definitions 74
13.3.1. Adverse Event 74
13.3.2. Serious Adverse Event 74
13.4. Clinical Management of Adverse Events 76
13.4.1. Early Study Drug Cessation 76
13.4.2. Identification of Adverse Events by the Investigator 76
13.4.3. Reporting of Adverse Events 77
13.4.4. Prompt Reporting of Serious Adverse Events 77
14. DATA SAFETY MONITORING BOARD 78
15. IMAGING CORE LABORATORY 78
16. CLINICAL EVENTS COMMITTEE 79
17. STATISTICS 79
17.1. Sample size estimates 79
17.2. Analysis Populations 79
17.2.1. Intention-to-Treat Population 79
17.2.2. Per-Protocol Population 80
17.2.3. As-Treated Population 80
17.2.4. Safety Population 80
17.3. Analysis of Primary Efficacy Outcome 80
17.4. Analysis of Key Secondary Outcome 81
17.5. Analysis of Secondary Outcomes and Safety Outcomes 81
17.6. Adjustment for Covariates and Subgroup Analyses 82
17.7. Handling of Missing Data 82
18. DIRECT ACCESS TO SOURCE DATA/DOCUMENTS 83
19. STUDY MONITORING AND QUALITY ASSURANCE 83
20. PROTOCOL AMENDMENTS 84
21. ETHICAL CONSIDERATION 84
22. DATA HANDLING AND RECORD KEEPING 85
22.1. Data Handling 85
22.2. Data Retention 85
22.3. Case Report Forms 85
22.4. Confidentiality 86

23. USE OF INFORMATION AND PUBLICATION 86
24. FUNDINGS 87
Appendix 1 – Classification of Subtype of Acute Ischemic Stroke 88
Appendix 2 - The Alberta Stroke Program Early Computed Tomography Score 93
Appendix 3 - Modified Rankin Scale 94
Investigator's Agreement 95
REFERENCES 96

List of Abbreviations

AE	Adverse Event
AIS	Acute Ischemia Stroke
ASPECTS	Alberta Stroke Program Early Computed Tomography Score
CRF	Case Report Form
CT	Computed Tomography
DSMB	Data Safety Monitoring Board
EQ-5D-5L	European Quality Five-Dimension Five-Level
eTICI	expanded Thrombolysis in Cerebral Infarction
EVT	Endovascular Treatment
GCP	Good Clinical Practice
ICH-GCP	International Conference on Harmonization-Good Clinical Practice
IRB	Institutional Review Board
ITT	Intention-to-Treat
LVO	Large Vessel Occlusion
MR	Magnetic Resonance
mRS	modified Rankin Scale
NIHSS	National Institutes of Health Stroke Scale
PI	Principal Investigator
REB	Research Ethics Board
RESCUE BT	The Endovascular Treatment With versus Without Tirofiban for Stroke Patients with Large Vessel Occlusion Trial
SAE	Serious Adverse Event
SICH	Symptomatic Intracerebral Hemorrhage
TOAST	Trial of Org 10172 in Acute Stroke Treatment

1101 **Study Synopsis**

**Study Title**	RESCUE BT: A multicenter, randomized, placebo-controlled, double-blind trial of endovascular treatment with versus without tirofiban for stroke patients with large vessel occlusion
**Study Phase**	Phase 3
**Indication**	Acute Ischemic Stroke
**Study drug**	Active drug: intravenous tirofiban (10μg/kg bolus and then 0.15μg/kg/min maintenance for up to 24 hours)<br>Placebo: saline placebo which is indistinguishable from the active drug
**No. Subjects**	Approximately 930 (465 subjects per arm)
**No. Centers**	Approximately 50 stroke centers in China
**Study Duration**	Total study duration is 36~48 months. Patients will participate in the trial for 12 months
**Recruitment Period**	30~42 months
**Trial Objectives**	To test the hypothesis that acute ischemic stroke patients with large vessel occlusion who receive endovascular treatment within 24 hours of symptom onset will have improved clinical outcomes when given intravenous tirofiban therapy compared to placebo.
**Trial Design**	This will be an investigator-initiated, multicenter, randomized, double-blind, placebo-controlled trial.
**Randomization**	Eligible patients will be consecutively randomized to tirofiban or placebo group with a ratio of 1:1. Randomization is stratified according to stroke severity (National Institutes of Health Stroke Scale (NIHSS) score ≤17 vs. > 17), occlusion site (the intracranial internal carotid artery or not), and participating center.
**Inclusion Criteria**	(8) Aged 18 years or older;<br>(9) Presenting with acute ischemic stroke with symptoms within 24

	hours of time last known well (those who have not received intravenous thrombolysis within 4.5 hours due to contraindications, refusal of intravenous thrombolysis, and other various reasons may also be candidates);<br>(10) Baseline NIHSS score ≤ 30;<br>(11) Baseline Alberta Stroke Program Early CT Score (ASPECTS) ≥ 6;<br>(12) Occlusion of the intracranial internal carotid artery, the first or second segment of the middle cerebral artery confirmed by CT, MR angiography, or digital subtraction angiography;<br>(13) Planned treatment with endovascular treatment by clinical care team;<br>(14) Informed consent obtained from patients or their legal representatives.
**Exclusion Criteria**	(15) CT or MR evidence of hemorrhage;<br>(16) Dual antiplatelet therapy within 1 week of the index stroke;<br>(17) Treated with intravenous thrombolysis after the index stroke;<br>(18) Currently pregnant or lactating (women patients);<br>(19) Allergy to tirofiban, radiographic contrast agents, or nitinol devices;<br>(20) Gastrointestinal or urinary tract hemorrhage within 30 days of the index stroke;<br>(21) Any major surgery within 14 days of the index stroke;<br>(22) History of heparin-induced thrombocytopenia;<br>(23) Any terminal illness with life expectancy less than 6 months;<br>(24) Preexisting neurological or psychiatric disease that would confound the neurological functional evaluations;<br>(25) Platelet count of routine blood test < 100 * 10^9/L;<br>(26) Severe renal insufficiency (glomerular filtration rate < 30ml/min

	or serum creatinine > 220μmol/L (2.5mg/dl));<br>(27) Arterial tortuosity and/or other arterial disease that would prevent the device from reaching the target vessel;<br>(28) Unlikely to be available for 90-day follow-up.
**Treatments**	Tirofiban 10μg/kg bolus and then 0.15μg/kg/min maintenance for up to 24 hours (or matching normal saline placebo volume) will be administered intravenous infusion in the upper or lower extremity using an infusion pump starting after randomization.<br>All subjects will undergo attempted endovascular treatment and receive best medical care according to modern acute stroke care guidelines.
**Consent**	Explicit written, signed informed consent from the subject or legally authorized representative will be obtained prior to any protocol specific procedures.
**Duration of Treatment**	This study consists of one 90-day study period for each subject.<br>Subjects will be hospitalized for care after their acute stroke according to the current standard of care. Subjects are required to return to clinic on Day 90 for end-of-study procedures.
**Laboratory Tests**	In order to support the assessment of patient safety baseline, chemistry laboratory tests will be completed. At baseline, blood work will be evaluated which includes: blood cell counts, triglyceride, cholesterol, low density lipoprotein, high density lipoprotein, homocysteine, glucose, procalcitonin, HbA1C, prothrombin time, activated partial thromboplastin time, thrombin time, fibrinogen, D-dimer, international normalized ratio.<br>If the subject is female and is of childbearing potential, a pregnancy test (urine or serum point-of-care pregnancy test) must be completed and a negative test result obtained prior to inclusion in the trial.<br>Electrocardiograms will also be collected and reviewed at baseline.

**Assessment of Efficacy**	The **primary efficacy outcome** is assessed on the modified Rankin Scale (mRS) score at 90 days from randomization. The mRS, a global measure of disability, comprises of seven grades ranging from 0 (no symptoms) to 6 (death).<br>The **secondary clinical efficacy outcomes** include:<br>7) Proportion of patients non-disabled (mRS score 0 to 1) at 90 days or return to pre-morbid mRS score at 90 days (for patients with mRS > 1);<br>8) Proportion of mRS score 0 to 2 at 90 days;<br>9) Proportion of mRS score 0 to 3 at 90 days;<br>10) Change of the NIHSS score at 24 hours from baseline;<br>11) Change of the NIHSS score at 5-7 days or discharge if earlier from baseline;<br>12) European Quality Five Dimensions Five Level scale score at 90 days.<br>The **secondary technical efficacy outcomes** include:<br>5) Substantial reperfusion before endovascular treatment, as assessed on initial digital subtraction angiography. Substantial reperfusion is defined as expanded Thrombolysis in Cerebral Infarction score of 2b (substantial perfusion), 2c (near-complete perfusion) or 3 (complete reperfusion);<br>6) Substantial reperfusion at final angiogram;<br>7) Proportion of Rescue Drug utilization;<br>8) Recanalization at 48 hours evaluated by CT or MR angiography, assessed with the Arterial Occlusive Lesion scale.
**Assessment of Safety**	1) Symptomatic intracerebral hemorrhage rate within 48 hours;<br>2) Proportion of patients with any intracranial hemorrhage within 48 hours;<br>3) Mortality at 90 days;

	4) Procedure-related complications such as arterial perforation, iatrogenic arterial dissection, arterial access site hematoma, retroperitoneal hematoma, etc;<br>5) Incidence of serious adverse events.

Schedule of Assessments

	Baseline	Day 1~2	Day 5~7	Day 90
Eligibility criteria	X			
Demographics	X			
Medical history	X			
Prior medication	X			
Laboratory results	X			
Electrocardiography	X			
Clinical examination	X	X	X	
Weight[1]	X			
Informed consent	X			
Randomization	X			
mRS score	X			X
NIHSS score	X	X	X	
ASPECTS	X			
Brain CT+CTA or MRI+MRA	X	X		
DSA	X			
Concomitant medication		X	X	X
Adverse event		X	X	X
EQ-5D-5L				X

1.The subject's actual weight will be measured in hospital using standard hospital scales (i.e., stand up or in-bed scales if the subject is not ambulatory). If actual weight cannot be measured for any reason (due to, for example severe illness or unavailability of in-bed scales at the site), weight will be determined by first asking the subject, second asking a family member or third by estimation.

1. BACKGROUND INFORMATION

The burden of stroke

Stroke is the leading cause of death and the most frequent cause of permanent disability in China[1,2]. The National Epidemiological Survey of Stroke in China (NESS-China) shown that the age-standardized stroke prevalence is 1,115 cases per 100,000 people, annual age-standardized incidence 247 cases per 100,000, and mortality 115 cases per 100,000[3].
Acute ischemic stroke (AIS) accounts for approximate 75% of all strokes, and intracranial large vessel occlusion (LVO) is a common cause of AIS, accounting for about 20%. According to this incidence and percentage, there would be approximate 518,700 LVO patients per year in China. Remarkably, LVO stroke often leads to severe disability and high mortality when comparing non-LVO stroke.

Endovascular treatment in large vessel occlusive stroke

In 1996, alteplase was approved for the treatment of AIS. However, it has several major therapeutic limitations, one of which is the low recanalization rate in LVO stroke[4,5]. To overcome this limitation, endovascular treatments (EVT) have been gradually developed over the last 20 years. Since 2015, at least 7 high-quality multicenter randomized controlled trials shown that for AIS patients caused by anterior LVO, EVT combined with usual medical care can significantly improve clinical outcomes compared with usual medical care alone[4,6-11]. Both the American Heart Association/American Stroke Association and the Chinese stroke early management guidelines strongly recommend EVT as the first line treatment for AIS due to anterior circulation LVO[12,13]. EVT include mechanical thrombectomy using stent-retriever or aspiration devices, angioplasty and/or stenting, intraarterial thrombolysis, and clot disruption using a guidewire or microcatheter (thromborrhexis), all of which may cause traumatic damage of the vascular endothelial with subendothelial matrix exposure, leading to platelet activation, adhesion, and aggregation, and potentially resulting in re-occlusion and thromboembolic complications[14,15].

The platelet glycoprotein IIb/IIIa receptor inhibitor, Tirofiban

The binding of fibrinogen or von Willebrand factor to the platelet glycoprotein IIb/IIIa receptor is the final common pathway for platelet aggregation and subsequent formation of thrombi, which can be reversibly inhibits by tirofiban, a non-peptide selective platelet

glycoprotein IIb/IIIa receptor inhibitor. Tirofiban is the most widely used glycoprotein IIb/IIIa receptor inhibitor due to its pharmacological characteristics including rapid onset of action, short half-life, high selectivity and affinity, and reversible inhibition of fibrinogen-dependent platelet aggregation. It has been proven that the combined use of tirofiban, especially when administered early, can reduce the risk of vascular complications and the need for revascularization during percutaneous coronary intervention[16]. Based on the positive experience and findings in ischemic heart disease patients who receiving percutaneous coronary intervention[17,18], many investigators have evaluated the safety and efficacy of tirofiban as an adjunctive therapy in LVO stroke patients undergoing EVT.

Several studies have suggested that tirofiban may be beneficial to patients with AIS. A randomized, placebo-controlled, open-label treatment, multicenter trial recruited 260 stroke patients who had a National Institutes of Health Stroke Scale (NIHSS) score between 4 and 18 and indicated that tirofiban might decrease mortality in the long-term follow up without increasing the incidence of intracranial hemorrhage (ICH)[19]. However, this study included LVO and non-LVO strokes and predated the era of modern endovascular stroke therapy. A multicenter retrospective study involving 148 LVO stroke patients undergoing rescue stenting who failed in mechanical thrombectomy indicated that adjuvant tirofiban is correlated with stent patency without inducing more symptomatic intracranial hemorrhage (SICH)[20]. An observational study including 180 LVO stroke patients (90 patients in each group with and without tirofiban) suggested that the clinical outcomes of the tirofiban group was better than that of the non-tirofiban group, and the incidence of SICH did not increase significantly[21]. Conversely, several studies have suggested that tirofiban might actually increase the risk of fatal ICH and unfavorable clinical outcomes[22,23]. However, most of the available data is comprised by small, single-center, retrospective studies. There is currently no randomized controlled trial evaluating the effect of tirofiban in EVT of AIS.

2. TRIAL OBJECTIVES

Primary objective

The primary objective is to determine the efficacy of the non-peptide selective platelet glycoprotein IIb/IIIa receptor inhibitor, tirofiban, in reducing severity of disability in patients

with AIS with LVO and within 24 hours of last known well selected for EVT.

Secondary objective

The secondary objectives are to determine the efficacy of tirofiban in:

- Increasing proportion of patients non-disabled (modified Rankin Scale (mRS) score 0 to 1) or return to pre-morbid mRS score (for patients with mRS > 1)
- Improving functional independence (mRS score 0 to 2)
- Reducing re-occlusion

Leading Safety Objectives

The leading safety objectives are to determine the effect of tirofiban to patient with LVO stroke who are selected for EVT on SICH, and 90-day mortality.

3. TRIAL DESIGN

The Endovascular Treatment With versus Without Tirofiban for Stroke Patients with Large Vessel Occlusion (RESCUE BT) Trial is an investigator-initiated, multicenter, prospective, randomized, placebo-controlled, double-blind clinical trial, aiming to specifically assess the efficacy and safety of intravenous tirofiban in anterior circulation LVO stroke patients who undergo EVT within 24 hours of symptom onset. The trial has been registered at Chinese Clinical Trial Registry (www.chictr.org.cn, unique identifier ChiCTR-INR-17014167). The study patient flow outline was shown in Figure 1.

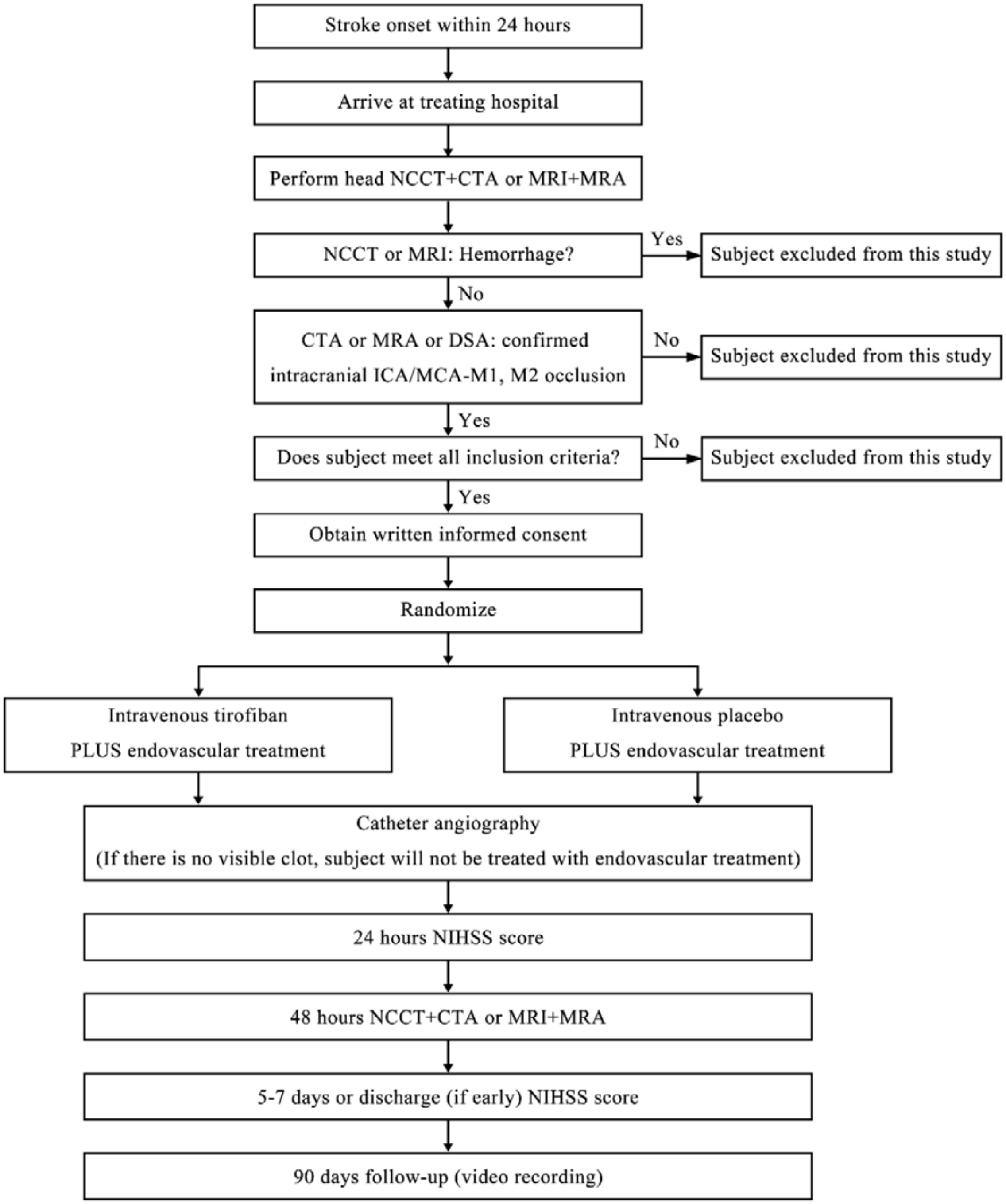

Figure 1 Study flowchart of RESCUE BT trial.

4. PATIENT POPULATION

All subjects will undergo a neurological and clinical assessment, routine laboratory blood test and baseline brain imaging. All these materials will be collected and screened by site physician. This includes standard of care use of non-contrast CT/ CT angiography or diffusion

weighted imaging/MR angiography. In order to track the potential for enrollment, each enrolling site will provide the total number of patients admitted to that site with the diagnosis of AIS-LVO. However, screening log is not required. If the patient is still eligible after completing the routine screening, the patient will be agreed (as needed) and included in the study. Patients who are randomized but do not receive study drug will still be followed up to the 90-day study period.

4.1. Inclusion criteria

(1) Aged 18 years or greater;
(2) Presenting with AIS symptom within 24 hours of time last known well (those who have not received intravenous thrombolysis within 4.5 hours due to contraindications, refusal of intravenous thrombolysis, and other various reasons may also be candidates);
(3) Baseline NIHSS score ≤ 30;
(4) Baseline Alberta Stroke Program Early Computed Tomography Score (ASPECTS) ≥ 6;
(5) Occlusion of the intracranial internal carotid artery, the first or second segment of the middle cerebral artery confirmed by CT, MR angiography, or digital subtraction angiography;
(6) Planned treatment with EVT by clinical care team;
(7) Informed consent obtained from patients or their legal representatives.

4.2. Exclusion criteria

(15) CT or MR evidence of hemorrhage;
(16) Dual antiplatelet therapy within 1 week of the index stroke;
(17) Treated with intravenous thrombolysis after the index stroke;
(18) Currently in pregnant or lactating on admission;
(19) Contraindication to radiographic contrast agents, nickel, titanium metals or their alloys;
(20) Gastrointestinal or urinary tract hemorrhage within 30 days of the index stroke;
(21) Any major surgery within 14 days of the index stroke;
(22) History of heparin-induced thrombocytopenia;
(23) Any terminal illness with life expectancy less than 6 months;
(24) Patients with a preexisting neurological or psychiatric disease that would confound the neurological functional evaluations;

(25) Platelet count of routine blood test < 100 * 10^9/L;

(26) Severe renal insufficiency (glomerular filtration rate < 30ml/min or serum creatinine > 220μmol/L (2.5mg/dl));

(27) Arterial tortuosity and/or other arterial disease that would prevent the device from reaching the target vessel;

(28) Unlikely to be available for 90-day follow-up;

Patients can leave the study at any time upon request. Patients who refuse to participate in the study will be treated as usual. Patients who choose to withdraw from the study should attend the final withdrawal visit. The withdrawal date and reason should be documented in the Case Report Form (CRF), and Subject Withdrawal Form.

5. BASELINE CLINICAL AND LABORATORY EVALUATIONS

At Baseline, all subjects will undergo a routine neurological and clinical assessment (including history, examination and vital signs), and the NIHSS.

Emergency blood work will be drawn including: blood cell counts, triglyceride, cholesterol, low density lipoprotein, high density lipoprotein, homocysteine, glucose, procalcitonin, HbA1C, prothrombin time, activated partial thromboplastin time, thrombin time, fibrinogen, D-dimer, international normalized ratio. The study will use and collect local laboratory results and will not use a central lab. The results of this blood work are not required prior to randomization.

If the subject is female and is of childbearing potential a pregnancy test (urine point-of-care pregnancy test) must be completed and a negative test result obtained prior to inclusion in the trial. Other laboratory or point-of-care testing may be performed at the discretion of the attending physicians and team.

An electrocardiogram will also be collected and reviewed at baseline.

6. PARTICIPATING CENTER ELIGIBILITY

To be fully eligible for participation in this trial, study centers were required have performed at least 80 endovascular procedures annually, including at least 50 thrombectomy procedures

with the stent-retriever devices or contact aspiration devices. Moreover, all neuro-interventionists with more than five years' experience in cerebrovascular intervention and at least 10 cases of mechanical thrombectomy annually.

7. RANDOMIZATION

Eligible patients will be randomly assigned to tirofiban or placebo group with a ratio of 1:1. Randomization will be carried out immediately via a web-based App on mobile phone or computer (www.ratjin.com) after the patient's eligibility status has been confirmed. Randomization is stratified by stroke severity (NIHSS ≤ 17 vs. > 17), occlusion site (the intracranial internal carotid artery or not), and participating center. The stratified randomization approach helps avoid potential imbalances between the two treatment groups that may affect the clinical outcomes and biased results. Randomization will be completely concealed by having both web-based real-time allocation and identical appearance of tirofiban and placebo bottles. All bottles will have a unique number. Subjects will be assigned a random serial number according to the time they were enrolled, and corresponding masked medications will be provided.

8. BLINDING/UNBLINDING

All trial personnel (investigators, their clinical staff, and the data management group) and patients will be blinded to treatment assignment. The Data Safety Monitoring Board (DSMB) will have access to grouped data, but not unblinded in treatment assignment.

Both the person responsible for investigational product labels and the independent statistical group that prepare reports for the DSMB will be unblinded. In addition, the person in charge of the data management group responsible for managing the programming of the randomized system will be unblinded. If it is necessary to unblind, this person will become the contact person. This person will not participate in data management and will only communicate the unblinded data listed below when contacted by the medical monitor.

If the treatment code needs to be cracked for the safety of the patient, the site principal investigator (PI) will call the medical monitor for all unblinding queries. The medical monitor will discuss with the researcher whether it is necessary to unblind the patient. If the medical

monitor determines that the PI should be unblinded for the patient, the medical monitor will contact with the person in charge of the data management group by e-mail or phone, instructing the unblinding of the specific patient. Members of the unblinding data management team will provide only to the site PI with allocation information, the unblinding date, site number, PI name, and patient number via email. Any cases unblinded in this way will be recorded in the central file. Only the physician who requests the unblinding will receive the unblinding message. Study drugs will be stopped afterwards. It is not expected that there will be any clinical cases that need to be unblinded. The randomization data will be strictly confidential, and only authorized personnel can access it until the data is locked at the end of the study.

9. CONTENTS OF INVESTIGATIONAL PRODUCT KIT

Tirofiban and its placebo (saline) are manufactured and provided by Lunan Pharmaceutical Group Co., Ltd., Linyi, China. Aspirin and Clopidogrel are produced by Bayer HealthCare Manufacturing S.r.l. and Sanofi Winthrop Industrie respectively, and purchased and provided by Lunan Pharmaceutical Group Co., Ltd., Linyi, China. All study medication will be manufactured, tested, released, packaged, labelled, and shipped in accordance with Good Manufacturing Practice, Good Clinical Practice (GCP) guidelines, and any national regulatory requirements.

Tirofiban and its placebo (saline) are prepared in numbered and are visually identical (such as labeling, dosage form, size, and color), except for a unique number. Tirofiban and saline placebo will be packed in sterile, disposable, individually labeled bottles. Each kit has a unique six-digit identification number and will be stored in a safe location at room temperature on the clinical site with restricted access.

9.1. Tirofiban group

The box of tirofiban group contains tirofiban hydrochloride injection (12.5mg/250ml*2 bottles, the bottles are marked as Study Drug) and saline placebo (250ml*2 bottles, Rescue Drug).

9.2. Placebo group

The box of control group contains saline placebo (250ml*2 bottles, Study Drug) and tirofiban hydrochloride injection (12.5mg/250ml*2 bottles, Rescue Drug).

10. TREATMENTS

Eligible patients will be randomly assigned a number corresponding to a blinded sealed medication kit that is given to each patient. It is recommended that the usage of Study Drugs be started within 5 minutes after randomization.

Patients in the tirofiban group and the placebo group will receive intravenous bolus followed by continuous infusion of tirofiban and saline placebo (Study Drug: 10μg/kg bolus and then 0.15μg/kg/min maintenance for up to 24 hours), respectively. Endovascular treatment should be initiated as soon as possible. The treatment technique choice is at the treating neuro-interventionalists' discretion. EVT consisted of mechanical thrombectomy, thromboaspiration, balloon dilation, stenting, intra-arterial thrombolysis, clot disruption using a guidewire or microcatheter (thromborrhexis), or various combinations of these approaches. However, the intra-arterial infusion of thrombolytics (such as alteplase or urokinase), Study Drug, Rescue Drug, or other glycoprotein IIb/IIIa inhibitors is not recommended and will be recorded in the concomitant medications list of CRF. After recanalization of the target artery, all patients will get stroke unit care and postoperative management.

We acknowledge that patients undergoing mechanical thrombectomy might occasionally require rescue treatment with balloon angioplasty and stenting. If the antegrade blood flow cannot be maintained after angioplasty and/or stenting, Rescue Drug can be used. The Rescue Drug is available as a second medication bottle in the study kits and it used in the same manner as the Study Drug. Rescue Drug and Study Drug are injected intravenously through two different infusion pipelines at the same time.

At the 20th hour after using the Study Drug, the two treatment groups will receive oral antiplatelet therapy with aspirin and/or clopidogrel tablets. Patients undergoing angioplasty/stenting will be given dual-antiplatelet therapy with aspirin and clopidogrel. In other cases, mono-antiplatelet therapy is given. If the patient takes aspirin before the index stroke, he will continue to use aspirin, while those who use clopidogrel will use clopidogrel. At the 24th hour, the Study Drug will be discontinued. After that, all patients will be managed

according to the current American Heart Association/American Stroke Association guidelines[13]. Risk factors such as hypertension, atrial fibrillation, diabetes mellitus, hyperlipidemia, decreased physical activity, and smoking should be treated appropriately. The treatment scheme is showed in Figure 2.

Intravenous heparin may be used during the thrombectomy procedure at the discretion of the operator. In addition, the post-procedural use of subcutaneous heparin or low-molecular-weight heparin for deep vein thrombosis prophylaxis is permitted. However, the use of any other (intravenous or oral) anticoagulants or antiplatelet agents is not allowed during the first 24 hours post randomization. Concomitant medications, both within one week before enrollment and during the whole course of the study, will be documented.

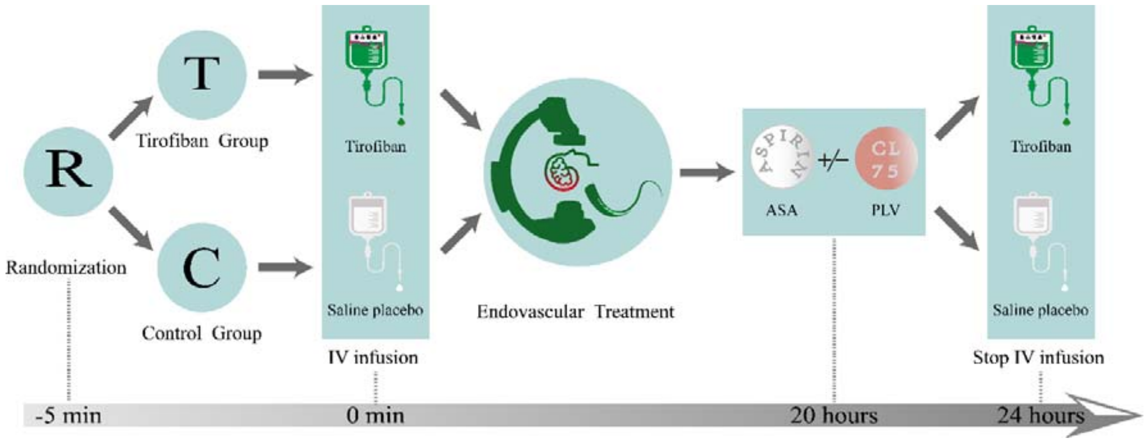

Figure 2 The treatment flow chart of the RESCUE BT trial

11. OUTCOMES

11.1. Primary Efficacy Outcome

The primary end-point is the distribution of global disability on the mRS score at 90 days after randomization. To ensure the reliability, evaluability, and traceability of the mRS score, we will keep patients' video or voice version of follow-up at 90 days except those who refuse to take a video. The mRS scores will be centrally assessed by two independent certified neurologists in a blinded manner by the use of the video or voice recording. Disagreements are resolved by consensus. For those who decline to participate in a video or voice recording, the outcomes will be determined in person by site neurologists blinded to the treatment allocation.

11.2. Secondary Efficacy Outcomes

11.2.1. Secondary Clinical Efficacy Outcomes

(7) Proportion of patients non-disabled (mRS score 0 to 1) or return to pre-morbid mRS score at 90 days (for patients with mRS > 1);

(8) Proportion of patients with functional independence at 90 days (mRS score 0 to 2);

(9) Proportion of patients ambulatory or bodily needs-capable or better (mRS score 0 to 3);

(10) Change of the NIHSS score at 24 hours from baseline;

(11) Change of the NIHSS score at 5-7 days or discharge if earlier from baseline;

(12) Health-related quality of life, assessed with the European Quality Five Dimensions Five Level scale (EQ-5D-5L) at 90 days.

11.2.2. Secondary Technical Efficacy Outcomes

(5) Proportion of substantial reperfusion before endovascular treatment, as assessed on initial digital subtraction angiography. Substantial reperfusion is defined as expanded Thrombolysis in Cerebral Infarction score of 2b (substantial perfusion), 2c (near-complete perfusion) or 3 (complete reperfusion)[24];

(6) Proportion of substantial reperfusion at final angiogram;

(7) Proportion of Rescue Drug utilization;

(8) Proportion of vessel recanalization at 48 hours evaluated by CT or MR angiography, assessed with the Arterial Occlusive Lesion scale;

11.3. Safety Outcomes

(6) SICH rate within 48 hours. ICH will be evaluated according to the Heidelberg Bleeding Classification. SICH is diagnosed if the new observed ICH is associated with any of the following conditions: 1) NIHSS score increased more than 4 points than that immediately before worsening; 2) NIHSS score increased more than 2 points in one category; 3) Deterioration led to intubation, hemicraniectomy, external ventricular drain placement or any other major interventions. Additionally, the symptom deteriorations could not be explained by causes other than the observed ICH. Hemicraniectomy will be defined as that surgical procedure used to decompress the swollen hemisphere;

(7) Proportion of patients with any ICH within 48 hours;

(8) Mortality at 90 days. Mortality rates are defined as the number of deaths observed divided by the number of subjects observed over the 90-day study period;

(9) Procedure-related complications such as arterial perforation, iatrogenic arterial dissection, embolization in previously uninvolved vascular territory, arterial access site hematoma, and retroperitoneal hematoma. Arterial perforation will be defined at angiography by the operator and associated with subarachnoid hemorrhage. Iatrogenic arterial dissection will be defined at angiography by the operator. Arterial access site hematoma will be assessed as a complication of arterial access puncture and defined by clinical examination and anatomic imaging. Retroperitoneal hematoma will be assessed as a complication of groin puncture and defined by imaging (ultrasound or CT or MR angiography). The definition of embolization in previously uninvolved vascular territory is noted after recanalization of the primary occlusion site, any vessel occlusions distal from the primary occlusion site are considered emboli due to periprocedural thrombus fragmentation.

(10) Incidence of serious adverse events.

12. ASSESSMENT OF EFFICACY

12.1. The Modified Rankin Scale

The mRS is a valid and reliable clinician-reported measure of global disability that has been widely applied for evaluating recovery from stroke. It is a scale used to measure functional recovery (the degree of disability or dependence in daily activities) of people who have suffered a stroke[25,26]. mRS scores range from 0 to 6, with 0 indicating no residual symptoms; 5 indicating bedbound, requiring constant care; and 6 indicating death. The mRS score will be obtained at Day 90. Premorbid mRS status will also be obtained retrospectively and reported on the 24 Hours CRF page. The mRS will only be scored by those who have been trained and certified to use this scale using the table below.

Table 1. Level of Function Survey - mRS-9Q

Q 1: Do you have any symptoms that are bothering you?	□Yes □No
Q 2: Are you able to do the same work as before?	□Yes □No
Q 3: Are you able to keep up with your hobbies?	□Yes □No

Q 4: Have you maintained your ties to friends and family?	□Yes □No
Q 5: Do you need help making a simple meal, doing household chores, or balancing a checkbook?	□Yes □No
Q 6: Do you need help with shopping or traveling close to home?	□Yes □No
Q 7: Do you need another person to help you walk?	□Yes □No
Q 8: Do you need help with eating, going to the toilet, or bathing?	□Yes □No
Q 9: Do you stay in bed most of the day and need constant nursing care?	□Yes □No

mRS-9Q: the mRS calculator (http://www.modifiedrankin.com/)

12.2. The National Institutes of Health Stroke Scale

The NIHSS is a standardized neurological examination score that is a valid and reliable measure of disability and recovery after acute stroke[27]. Scores range from 0 to 42, with higher scores indicating more severe disability. The scale includes measures of level of consciousness, extra ocular movements, motor and sensory tests, coordination, language and speech evaluations. The NIHSS will be administered at baseline, at 24 hours from baseline, and Day 5-7or discharge. The NIHSS will only be scored by those trained and certified in the use of this scale. In this trial, we will be kept video version of NIHSS score except those who die or refuse to take a video. The NIHSS score will be centrally assessed by two independent certified neurologists in a blinded manner via the video. Disagreements are resolved by consensus. For those who decline to participate in a video recording, the outcomes will be determined in person by site neurologists blinded to the treatment assignment.

12.3. European Quality Five Dimensions Five Level scale

The EQ-5D-5L is a generic instrument for describing and valuing health. It is based on a descriptive system that defines health in terms of five dimensions: Mobility, Self-Care, Usual Activities, Pain/Discomfort, and Anxiety/Depression[28]. Each dimension has five response categories corresponding to: no problems, slight, moderate, severe and extreme problems[29]. The instrument is designed for self-completion, and respondents also rate their overall health on the day of the interview on a 0-100 hash-marked, vertical visual analogue scale. The EQ-5D-5L will be administered on Day 90 by those trained in the use of this scale.

13. ASSESSMENT OF SAFETY

13.1. Symptomatic Intracranial Hemorrhage

In addition to the efficacy endpoints, we will investigate SICH being the most feared complication of intravenous tirofiban for LVO stroke. SICH as defined in Heidelberg Bleeding Classification. SICH is diagnosed if the new observed ICH is associated with any of the following conditions: 1) NIHSS score increased more than 4 points than that immediately before worsening; 2) NIHSS score increased more than 2 points in one category; 3) Deterioration led to intubation, hemicraniectomy, external ventricular drain placement or any other major interventions. Additionally, the symptom deteriorations could not be explained by causes other than the observed ICH.

13.2. Mortality at 90 days

In addition to SICH, mortality at 90 days is also one of the most important safety endpoints of the RESCUE BT trial. Mortality rates are defined as the number of deaths observed divided by the number of subjects observed over the 90-day study period.

The Highly Effective Reperfusion evaluated in Multiple Endovascular Stroke Trial collaboration, a pooled analysis of patient-level data, shown that stroke mortality rate after EVT is 15.3%[30]. For LVO stroke patients treated between 6 hours and 24 hours mortality is 19%[6].

13.3. Adverse Event Definitions

13.3.1. Adverse Event

Any adverse change in health or the appearance of or worsening of any undesirable sign, symptom or medical condition occurring after enrollment into the trial will be recorded as Adverse Event (AE) whether or not it is considered to be related to the study drug. An AE also includes a new illness; aggravated in severity of frequency from the baseline condition, abnormal results of diagnostic procedures, or a combination of the above. Pre-existing medical conditions are not to be reported as AEs. AEs will be coded using the Medical Dictionary for Regulatory Activities coding dictionary and grouped by system organ class.

13.3.2. Serious Adverse Event

A serious adverse event (SAE) is any untoward medical occurrence that at any dose: Result in death; Are life-threatening or fatal; Require or prolong hospitalization; Result in persistent or significant disability/incapacity, Constitutes a congenital anomaly or birth defect, or; Significant medical event.

A SAE can also be an important medical event that may not result in death, be life-threatening, or require hospitalization, but may jeopardize the subject and may require medical or surgical intervention to prevent one of the outcomes listed in this definition. For example, any new diagnosis of cancer (made after study enrollment) is considered an important medical event. Because our primary safety outcomes for the trial are also SAEs by definition, they will be reported dually as SAEs and as outcomes. SAEs should be managed according to the best current standard of care.

All deaths occurring during the follow up to Day 90 will be reported as an SAE. When reporting a death, the event or condition that caused or contributed to the fatal outcome should be reported as a single medical concept.

AE occurring within 30 days of randomization and all SAEs will be reported in the CRF. The frequencies and incidences of SAEs occurring in subjects in the tirofiban and placebo groups will be summarized within treatment group by the Medical Dictionary for Regulatory Activities and grouped by system organ class.

Table 2. Severity and relationship definitions of Adverse Event.

**AE Severity**	
Mild	Awareness of sign or symptom but easily tolerated
Moderate	Discomfort sufficient to cause interference with normal activities.
Severe	Incapacitating, with inability to perform normal activities.
**AE Relationship**	
Related	A clinical event, including laboratory test abnormality, where there is a “reasonable possibility” that the SAE was caused by the study drug, meaning that there is evidence or arguments to suggest a causal relationship.

Probably	A clinical event, including laboratory test abnormality, with a reasonable time sequence to drug administration, unlikely to be attributed to concurrent disease or other drugs or chemicals, and which follows a clinically reasonable response on withdrawal.
Possibly	A clinical event, including laboratory test abnormality, with a reasonable time sequence to drug administration, but which could also be explained by concurrent disease or other drugs or chemicals. Information on drug withdrawal may be lacking or unclear.
Unrelated	This category is applicable to AEs which are judged to be clearly and incontrovertibly due to extraneous causes (diseases, environment, etc.) and do not meet the criteria for drug relationship listed for the above-mentioned conditions.

13.4. Clinical Management of Adverse Events

13.4.1. Early Study Drug Cessation

The intervention is the intravenous administration of Study Drug over 24 hours to subject undergoing EVT. If any SAE is observed during dosing, dosing shall be immediately terminated. If any moderate or severe AE is observed, the physician may terminate drug administration at his/her discretion.

13.4.2. Identification of Adverse Events by the Investigator

AE monitoring and reporting will be followed-up until Day 30. SAEs will be followed through the final study exit visit (Day 90 Visit or death or end of study whichever is sooner) or until the subject is deemed "lost to follow-up".

AE identification while the subject is admitted to the acute stroke hospital will be collected via acute stroke hospital patient records and verbal histories from the subject or legally authorized representative. For follow up visits after discharge from the acute stroke hospital the subject (or legally authorized representative if the subject is not able to respond to the questions) will be asked about the occurrence of AEs since the last contact, and if available, from records at the acute stroke hospital. AEs that were ongoing at the last contact will be

updated with a stop date or confirmed as ongoing. AE collection will continue until Day 30, and SAE to Day 90 or the final contact.

A consistent methodology of eliciting AEs at all subject evaluation timepoints will be used. Non-directive questions include: How have you felt since your last clinical visit/hospital discharge? Have you had any new or changed health problems since you were last here? Have you had any unusual or unexpected worsening of your underlying medical condition or overall health? Have there been any changes in the medicines you take since your last clinical visit/hospital discharge?

Diagnosis versus signs and symptoms for the purpose of AE reporting: if known at the time of reporting, a diagnosis should be reported rather than individual signs and symptoms. However, if a constellation of signs and/or symptoms cannot be medically characterized as a single diagnosis it is acceptable to report the information that is ultimately available.

13.4.3. Reporting of Adverse Events

AEs should be reported as they occur on the CRF. Documentation must be supported by an entry in the subject's file. Each event should be described in detail along with start and stop dates, severity, relationship to investigational product as judged by the investigator, action taken, and outcome.

13.4.4. Prompt Reporting of Serious Adverse Events

Serious Adverse Events require immediate action.

Once an investigator becomes aware that an SAE has occurred, he/she will immediately notify the clinical coordinator via telephone within one working day. The study SAE form must be completed as thoroughly as possible with all available details of the event, signed by the investigator (or appropriately qualified designee), and faxed to the study manager within one working day of first becoming aware of the event. The equivalent SAE page should be filled in on the CRF.

If the investigator does not have all information regarding an SAE, he/she will not wait to receive additional information before notifying the study monitor of the event and completing the form. The form will be updated when additional information is received.

The investigator will always provide an assessment of causality at the time of the initial report as described previously. If data obtained after reporting indicates that the assessment of

causality is incorrect, then the SAE form may be appropriately amended, signed and dated, and resubmitted.

In accordance with local Ethics Committee requirements, the investigator must also notify their Ethics Committee of any SAEs according the guidelines of the Ethics Committee. The investigator and others responsible for subject care should institute any supplementary investigations of SAEs based on their clinical judgement of the likely causative factors. This may include seeking further opinion from a specialist in the field of the adverse event or requesting extra tests. If a subject dies, any post-mortem findings, including histopathology will be provided if available. No medical help, diagnosis, or advice should be withheld from the subject due to an inability to contact the study manager/medical monitor.

14. DATA SAFETY MONITORING BOARD

The independent DSMB will be composed of an experienced neurologist, an interventionalist, and a biostatistician, which are neither involved in the trial nor affiliated with the sponsor. The DSMB will meet at least once a year, and is provided with structured unmasked reports, prepared by the trial statistician, for their reference only. DSMB is responsible for recommendations to the executive committee regarding stopping or extending the trial. In addition, the DSMB will review the occurrence of SAEs and make recommendations to the executive committee regarding safety of the trial. Interim safety analysis will be conducted when approximate 465 patients have completed their Day 90 assessments. If there are concerns about the safety of patients, this board will make a recommendation to the trial steering committee about continuing, stopping, or modifying the trial. No formal interim efficacy or futility analyses are planned.

15. IMAGING CORE LABORATORY

Centralized imaging core laboratories will be used in this trial to provide consistent assessment of all the images. CT/MR and angiographic images will be independently reviewed by two independent central imaging core laboratories respectively. CT/MR core laboratory will review CT/MR images obtained at baseline and within 24 hours for confirmation of inclusion criteria, ASPECTS score, collateral circulation classification, and

presence/absence of hemorrhage. Angiographic core laboratory will review angiographic images from the procedure to determine clot location, collateral compensation, and recanalization. CT/MR core laboratory will be independent from the angiographic core laboratory to ensure the CT/MR core laboratory is blinded to the treatment allocation.

16. CLINICAL EVENTS COMMITTEE

The Clinical Events Committee will be comprised of three expert physicians independent of the investigational sites. This committee will validate all the complications that occur over the course of the study and categorized for severity and relatedness according to the definition in the AE section. The Clinical Events Committee can request any additional source information and images supporting the AEs to assist with the adjudication.

17. STATISTICS

17.1. Sample size estimates

The distribution of 90-day mRS scores in the placebo group of this trial is derived from the Highly Effective Reperfusion evaluated in Multiple Endovascular Stroke collaboration's intervention population who are ineligible for alteplase as following: mRS 0: 10.2%, mRS 1: 15.7%, mRS 2: 17.6%, mRS 3: 18.5%, mRS 4: 7.4%, mRS 5: 7.4%, and mRS 6: 23.1%[30]. It is assumed that a favorable treatment effect with a common odds ratio of 1.50 would be achieved in the tirofiban group compared with the placebo group, corresponding to 8.5% absolute increase in the proportion of 90-day mRS score 0 to 1. A total sample size of 930 (465 patients per arm) patients would provide 90% power at a 2-sided significance level of 0.05, taking 15% attrition rate into account. This estimation is performed based on PASS (NCSS, LLC. Kaysville, Utah, USA) version 15.0.

17.2. Analysis Populations

17.2.1. Intention-to-Treat Population

The primary efficacy analysis will be conducted in the intention-to-treat (ITT) population, defined as all subjects randomized into the trial with grouping by randomized treatment, regardless of treatment actually received. An ITT analysis will also be conducted for the secondary endpoints, with subject grouped according to the randomized (intended) treatment.

Ineligible patients and patients who receive neither Study Drug therapy nor endovascular treatment should be excluded from the ITT population.

17.2.2. Per-Protocol Population

The primary analysis will be repeated on the per-protocol population, defined to be all subjects randomized and treated, with no major protocol deviations. This population will be determined via blinded review of protocol deviations at the end of the trial before database lock and unblinding. Prior to unblinding, the imaging from each subject at the time of inclusion will be adjudicated to determine whether they have met the criteria for endovascular intervention, and hence for the trial. This will include review of baseline non-contrast CT and CT angiography. Subjects who do not meet the imaging criteria outlined in the trial inclusion/exclusion criteria, will not be included in the per-protocol population.

The following situations are major protocol deviations: (1) Rescue Drug is used, (2) no EVT procedure is conducted, and (3) procedure-related hemorrhage (e.g., artery perforation) resulting in premature termination of Study Drug and EVT and failure of substantial reperfusion.

17.2.3. As-Treated Population

The same as the ITT population except subjects are analyzed based upon actual treatment received. Subjects who receive both Study Drug and Rescue Drug are included in the tirofiban arm, and subjects who receive neither Study Drug nor Rescue Drug are included in the placebo arm.

17.2.4. Safety Population

The safety population will consist of all patients who received any dose of Study Drug. The main analyses will be frequency of SICH and 90-day mortality. It is expected that the safety population and the ITT population will be near-identical. Full details will be specified in detail in the Statistical Analysis Plan.

Patients who withdraw informed consent immediately after randomization and are not to receive any treatment should be excluded from all analysis populations.

17.3. Analysis of Primary Efficacy Outcome

The primary efficacy outcome is the differences in 90-day mRS outcome across the full mRS scale between tirofiban and placebo groups and will be analyzed using ordinal logistic

regression unless the proportional odds assumption is violated, in which case the assumption-free Wilcoxon-Mann-Whitney generalized odds ratio approach will be used. The proportional odds assumption will be tested using a Brant test.

17.4. Analysis of Key Secondary Outcome

For the key secondary outcome analysis, the proportions of 90-day mRS 0-1 or return to pre-morbid mRS score (for patients with mRS > 1) outcomes will be compared between tirofiban and placebo arms using binary logistic regression model. Both adjusted and unadjusted odds ratio their 95% confidence intervals will be reported. Between-group differences will be tested using a chi-square test.

The fixed sequential order for testing in the ITT population is:

1) Primary efficacy outcome

2) Key secondary outcome analysis

3) Secondary and safety endpoints, as specified in the order presented below

17.5. Analysis of Secondary Outcomes and Safety Outcomes

Other secondary outcomes analyses will be carried out in the following order:

(15) Proportion of patients functionally independent (mRS score 0 to 2) at 90 days;

(16) Proportion of patients ambulatory or bodily needs-capable or better (mRS score 0 to 3);

(17) Change of the NIHSS score at 24 hours from baseline;

(18) Change of the NIHSS score at 5-7 days or discharge if earlier from baseline;

(19) Health-related quality of life, assessed with the EQ-5D-5L at 90 days.

(20) Substantial reperfusion before endovascular treatment, as assessed on initial digital subtraction angiography. Substantial reperfusion is defined as an expanded Thrombolysis in Cerebral Infarction score of 2b (substantial perfusion), 2c (near-complete perfusion) or 3 (complete reperfusion)[24];

(21) Substantial reperfusion at final angiogram;

(22) Proportion of Rescue Drug utilization;

(23) Recanalization at 48 hours evaluated by CT or MR angiography, assessed with the Arterial Occlusive Lesion scale;

(24) SICH rate within 48 hours. SICH will be evaluated according to the Heidelberg Bleeding Classification;

(25) Proportion of patients with any ICH within 48 hours;

(26) Mortality at 90 days;

(27) Procedure-related complications including arterial perforation, vessel dissection, embolization into a new vascular territory, and puncture access complications;

(28) Incidence of serious adverse events.

17.6. Adjustment for Covariates and Subgroup Analyses

In addition to the primary and secondary analyses adjusting for age, baseline NIHSS score, baseline ASPECTS score, occlusion site, and onset-to-randomization time, exploratory analyses will be conducted to determine the potential roles of common baseline characteristics and assess potential heterogeneity of treatment effect across subgroups. Specific subgroups of interest include the age > median vs. ≤ median years old, sex male vs. female, baseline NIHSS score > median vs. ≤ median, baseline ASPECTS > median vs. ≤ median, occlusion location (intracranial internal carotid artery occlusion: no vs. yes), stroke etiology (large artery atherosclerosis: no vs. yes), rescue therapy (no vs. yes), onset to randomization time > median vs. ≤ median, and other variables of interest. Full details will be specified in detail in the Statistical Analysis Plan.

17.7. Handling of Missing Data

Every effort will be made to keep missing data, particularly the Day 90 outcome assessments, to a minimum. Regular reminders of patient follow-up due dates will be provided to participating centers to facilitate scheduling of follow-up visits. Nevertheless, some missing data may be inevitable due to, for example, loss to follow-up. Since all randomized patients will be included in the primary endpoint analysis (ITT), every patient must have a 90-day mRS score. Patients who die within the study period will be assigned the worst score on all outcome measures and taken into the analysis.

Missing outcome data of mRS scores at 90 days in the lead analysis will be imputed using multiple imputation. Complete case and worst-case analyses will be performed as sensitivity analyses. In the worst-case analysis, if the patient was identified to be alive, we will assign a score of 5. Proportions of missing values for all variables will be reported. Variables that will be used to adjust the primary and secondary effect analyses (age, NIHSS score and ASPECTS at baseline, time to randomization, and site of occlusion) are designated as key variables.

Missing values for these variables (if any) will be analyzed for randomness and imputed with standard methods.

18. DIRECT ACCESS TO SOURCE DATA/DOCUMENTS

The sponsor or delegate will be permitted to visit the study facilities at any reasonable time in order to maintain current, detailed knowledge of the study through review of the records, source documents, observation, and discussion of the conduct and progress of the study. In addition, the sponsor will maintain regular telephone and written communication with all investigators through the coordinating center. The sponsor (or delegate) will be given complete access to all components of the study facility that pertain to the conduct of this study, and may be present to observe any aspect of the conduct of the study by medical and paramedical staff, including but not limited to drug preparations, dosing, sample collections, and clinical observations. CRFs will be monitored with sufficient frequency to assess the following: Subject randomization, compliance with protocol procedures, the completeness and accuracy of data entered into the CRFs, verification of CRF data against original source documents, and occurrence of AEs. Adequate time and all documents for these monitoring visits must be made available by the investigators. The investigators will permit trial-related monitoring, audits, Research Ethics Board/Institutional Review Board (REB/IRB) review, and regulatory inspections, providing direct access to source data/documents.

19. STUDY MONITORING AND QUALITY ASSURANCE

The investigators promise to conduct the RESCUE BT trial in accordance with this trial protocol, International Conference on Harmonization-GCP (ICH-GCP) Guidelines and applicable regulatory requirements.

The investigators must ensure compliance with all procedures required by the trial protocol and all research procedures (including safety rules) provided by the sponsor. The investigators agree to provide reliable data and all information required by the trial protocol in an accurate and legible manner according to the instructions provided, and to ensure direct access to the source documents by the sponsor's representative.

The sponsor of this clinical trial is responsible to the health authorities and takes all reasonable measures to ensure the correct implementation of the clinical trial protocol in terms of ethics, clinical trial protocol compliance, and the completeness and validity of the data recorded on the CRF.

The main responsibility of the monitoring team is to help researchers and sponsors to ensure that all aspects of clinical trials are highly ethical, scientific, professional and standardized. According to the ICH-GCP guidelines, the monitoring team must check CRF entries based on source documents, unless predetermined.

The monitoring team will regularly contact the centers through field visits, emails or phone calls, and send inspectors to assess the progress of the research, the compliance of investigators and patients with the research protocol, and to resolve urgent issues. During these inspection visits, the inspector will work with the on-site investigator. The main aspects of inspection and monitoring are as follows (not exclusive): patient informed consent, patient recruitment and follow-up, serious adverse event recording and reporting, study drug supply, treatment compliance of study drug group participants, study drug count, concomitant treatment and data quality.

20. PROTOCOL AMENDMENTS

The Protocol will not be modified by the Investigator without first obtaining the agreement of the other in writing. During the trial, any amendment or modification to the protocol should be submitted to the REB/IRB for approval prior to implementation. It should also be informed of any event likely to affect the safety of patients or the continued conduct of the trial, in particular any change in safety. If requested, a progress report will be sent to the REB/IRB annually and a summary of the trial's outcome at the end of the trial.

21. ETHICAL CONSIDERATION

This research followed the ethical principles of the Helsinki Declaration. This protocol and the consent forms will be submitted to each hospital’s REB/IRB. Before initiation of the study, a copy of the REB/IRBs’ approval letters will be provided to the sponsor and the membership list of the REB/IRB will be kept on file. To make sure the subjects fully understand about this

trial, the investigators must provide the patients or their legal representatives with detailed information about the clinical trial, including the purpose of the trial, possible benefits and risks, and the rights/obligations. Subjects have the right to withdraw from the study at any time if they wish to do so. The privacy protection of subjects has to be ensured. The patients or their legal representatives give their written informed consent prior to the study. Each patient must leave contact information to the investigator of the participating center. At the same time, the investigator must leave his own phone number to the patient so that the patient can find the investigator at any time. Ethical approval for the study was obtained by the Ethics Committee of the participating centers. SAEs will be reported to the REB/IRB according to their requirements.

22. DATA HANDLING AND RECORD KEEPING

22.1. Data Handling

During the trial, clinical data reported in the CRFs will be integrated into the clinical database under the responsibility of the Sponsor or their qualified representative. Quality control in the form of computerized logic and/or consistency checks will be systematically applied in order to detect errors or omissions. In addition, safety reviews may be performed several times by the Sponsor's staff in the course of the trial. Any questions pertaining to the reported clinical data will be submitted to the investigator for resolution. Each step of this process will be monitored through the implementation of individual passwords to maintain appropriate database access and to ensure database integrity.

After integration of all corrections in the complete set of data, the database will be released for statistical analysis.

22.2. Data Retention

The double reviewed CRF and imaging data will be sent to the data management group. The person in charge of the data management group will check and sign the receipt form. The CRF will be kept by the research center after data entry is completed.

22.3. Case Report Forms

For each subject randomized, an CRF must be completed and signed by the investigator. If a subject withdraws from the study, the reason must be noted on the CRF. All forms should be

completed within five business days of subject visit. All corrections will be tracked in the CRF audit trail. The investigator should ensure the accuracy, completeness, legibility, and timeliness of the data reported to the sponsor in the CRFs and in all required reports.

22.4. Confidentiality

All study investigators at the clinical sites must ensure that the confidentiality of personal identity and all personal medical information of study subjects are maintained at all times. Researchers who use information about the health of their research participants are required, except in specific circumstances, to get written permission to use their participant's Protected Health Information for the research study. Each participating clinical center is expected to comply with its individual performance site's requirements established for compliance of the local confidentiality policies.

23. USE OF INFORMATION AND PUBLICATION

All information concerning the RESCUE BT trial supplied to the investigators by the Steering Committee and not previously published is considered confidential and shall remain the sole property of the RESCUE BT Steering Committee. The investigator agrees to use this information only in accomplishing the study and will not use it or the data generated from the study for other purposes without first obtaining the written authorization from the RESCUE BT Steering Committee.

It is understood that the RESCUE BT Steering Committee may disclose this information as required to other RESCUE BT clinical investigators or to government regulatory agencies. The investigator understands that she or he has the obligation to provide complete test results and all data collected during this study to the Steering Committee.

A writing committee will be formed to review and publish the data from the study. This committee will consist of the Steering Committee and a subset of investigators. The writing committee will write/review all drafts of abstracts and full-length manuscripts and will choose the appropriate journal (for manuscripts) or meeting (for abstracts) for submission.

The RESCUE BT Steering Committee commits that when the study is completed, the data from this study will be published, regardless of the outcome of the study and the trial will be listed on the Chinese Clinical Trial Registry website.

24. FUNDINGS

RESCUE BT trial is an investigator-initiated study which is organized by the second affiliated hospital of the Third Military Medical University and conducted in about 50 comprehensive stroke centers in China. The authors disclosed receipt of the following financial support: (1) Lunan Pharmaceutical Group Co., Ltd., China, (2) National Natural Science Foundation of China (No. 82090040, 81901236, 82071323, and 81801157), and (3) Army Medical University Clinical Medical Research Talent Training Program (No. 2019XLC2008 and 2019XLC3016). The funders had no involvement in the study design, data collection, analysis and interpretation, writing or decision to submit the paper.

Appendix 1 – Classification of Subtype of Acute Ischemic Stroke

The TOAST classification system includes five categories: 1) large-artery atherosclerosis, 2) cardioembolism, 3) small-artery occlusion (lacunae), 4) stroke of other determined etiology, and 5) stroke of undetermined etiology (Table 3)[31]. Diagnoses are based on clinical features and on data collected by tests such as brain imaging (CT/MR), cardiac imaging (echocardiography, etc.), duplex imaging of extracranial arteries, arteriography, and laboratory assessments for a pro-thrombotic state.

The physician can apply the clinical and imaging findings when first assessing the patient and then consider the results of other diagnostic tests later. An important part of the classification is the ability of the physician to categorize a specific subtype diagnosis as probable or possible based on the degree of certainty. A "probable" diagnosis is made if the clinical findings, neuroimaging data, and results of diagnostic studies are consistent with one subtype and other etiologies have been excluded. A "possible" diagnosis is made when the clinical findings and neuroimaging data suggest a specific subtype but other studies are not done. Because many patients will have a limited number of diagnostic tests, the probable and possible subcategorizations allow the physician to make as precise a subgroup diagnosis as can be achieved.

Large artery atherosclerosis

These patients will have clinical and brain imaging findings of either significant (>50%) stenosis or occlusion of a major brain artery or branch cortical artery, presumably due to atherosclerosis (Table 4). Clinical findings include those of cerebral cortical impairment (aphasia, neglect, restricted motor involvement, etc.) or brain stem or cerebellar dysfunction. A history of intermittent claudication, transient ischemic attacks in the same vascular territory, a carotid bruit, or diminished pulses helps support the clinical diagnosis. Cortical or cerebellar lesions and brain stem or subcortical hemispheric infarcts greater than 1.5 cm in diameter on CT or MR are considered to be of potential large-artery atherosclerotic origin. Supportive evidence by duplex imaging or arteriography of a stenosis of greater than 50% of an appropriate intracranial or extracranial artery is needed. Diagnostic studies should exclude potential sources of cardiogenic embolism. The diagnosis of stroke secondary to large artery

atherosclerosis cannot be made if duplex or arteriographic studies are normal or show only minimal changes.

Cardioembolism

This category includes patients with arterial occlusions presumably due to an embolus arising in the heart (Table 4). Cardiac sources are divided into high-risk and medium-risk groups based on the evidence of their relative propensities for embolism (Table 5). At least one cardiac source for an embolus must be identified for a possible or probable diagnosis of cardioembolic stroke. Clinical and brain imaging findings are similar to those described for large-artery atherosclerosis. Evidence of a previous transient ischemic attack or stroke in more than one vascular territory or systemic embolism supports a clinical diagnosis of cardiogenic stroke. Potential large-artery atherosclerotic sources of thrombosis or embolism should be eliminated. A stroke in a patient with a medium-risk cardiac source of embolism and no other cause of stroke is classified as a possible cardioembolic stroke.

Small artery occlusion (lacunae)

This category includes patients whose strokes are often labeled as lacunar infarcts in other classifications (Table 4). The patient should have one of the traditional clinical lacunar syndromes and should not have evidence of cerebral cortical dysfunction. A history of diabetes mellitus or hypertension supports the clinical diagnosis. The patient should also have a normal CT/MR examination or a relevant brain stem or subcortical hemispheric lesion with a diameter of less than 1.5cm demonstrated. Potential cardiac sources for embolism should be absent, and evaluation of the large extracranial arteries should not demonstrate a stenosis of greater than 50% in an ipsilateral artery.

Acute stroke of other determined etiology

This category includes patients with rare causes of stroke, such as nonatherosclerotic vasculopathies, hypercoagulable states, or hematologic disorders. Patients in this group should have clinical and CT or MR findings of an acute ischemic stroke, regardless of the size or location. Diagnostic studies such as blood tests or arteriography should reveal one of these unusual causes of stroke. Cardiac sources of embolism and large-artery atherosclerosis should be excluded by other studies.

Stroke of undetermined etiology

In several instances, the cause of a stroke cannot be determined with any degree of confidence. Some patients will have no likely etiology determined despite an extensive evaluation. In others, no cause is found but the evaluation was cursory. This category also includes patients with two or more potential causes of stroke so that the physician is unable to make a final diagnosis. For example, a patient with a medium-risk cardiac source of embolism who also has another possible cause of stroke identified would be classified as having a stroke of undetermined etiology. Other examples would be a patient who has atrial fibrillation and an ipsilateral stenosis of 50%, or the patient with a traditional lacunar syndrome and an ipsilateral carotid stenosis of 50%.

Table 3. TOAST Classification of Subtypes of Acute Ischemic Stroke

Large artery atherosclerosis (embolus/thrombosis)*
Cardioembolism (high-risk/medium-risk)*
Small-vessel occlusion (lacunae)*
Stroke of other determined etiology*
Stroke of undetermined etiology
a. Two or more causes identified
b. Negative evaluation
c. Incomplete evaluation

TOAST denotes Trial of Org 10172 in Acute Stroke Treatment.

*Possible or probable depending on results of ancillary studies.

Table 4. Features of TOAST Classification of Subtypes of Ischemic Stroke

	Subtype			
Features	Large artery atherosclerosis	Cardioembolism	Small artery occlusion (lacunae)	Other cause
**Clinical**				
Cortical or	+	+	-	+/-

cerebellar dysfunction				
Lacunar syndrome	-	-	+	+/-
**Imaging**				
Cortical, cerebellar, brain stem, or subcortical infarct > 1.5 cm	+	+	-	+/-
Subcortical or brain stem infarct < 1.5 cm	-	-	+/-	+/-
**Tests**				
Stenosis of extracranial internal carotid artery	+	-	-	-
Cardiac source of emboli	-	+	-	-
Other abnormality on tests	-	-	-	+

Table 5. TOAST Classification of High- and Medium-Risk Sources of Cardioembolism

High-risk sources

- Mechanical prosthetic valve
- Mitral stenosis with atrial fibrillation
- Atrial fibrillation (other than lone atrial fibrillation)
- Left atrial/atrial appendage thrombus
- Sick sinus syndrome
- Recent myocardial infarction (<4 weeks)
- Left ventricular thrombus
- Dilated cardiomyopathy
- Akinetic left ventricular segment
- Atrial myxoma
- Infective endocarditis

Medium-risk sources

- Mitral valve prolapse
- Mitral annulus calcification
- Mitral stenosis without atrial fibrillation
- Left atrial turbulence (smoke)
- Atrial septal aneurysm
- Patent foramen ovale
- Atrial flutter
- Lone atrial fibrillation
- Bioprosthetic cardiac valve
- Nonbacterial thrombotic endocarditis
- Congestive heart failure
- Hypokinetic left ventricular segment
- Myocardial infarction (> 4 weeks, < 6 months)

Appendix 2 - The Alberta Stroke Program Early Computed Tomography Score

Non-contrast CT shall be scored using ASPECTS, a 10-point score derived by examining each of 10 regions on the middle cerebral artery territory[32]. Ischemic change present is scored as 0; ischemic change absent is score as 1. Adding up the score gives a maximum of 10 (favorable scan) and minimum of 0 (unfavorable scan). The score is highly reliable when trichotomized into 0-4 (severe ischemic change, large core), 5-7 (moderate ischemic change) and 8-10 (minimal ischemic change, small core). ASPECTS may be less reliable early in stroke (i.e. within 90 minutes of onset); however, at later time windows it should be easy to recognize large areas of irreversible damage. Having a good quality scan and optimization of scanner is key to successful interpretation. Further information is available at: ww.aspectsinstroke.com.

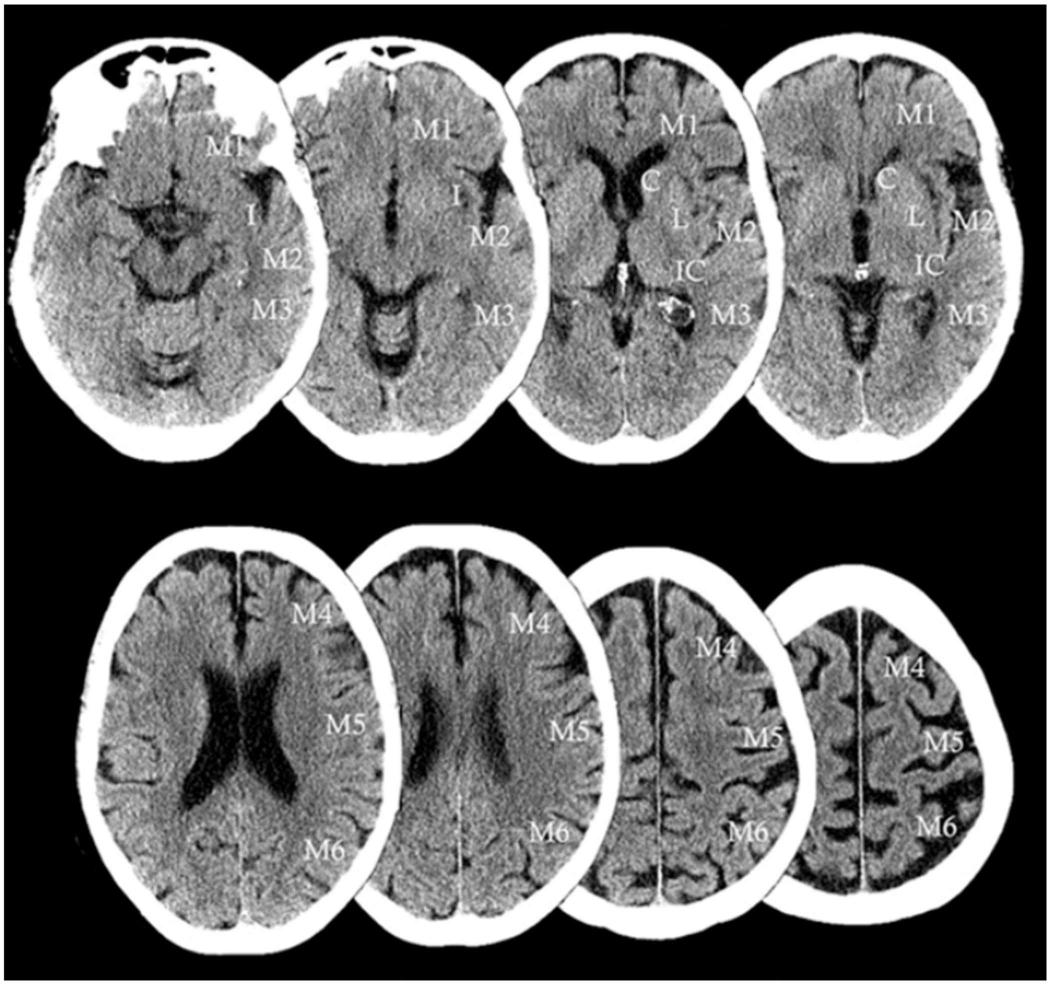

Appendix 3 - Modified Rankin Scale

Grade	Description[33]
0	No symptoms at all
1	No significant disability despite symptoms: able to carry out all usual duties and activities
2	Slight disability: unable to carry out all previous activities but able to look after own affairs without assistance
3	Moderate disability: requiring some help, but able to walk without assistance
4	Moderately severe disability: unable to walk without assistance, and unable to attend to own bodily needs without assistance
5	Severe disability: bedridden, incontinent, and requiring constant nursing care and attention
6	Death

Investigator's Agreement

I have read the attached protocol: **A multicenter, randomized, placebo-controlled, double-blind trial of endovascular treatment with versus without tirofiban for stroke patients with large vessel occlusion (RESCUE BT trial)**, Version 2.0 dated 5th August 2020 and agree to abide by all provisions set forth therein. I agree to comply with the current International Conference on Harmonization Guidelines for Good Clinical Practice and the laws, rules, regulations and guidelines of the community, country, state or locality relating to the conduct of the clinical study. I also agree that persons debarred from conducting or working on clinical studies by any court or regulatory agency will not be allowed to conduct or work on studies for the sponsor.

Name Site Principal Investigator Signature

Name of Clinical Site Date

REFERENCES

1. Global, regional, and national age-sex specific mortality for 264 causes of death, 1980-2016: a systematic analysis for the Global Burden of Disease Study 2016. Lancet (London, England) 2017;390:1151-210.
2. Zhou M, Wang H, Zhu J, et al. Cause-specific mortality for 240 causes in China during 1990-2013: a systematic subnational analysis for the Global Burden of Disease Study 2013. Lancet (London, England) 2016;387:251-72.
3. Wang W, Jiang B, Sun H, et al. Prevalence, Incidence, and Mortality of Stroke in ChinaClinical Perspective. Circulation 2017;135:759-71.
4. Saver JL, Goyal M, Bonafe A, et al. Stent-retriever thrombectomy after intravenous t-PA vs. t-PA alone in stroke. The New England journal of medicine 2015;372:2285-95.
5. Bhatia R, Hill MD, Shobha N, et al. Low rates of acute recanalization with intravenous recombinant tissue plasminogen activator in ischemic stroke: real-world experience and a call for action. Stroke; a journal of cerebral circulation 2010;41:2254-8.
6. Nogueira RG, Jadhav AP, Haussen DC, et al. Thrombectomy 6 to 24 Hours after Stroke with a Mismatch between Deficit and Infarct. The New England journal of medicine 2018;378:11-21.
7. Albers GW, Marks MP, Kemp S, et al. Thrombectomy for Stroke at 6 to 16 Hours with Selection by Perfusion Imaging. The New England journal of medicine 2018;378:708-18.
8. Goyal M, Demchuk AM, Menon BK, et al. Randomized assessment of rapid endovascular treatment of ischemic stroke. The New England journal of medicine 2015;372:1019-30.
9. Campbell BC, Mitchell PJ, Kleinig TJ, et al. Endovascular therapy for ischemic stroke with perfusion-imaging selection. The New England journal of medicine 2015;372:1009-18.
10. Berkhemer OA, Fransen PS, Beumer D, et al. A randomized trial of intraarterial treatment for acute ischemic stroke. The New England journal of medicine 2015;372:11-20.
11. Jovin TG, Chamorro A, Cobo E, et al. Thrombectomy within 8 hours after symptom onset in ischemic stroke. The New England journal of medicine 2015;372:2296-306.
12. Association NSoCM. Chinese guidelines for the endovascular treatment of acute ischemic stroke 2018. Chinese Journal of Neurology 2018;51:683.
13. Powers WJ, Rabinstein AA, Ackerson T, et al. 2018 Guidelines for the Early Management of Patients With Acute Ischemic Stroke: A Guideline for Healthcare Professionals From the American Heart Association/American Stroke Association. Stroke; a journal of cerebral circulation 2018;49.
14. Heo JH, Lee KY, Kim SH, Kim DI. Immediate reocclusion following a successful thrombolysis in acute stroke: a pilot study. Neurology 2003;60:1684-7.
15. Power S, Matouk C, Casaubon LK, et al. Vessel wall magnetic resonance imaging in acute ischemic stroke: effects of embolism and mechanical thrombectomy on the arterial wall. Stroke; a journal of cerebral circulation 2014;45:2330-4.
16. Cura FA, Bhatt DL, Lincoff AM, et al. Pronounced benefit of coronary stenting and adjunctive platelet glycoprotein IIb/IIIa inhibition in complex atherosclerotic lesions. Circulation 2000;102:28-34.
17. Stone GW, Grines CL, Cox DA, et al. Comparison of angioplasty with stenting, with or without abciximab, in acute myocardial infarction. The New England journal of medicine 2002;346:957-66.
18. Steen H, Lehrke S, Wiegand UKH, et al. Very early cardiac magnetic resonance imaging for quantification of myocardial tissue perfusion in patients receiving tirofiban before percutaneous coronary intervention for ST-elevation myocardial infarction. American heart journal 2005;149:564.
19. Siebler M, Hennerici MG, Schneider D, et al. Safety of Tirofiban in acute Ischemic Stroke: the SaTIS trial. Stroke; a journal of cerebral circulation 2011;42:2388-92.

20. Chang Y, Kim BM, Bang OY, et al. Rescue Stenting for Failed Mechanical Thrombectomy in Acute Ischemic Stroke: A Multicenter Experience. Stroke; a journal of cerebral circulation 2018;49:958-64.

21. Zhao W, Che R, Shang S, et al. Low-Dose Tirofiban Improves Functional Outcome in Acute Ischemic Stroke Patients Treated With Endovascular Thrombectomy. Stroke; a journal of cerebral circulation 2017;48:3289-94.

22. Wu Y, Yin C, Yang J, Jiang L, Parsons MW, Lin L. Endovascular Thrombectomy. Stroke; a journal of cerebral circulation 2018;49:2783-5.

23. Kellert L, Hametner C, Rohde S, et al. Endovascular stroke therapy: tirofiban is associated with risk of fatal intracerebral hemorrhage and poor outcome. Stroke; a journal of cerebral circulation 2013;44:1453-5.

24. Liebeskind DS, Bracard S, Guillemin F, et al. eTICI reperfusion: defining success in endovascular stroke therapy. Journal of neurointerventional surgery 2019;11:433-8.

25. Banks JL, Marotta CA. Outcomes validity and reliability of the modified Rankin scale: implications for stroke clinical trials: a literature review and synthesis. Stroke; a journal of cerebral circulation 2007;38:1091-6.

26. Quinn TJ, Dawson J, Walters MR, Lees KR. Reliability of the modified Rankin Scale: a systematic review. Stroke; a journal of cerebral circulation 2009;40:3393-5.

27. Brott T, Adams HP, Jr., Olinger CP, et al. Measurements of acute cerebral infarction: a clinical examination scale. Stroke; a journal of cerebral circulation 1989;20:864-70.

28. Brooks R. EuroQol: the current state of play. Health Policy 1996;37:53-72.

29. Herdman M, Gudex C, Lloyd A, et al. Development and preliminary testing of the new five-level version of EQ-5D (EQ-5D-5L). Quality of life research : an international journal of quality of life aspects of treatment, care and rehabilitation 2011;20:1727-36.

30. Goyal M, Menon BK, van Zwam WH, et al. Endovascular thrombectomy after large-vessel ischaemic stroke: a meta-analysis of individual patient data from five randomised trials. The Lancet 2016;387:1723-31.

31. Adams HP, Jr., Bendixen BH, Kappelle LJ, et al. Classification of subtype of acute ischemic stroke. Definitions for use in a multicenter clinical trial. TOAST. Trial of Org 10172 in Acute Stroke Treatment. Stroke; a journal of cerebral circulation 1993;24:35-41.

32. Pexman JH, Barber PA, Hill MD, et al. Use of the Alberta Stroke Program Early CT Score (ASPECTS) for assessing CT scans in patients with acute stroke. AJNR American journal of neuroradiology 2001;22:1534-42.

33. Bonita R, Beaglehole R. Recovery of motor function after stroke. Stroke; a journal of cerebral circulation 1988;19:1497-500.

Summary of Changes - Protocol RESCUE BT Version 1.0 to Version 2.0

Below is the table of changes. Deleted items are identified with Strikethrough font. Additional wording is in bold font

Section(s)	Protocol Version 1.0<br>Change From:	Protocol Version 2.0<br>Change To:	Rationale
**List of Abbreviations**	mTICI - modified Treatment in Cerebral Infarction	eTICI - expanded Thrombolysis in Cerebral Infarction	Addition<br>To be consistent with the Change of Secondary Efficacy Outcomes
**Study Synopsis - Inclusion Criteria**<br><br>**Section 4.1. Inclusion criteria**	(2) Acute ischemic stroke occurs between 4.5 and 12 hours of time last known well;	(2) Presenting with acute ischemic stroke with symptoms within 24 hours of time last known well (those who have not received intravenous thrombolysis within 4.5 hours due to contraindications, refusal of intravenous thrombolysis, and other various reasons may also be candidates);	(1) Recent randomized controlled trials have shown that the time window for mechanical thrombectomy can be extended to within 24 hours of stroke onset<br><br>(2) Expand the candidate

			population and speed up the enrollment
**Study Synopsis – Assessment of Efficacy**<br><br>**Section 11.2.2. Secondary Technical Efficacy Outcomes**<br><br>**Section 17.5. Analysis of Secondary Outcomes and Safety Outcomes**	1) Proportion of substantial reperfusion before endovascular treatment, as assessed on initial digital subtraction angiography. Substantial reperfusion is defined as a modified Treatment in Cerebral Infarction score of 2b (50 to 99% reperfusion) or 3 (complete reperfusion);	1) Substantial reperfusion before endovascular treatment, as assessed on initial digital subtraction angiography. Substantial reperfusion is defined as an **expanded Thrombolysis in Cerebral Infarction score** of 2b (**substantial perfusion), 2c (near-complete perfusion)** or 3 (complete reperfusion);	Updated definition, in response to a comment received from the Research Committee
**Schedule of Assessments**	Weight	Weight[1]<br>**[1]The subject's actual weight will be measured in hospital using standard hospital scales (i.e., stand up or in-bed scales if the subject is not ambulatory). If actual weight cannot be measured for any reason (due to, for example**	Clarification

		**severe illness or unavailability of in-bed scales at the site), weight will be determined by first asking the subject, second asking a family member or third by estimation.**	
**Section 11.1. Primary Efficacy Outcome**	To ensure the reliability, evaluability, and traceability of the mRS score, we will keep patients' video or voice version of follow-up at 90 days except those who die or refuse to take a video.	To ensure the reliability, evaluability, and traceability of the mRS score, we will keep patients' video or voice version of follow-up at 90 days except those who ~~die or~~ refuse to take a video.	Those who die are automatically assigned an mRS score of 6.
**Section 12.1 The Modified Rankin Scale**	Premorbid mRS status will also be obtained retrospectively~~ at 24 Hours~~	Premorbid mRS status will also be obtained retrospectively **and reported on the 24h CRF page.**	Clarification of the premorbid mRS collection time and reporting on the CRF
**Section 13.3.1. Adverse Event**		**AEs will be coded using the Medical Dictionary for Regulatory Activities coding dictionary and grouped by system organ class.**	Addition,<br>To be consistent with the Statistical Analysis Plan

**Section 13.3.2. Serious Adverse Event**		**The frequencies and incidences of SAEs occurring in subjects in the tirofiban and placebo groups will be summarized within treatment group by the Medical Dictionary for Regulatory Activities and grouped by system organ class.**	Addition, To be consistent with the Statistical Analysis Plan
**Section 24. FUNDINGS**	(2) National Natural Science Foundation of China (No. 81901236, 82071323, and 81801157).	(2) National Natural Science Foundation of China (No. **82090040**, 81901236, 82071323, and 81801157), and **(3) Army Medical University Clinical Medical Research Talent Training Program (No. 2019XLC2008 and 2019XLC3016).**	Addition
